# Supplementary material for: Mapping protein carboxymethylation sites provides insights into their role in proteostasis and cell proliferation
Source: Nat Commun. 2021 Nov 18;12:6743. doi: 10.1038/s41467-021-26982-6 (PMC8602705; doi:10.1038/s41467-021-26982-6)
Supplement: Supplementary file 1 — Supplementary information [file 41467_2021_26982_MOESM1_ESM.pdf]

Mapping protein carboxymethylation sites provides insights into their role in proteostasis and cell proliferation

Simone Di Sanzo, Katrin Spengler, Anja Leheis, Joanna M. Kirkpatrick, Theresa L. Rändler, Tim Baldensperger, Theresa Dau, Christian Henning, Luca Parca, Christian Marx, Zhao-Qi Wang, Marcus A. Glomb, Alessandro Ori and Regine Heller

Correspondence to: alessandro.ori@leibniz-fli.de or regine.heller@med.uni-jena.de

Supplementary information

This PDF includes:

Supplementary Figures 1 to 9.

Supplementary Figures

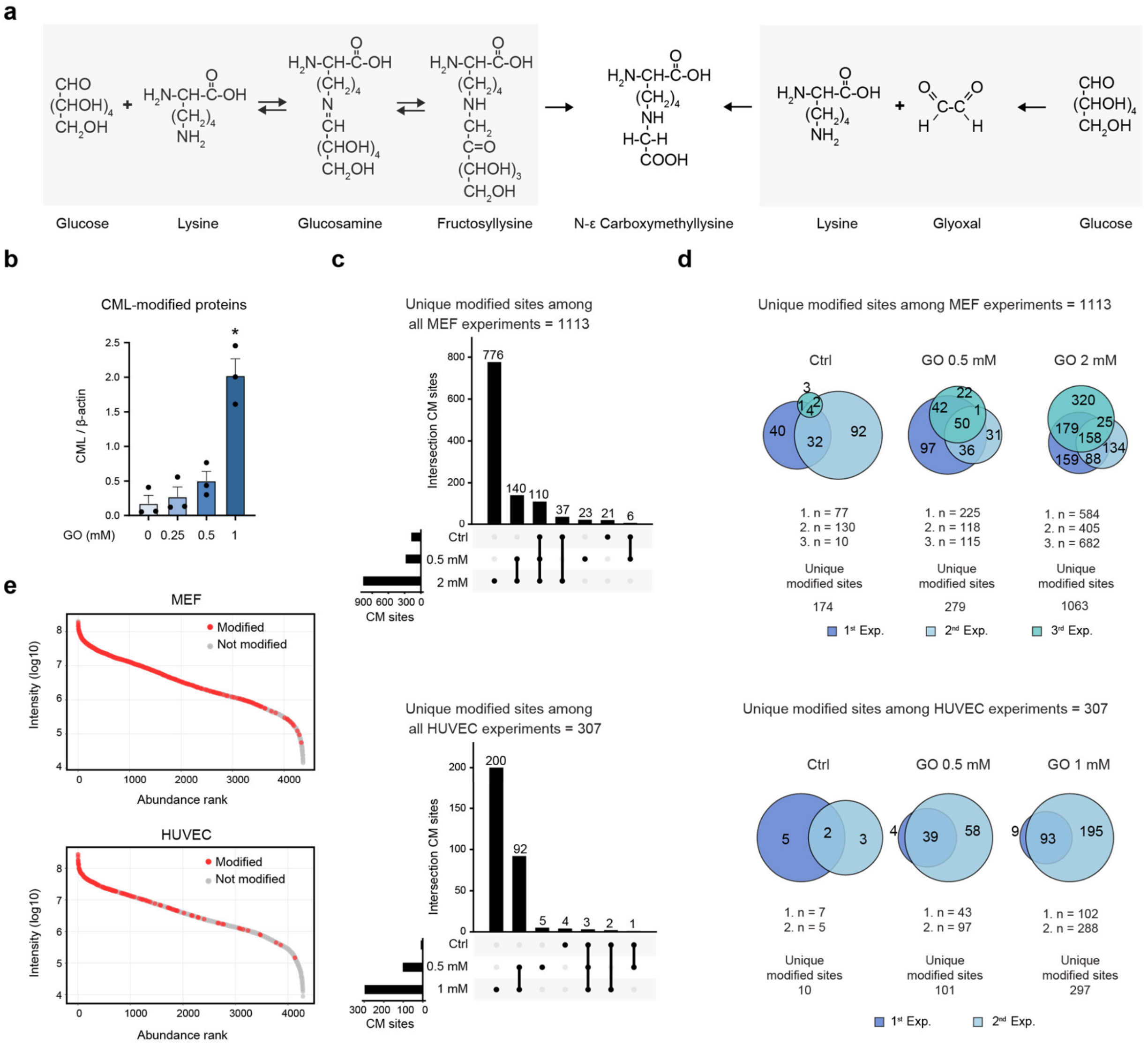

Supplementary Figure 1.

a. Simplified scheme of reaction cascade for the formation of carboxymethyllysine starting from glucose (left to right) or starting from glyoxal (right to left). **b**. HUVEC were treated with GO for 48 h and subjected to immunoblot analysis. Densitometric evaluation related to Fig. 1c is shown. n=3, mean + SEM, * $p<0.05$ *vs.* control, one-way repeated measurement ANOVA corrected via Holm-Šidák method. **c**. UpSet plot displays the intersections of carboxymethylation (CM) sites among different conditions (upper bar plot). The lower left bar plot shows the overall number of unique CM sites identified in each condition. **d**. Venn diagram representing CM sites identification across independent experiments. **e**. Rank plot showing the abundance distribution of proteins identified as targets of CM. Source data are provided as a Source Data file. Specific p values are listed in Supplementary Data 6. Related to Fig. 1 and Supplementary Data 1.

a

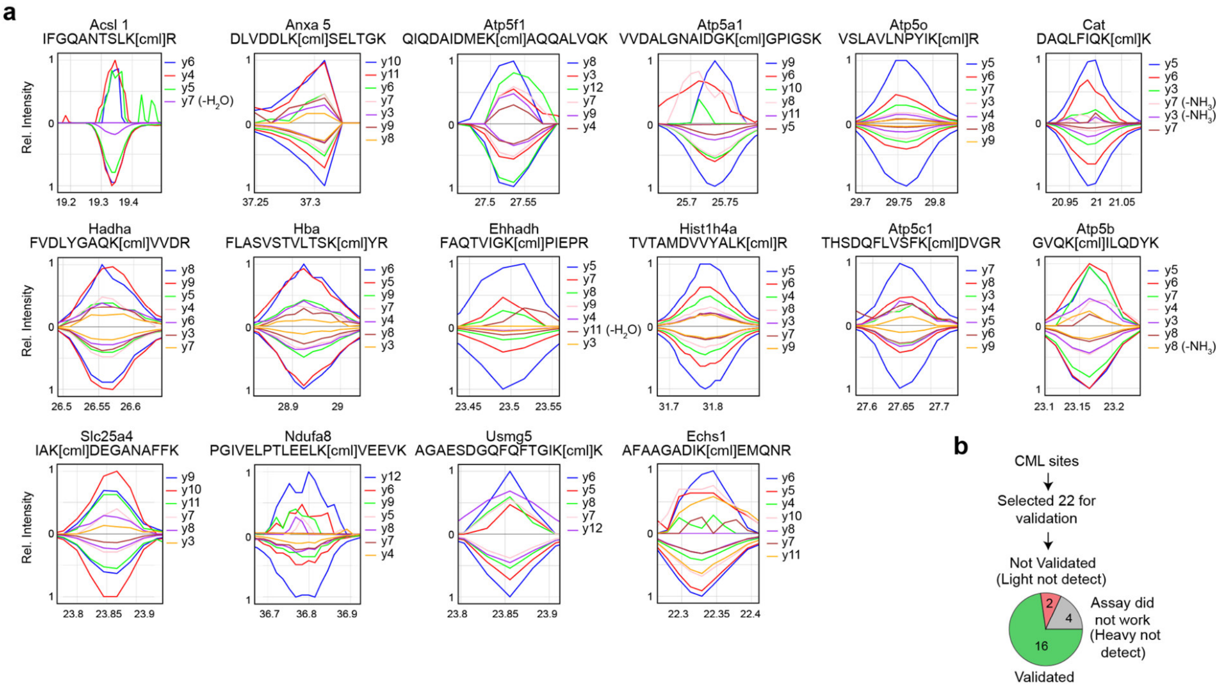

c

ID	Gene	CM site
P62806	HIST1H4A	Heart, Kidney, Liver (K92)
P43277	HIST1H1D	Heart (K64)
P10853	HIST1H2BF, HIST1H2BM, HIST1H2BB, HIST1H2BH, HIST2H2BB, HIST1H2BC, HIST1H2BK, HIST1H2BP	Heart (K47; K35)
P22752	HIST1H2AB, HIST3H2A, HIST1H2AF, HIST1H2AH, HIST1H2AK, H2AFJ	Heart, Kidney, Liver (K96)
P02301	H3F3C, H3F3A	Liver (K123)
Q64523	HIST2H2AC, HIST2H2AA1	Liver (K96)

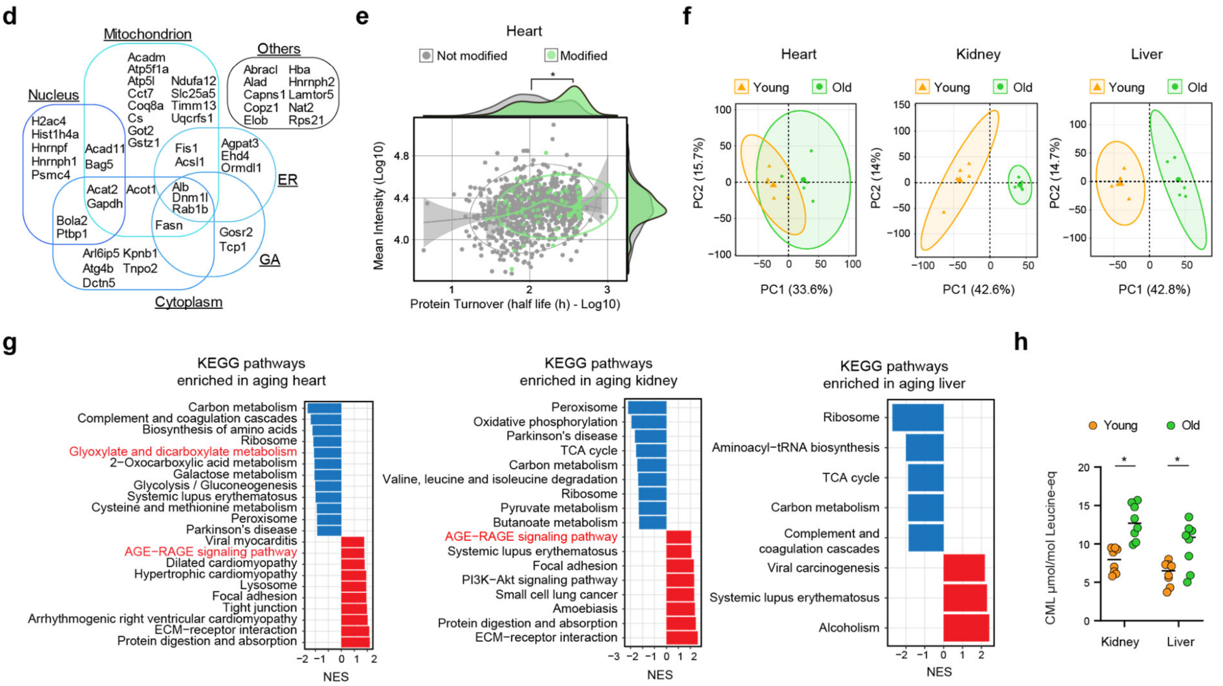

Supplementary Figure 2.

a. Validation of CML-modified peptides. Elutions from CMLpepIP were spiked with synthetic heavy peptides and analysed by parallel reaction monitoring (PRM). Transitions from endogenous (light, upper traces) and reference (heavy, bottom traces) are shown. **b**. Overview about validation of identified CML sites using parallel reaction monitoring (PRM) and heavy labelled spiked-in reference peptides. **c.** Carboxymethylation (CM) sites of histones identified by CMLpepIP in all analyzed mouse organs. **d**. Proteins identified as carboxymethylated in all analyzed mouse organs (50 proteins from the Venn diagram in Fig. 2b) divided by cell compartment as defined by Gene Ontology annotation. **e**. Scatterplot comparing protein abundance (average protein intensity) and protein turnover (half-life) of CM-modified proteins. Turnover data were taken from Fornasiero *et al*.[1]. Density plot shows the distribution of each dataset. n=5. Data were fitted with a generalized additive model and the shades represent 95 % confidence intervals * $p<0.001$, Wilcoxon Rank Sum test with continuity correction, two-sided. **f**. Principal component analysis of proteome data from young and old mice (n=5 per age group). The smaller dots represent individual samples and the larger dots the centroids of each age-matched group. Ellipses represent 95 % confidence intervals. The percentage of variance explained by the first two principal components (PC) axes is reported in the axis titles. **g**. KEGG pathway Gene Set Enrichment Analysis (GSEA) in aging tissues performed using WebGestalt[2]. Normalized enrichment score (NES) indicates pathways enriched among proteins that increase (red) or decrease (blue) with aging (FDR<0.05). All the proteins quantified in each experiment were ranked according to their log2 fold change and used as input for GSEA. **h**. Quantification of total CML levels during aging in mouse kidney and liver (Y=young, O=old). n=8, line shows the median, * $p<0.01$, unpaired multiple t-test corrected via Holm-Šidák method, two-tailed. Source data are provided as a Source Data file. Specific p values are listed in Supplementary Data 6. Related to Fig. 2 and Supplementary Data 2.

a

ID	Gene	Type	Protein name	CM site
Q9R1P4	PSMA1	26S Proteasome	Proteasome subunit alpha type-1	MEF (K30)
P54775	PSMC4	26S Proteasome	26S protease regulatory subunit 6B	MEF (K330), Heart, Kidney, Liver (ProtNterm)
Q9R1P3	PSMB2	26S Proteasome	Proteasome subunit beta type-2	Kidney (ProtNterm)
Q3TXS7	PSMD1	26S Proteasome	26S proteasome non-ATPase regulatory subunit 1	Kidney (ProtNterm)
O95352	ATG7	E1 ligase	Ubiquitin-like modifier-activating enzyme ATG7	HUVEC (K140)
Q9Z1F9	UBA2	E1 ligase	SUMO-activating enzyme subunit 2	MEF (K271), HUVEC (K271)
Q8C7R4	UBA6	E1 ligase	Ubiquitin-like modifier-activating enzyme 6	MEF (K519)
P62983	RPS27A	E1, E2, E3 ligase	Ubiquitin-40S ribosomal protein S27a	MEF (K63; K11; K6; K33), HUVEC (K63; K11; K27), Heart (K11; K48)
P63280	UBE2I	E2 ligase	SUMO-conjugating enzyme UBC9	MEF (K74)
P68037	UBE2L3	E2 ligase	Ubiquitin-conjugating enzyme E2 L3	MEF (K73)
Q6ZPJ3	UBE2O	E2 ligase	(E3-independent) E2 ubiquitin-conjugating enzyme UBE2O	MEF (K360; K368; K379; K380)
Q9Z0H7	BCL10	E3 ligase	B-cell lymphoma/leukemia 10	MEF (K115; K118)
Q7TMY8	HUWE1	E3 ligase	E3 ubiquitin-protein ligase HUWE1	MEF (K4137; K4142), HUVEC (K4134; K4139)
P46935	NEDD4	E3 ligase	E3 ubiquitin-protein ligase NEDD4	MEF (K484; K429; K186)
P17918	PCNA	E3 ligase	Proliferating cell nuclear antigen	MEF (K254; K117; K80)
P29590	PML	E3 ligase	Protein PML	HUVEC (K487; K490)
Q8BFU3	RNF214	E3 ligase	RING finger protein 214	MEF (K136; K142; K360; K366)
Q8K2Y0	RNF219	E3 ligase	RING finger protein 219	MEF (K498; K505)
Q62318	TRIM28	E3 ligase	Transcription intermediary factor 1-beta	MEF (K774; K779; K770), HUVEC (K366; K304)
Q6WKZ8	UBR2	E3 ligase	E3 ubiquitin-protein ligase UBR2	MEF (K1496)
A2AN08	UBR4	E3 ligase	E3 ubiquitin-protein ligase UBR4	MEF (K1929), HUVEC (K1931; K1060)
Q8BU04	UBR7	E3 ligase	Putative E3 ubiquitin-protein ligase UBR7	MEF (K412; K413)
Q01853	VCP	E3 ligase	Transitional endoplasmic reticulum ATPase	MEF (K236), HUVEC (K236)
Q0P5W1	VPS8	E3 ligase	Vacuolar protein sorting-associated protein 8 homolog	MEF (K1313; K1316)
Q80YR4	ZNF598	E3 ligase	Zinc finger protein 598	MEF (K94)
B2RRE7	OTUD4	Otubain Proteases	OTU domain-containing protein 4	MEF (K990)
Q80U87	USP8	Ubiquitin-Specific Proteases	Ubiquitin carboxyl-terminal hydrolase 8	MEF (K707; K713)
P70398	USP9X	Ubiquitin-Specific Proteases	Probable ubiquitin carboxyl-terminal hydrolase FAF-X	MEF (K1722; K360)
Q92995	USP13	Ubiquitin-Specific Proteases	Ubiquitin carboxyl-terminal hydrolase 13	HUVEC (K99)
Q99LD4	GPS1	UPS-associated	COP9 signalosome complex subunit 1	MEF (K204)
P56399	USP5	UPS-associated	Ubiquitin carboxyl-terminal hydrolase 5	MEF (K288; K291), HUVEC (K288; K291)

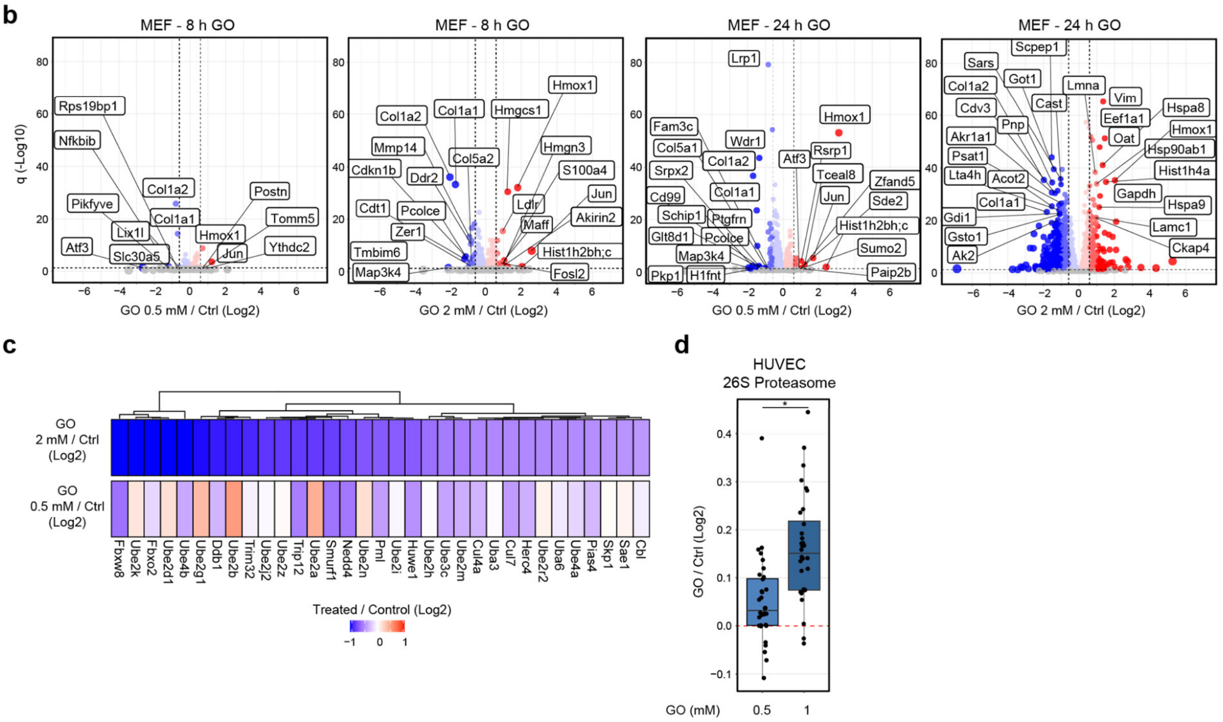

Supplementary Figure 3.

a. Table of CM sites identified on proteins related to the ubiquitin proteasome system. **b**. Protein abundance changes induced in MEF treated with glyoxal (GO). Volcano plots depict proteins that significantly increase (red) or decrease (blue) abundance upon GO treatment. Proteins not affected are shown in gray. Horizontal dashed line indicates a significance cut-off of q<0.05 and vertical dashed lines an absolute fold change (log2)>0.58. **c**. Heatmap representing the abundance of proteins involved in the ubiquitin-mediated proteolysis (Fig. 3d in red) induced in MEF by 0.5 mM and 2 mM GO after 24 h of treatment. **d**. Boxplot of fold changes for members of the 26S proteasome in HUVEC treated with GO for 48 h compared to control (Ctrl). n=4. * p<0.001, Wilcoxon Rank Sum test with continuity correction, two-sided. Source data are provided as a Source Data file. Specific p values are listed in Supplementary Data 6. Related to Fig. 3 and Supplementary Data 3.

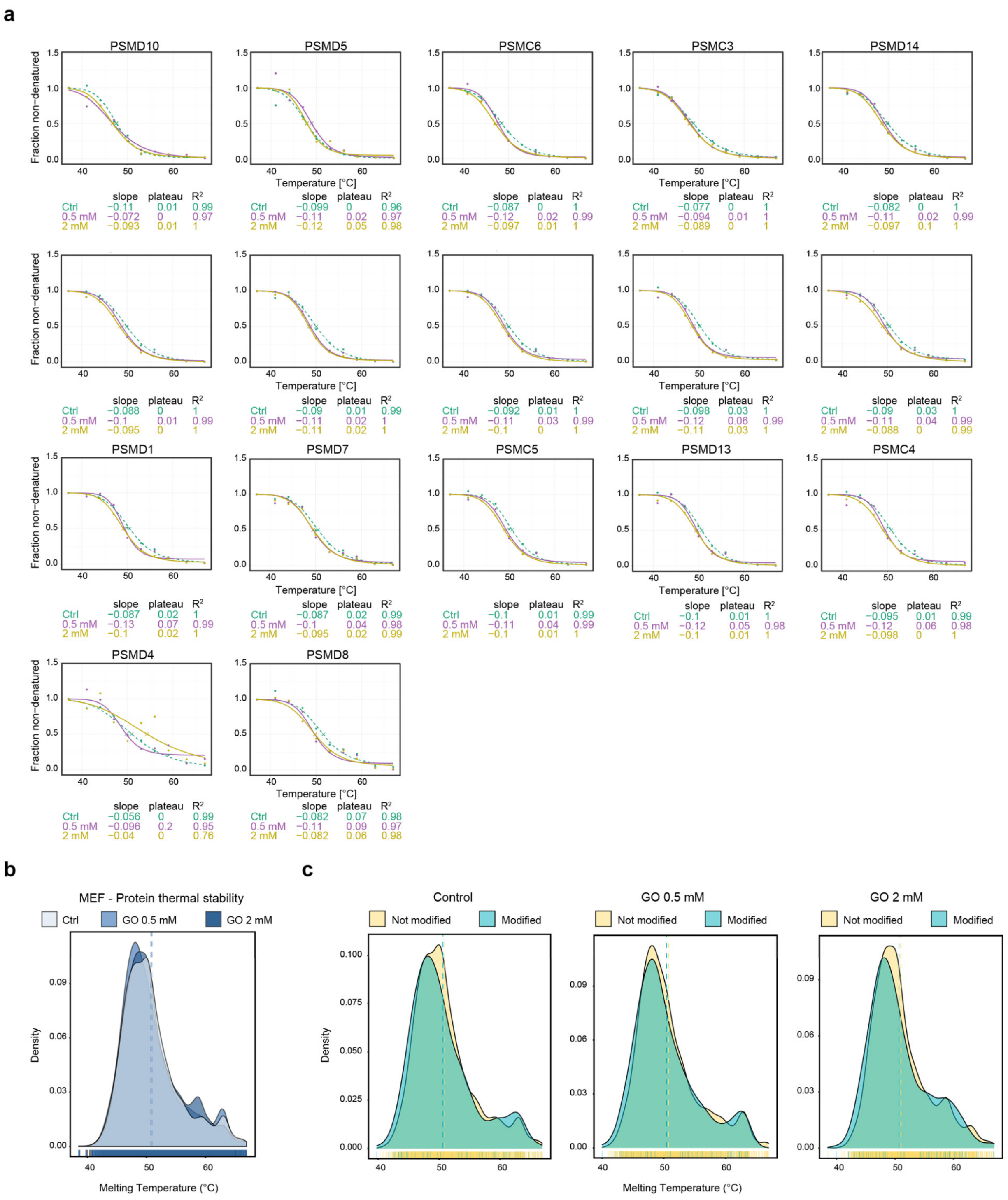

Supplementary Figure 4.

a. Melting curves of proteasome subunits represented in Fig. 3g. For each melting curve, the curve slope, the plateau and the R^2 are reported. Curves are colored based on the glyoxal (GO) treatment for 24 h: 0 mM (Ctrl, green), 0.5 mM (purple) and 2 mM (yellow). The cross, in each plot, shows at which temperature the protein reaches its 50 % of fraction

non-denaturated. **b**. Effect of GO on global protein thermal stability in MEF treated with GO for 24 h. Melting temperatures for 4367 (Ctrl), 4174 (GO 0.5 mM) and 4646 (GO 2 mM) protein groups were estimated using thermal proteome profiling (see Methods for details). **c**. Density plot representing the distribution of melting temperature of the entire dataset grouped by GO treatment (Ctrl, 0.5 mM and 2 mM). The distribution of non-modified proteins is reported in yellow, while the distribution of CM-modified proteins is shown in green. The list of modified proteins was obtained from CMLpepIP experiments performed in MEF. Dashed lines represent the group mean. Source data are provided as a Source Data file. Related to Fig. 3 and Supplementary Data 3.

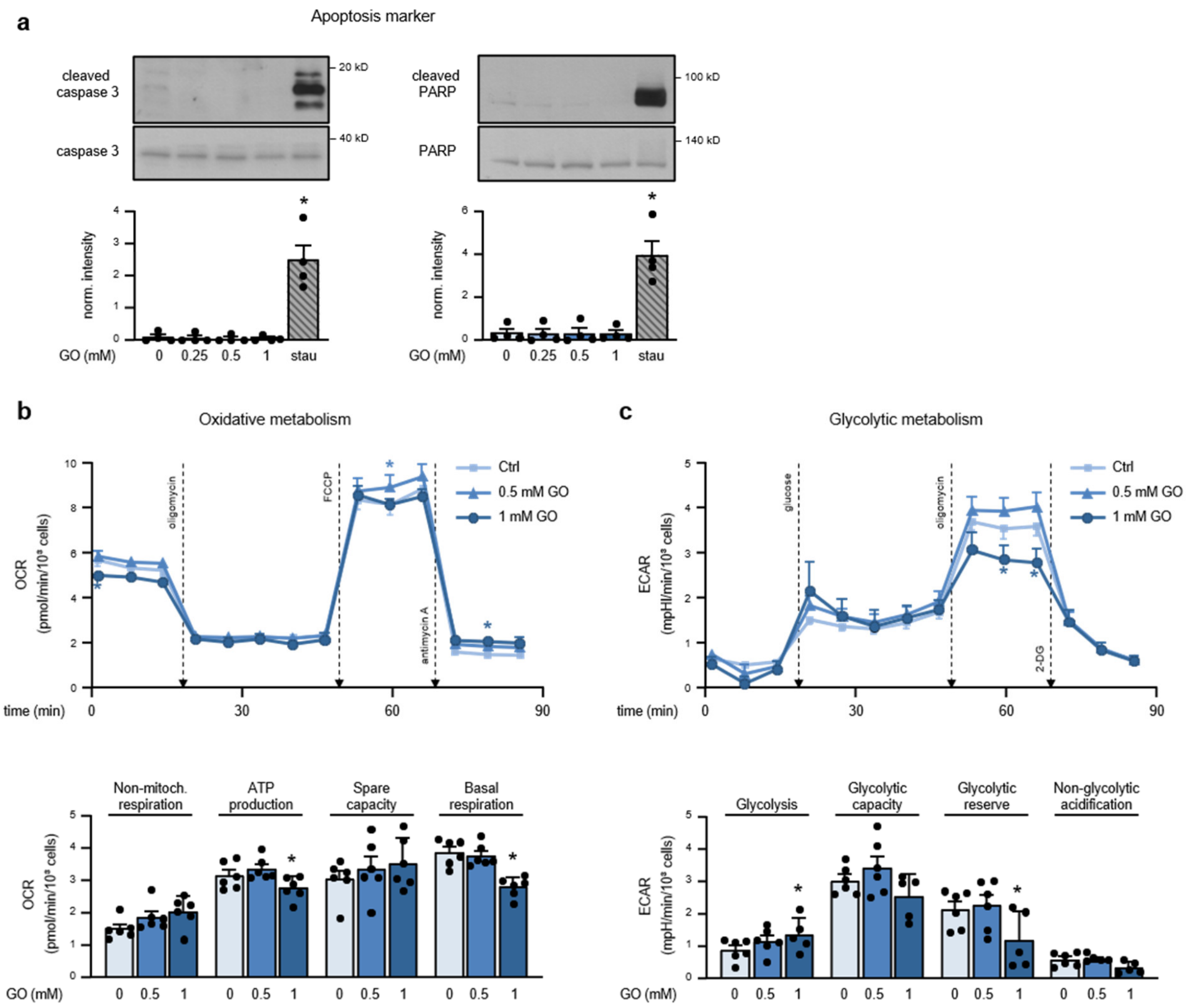

Supplementary Figure 5.

a. HUVEC were treated with the indicated concentrations of glyoxal (GO) for 48 h. The positive control was treated with 1 µM staurosporin (stau) for 2 h. Cell lysates were subjected to immunoblot analysis. Data are represented as mean + SEM. n=4, * p<0.05 *vs*. control. **b**. Mito stress test: After GO treatment (48 h) oxygen consumption rates (OCR) were measured via the Seahorse technology at baseline and after addition of pharmacological agents (oligomycin: inhibitor of mitochondrial electron transport chain (ETC) complex V, the ATP synthase; carbonyl cyanide-4-(trifluoromethoxy)phenylhydrazone (FCCP): mitochondrial uncoupling agent; antimycin A: inhibitor of ETC complex III). Non-mitochondrial respiration, ATP production, spare capacity and basal respiration were calculated from OCR values. n=6. **c**. Glycolysis stress test: After GO treatment (48 h) extracellular acidification rates (ECAR) were measured via the Seahorse technology at baseline and after addition of glucose, oligomycin and 2-deoxy-D-glucose, an inhibitor of glycolysis. Glycolytic activity, capacity and reserve, and non-glycolytic acidification were calculated from the measured ECAR data. Upper panels show the time-dependent measurement of OCR and ECAR. Lower panels display the calculated metabolic parameters. Data are represented as mean + SEM. n=6. * p<0.05 *vs.* control. Statistical significance was analyzed using one-way repeated measurement ANOVA corrected via Holm-Šidák method. Source data are provided as a Source Data file. Specific p values are listed in Supplementary Data 6. Related to Fig. 4.

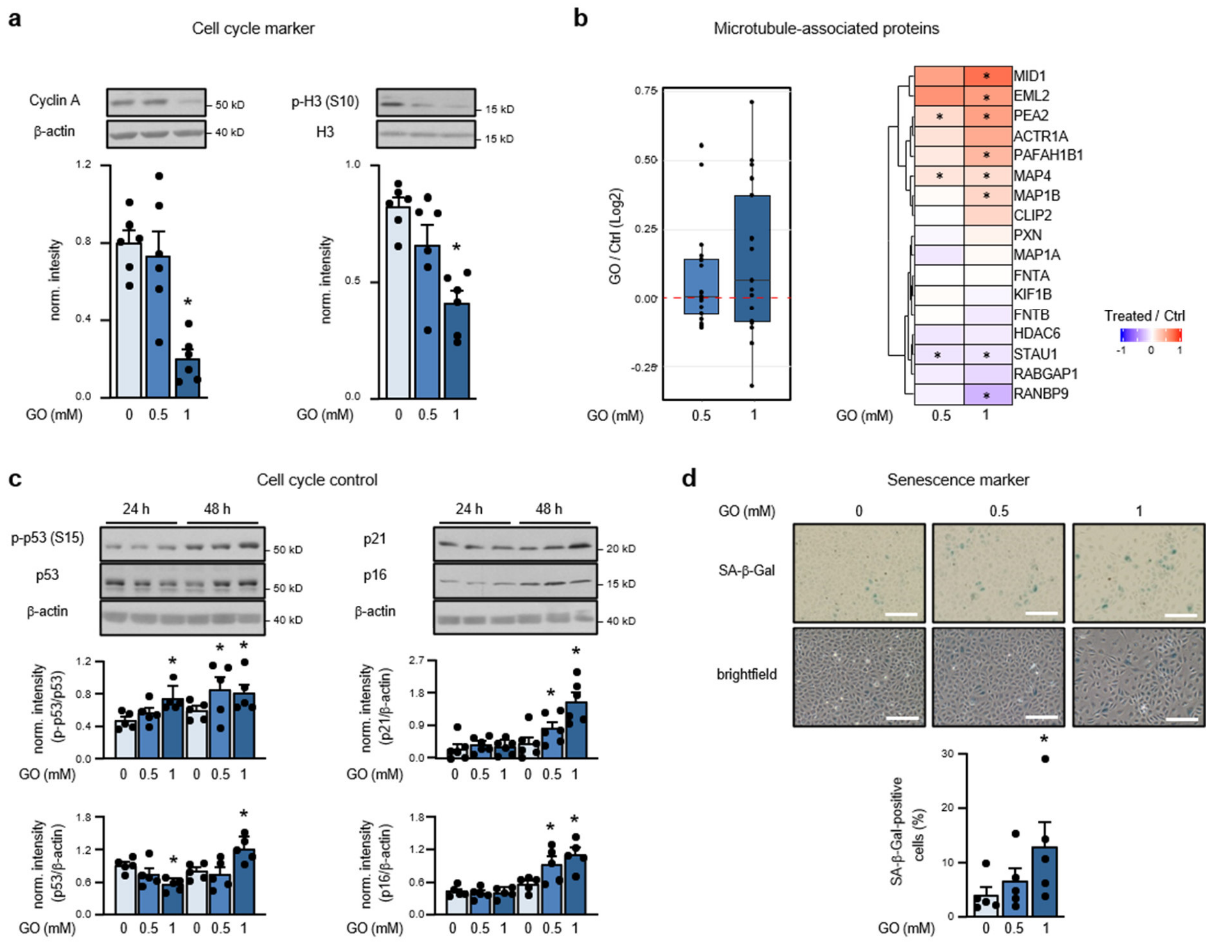

Supplementary Figure 6.

a-d. HUVEC were treated with GO at the indicated concentrations for 24 h (**a,c**) or 48 h (**b-c**). **a,c**. Cell lysates were subjected to immunoblot analysis of cell cycle markers and regulators. n=6 (**a**), n=5 (**c**), * p<0.05 *vs.* respective control. **b**. Box plot (left) and heatmap (right) show the effect of GO treatment of HUVEC on proteins assigned as "microtubule associated" from Gene Ontology Cellular Compartment annotation. n=4, * q<0.05. Ctrl: control. **d**. Senescence-associated β-galactosidase (SA-β-Gal) was stained. Representative pictures and quantification of SA-β-Gal-positive cells are shown. n=5, * p<0.05 *vs.* control. Scale bar=100 µm. **a,c,d**. Data are represented as mean + SEM. Statistical significance was analyzed using one-way repeated measurement ANOVA corrected via Holm-Šidák method. Source data are provided as a Source Data file. Specific p values are listed in Supplementary Data 6. Related to Fig. 5 and Supplementary Data 4.

a

Identified CML sites on tubulin

ID	Gene	Protein Name	CM site
P68363	TUBA1B	Tubulin alpha-1B chain	HUVEC (K394; K401; K163; K164; K326; K336; K338), MEF (K394; K401; K163; K164; K336; K338; K326; K96; K352; K370; K60)
P07437	TUBB	Tubulin beta chain	HUVEC (K379; K58)
P68371	TUBB4B	Tubulin beta-4B chain	HUVEC (K324; K58), MEF (K324; K58; K216; K336), Heart, Kidney (K324)
Q9BUF5	TUBB6	Tubulin beta-6 chain	HUVEC (K379), MEF (K57; K58)
Q13509	TUBB3	Tubulin beta-3 chain	HUVEC (K58)
P99024	TUBB5	Tubulin beta-5 chain	MEF (K379; K58; K362)

b

Matched sites of tubulin glycation

ID Porcine	Sequence	ID Human	Gene	Protein name	CM site Human
Q2XVP4	DVNAAIATIK(1)TK(1)R	P68363	TUBA1B	Tubulin alpha-1B chain	K336; K338
Q2XVP4	FDLMYAK(1)R	P68363	TUBA1B	Tubulin alpha-1B chain	K401
Q2XVP4	GDVVPK(1)DVNAAIATIK(1)TK	P68363	TUBA1B	Tubulin alpha-1B chain	K336; K326
Q2XVP4	LDHK(1)FDLMYAK	P68363	TUBA1B	Tubulin alpha-1B chain	K394
Q2XVP4	LDHK(1)FDLMYAK(1)R	P68363	TUBA1B	Tubulin alpha-1B chain	K401; K394
Q2XVP4	LSVDYGK(1)K(1)SK	P68363	TUBA1B	Tubulin alpha-1B chain	K163; K164
Q767L7	ISVYYNEATGGK(1)YVPR	P07437	TUBB	Tubulin beta chain	K58
Q767L7	MAVTFIGNSTAIQELFK(1)R	P07437	TUBB	Tubulin beta chain	K379
A0A5G2R693	ISVYYNEASSHK(1)YVPR	Q13509	TUBB3	Tubulin beta-3 chain	K58
A0A287A275	INVYYNEATGGK(1)YVPR	P68371	TUBB4B	Tubulin beta-4B chain	K58
P02554	MSMK(1)EVDEQMLNVQNK	P68371	TUBB4B	Tubulin beta-4B chain	K324

c

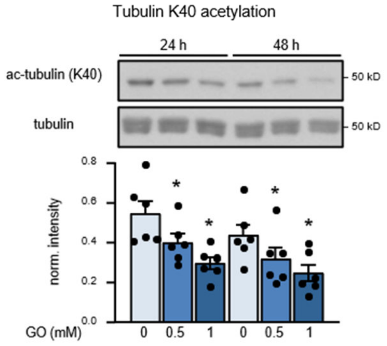

d

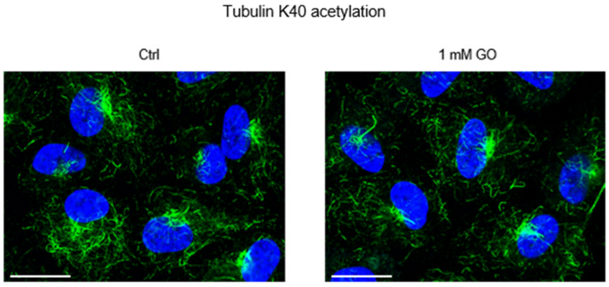

Supplementary Figure 7.

a. Table of carboxymethyllysine (CML) sites identified on tubulins either in cells treated with glyoxal (GO) or mouse organs. **b**. Table of identified CML sites matched between purified porcine tubulin and endogenous tubulin in HUVEC. **c,d**. Detection of tubulin K40 acetylation. **c**. HUVEC were treated with GO, lysed and subjected to immunoblot analysis. n=6. **d**. HUVEC were treated with GO for 24 h and stained against ac-tubulin (K40) and DAPI. A representative picture out of n=3 is shown. Scale bar=20 µm. Source data are provided as a Source Data file. Specific p values are listed in Supplementary Data 6. Related to Fig. 6 and Supplementary Data 5.

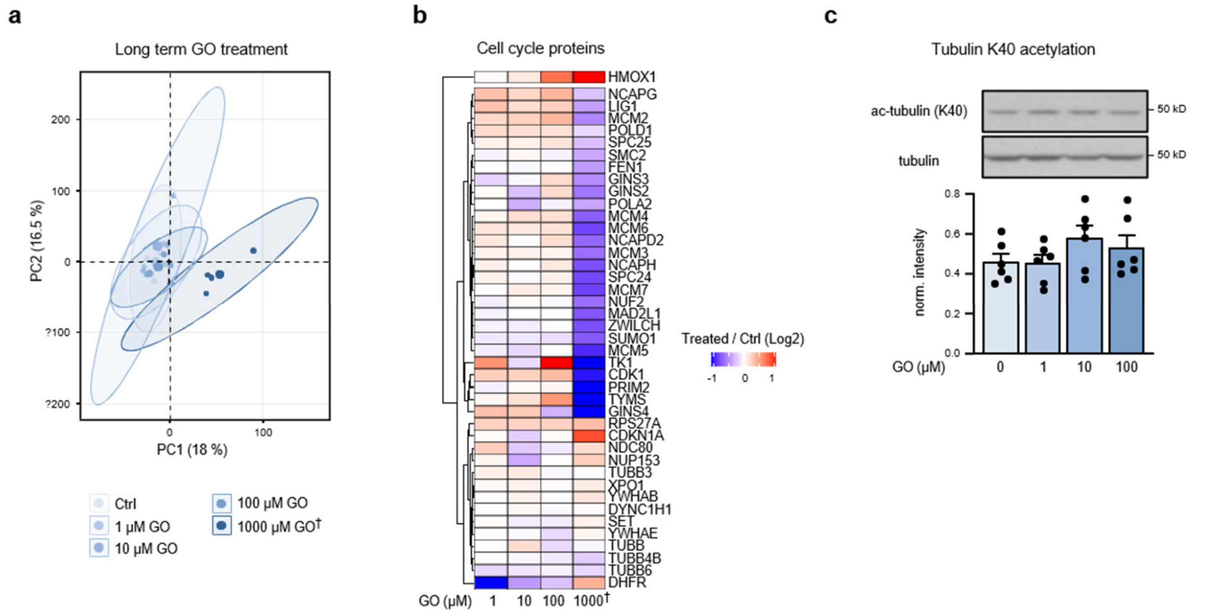

Supplementary Figure 8.

a-c. One day after seeding, 1 - 100 µM glyoxal (GO) was added to HUVEC and treatment was continued for 14 d (day 1 - 15). A treatment with 1000 µM GO for 48 h was used as positive control (†) (**a**,**b**). **a**. Principal component analysis of proteome data from HUVEC treated with 1, 10, 100, 1000† µM GO and untreated control cells (Ctrl). The smaller dots represent individual samples and the larger dots the centroids of each treated/untreated-matched group. Ellipses represent 95 % confidence intervals. The percentage of variance explained by the first two principal components (PC) axes is reported in the axis titles. n=4. **b**. Heatmap representing protein abundance changes of proteins involved in cell cycle, used for the analysis in Fig. 5c. Cell cycle proteins were selected according to Reactome annotation and filtered as significantly altered expression (absolute log2 fold change>0.58 and q<0.05) in the condition 1000 µM†. Additionally, the protein expression of Hmox1 (Heme oxygenase 1) is shown as an example of a protein consistently affected by all GO treatments. **c**. Detection of tubulin K40 acetylation. HUVEC were treated with GO, lysed and subjected to immunoblot analysis. n=6. Source data are provided as a Source Data file. Specific p values are listed in Supplementary Data 6. Related to Fig. 7 and Supplementary Data 4.

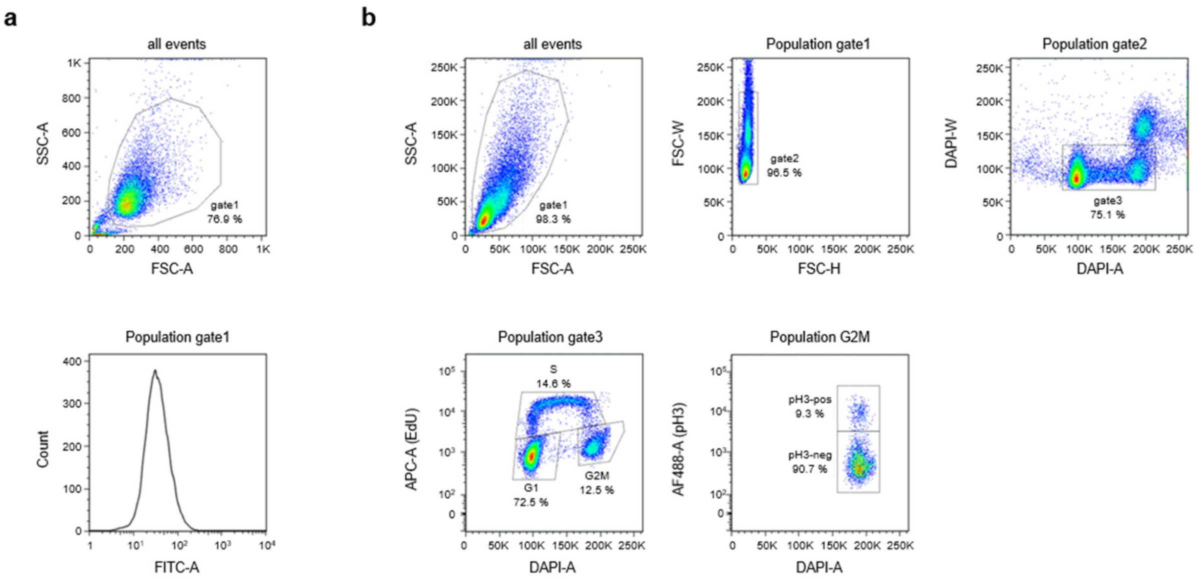

Supplementary Figure 9.

a. For monitoring MitoTracker (Fig. 4h), carboxyfluorescein succinimidyl ester (CFSE) (Fig. 5b), MitoSOX and dichlorodihydrofluorescein diacetate (H2DCFDA) (Fig. 5g,7e), cells were gated in an FSC-SSC dot blot to exclude debris. Median values of fluorescence intensity of the respective staining were evaluated. A representative dot plot with gate and its population abundances included (upper panel), and a histogram for staining intensity (lower panel) are shown. **b**. For cell cycle analysis (Fig. 5a,7b), cell populations were initially gated based on FSC/SSC to exclude debris, followed by FSC-H/FSC-W and DAPI-A/DAPI-W gating to exclude doublets (gate1-3, upper row). The resulting cells in gate3 were then gated for subpopulations of the respective cell cycle phases (G1 phase, S phase, G2M phase, lower left panel) according to their EdU (APC-A) and DAPI-A distribution. The population G2M was further gated to differentiate p-H3 (S10)-positive (M phase) and p-H3 (S10)-negative (G2 phase) cells (lower right panel). Representative dot plots with gates are shown. Cells in the respective cell cycle phases were indicated as percentages of the respective parental gates (gate 3 or G2M) and percentages finally corrected to 100 % total. Population abundances relative to the parental gates are indicated next to the gates. SSC: side scatter; FSC: forward scatter; FITC: Fluorescein isothiocyanate; DAPI: 4′,6-diamidino-2-phenylindole; Edu: 5-Ethynyl-2′-deoxyuridine; APC: allophycocyanin; AF488: Alexa Fluor 488.

Supplementary References:

1. Fornasiero EF*, et al.* Precisely measured protein lifetimes in the mouse brain reveal differences across tissues and subcellular fractions. *Nat Commun* **9**, 4230 (2018).

2. Liao Y, Wang J, Jaehnig EJ, Shi Z, Zhang B. WebGestalt 2019: gene set analysis toolkit with revamped UIs and APIs. *Nucleic Acids Res* **47**, W199-W205 (2019).
