# Supplementary material for: Mapping protein carboxymethylation sites provides insights into their role in proteostasis and cell proliferation
Source: Nat Commun. 2021 Nov 18;12:6743. doi: 10.1038/s41467-021-26982-6 (PMC8602705; doi:10.1038/s41467-021-26982-6)
Supplement: Supplementary file 3 — Description of Additional Supplementary Files [file 41467_2021_26982_MOESM3_ESM.docx]

**Description of Additional Supplementary Files**

**File Name:** Supplementary Data 1

**Description:** List of CM sites identified in MEF and HUVEC. Related to Figure 1 and Supplementary Fig. 1.

**File Name:** Supplementary Data 2

**Description:** List of CM sites identified in mouse organs and proteome changes induced by aging. Related to Figure 2 and Supplementary Fig. 2.

**File Name:** Supplementary Data 3

**Description:** Proteome and thermal stability changes induced in MEF by glyoxal treatment. Related to Figure 3 and Supplementary Fig. 3, 4.

**File Name:** Supplementary Data 4

**Description:** Proteome changes induced in HUVEC by glyoxal treatment. Related to Figure 4, 7 and Supplementary Fig. 6, 8.

**File Name:** Supplementary Data 5

**Description:** List of cell cycle-related proteins affected in HUVEC by glyoxal or directly modified by carboxymethylation. Related to Figure 5 and Supplementary Fig. 7.

**File Name:** Supplementary Data 6

**Description:** List of specific p-values. Related to all Figures.
