# Supplementary material for: Mapping protein carboxymethylation sites provides insights into their role in proteostasis and cell proliferation
Source: Nat Commun. 2021 Nov 18;12:6743. doi: 10.1038/s41467-021-26982-6 (PMC8602705; doi:10.1038/s41467-021-26982-6)
Supplement: Supplementary file 10 — Reporting Summary [file 41467_2021_26982_MOESM10_ESM.pdf]

nature research

Corresponding author(s): Alessandro Ori
Regine Heller

Last updated by author(s): Oct 28, 2021

Reporting Summary

Nature Research wishes to improve the reproducibility of the work that we publish. This form provides structure for consistency and transparency in reporting. For further information on Nature Research policies, see our Editorial Policies and the Editorial Policy Checklist.

Statistics

For all statistical analyses, confirm that the following items are present in the figure legend, table legend, main text, or Methods section.

n/a	Confirmed	
☐	☒	The exact sample size (*n*) for each experimental group/condition, given as a discrete number and unit of measurement
☐	☒	A statement on whether measurements were taken from distinct samples or whether the same sample was measured repeatedly
☐	☒	The statistical test(s) used AND whether they are one- or two-sided<br>*Only common tests should be described solely by name; describe more complex techniques in the Methods section.*
☒	☐	A description of all covariates tested
☐	☒	A description of any assumptions or corrections, such as tests of normality and adjustment for multiple comparisons
☐	☒	A full description of the statistical parameters including central tendency (e.g. means) or other basic estimates (e.g. regression coefficient) AND variation (e.g. standard deviation) or associated estimates of uncertainty (e.g. confidence intervals)
☐	☒	For null hypothesis testing, the test statistic (e.g. F, t, r) with confidence intervals, effect sizes, degrees of freedom and P value noted<br>*Give P values as exact values whenever suitable.*
☒	☐	For Bayesian analysis, information on the choice of priors and Markov chain Monte Carlo settings
☒	☐	For hierarchical and complex designs, identification of the appropriate level for tests and full reporting of outcomes
☒	☐	Estimates of effect sizes (e.g. Cohen's d, Pearson's r), indicating how they were calculated

Our web collection on statistics for biologists contains articles on many of the points above.

Software and code

Policy information about availability of computer code

Data collection	Xcalibur 4.0 or 4.1 and Tune version 2.1 or 2.9 (both Thermo Fisher) for mass spectrometry data, BD FACSDIVA-Software v 7.0 (Becton, Dickinson and Company) for Flow Cytometry data
Data analysis	Spectronaut Professional (v.10-15, Biognosys AG), Spectrodive (v.9-10, Biognosys AG), Spectromine v.2 (Biognosys AG), Proteome Discoverer v2.0 (Thermo Fisher Scientific, Waltham, MA, USA), Mascot v2.5.1 (Matrix Science), R (v.3.6.3), R studio server (v. 1.2.5042), R packages: MSnbase v2.12.0, limma v3.42.2, TPP v3.20.1, Plotly v4.9.4.1, WebGestaltR v0.4.4; isobarquant v1.1.0, Cytoscape v3.7.2, clueGO v2.5.6, Fiji ImageJ 1.52p (NIH), FlowJo 7.6.5 (Becton, Dickinson and Company), SigmaPlot 14.0 (Systat Software GmbH), GraphPad Prism (GraphPad Software, LLC, v8.3.0)

For manuscripts utilizing custom algorithms or software that are central to the research but not yet described in published literature, software must be made available to editors and reviewers. We strongly encourage code deposition in a community repository (e.g. GitHub). See the Nature Research guidelines for submitting code & software for further information.

Data

Policy information about availability of data

All manuscripts must include a data availability statement. This statement should provide the following information, where applicable:

- Accession codes, unique identifiers, or web links for publicly available datasets
- A list of figures that have associated raw data
- A description of any restrictions on data availability

The source data of all analyses are supplied as Supplementary Data 1–5 and in the associated source data file. The mass spectrometry data generated in this study have been deposited to the ProteomeXchange Consortium via the PRIDE partner repository under accession code PXD027526 [https://www.ebi.ac.uk/pride/archive/projects/PXD027526] (DDA data for CMLpepIP);

PXD021883 [https://www.ebi.ac.uk/pride/archive/projects/PXD021883] (TMT-10plex data for organ aging proteome);
PXD021985 [https://www.ebi.ac.k/pride/archive/projects/PXD021985] (DIA for MEF and HUVEC cells);
PXD021891 [https://www.ebi.ac.uk/pride/archive/projects/PXD021891] (DIA for acetylated peptides);
PXD027468 [https://www.ebi.ac.uk/pride/archive/projects/PXD027468] (DIA for long-term treatment with low-doses of glyoxal).
The Uniprot protein databases used for proteomic analysis are accessible at: https://ftp.uniprot.org/pub/databases/uniprot/previous_releases/release-2016_01/
In addition, the proteomics data presented in this manuscript are available via a R shiny web server [https://genome.leibniz-fli.de/shiny/orilab/CMLsites/]

Field-specific reporting

Please select the one below that is the best fit for your research. If you are not sure, read the appropriate sections before making your selection.

☒ Life sciences ☐ Behavioural & social sciences ☐ Ecological, evolutionary & environmental sciences

For a reference copy of the document with all sections, see nature.com/documents/nr-reporting-summary-flat.pdf

Life sciences study design

All studies must disclose on these points even when the disclosure is negative.

Sample size	For proteomics analysis a sample size of 3-8 was used (Gebert et al. Cell Reports 2020, Sacramento et al., Mol Sys Biol 2020). This was determined by statistical tests performed with similar experiments in studies performed earlier. For functional assays, observed differences were tested for statically significance with a sample size of 4-6 based on previous experiences. In most cases, parameters were investigated after treatment with different concentrations of glyoxal and at different incubation times as well as with different methodological approaches thus verifying a certain effect under multiple conditions.
Data exclusions	Few replicates of mouse organ lysates (Figure 2h) were excluded due to poor quality of the loading control.
Replication	Typically, 3-6 biological replicates per experiment were performed. All attempts at replication were successful.
Randomization	Not relevant for mice studies as only animals of different age groups were compared.<br>Experimental series for MEF and HUVEC were performed several times with different cell batches (HUVEC) or passages (MEF) for biological replicates. In each experiment, controls and treated samples were run in parallel as paired samples. Randomization was not relevant.
Blinding	Immunofluorescence pictures (tubulin stainings) were evaluated in a blinded approach by a person, who was not involved in performing experiments, stainings, coding/decoding and randomization of data. Evaluation involved counting of strongly structured tubulin filaments per high-power field and normalization to the number of DAPI-stained nuclei.<br>For biochemical experiments, i.e., immunoblots, activity assays, mass spectrometry analyses, blinding was not performed since these experiments did not involve counting but rather direct read outs of analyte levels or activity.

Reporting for specific materials, systems and methods

We require information from authors about some types of materials, experimental systems and methods used in many studies. Here, indicate whether each material, system or method listed is relevant to your study. If you are not sure if a list item applies to your research, read the appropriate section before selecting a response.

Materials & experimental systems

n/a	Involved in the study	
☐	☒	Antibodies
☐	☒	Eukaryotic cell lines
☒	☐	Palaeontology and archaeology
☐	☒	Animals and other organisms
☐	☒	Human research participants
☒	☐	Clinical data
☒	☐	Dual use research of concern

Methods

n/a	Involved in the study	
☒	☐	ChIP-seq
☐	☒	Flow cytometry
☒	☐	MRI-based neuroimaging

Antibodies

Antibodies used	β-actin (13E5) (CST #4970), suitability for application (western blot) and species (human) was tested and specified by the manufacturer, used for HUVEC samples, dilution 1:5000 used since the recommended 1:1000 gave too strong signals (https://www.cellsignal.de/products/primary-antibodies/b-actin-13e5-rabbit-mab/4970)<br><br>β-actin (AC-15) (Sigma #A5441), suitability for application (western blot) was tested and specified by the manufacturer, used for MEF samples according to a previous publication (Bunnell et al., 2011, PMID 21900491) in a dilution of 1:2000 (https://www.sigmaaldrich.com/DE/de/product/sigma/a5441)

Chk1 (2G1D5) (CST #2360), suitability for application (western blot) and species (human) was tested and specified by the manufacturer, dilution 1:1000 was used
(https://www.cellsignal.de/products/primary-antibodies/chk1-2g1d5-mouse-mab/2360)

p-Chk1 (S317) (D12H3) (CST #12302), suitability for application (western blot) and species (human) was tested and specified by the manufacturer, dilution 1:1000 was used
(https://www.cellsignal.de/products/primary-antibodies/phospho-chk1-ser317-d12h3-xp-rabbit-mab/12302)

p-Chk1 (S345) (133D3) (CST #2348), suitability for application (western blot) and species (human) was tested and specified by the manufacturer, dilution 1:1000 was used,
(https://www.cellsignal.de/products/primary-antibodies/phospho-chk1-ser345-133d3-rabbit-mab/2348)

cleaved caspase 3 (D175) (5A1E) (CST #9664), suitability for application (western blot) and species (human) was tested and specified by the manufacturer, dilution 1:1000 was used
(https://www.cellsignal.de/products/primary-antibodies/cleaved-caspase-3-asp175-5a1e-rabbit-mab/9664)

caspase 3 (8G10) (CST #9665), suitability for application (western blot) and species (human) was tested and specified by the manufacturer, dilution 1:1000 was used, product discontinued
(https://www.cellsignal.de/products/primary-antibodies/caspase-3-8g10-rabbit-mab/9665)

CML (CML26) (abcam #ab125145), applied in western blot, suitability for western blots was tested and specified by the manufacturer, antibody reacts species-independent, dilution 1:10000 was used
(https://www.abcam.com/carboxymethyl-lysine-antibody-cml26-ab125145.html)

CML (ImmuneChem Pharmaceuticals #CN1040), applied for immunoprecipitation, suitability for immunoprecipitation and species (mouse and human) was tested by the user, validation was performed by testing the enrichment with and without antibody
(https://www.immunechem.com/?app=product&act=look&type_id=8&id=125)

cyclin A (H432) (Santa Cruz Biotechnology #sc-751), suitability for application (western blot) and species (human) was tested and specified by the manufacturer, dilution 1:500 was used, product discontinued
(https://datasheets.scbt.com/sc-751.pdf)

γH2A.X (S139) (20E3) (CST #9718), suitability for application (western blot) and species (human) was tested and specified by the manufacturer, dilution 1:1000 was used
(https://www.cellsignal.de/products/primary-antibodies/phospho-histone-h2a-x-ser139-20e3-rabbit-mab/9718)

histone 3 (D1H2) (CST #4499), suitability for application (western blot) and species (human) was tested and specified by the manufacturer, dilution 1:10000 was used since the recommended 1:2000 gave too strong signals
(https://www.cellsignal.de/products/primary-antibodies/histone-h3-d1h2-xp-rabbit-mab/4499)

p-H3 (S10) (abcam #ab47297), applied in western blots, suitability for western blot and species (human) was tested and specified by the manufacturer, dilution 1:1000 was used,
(https://www.abcam.com/histone-h3-phospho-s10-antibody-ab47297.html)

p-H3 (S10) (Millipore #06-570), applied in flow cytometry, suitability for species (human) was tested and specified by the manufacturer, the antibody was used for flow cytometry according to a previous publication (Hauge et al., 2017, PMID 28030798) in a dilution of 1:200
(https://www.merckmillipore.com/DE/de/product/Anti-phospho-Histone-H3-Ser10-Antibody-Mitosis-Marker,MM_NF-06-570)

acetyl-lysine (agarose-conjugate) (ImmuneChem Pharmaceuticals #ICP0388), suitability for application (immunoprecipitation) was tested and specified by the manufacturer, antibody reacts species-independent,
(https://www.immunechem.com/?app=product&act=look&type_id=5&id=4)

cleaved PARP (D214) (D64E10) (CST #5625), suitability for application (western blot) and species (human) was tested and specified by the manufacturer, dilution 1:1000 was used
(https://www.cellsignal.de/products/primary-antibodies/cleaved-parp-asp214-d64e10-xp-rabbit-mab/5625)

p16 (BD Pharmingen #551154), suitability for application (western blot) and species (human) was tested and specified by the manufacturer, dilution 1:500 was used
(https://www.bdbiosciences.com/en-de/products/reagents/microscopy-imaging-reagents/immunohistochemistry-reagents/purified-mouse-anti-human-p16-with-control.551154)

p21 (12D1) (CST #2947), suitability for application (western blot) and species (human) was tested and specified by the manufacturer, dilution 1:1000 was used
(https://www.cellsignal.de/products/primary-antibodies/p21-waf1-cip1-12d1-rabbit-mab/2947)

p53 (1C12) (CST #2524), suitability for application (western blot) and species (human) was tested and specified by the manufacturer, dilution 1:1000 was used
(https://www.cellsignal.de/products/primary-antibodies/p53-1c12-mouse-mab/2524)

p-p53 (S15) (CST #9284), suitability for application (western blot) and species (human) was tested and specified by the manufacturer, dilution 1:1000 was used
(https://www.cellsignal.de/products/primary-antibodies/phospho-p53-ser15-antibody/9284)

PARP (46D11) (CST #9532), suitability for application (western blot) and species (human) was tested and specified by the manufacturer, dilution 1:1000 was used
(https://www.cellsignal.de/products/primary-antibodies/parp-46d11-rabbit-mab/9532)

proteasomal subunit 20S alpha 1-7 (abcam #ab22674), antibody was used for western blots of human proteasome in a dilution of 1:1000 according to a previous publication (Welk et al., 2016, PMID 27129254)
(https://www.abcam.com/proteasome-20s-alpha-123567-antibody-mcp231-ab22674.html)

α/ß-tubulin (CST #2148), suitability for application (western blot) and species (human) was tested and specified by the manufacturer, application for purified porcine tubulin was tested by the user, dilution 1:2000 was used since the recommended 1:1000 gave too strong signals
(https://www.cellsignal.de/products/primary-antibodies/a-b-tubulin-antibody/2148)

ac-tubulin (K40) (D20G3) (CST #5335), suitability for application (western blot) and species (human) was tested and specified by the manufacturer, dilution 1:1000 was used
(https://www.cellsignal.de/products/primary-antibodies/acetyl-a-tubulin-lys40-d20g3-xp-rabbit-mab/5335)

mono- and polyubiquitinylated conjugates (FK2) (Enzo Life Sciences #ENZ-ABS840), suitability for application (western blot) was tested and specified by the manufacturer, antibody reacts species-independent, dilution 1:2000 was used since the recommended 1:1000 gave too strong signals
(https://www.enzolifesciences.com/ENZ-ABS840)

vinculin (CST #4650), suitability for application (western blot) and species (human) was tested and specified by the manufacturer, dilution 1:1000 was used
(https://www.cellsignal.de/products/primary-antibodies/vinculin-antibody/4650)

CST: Cell Signaling Technology

Validation	In this study, only commercially available antibodies were used. All antibodies were applied according to the manufacturers' specifications (applications, species reactivity, sensitivity) as stated on the suppliers' websites. In several cases, an additional validation was performed by demonstrating the effect of positive controls (Figure 3f, ubiquitin; Figure S5a, cleaved caspase 3).

Eukaryotic cell lines

Policy information about cell lines

Cell line source(s)	Human umbilical vein endothelial cells (HUVEC) were isolated from anonymously acquired human umbilical cords according to the "Ethical principles for Medical Research Involving Human Subjects" (Declaration of Helsinki 1964). The donors were informed and gave written consent. SV40-immortalized mouse embryonic fibroblasts (MEF) were a kind gift of K. L. Rudolph (Leibniz Institute on Aging - Fritz Lipman Institute, Jena).
Authentication	Purity of primary HUVEC culture was confirmed by flow cytometric detection of endothelial markers (CD31) (>98 % positive). Primary MEF were not authenticated.
Mycoplasma contamination	MEF were tested negative for mycoplasma contamination. HUVEC were used in experiments within 10 to 14 days after isolation and disposed thereafter. Mycoplasma testing was not performed due to short culture.
Commonly misidentified lines (See ICLAC register)	none

Animals and other organisms

Policy information about studies involving animals; ARRIVE guidelines recommended for reporting animal research

Laboratory animals	C57BL/6J male, young (3-4 months), old (26-33 months).<br>All animals were kept in a specific pathogen-free animal facility with a 12 h light/dark cycle, at a temperature of 20 °C ± 2 and humidity of 55 % ± 15.
Wild animals	No wild animals were used in the study.
Field-collected samples	No field-collected samples were used in the study.
Ethics oversight	The protocols of animal maintenance and euthanasia were approved by the local authorities for animal welfare in the State of Thuringia (Thüringer Landesamt für Verbraucherschutz), Germany.

Note that full information on the approval of the study protocol must also be provided in the manuscript.

Human research participants

Policy information about studies involving human research participants

Population characteristics	Umbilical cords were collected anonymously from healthy donors after giving their consent. Age of donors was not considered.
Recruitment	Donors were not recruited.
Ethics oversight	The study protocol was approved by the Jena University Hospital Ethics Committee.

Note that full information on the approval of the study protocol must also be provided in the manuscript.

Flow Cytometry

Plots

Confirm that:

- [x] The axis labels state the marker and fluorochrome used (e.g. CD4-FITC).
- [x] The axis scales are clearly visible. Include numbers along axes only for bottom left plot of group (a 'group' is an analysis of identical markers).
- [x] All plots are contour plots with outliers or pseudocolor plots.
- [x] A numerical value for number of cells or percentage (with statistics) is provided.

Methodology

Sample preparation	For monitoring fluorecence of MitoTracker, MitoSOX, carboxyfluorescein succinimidyl ester (CSFE), dichlorodihydrofluorescein diacetate (H2DCFDA), cells were incubated with the respective dyes, washed with PBS, detached with trypsin/EDTA and transferred to HEPES containing 10% fetal calf serum (FCS). The cell suspension was pooled with HEPES/FCS obtained from rinsing the dish. 10 ml HEPES buffer were added and cells centrifuged (500 x g, 1 min). Cell pellets were resuspended in 300 µl PBS and subjected to flow cytometry analysis. Median values were evaluated using the FlowJo™ software.<br><br>For investigating cell cycle distribution via a triple staining approach, cells were incubated with 10 µM 5-ethynyl-2´-deoxyuridine (EdU), washed twice with PBS, detached with trypsin/EDTA and transferred to HEPES/FCS. The cell suspension was pooled with HEPES/FCS rinsing solution and samples were centrifuged. All centrifugations were carried out at 500 x g for 3 min at room temperature (RT) if not otherwise stated.<br>Cell pellets were washed once in BSA buffer (1 % BSA in PBS), resuspended in 100 µl of the same buffer, fixed with 100 µl of 4 % paraformaldehyde for 15 min, centrifuged and resuspended in 300 µl PBS. 700 µl of 100 % ethanol were added dropwise under constant gentle shaking and samples were frozen overnight. The next day, cells were centrifuged (700 x g, 3 min, RT), washed in 1 ml BSA buffer and permeabilized in 100 µl Triton-based BSA buffer (TBB, 0.2 % Triton X-100 in BSA buffer) for 30 min. Then, cells were centrifuged, resuspended in 500 µl BSA buffer, incubated for 1 h, centrifuged again and incubated in 150 µl of primary antibody solution (1:200 p-H3 (S10) antibody in TBB) for 2 h. This was followed by another addition of 500 µl TBB, centrifugation and incubation of cells in 150 µl of secondary antibody solution (1:500 AF488 goat anti-rabbit antibody in TBB) for 1 h. Next, after adding 500 µl TBB, centrifugation and an additional washing with 1 ml BSA buffer, cells were incubated in 100 µl of Click-iT reaction cocktail (2.5 mM CuSO4, 1:200 AF647 azide, 50 mM sodium ascorbate in PBS) for 30 min. Subsequently, another washing with 1 ml BSA buffer was performed and cells were incubated in 300 µl BSA buffer containing 1 µg DAPI for 30 min. Samples were subjected to flow cytometric analysis with triple detection of AF488, AF647 and DAPI. Percentages of cells in the respective cell cycle phases were evaluated using the FlowJo™ software.
Instrument	BD FACSCanto™ Flow Cytometer (Becton, Dickinson and Company) for monitoring fluorecence of MitoTracker, MitoSOX, carboxyfluorescein succinimidyl ester (CSFE), dichlorodihydrofluorescein diacetate (H2DCFDA); BD FACSCanto II (Becton, Dickinson and Company) equipped with a violet (405 nm; filter set for the DAPI channel - 450/50BP), blue (488 nm; filter set for the FITC/AF488 channel - 530/30BP; 502LP) and red (633 nm; filter set for the APC channel - 660/20BP) filter set for cell cycle analysis.
Software	BD FACSDIVA-Software v 7.0 (Becton, Dickinson and Company)<br>FlowJo v7.6.5 (Becton, Dickinson and Company)
Cell population abundance	In this study, primary endothelial cell population of high purity (>98 % as detected by CD31 staining) were employed. Seeding and culture conditions were comparable for all experiments and thus, flow cytometry analyses were performed with high abundance cell populations, which were comparable between individual experiments (> 300,000 cells/condition, > 20.000 cells counted). Gating of subpopulations, e.g. for evaluating distribution of cells in cell cycle phases, involved analyses of low abundance populations, which were , however, comparable between control and treatment conditions.

Gating strategy	For monitoring fluorescence of MitoTracker, MitoSOX, carboxyfluorescein succinimidyl ester (CSFE), dichlorodihydrofluorescein diacetate (H2DCFDA), gating in FSC-SSC dot blots was performed to exclude cell debris.<br>For investigating cell cycle distribution via triple staining method, a sequential gating strategy was applied with (1) exclusion of debris and cell aggregates, (2) gating for subpopulations according to distribution of cells in G1, S and G2M phases and (3) further gating of cells in G2M phase according to their positive/negative staining for a mitotic marker. This strategy is described in Figure S9 (supplementary figures).

☒ Tick this box to confirm that a figure exemplifying the gating strategy is provided in the Supplementary Information.
