# Supplementary material for: Mapping protein carboxymethylation sites provides insights into their role in proteostasis and cell proliferation
Source: Nat Commun. 2021 Nov 18;12:6743. doi: 10.1038/s41467-021-26982-6 (PMC8602705; doi:10.1038/s41467-021-26982-6)
Supplement: Supplementary file 11 — Source Data [file 41467_2021_26982_MOESM11_ESM.zip › Figure 2/2E/Layout 1.pdf]

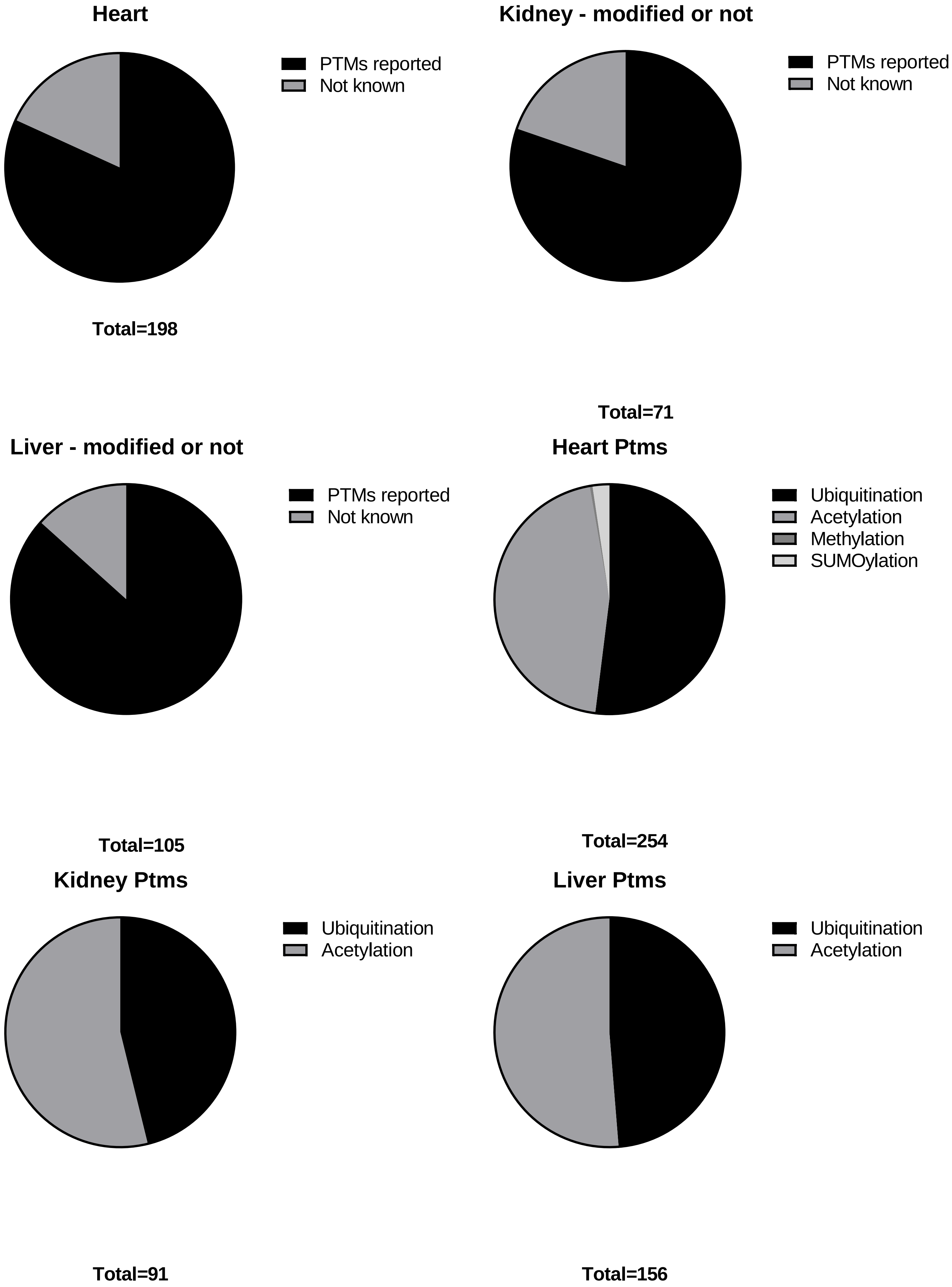
Heart
PTMs reported
Not known
Total=198
Kidney - modified or not
PTMs reported
Not known
Total=71
Liver - modified or not
PTMs reported
Not known
Total=105
Heart Ptms
Ubiquitination
Acetylation
Methylation
SUMOylation
Total=254
Kidney Ptms
Ubiquitination
Acetylation
Total=91
Liver Ptms
Ubiquitination
Acetylation
Total=156
