# Supplementary material for: Mapping protein carboxymethylation sites provides insights into their role in proteostasis and cell proliferation
Source: Nat Commun. 2021 Nov 18;12:6743. doi: 10.1038/s41467-021-26982-6 (PMC8602705; doi:10.1038/s41467-021-26982-6)
Supplement: Supplementary file 11 — Source Data [file 41467_2021_26982_MOESM11_ESM.zip › Figure 4/4B/Figure4_B_bottom.pdf]

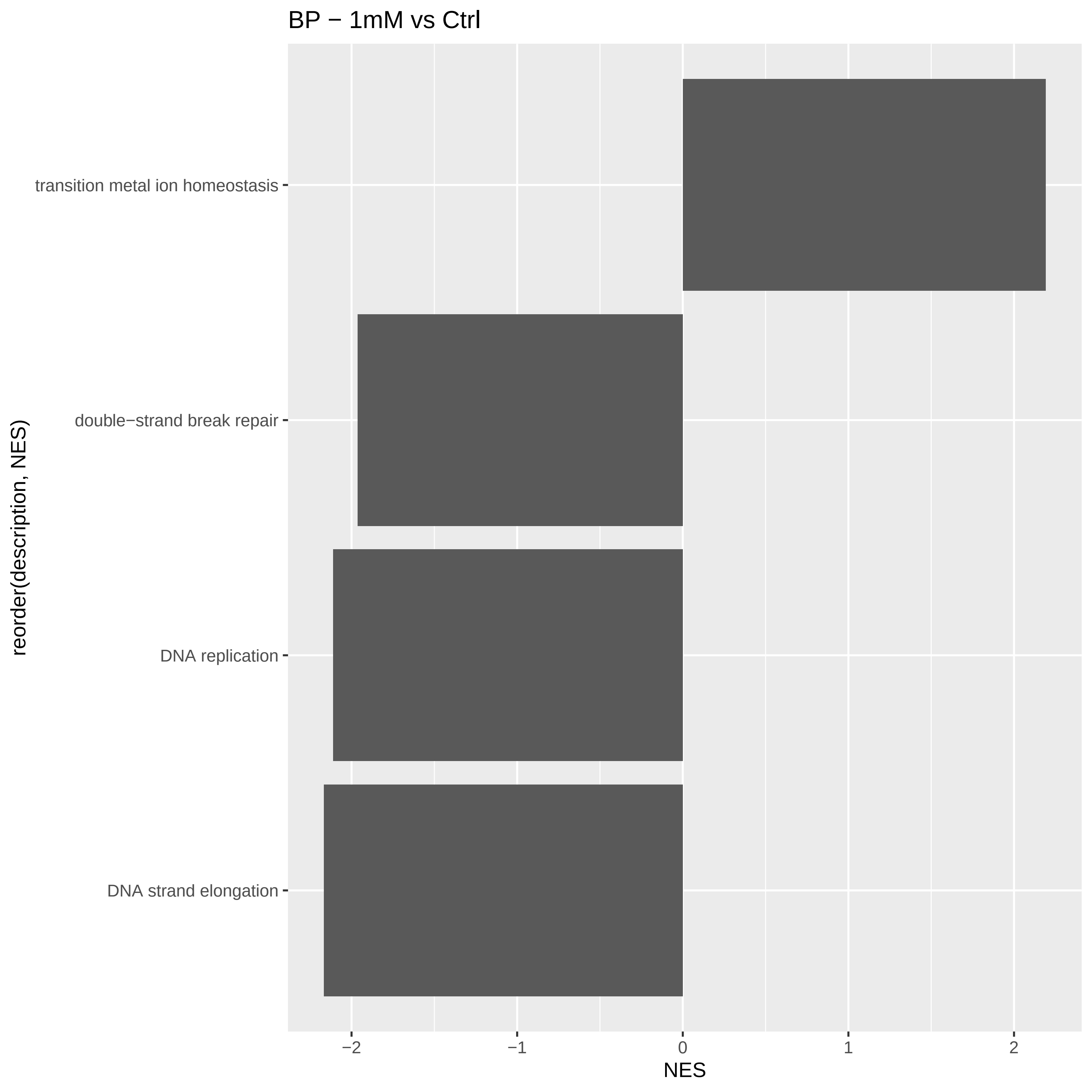

BP − 1mM vs Ctrl
transition metal ion homeostasis
double−strand break repair
DNA replication
DNA strand elongation
reorder(description, NES)
−2
−1
0
1
2
NES
