# Supplementary material for: Mapping protein carboxymethylation sites provides insights into their role in proteostasis and cell proliferation
Source: Nat Commun. 2021 Nov 18;12:6743. doi: 10.1038/s41467-021-26982-6 (PMC8602705; doi:10.1038/s41467-021-26982-6)
Supplement: Supplementary file 11 — Source Data [file 41467_2021_26982_MOESM11_ESM.zip › Figure 4/Fig 4A_B.pdf]

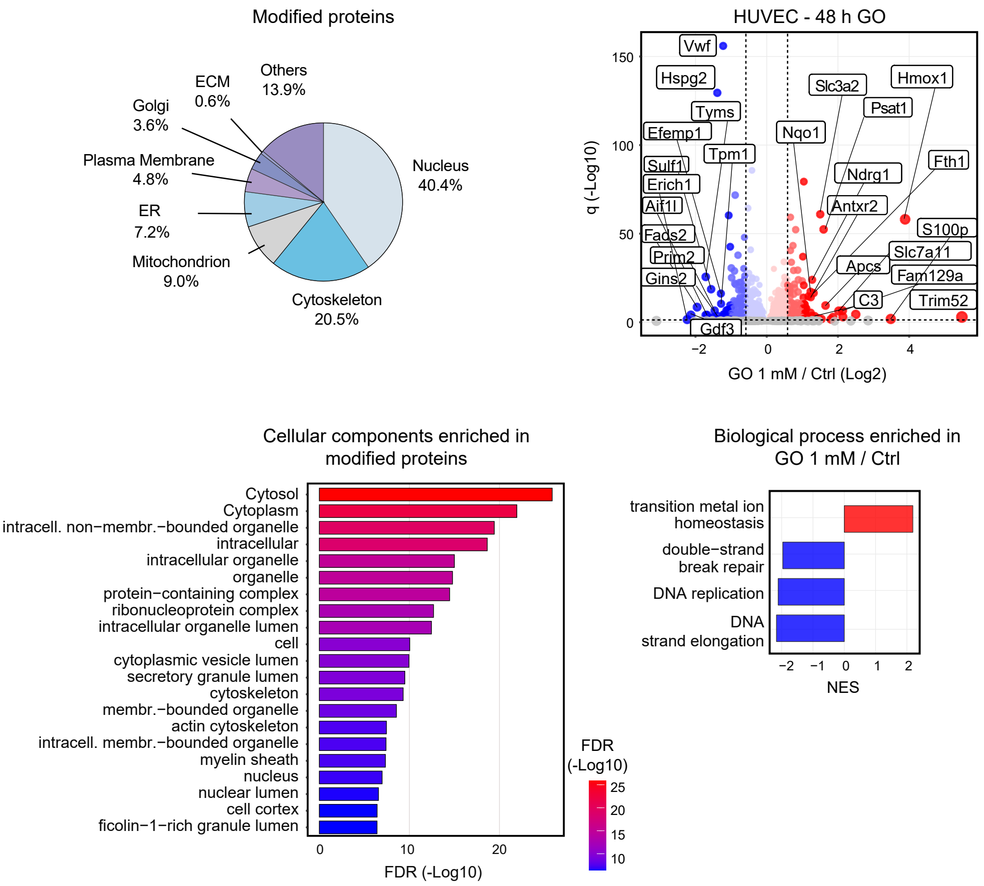
Modified proteins
Others 13.9%
ECM 0.6%
Golgi 3.6%
Plasma Membrane 4.8%
ER 7.2%
Mitochondrion 9.0%
Cytoskeleton 20.5%
Nucleus 40.4%
HUVEC - 48 h GO
q (-Log10)
GO 1 mM / Ctrl (Log2)
150
100
50
0
−2
0
2
4
Vwf
Hspg2
Tyms
Efemp1
Tpm1
Sulf1
Erich1
Aif1l
Fads2
Prim2
Gins2
Gdf3
Nqo1
Slc3a2
Psat1
Hmox1
Ndrg1
Fth1
Antxr2
S100p
Slc7a11
Apcs
Fam129a
C3
Trim52
Cellular components enriched in modified proteins
Cytosol
Cytoplasm
intracell. non−membr.−bounded organelle
intracellular
intracellular organelle
organelle
protein−containing complex
ribonucleoprotein complex
intracellular organelle lumen
cell
cytoplasmic vesicle lumen
secretory granule lumen
cytoskeleton
membr.−bounded organelle
actin cytoskeleton
intracell. membr.−bounded organelle
myelin sheath
nucleus
nuclear lumen
cell cortex
ficolin−1−rich granule lumen
0
10
20
FDR (-Log10)
FDR (-Log10)
25
20
15
10
Biological process enriched in GO 1 mM / Ctrl
transition metal ion homeostasis
double−strand break repair
DNA replication
DNA strand elongation
−2
−1
0
1
2
NES
