# Supplementary material for: Mapping protein carboxymethylation sites provides insights into their role in proteostasis and cell proliferation
Source: Nat Commun. 2021 Nov 18;12:6743. doi: 10.1038/s41467-021-26982-6 (PMC8602705; doi:10.1038/s41467-021-26982-6)
Supplement: Supplementary file 11 — Source Data [file 41467_2021_26982_MOESM11_ESM.zip › Figure 5/5C/CellCycle_Spectromine.pdf]

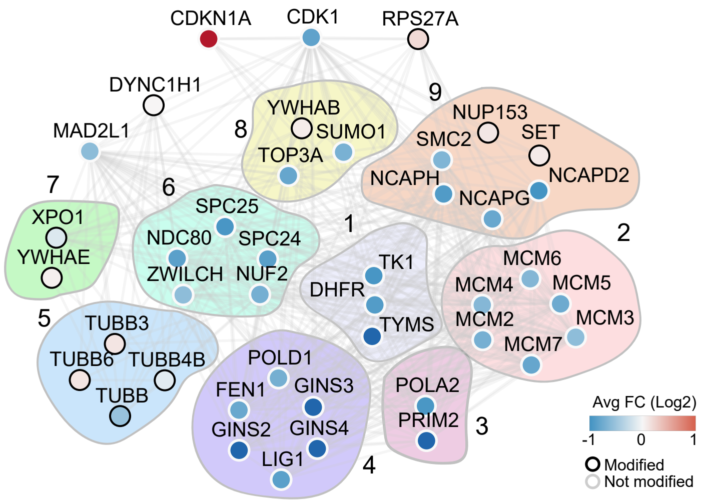

1. G1 G1/S Phase
2. G1 G1/S Phase, G2/M Checkpoints, S Phase
3. G1 G1/S Phase, S Phase
4. S Phase
5. G2 G2/M Phase, M Phase
6. M Phase, Spindle Checkpoints
7. G2 G2/M Phase, G2/M Checkpoints, M Phase
8. G2/M Checkpoints
9. M Phase
