# Supplementary material for: Mapping protein carboxymethylation sites provides insights into their role in proteostasis and cell proliferation
Source: Nat Commun. 2021 Nov 18;12:6743. doi: 10.1038/s41467-021-26982-6 (PMC8602705; doi:10.1038/s41467-021-26982-6)
Supplement: Supplementary file 11 — Source Data [file 41467_2021_26982_MOESM11_ESM.zip › FigureS1/FigureS1.pdf]

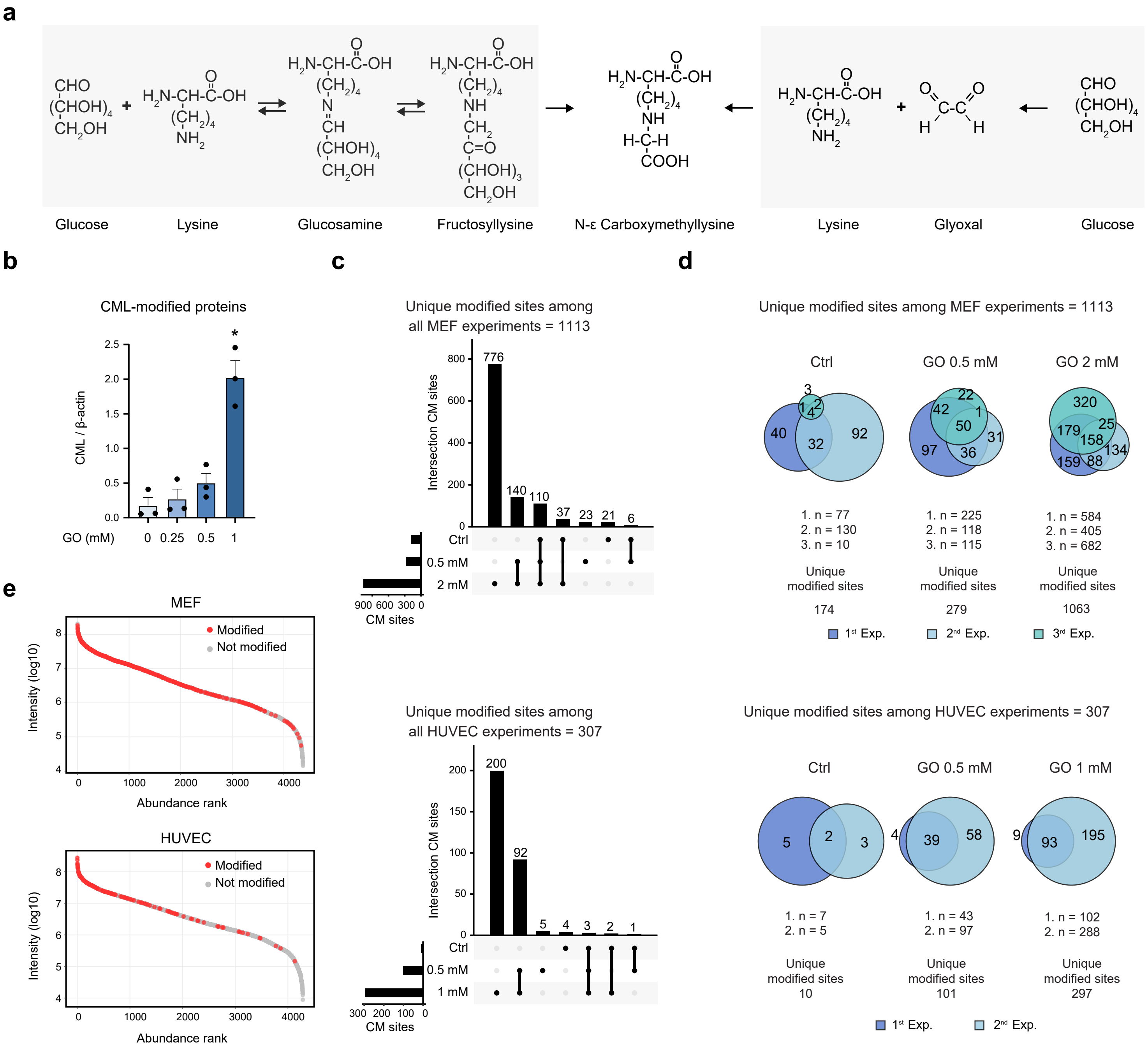
a
Glucose
Lysine
Glucosamine
Fructosyllysine
N-ε Carboxymethyllysine
Lysine
Glyoxal
Glucose
b
CML-modified proteins
CML / β-actin
GO (mM)
0 0.25 0.5 1
c
Unique modified sites among all MEF experiments = 1113
Intersection CM sites
776 140 110 37 23 21 6
Ctrl
0.5 mM
2 mM
CM sites
d
Unique modified sites among MEF experiments = 1113
Ctrl
GO 0.5 mM
GO 2 mM
1. n = 77
2. n = 130
3. n = 10
Unique modified sites
174
1. n = 225
2. n = 118
3. n = 115
Unique modified sites
279
1. n = 584
2. n = 405
3. n = 682
Unique modified sites
1063
1st Exp.
2nd Exp.
3rd Exp.
e
MEF
Modified
Not modified
Intensity (log10)
Abundance rank
HUVEC
Modified
Not modified
Intensity (log10)
Abundance rank
Unique modified sites among all HUVEC experiments = 307
Intersection CM sites
200 92 5 4 3 2 1
Ctrl
0.5 mM
1 mM
CM sites
Unique modified sites among HUVEC experiments = 307
Ctrl
GO 0.5 mM
GO 1 mM
1. n = 7
2. n = 5
Unique modified sites
10
1. n = 43
2. n = 97
Unique modified sites
101
1. n = 102
2. n = 288
Unique modified sites
297
1st Exp.
2nd Exp.
