# Supplementary figures and images for: Mapping protein carboxymethylation sites provides insights into their role in proteostasis and cell proliferation (part 2 of 2)
Source: Nat Commun. 2021 Nov 18;12:6743. doi: 10.1038/s41467-021-26982-6 (PMC8602705; doi:10.1038/s41467-021-26982-6)

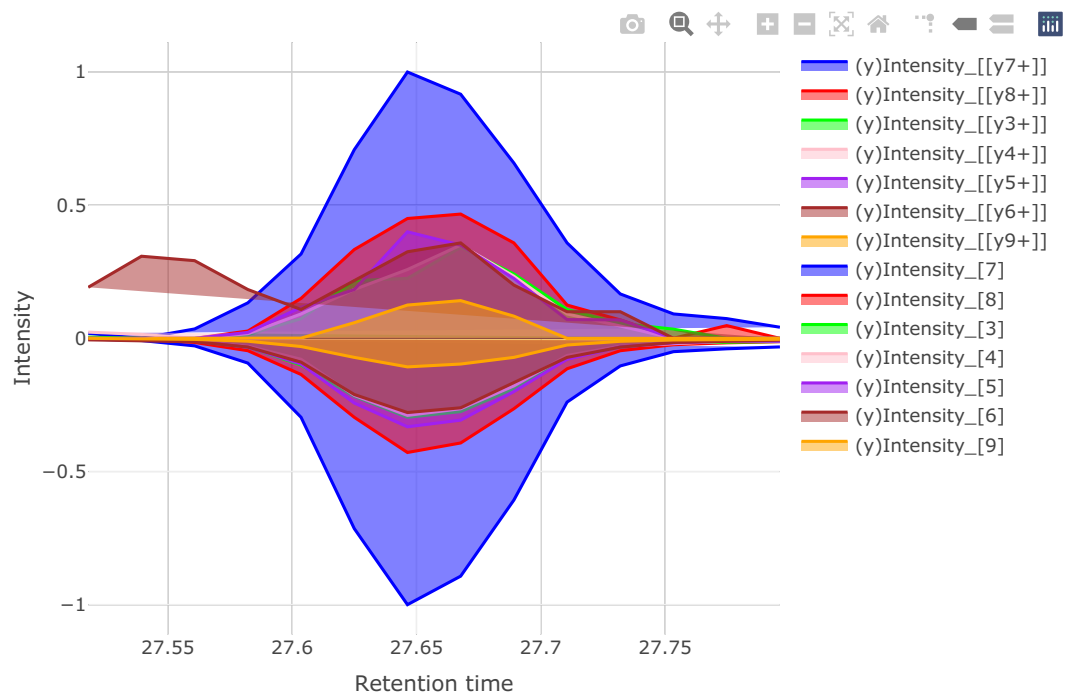

Supplement: Supplementary file 11 — Source Data [file 41467_2021_26982_MOESM11_ESM.zip › FigureS2/2A/ATPG_MOUSE._THSDQFLVSFK[CML]DVGR_.3.pdf]

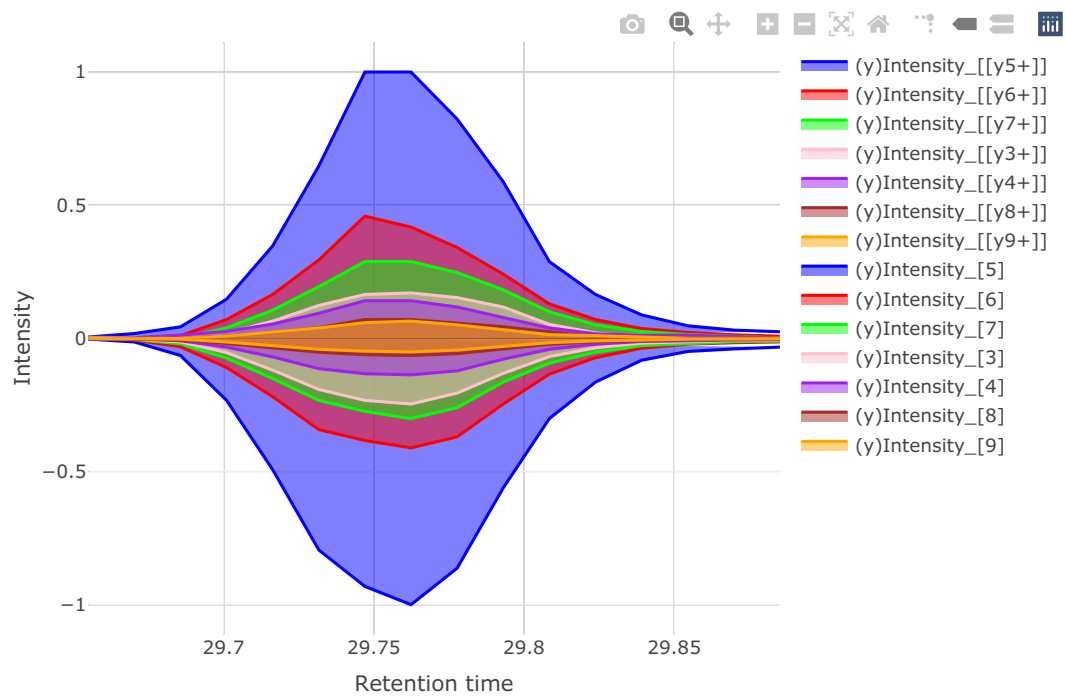

Supplement: Supplementary file 11 — Source Data [file 41467_2021_26982_MOESM11_ESM.zip › FigureS2/2A/ATPO_MOUSE._VSLAVLNPYIK[CML]R_.3.pdf]

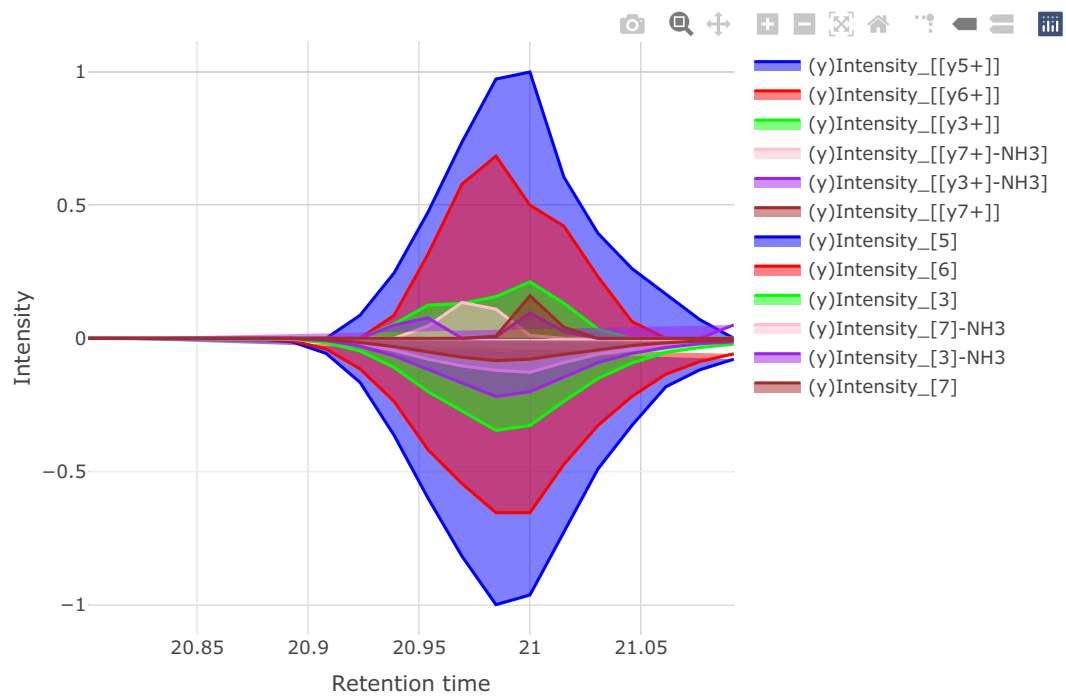

Supplement: Supplementary file 11 — Source Data [file 41467_2021_26982_MOESM11_ESM.zip › FigureS2/2A/CATA_MOUSE._DAQLFIQK[CML]K_.2.pdf]

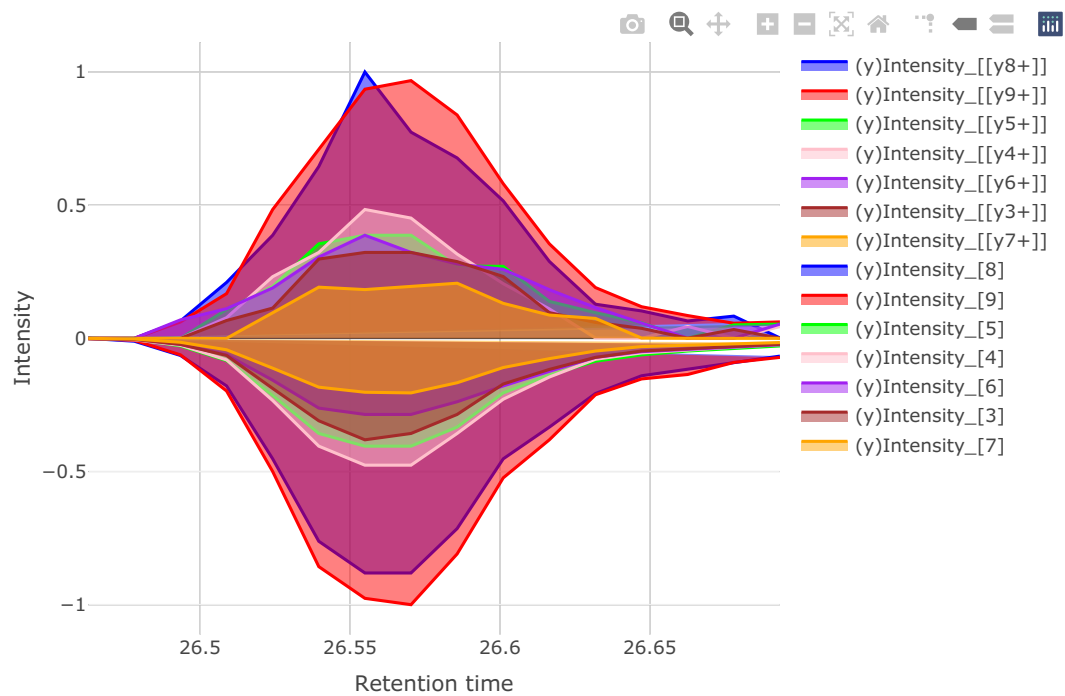

Supplement: Supplementary file 11 — Source Data [file 41467_2021_26982_MOESM11_ESM.zip › FigureS2/2A/ECHA_MOUSE._FVDLYGAQK[CML]VVDR_.3.pdf]

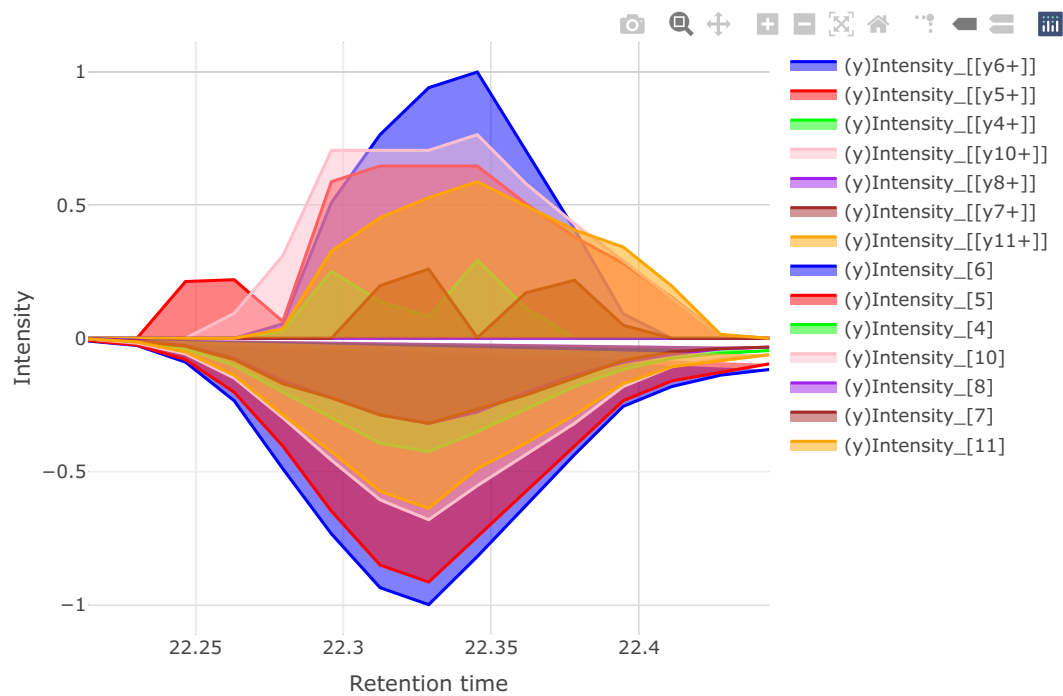

Supplement: Supplementary file 11 — Source Data [file 41467_2021_26982_MOESM11_ESM.zip › FigureS2/2A/ECHM_MOUSE._AFAAGADIK[CML]EMQNR_.3.pdf]

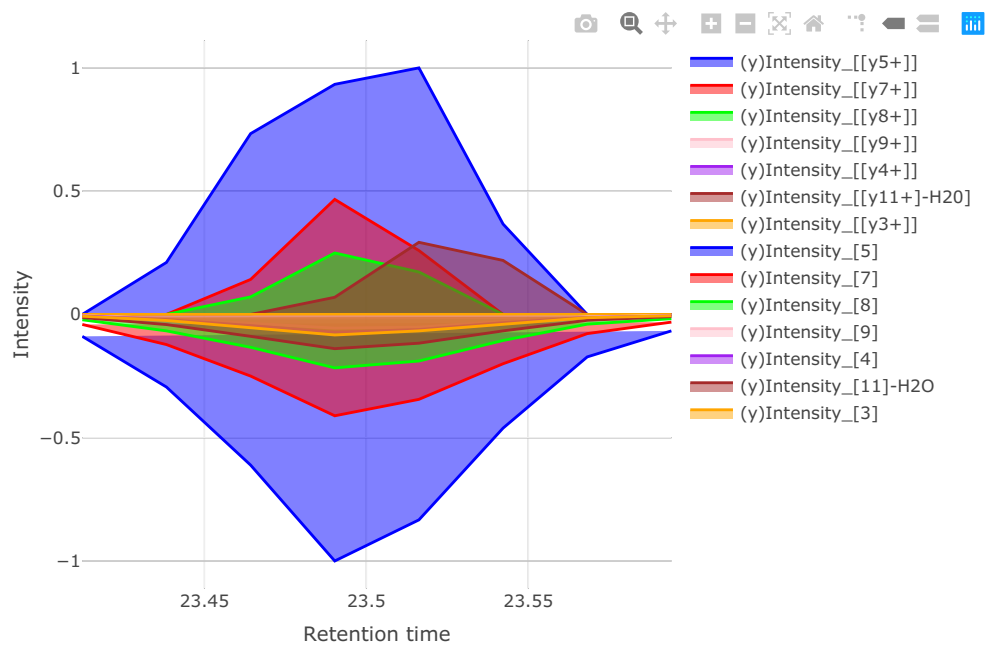

Supplement: Supplementary file 11 — Source Data [file 41467_2021_26982_MOESM11_ESM.zip › FigureS2/2A/ECHP_MOUSE._FAQTVIGK[CML]PIEPR_.3.pdf]

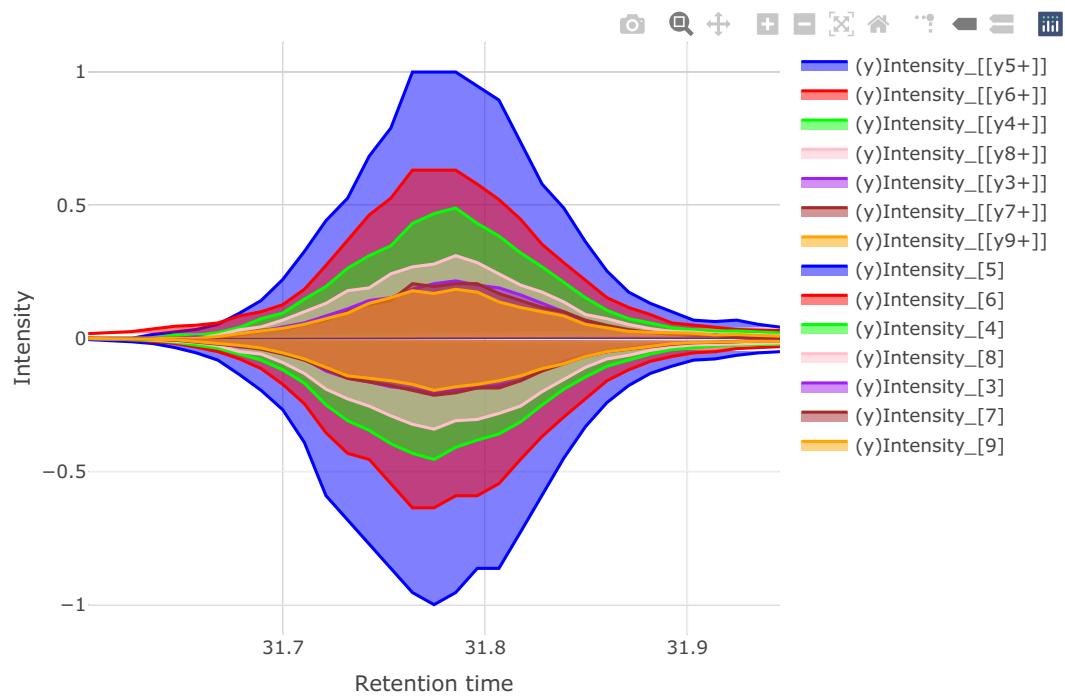

Supplement: Supplementary file 11 — Source Data [file 41467_2021_26982_MOESM11_ESM.zip › FigureS2/2A/H4_MOUSE._TVTAMDVVYALK[CML]R_.3.pdf]

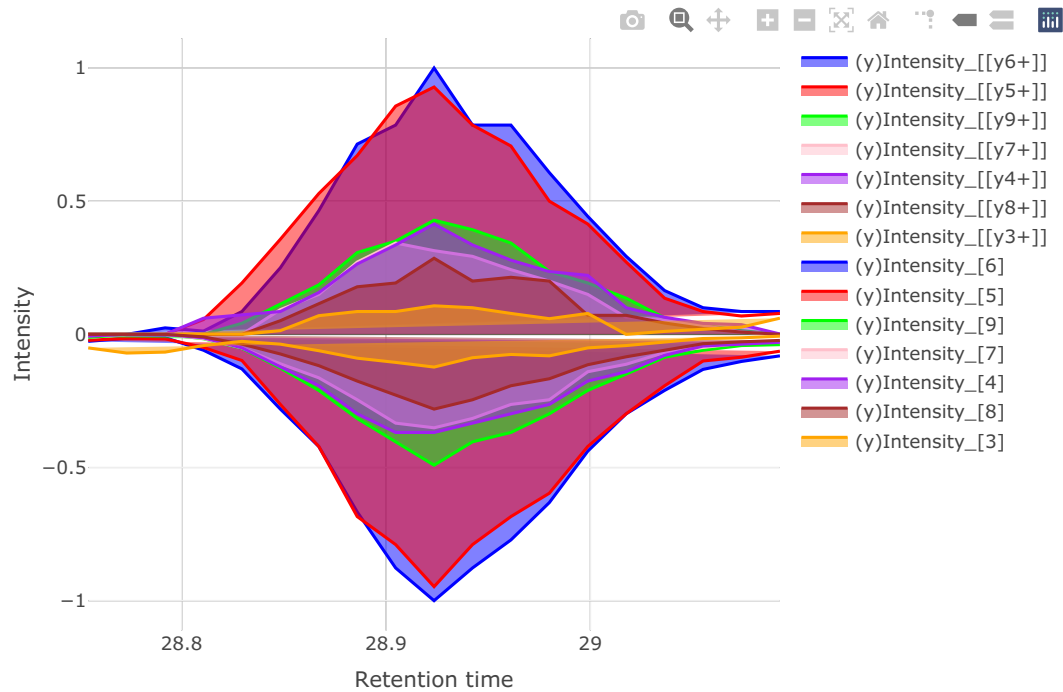

Supplement: Supplementary file 11 — Source Data [file 41467_2021_26982_MOESM11_ESM.zip › FigureS2/2A/HBA_MOUSE._FLASVSTVLTSK[CML]YR_.3.pdf]
