# Supplementary figures and images for: Mapping protein carboxymethylation sites provides insights into their role in proteostasis and cell proliferation (part 1 of 2)
Source: Nat Commun. 2021 Nov 18;12:6743. doi: 10.1038/s41467-021-26982-6 (PMC8602705; doi:10.1038/s41467-021-26982-6)

# 210621\_Intracell\_GO\_correct

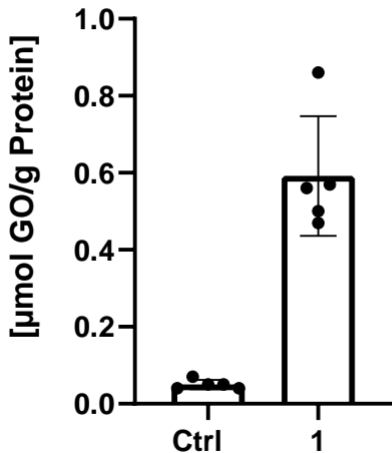

Supplement: Supplementary file 11 — Source Data [file 41467_2021_26982_MOESM11_ESM.zip › Figure 1/1B/210621_Intracell_GO.pdf]

# CML\_WesternBlot\_Huvec (48h GO)

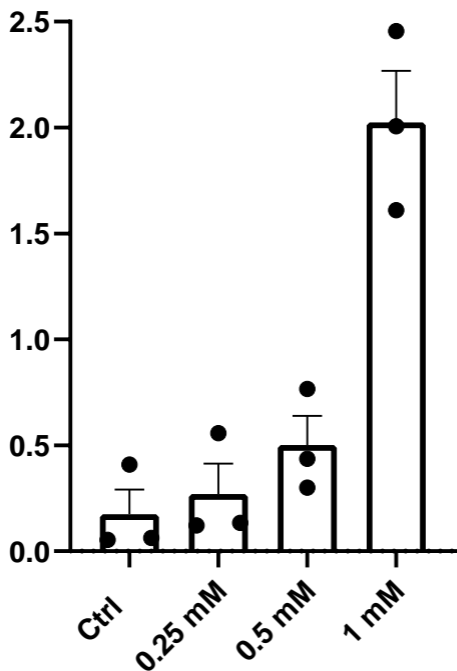

Supplement: Supplementary file 11 — Source Data [file 41467_2021_26982_MOESM11_ESM.zip › Figure 1/1C/CML_WesternBlot_Huvec (48h GO).pdf]

## MEF\_24h\_GO\_6repl

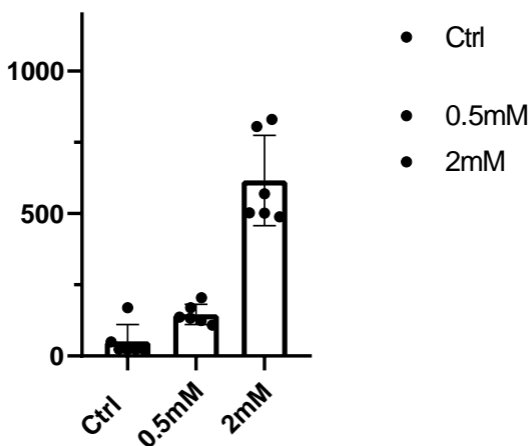

## 200827\_HUVEC

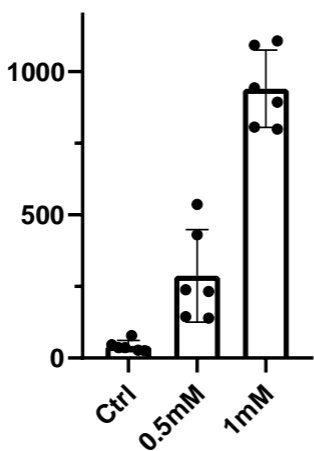

Supplement: Supplementary file 11 — Source Data [file 41467_2021_26982_MOESM11_ESM.zip › Figure 1/1D/Figure1_D.pdf]

# HUVEC\_%Using SM search together 2Exps

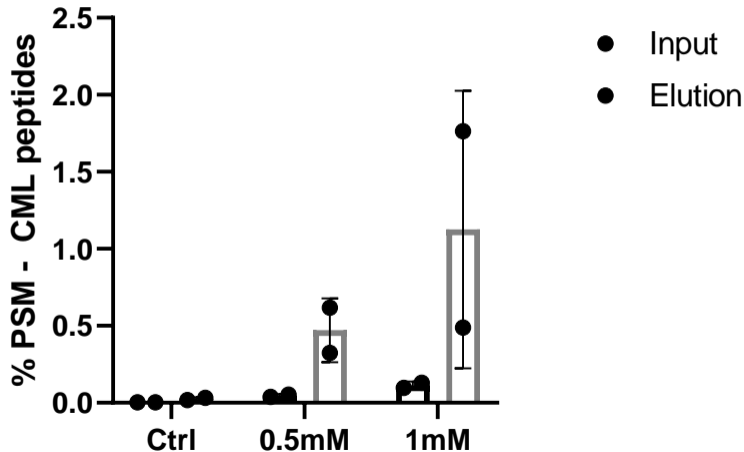

Supplement: Supplementary file 11 — Source Data [file 41467_2021_26982_MOESM11_ESM.zip › Figure 1/1E/HUVEC_%Using SM search together 2Exps.pdf]

# MEF\_%Using SM search together 3Exps

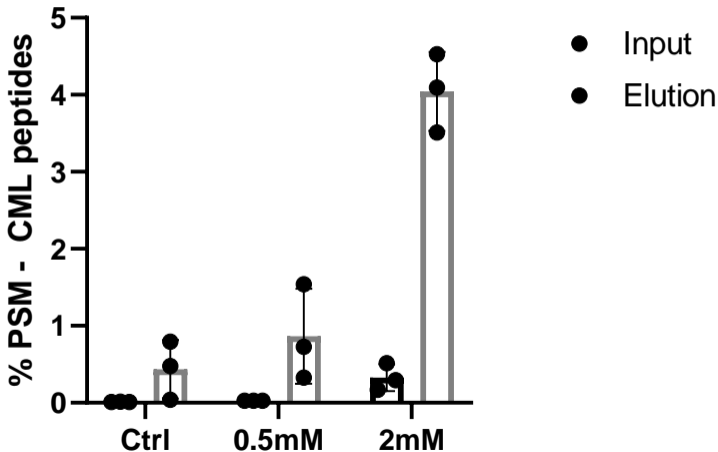

Supplement: Supplementary file 11 — Source Data [file 41467_2021_26982_MOESM11_ESM.zip › Figure 1/1E/MEF_%Using SM search together 3Exps.pdf]

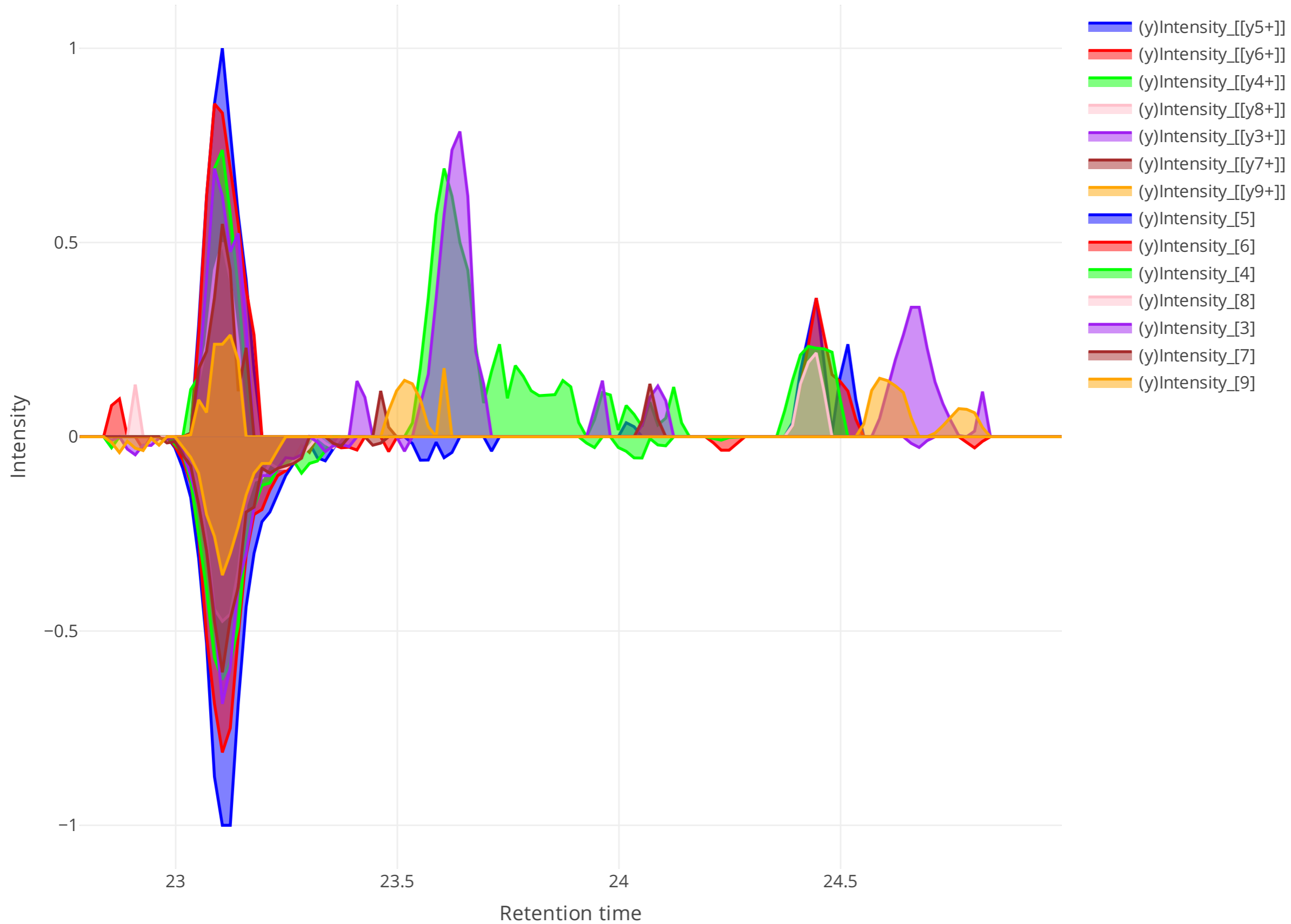

Supplement: Supplementary file 11 — Source Data [file 41467_2021_26982_MOESM11_ESM.zip › Figure 1/1F/Figure1F_left.pdf]

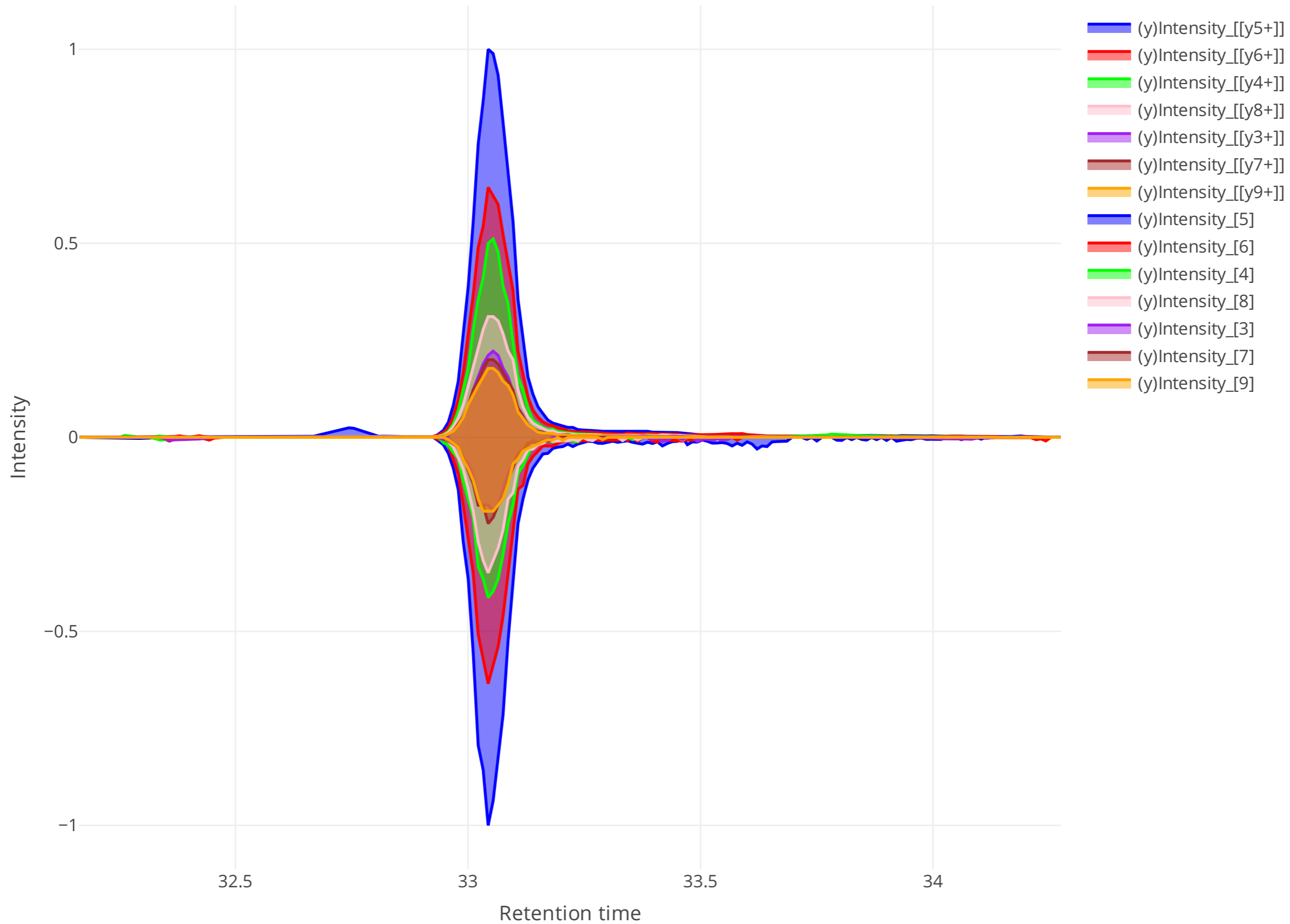

Supplement: Supplementary file 11 — Source Data [file 41467_2021_26982_MOESM11_ESM.zip › Figure 1/1F/Figure1F_right.pdf]

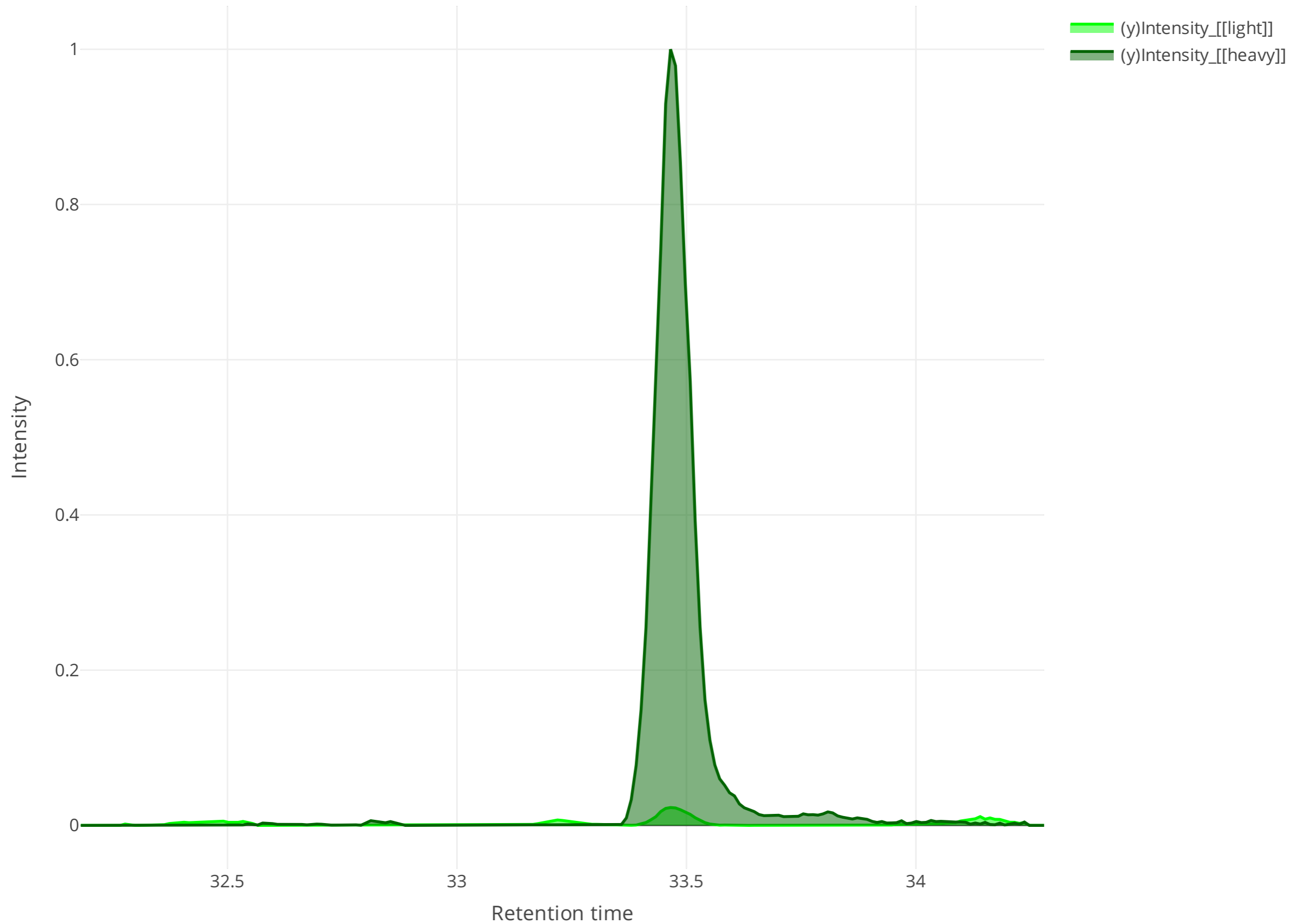

Supplement: Supplementary file 11 — Source Data [file 41467_2021_26982_MOESM11_ESM.zip › Figure 1/1G/Figure1G_left.pdf]

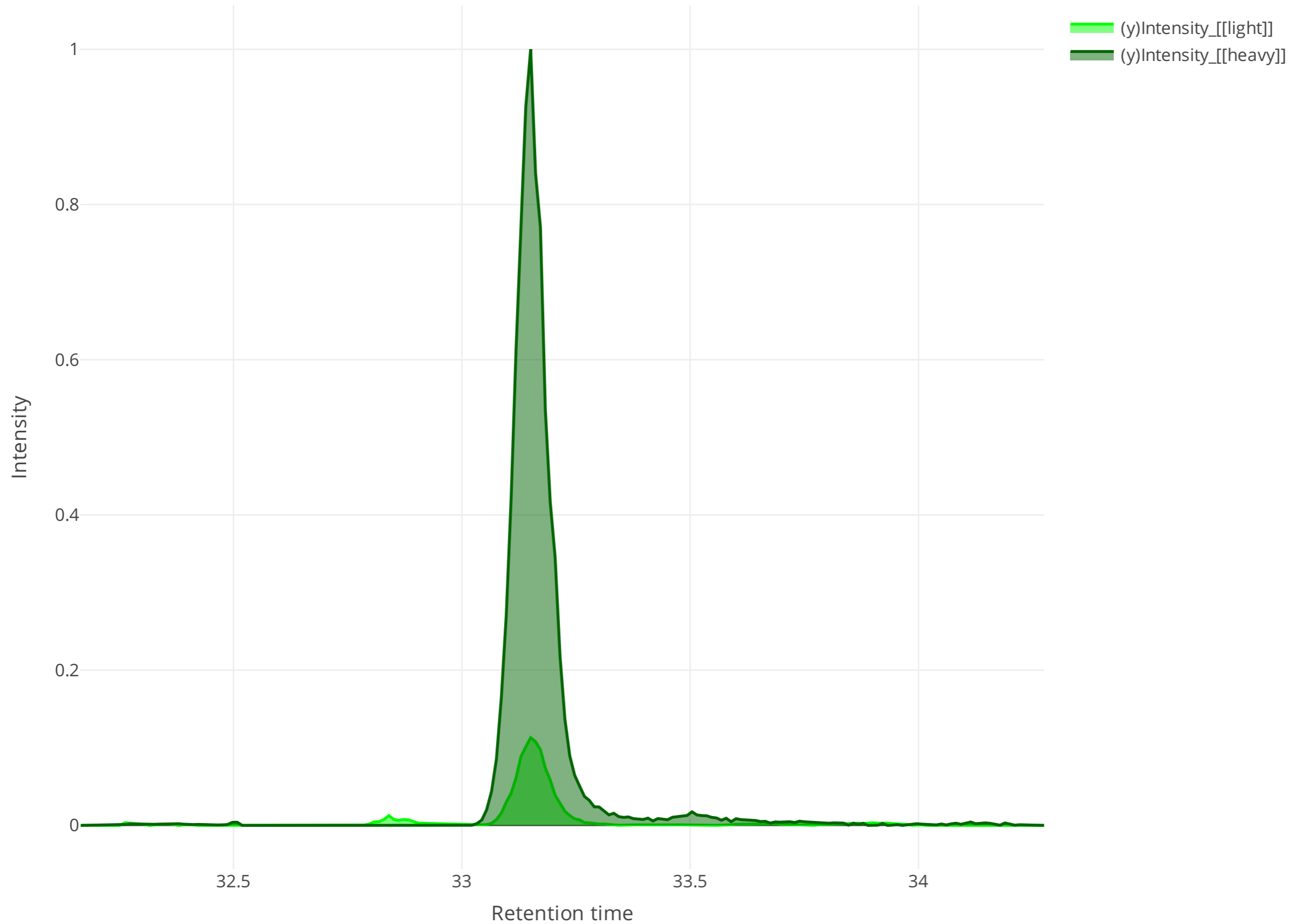

Supplement: Supplementary file 11 — Source Data [file 41467_2021_26982_MOESM11_ESM.zip › Figure 1/1G/Figure1G_middle.pdf]

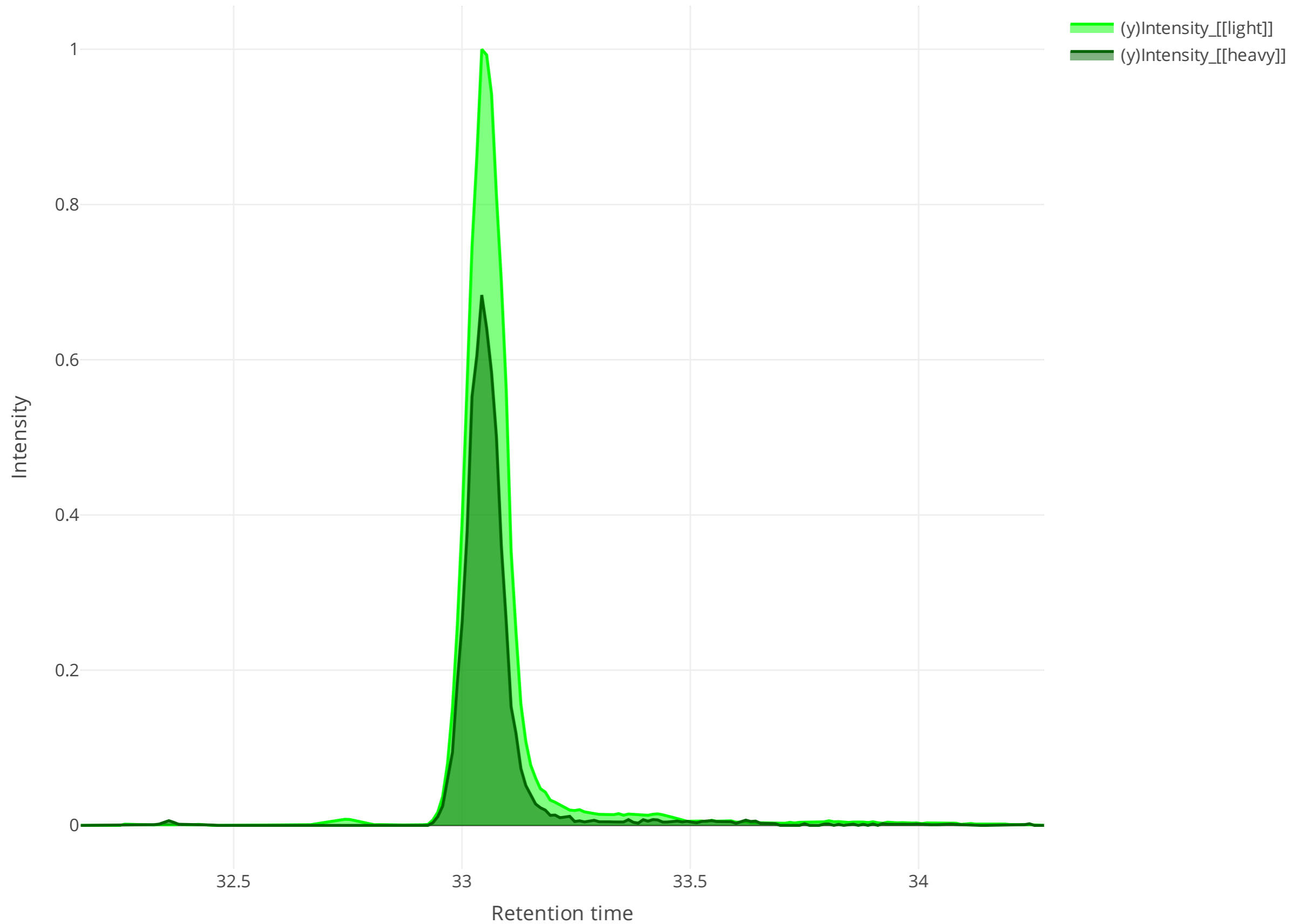

Supplement: Supplementary file 11 — Source Data [file 41467_2021_26982_MOESM11_ESM.zip › Figure 1/1G/Figure1G_right.pdf]

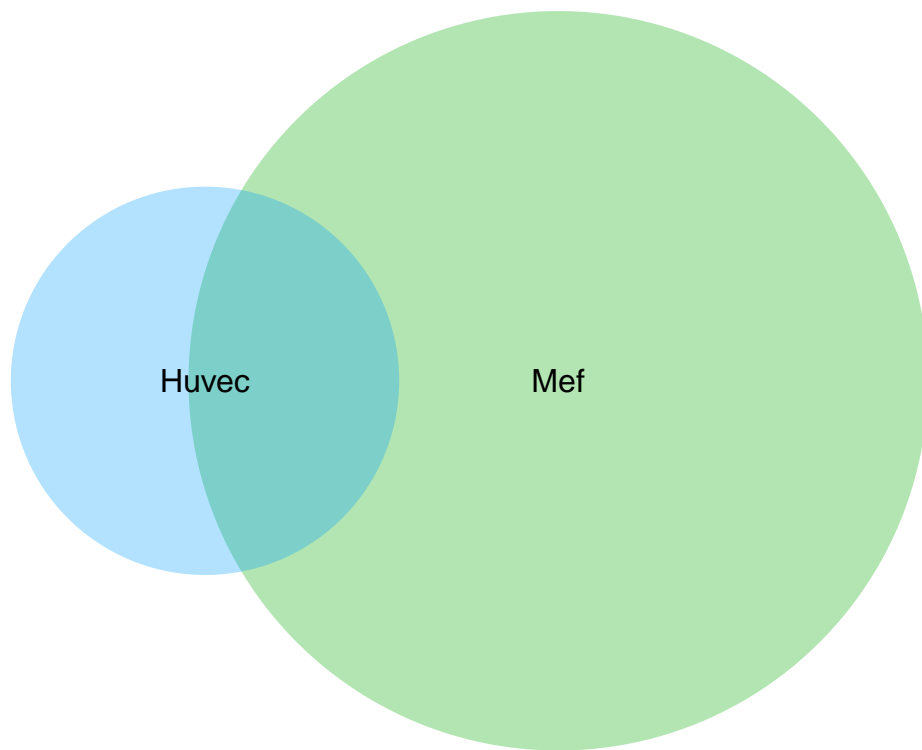

Supplement: Supplementary file 11 — Source Data [file 41467_2021_26982_MOESM11_ESM.zip › Figure 1/1H/210616_Overlap_Huvec_Mef.pdf]

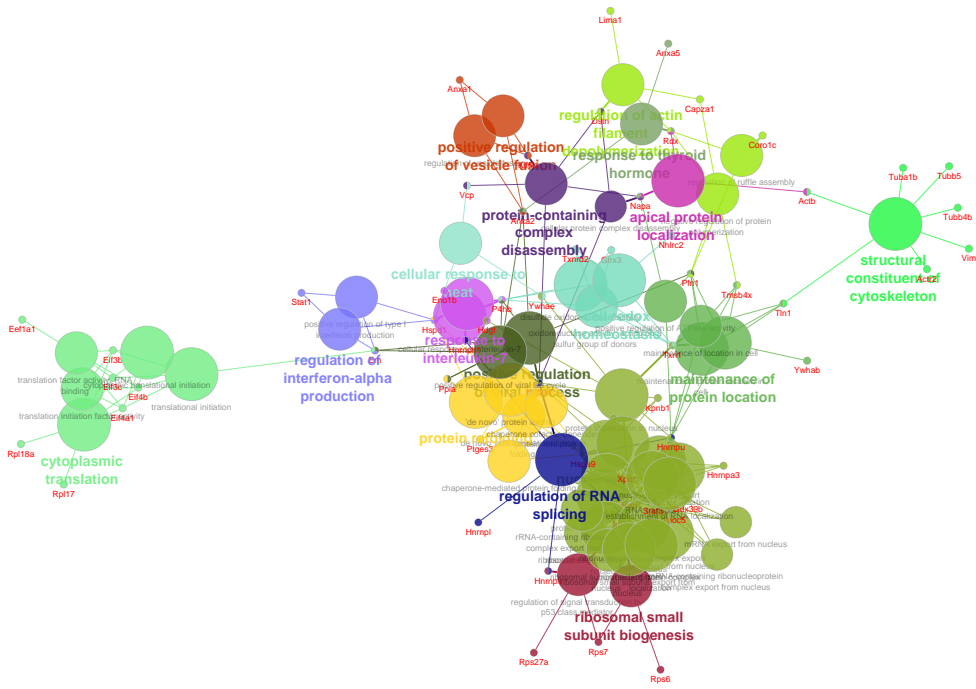

Supplement: Supplementary file 11 — Source Data [file 41467_2021_26982_MOESM11_ESM.zip › Figure 1/1I/210616_Overlap_Mef_Huvec_Spectromine.pdf]

# Hepato.cells turnover

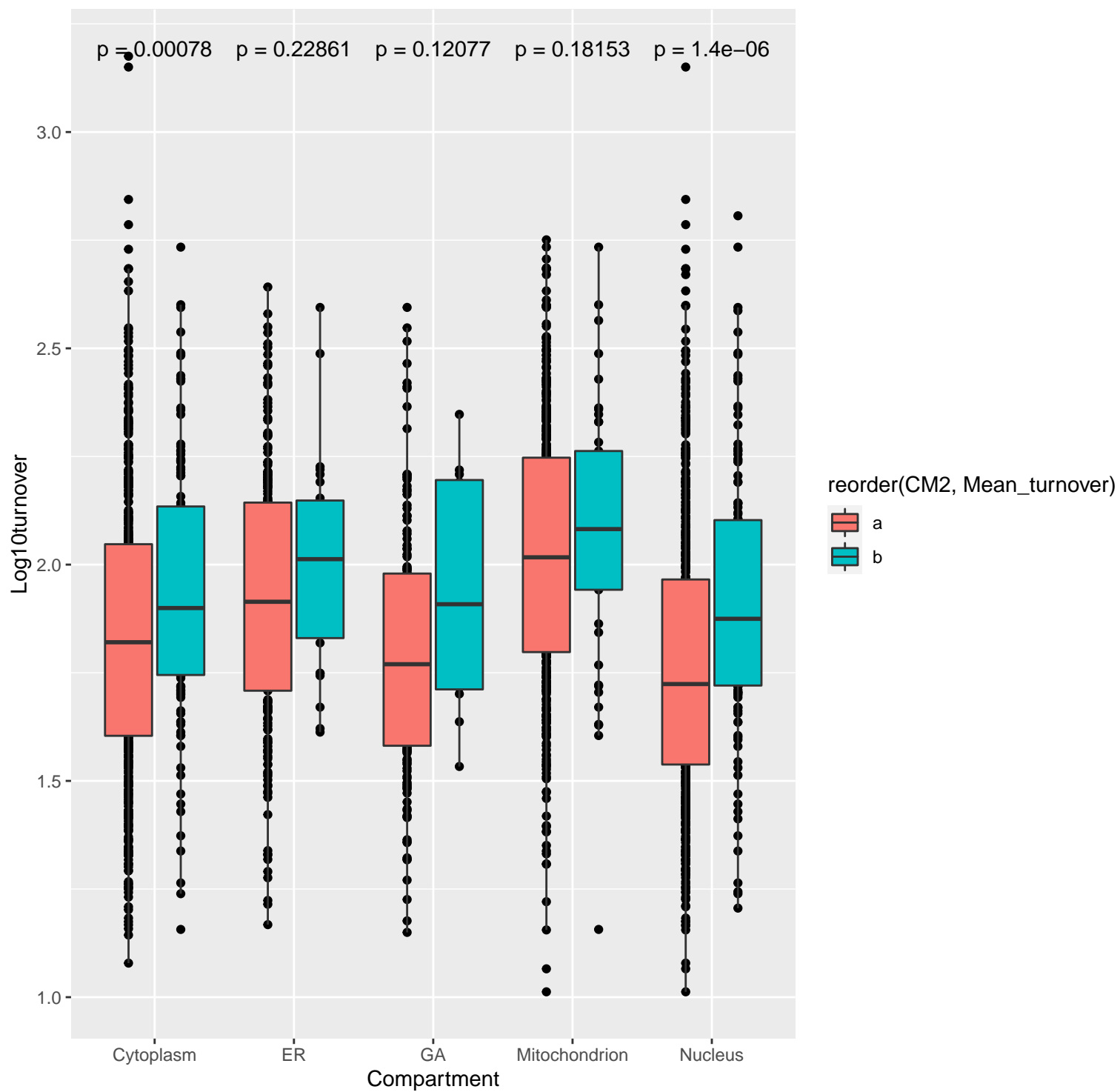

Supplement: Supplementary file 11 — Source Data [file 41467_2021_26982_MOESM11_ESM.zip › Figure 1/1J/201004_Boxplot_ProteinTurnover_Hepatocells.pdf]

# Hepatocytes turnover vs PG.Label.Free.Quant

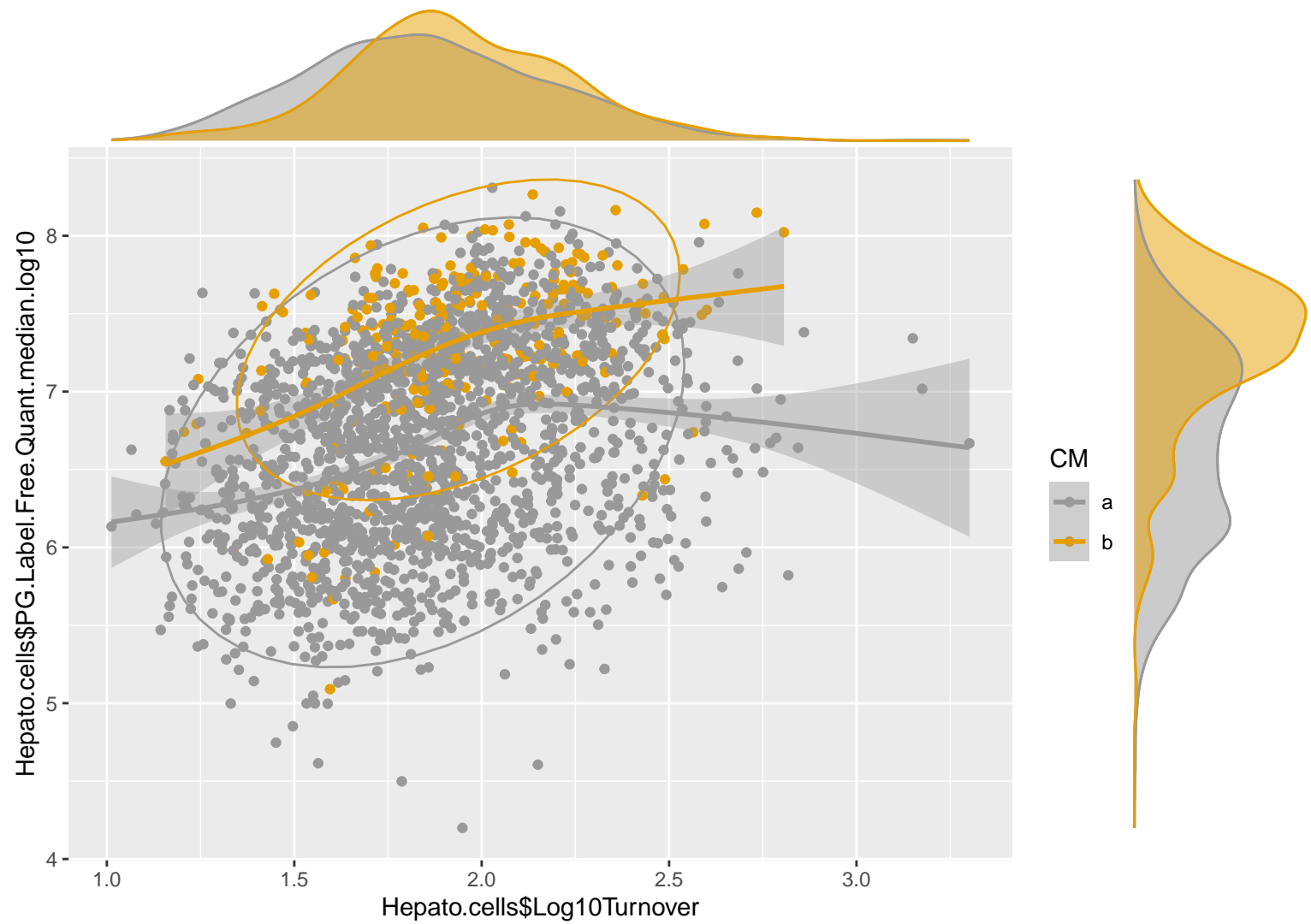

Supplement: Supplementary file 11 — Source Data [file 41467_2021_26982_MOESM11_ESM.zip › Figure 1/1J/210610_Scatterplot_turnover_PG.Label.Free.Quant.pdf]

# Hepato.cells turnover

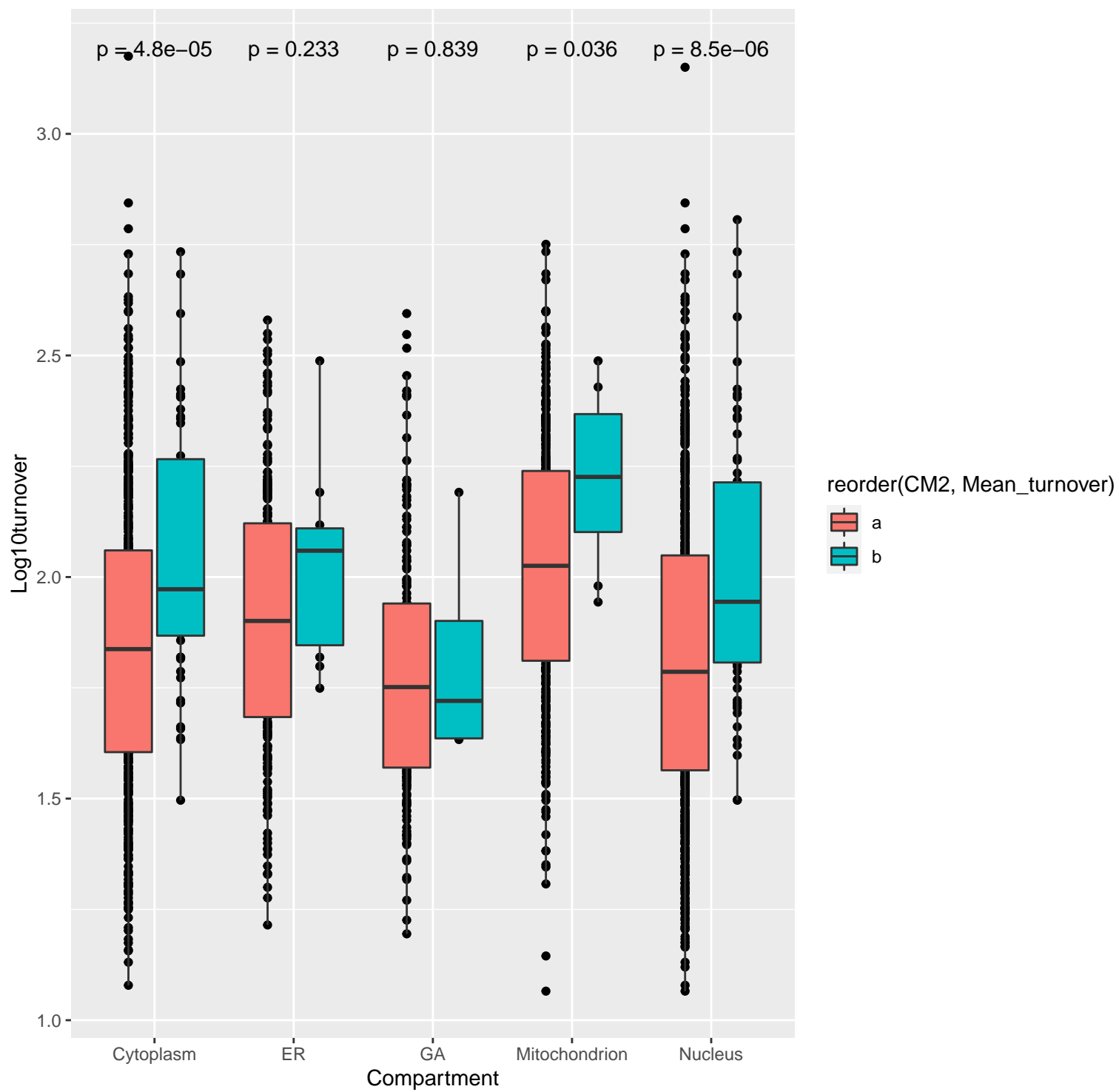

Supplement: Supplementary file 11 — Source Data [file 41467_2021_26982_MOESM11_ESM.zip › Figure 1/1K/201004_Boxplot_ProteinTurnover_Hepatocells.pdf]

# Hepatocytes turnover vs PG.Label.Free.Quant

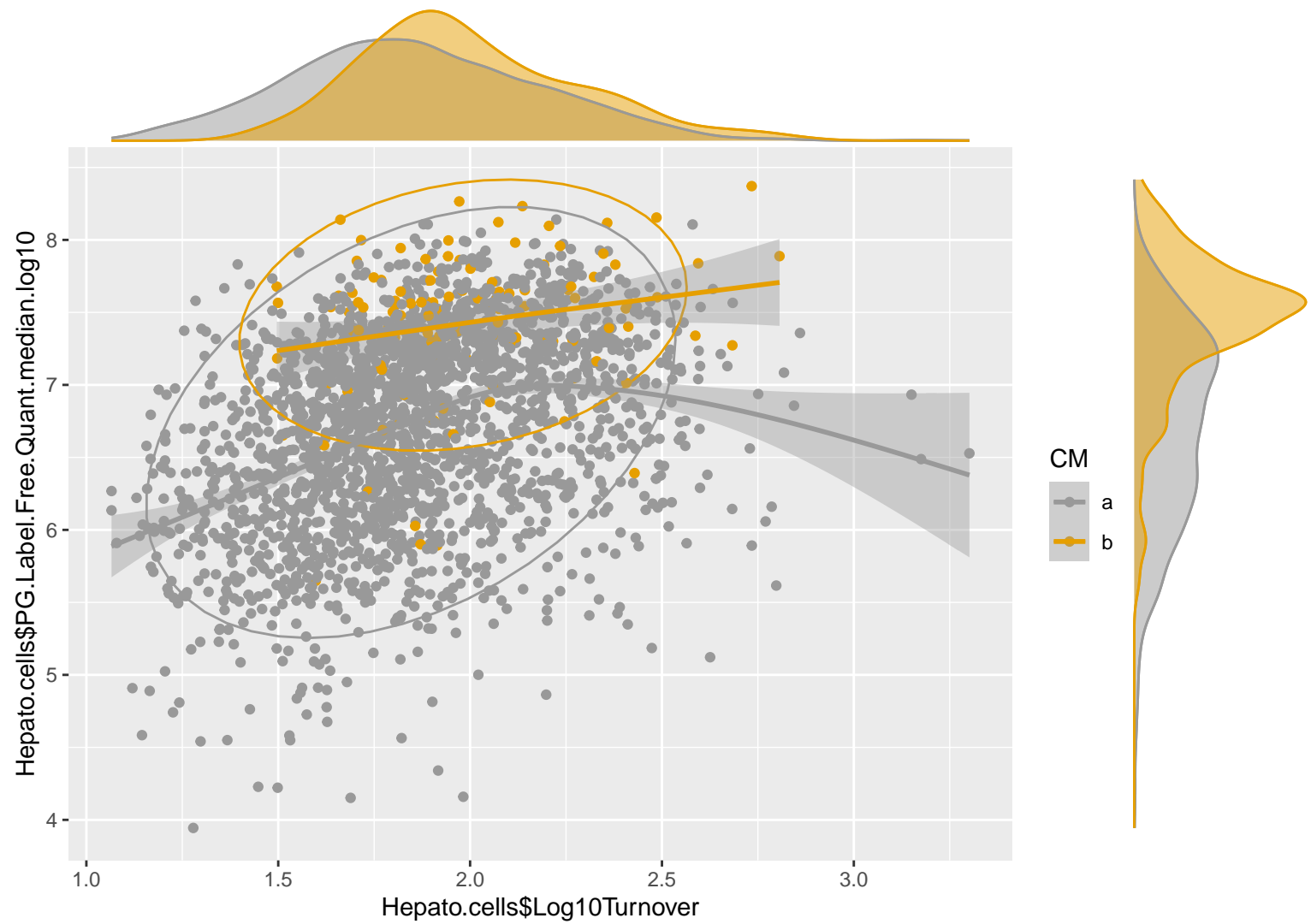

Supplement: Supplementary file 11 — Source Data [file 41467_2021_26982_MOESM11_ESM.zip › Figure 1/1K/210610_Scatterplot_turnover_PG.Label.Free.Quant_HUVEC_Cm.pdf]

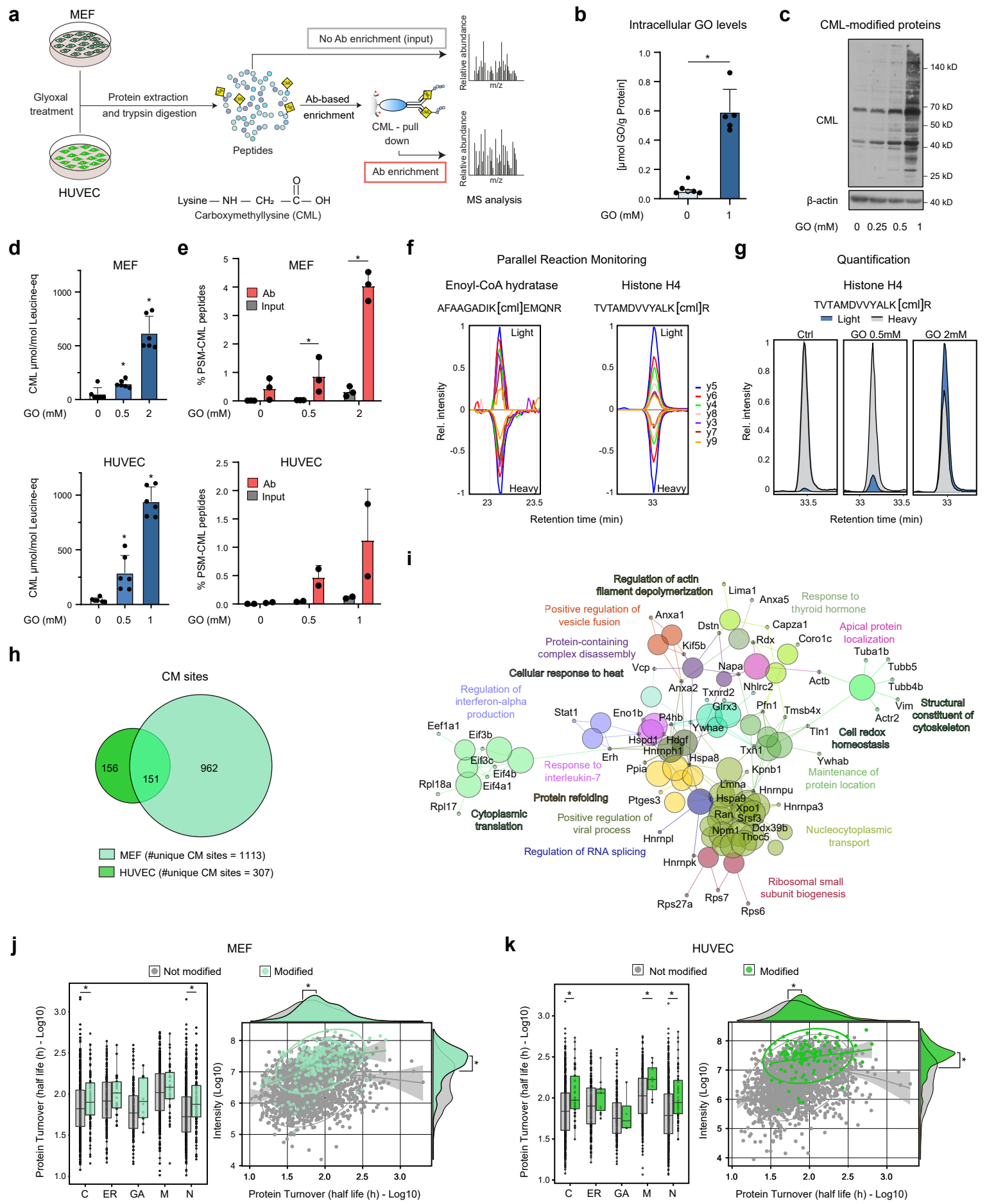

Supplement: Supplementary file 11 — Source Data [file 41467_2021_26982_MOESM11_ESM.zip › Figure 1/Figure1.pdf]

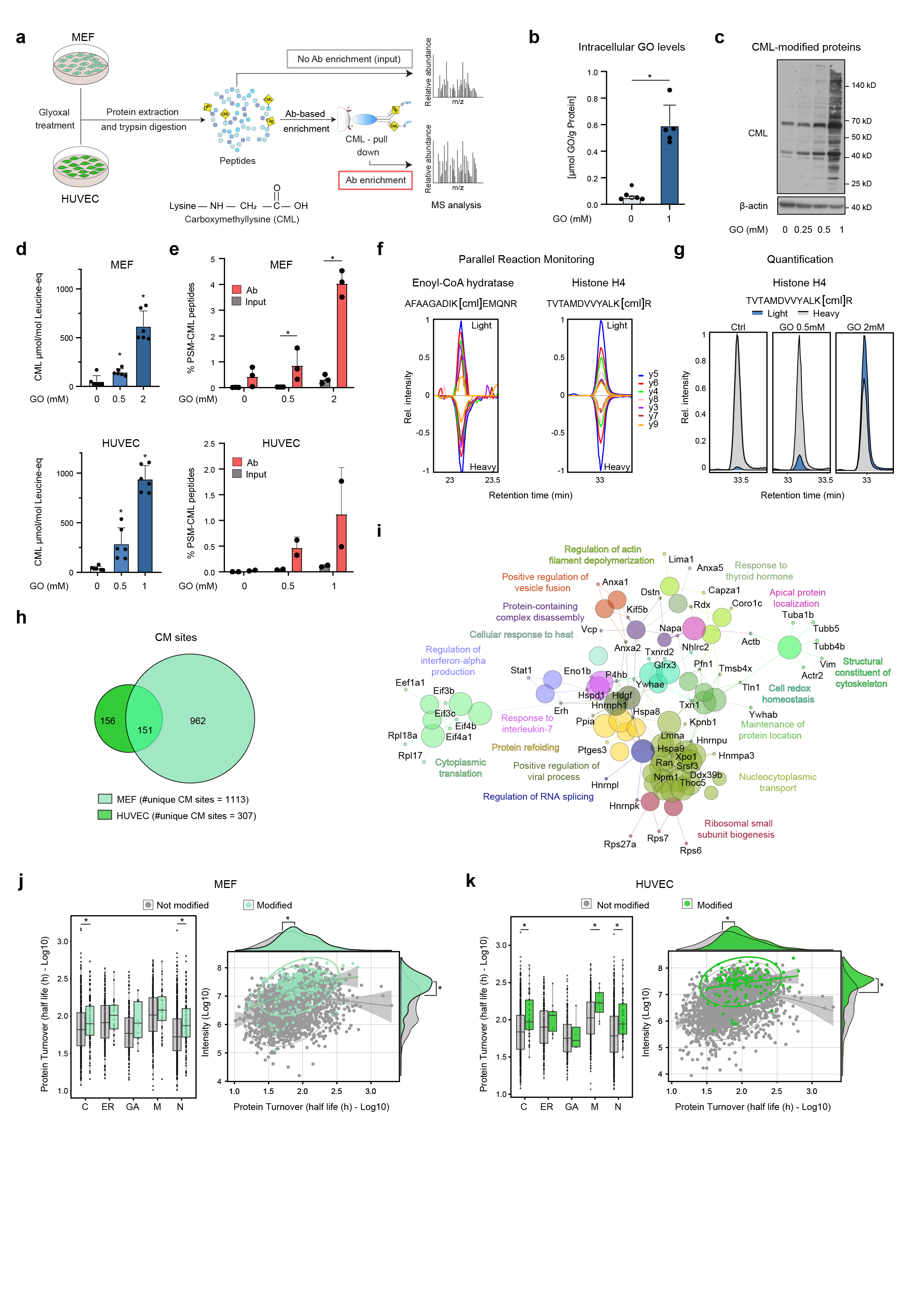

Supplement: Supplementary file 11 — Source Data [file 41467_2021_26982_MOESM11_ESM.zip › Figure 1/Figure1.png]

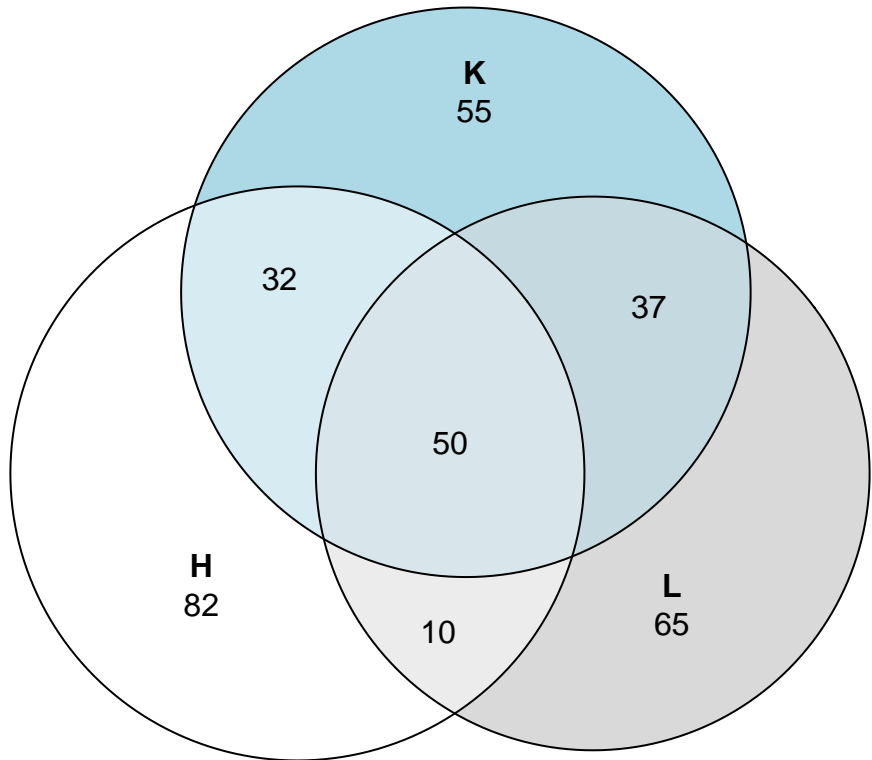

Supplement: Supplementary file 11 — Source Data [file 41467_2021_26982_MOESM11_ESM.zip › Figure 2/2B/VD_Heart.Liver.Kidney.ProteinAccessions.pdf]

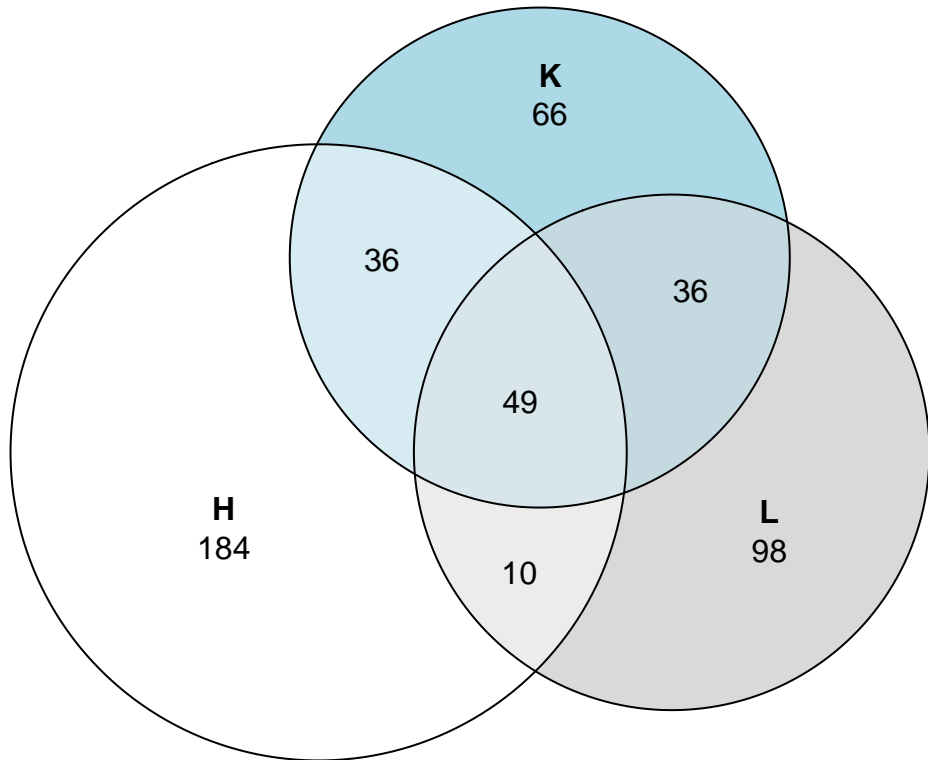

Supplement: Supplementary file 11 — Source Data [file 41467_2021_26982_MOESM11_ESM.zip › Figure 2/2B/VD_Heart.Liver.Kidney.Sites.pdf]

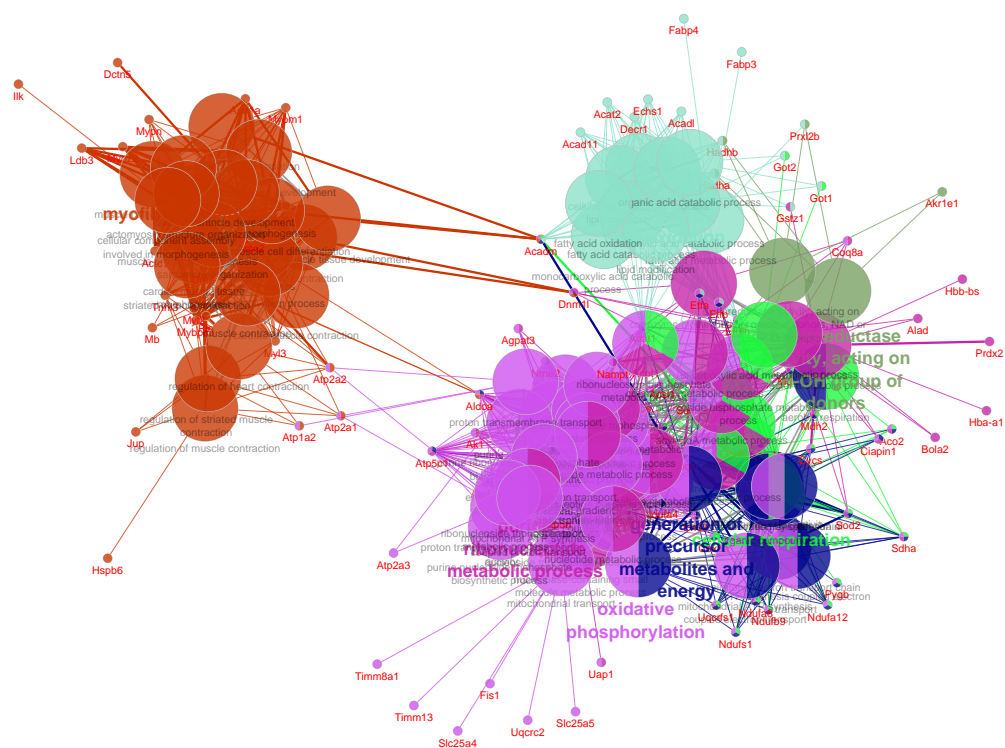

Supplement: Supplementary file 11 — Source Data [file 41467_2021_26982_MOESM11_ESM.zip › Figure 2/2C/Heart_8genes.pdf]

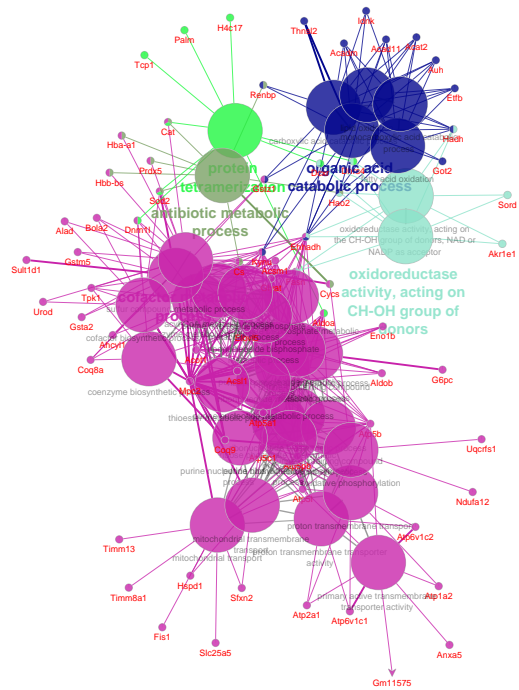

Supplement: Supplementary file 11 — Source Data [file 41467_2021_26982_MOESM11_ESM.zip › Figure 2/2C/Kidney_8genes.pdf]

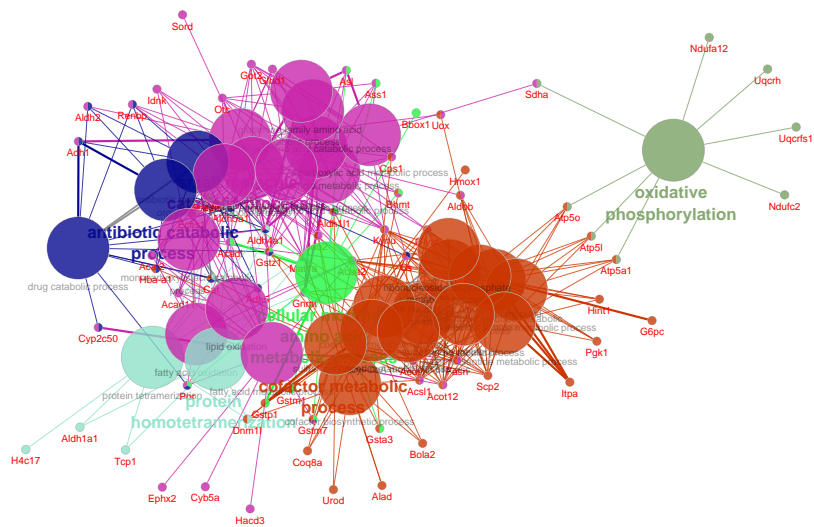

Supplement: Supplementary file 11 — Source Data [file 41467_2021_26982_MOESM11_ESM.zip › Figure 2/2C/Liver_8genes.pdf]

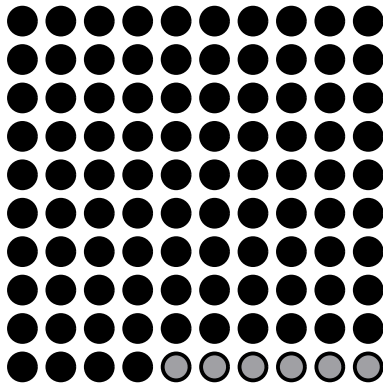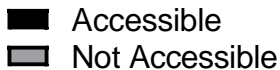

**Total=100**

Supplement: Supplementary file 11 — Source Data [file 41467_2021_26982_MOESM11_ESM.zip › Figure 2/2D/Accessibility Tissue [Perc. of sites over 0.pdf]

# Tissue

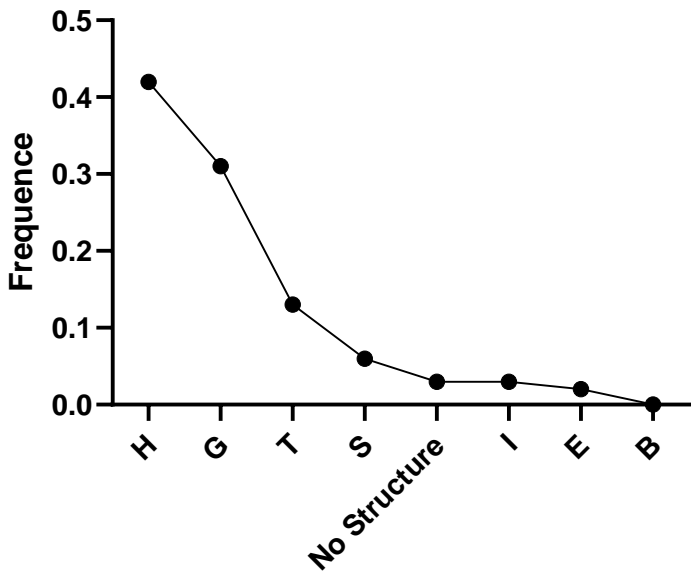

Supplement: Supplementary file 11 — Source Data [file 41467_2021_26982_MOESM11_ESM.zip › Figure 2/2D/Tissue.pdf]

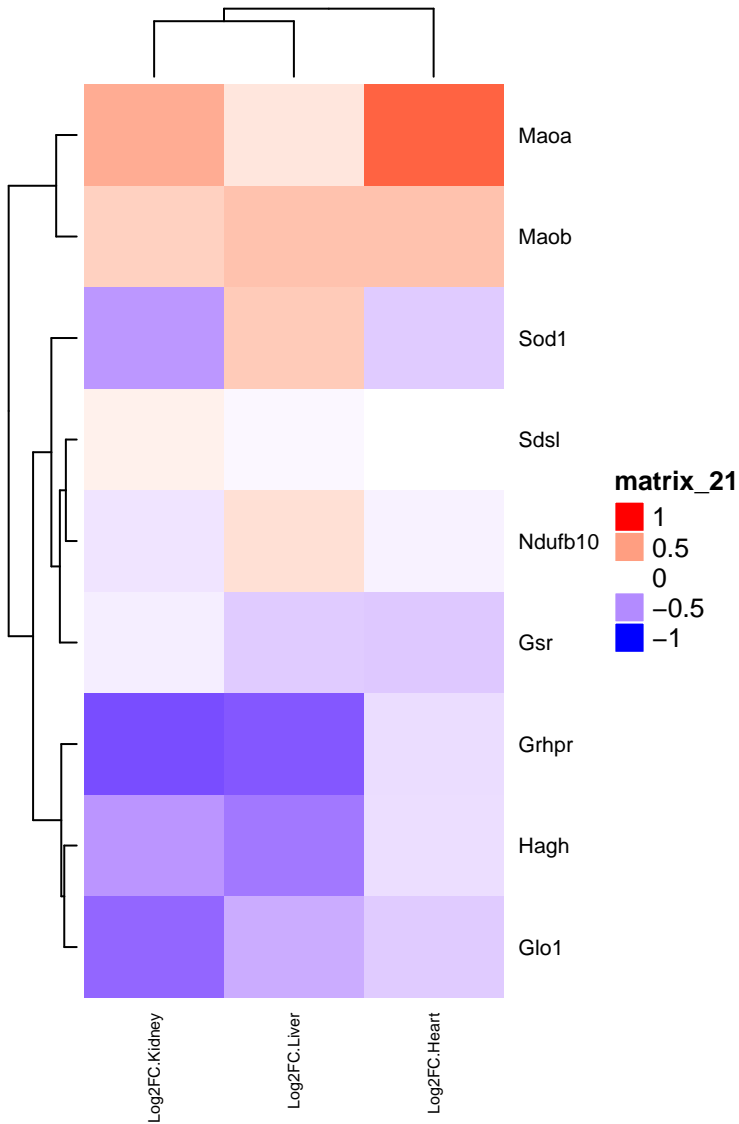

Supplement: Supplementary file 11 — Source Data [file 41467_2021_26982_MOESM11_ESM.zip › Figure 2/2F/Figure2_F.pdf]

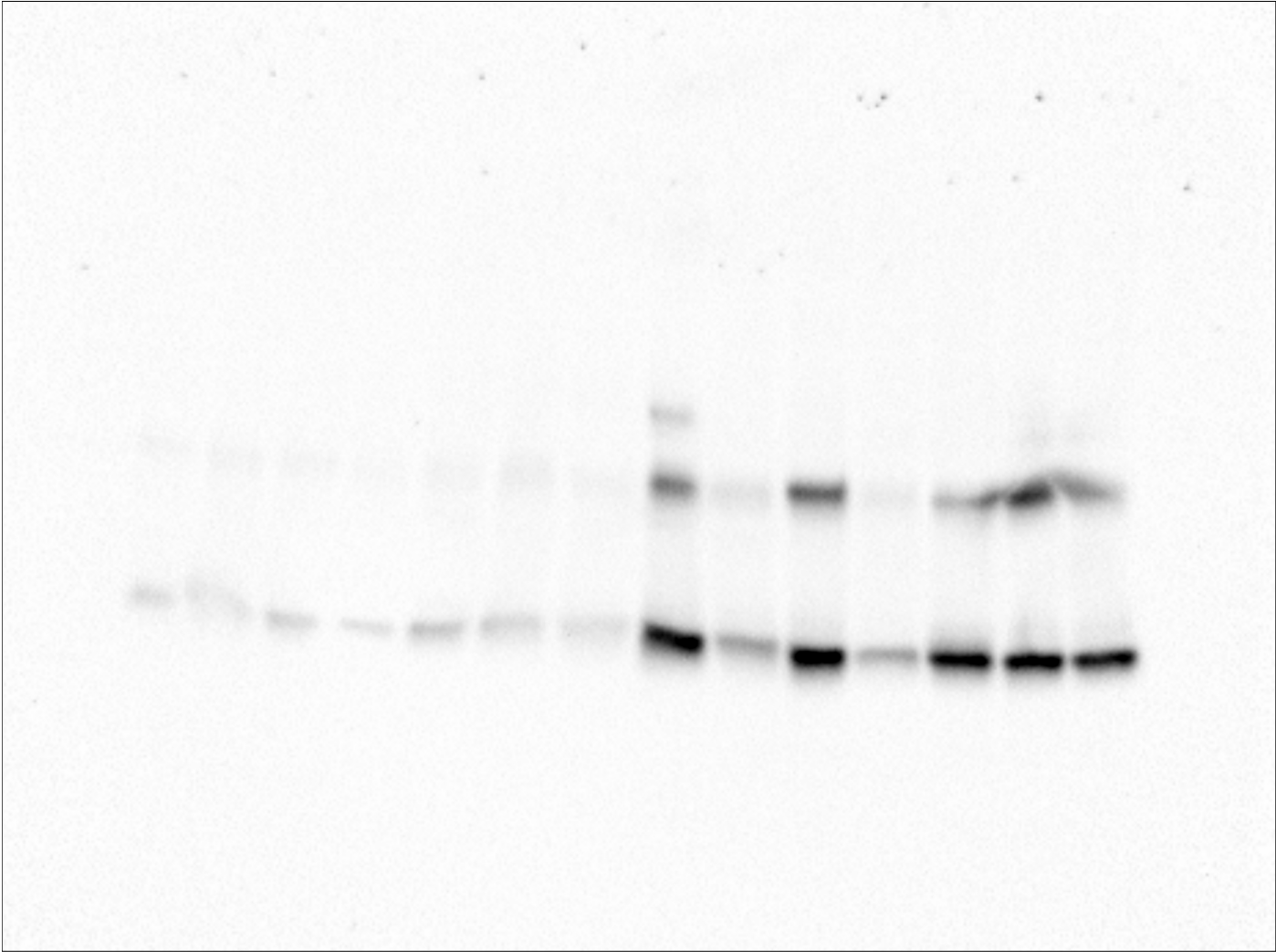

Supplement: Supplementary file 11 — Source Data [file 41467_2021_26982_MOESM11_ESM.zip › Figure 2/2G/bActin_Heart_Exposure_27.8sec.pdf]

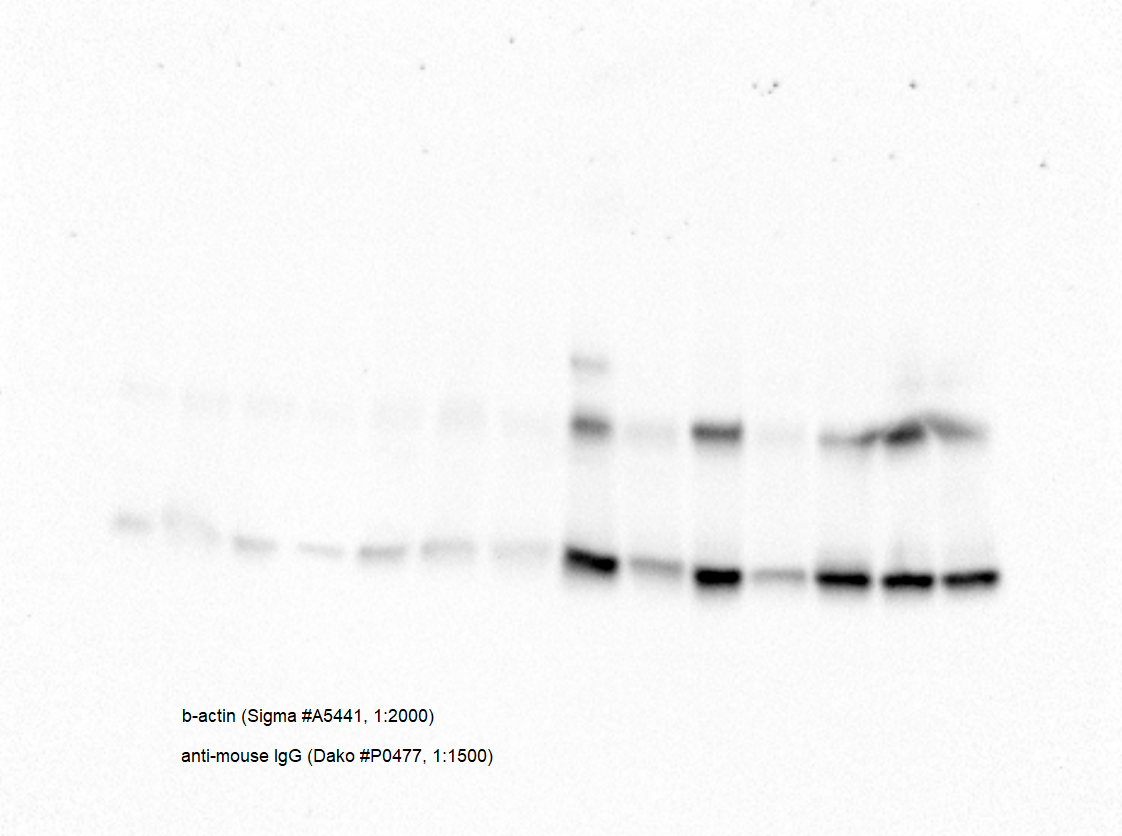

Supplement: Supplementary file 11 — Source Data [file 41467_2021_26982_MOESM11_ESM.zip › Figure 2/2G/bActin_Heart_Exposure_27.8sec.tif]

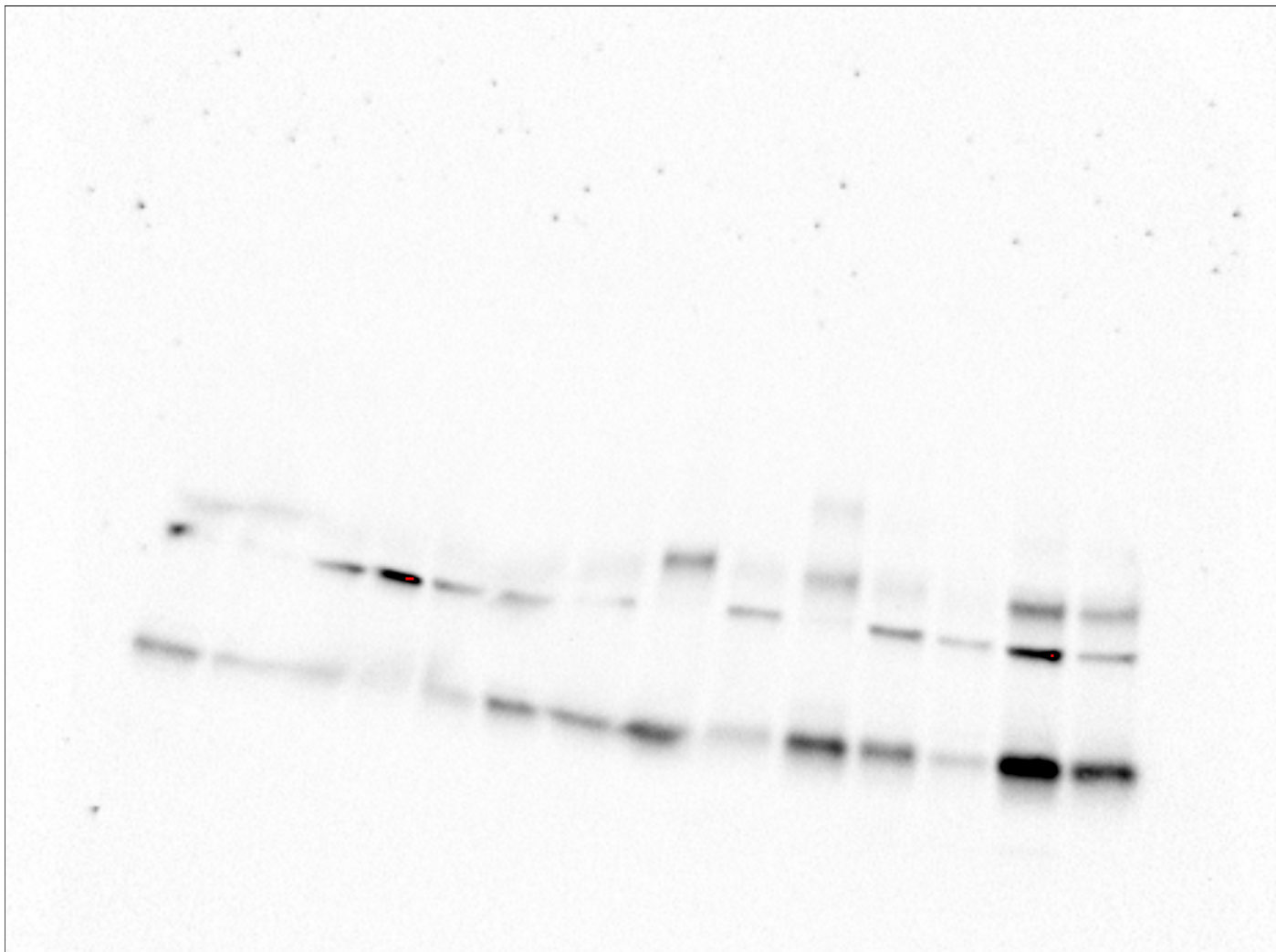

Supplement: Supplementary file 11 — Source Data [file 41467_2021_26982_MOESM11_ESM.zip › Figure 2/2G/bActin_Kidney_Exposure_27.8sec.pdf]

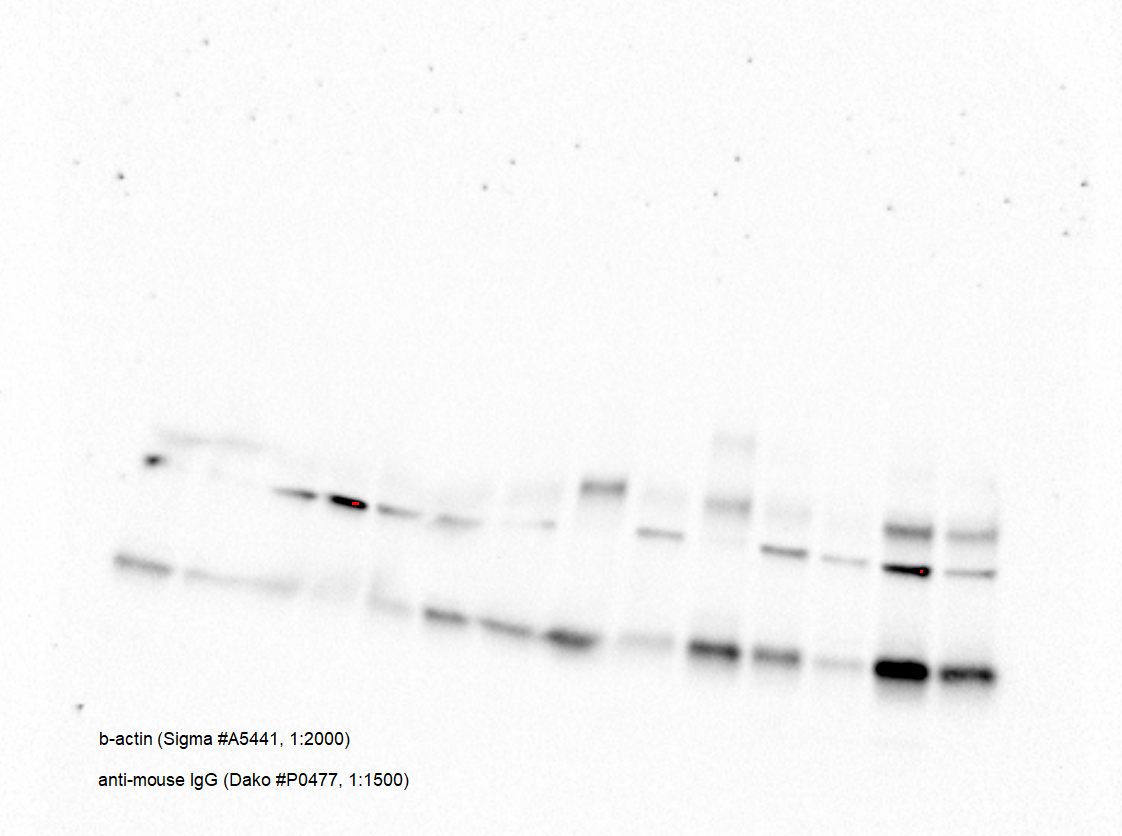

Supplement: Supplementary file 11 — Source Data [file 41467_2021_26982_MOESM11_ESM.zip › Figure 2/2G/bActin_Kidney_Exposure_27.8sec.tif]

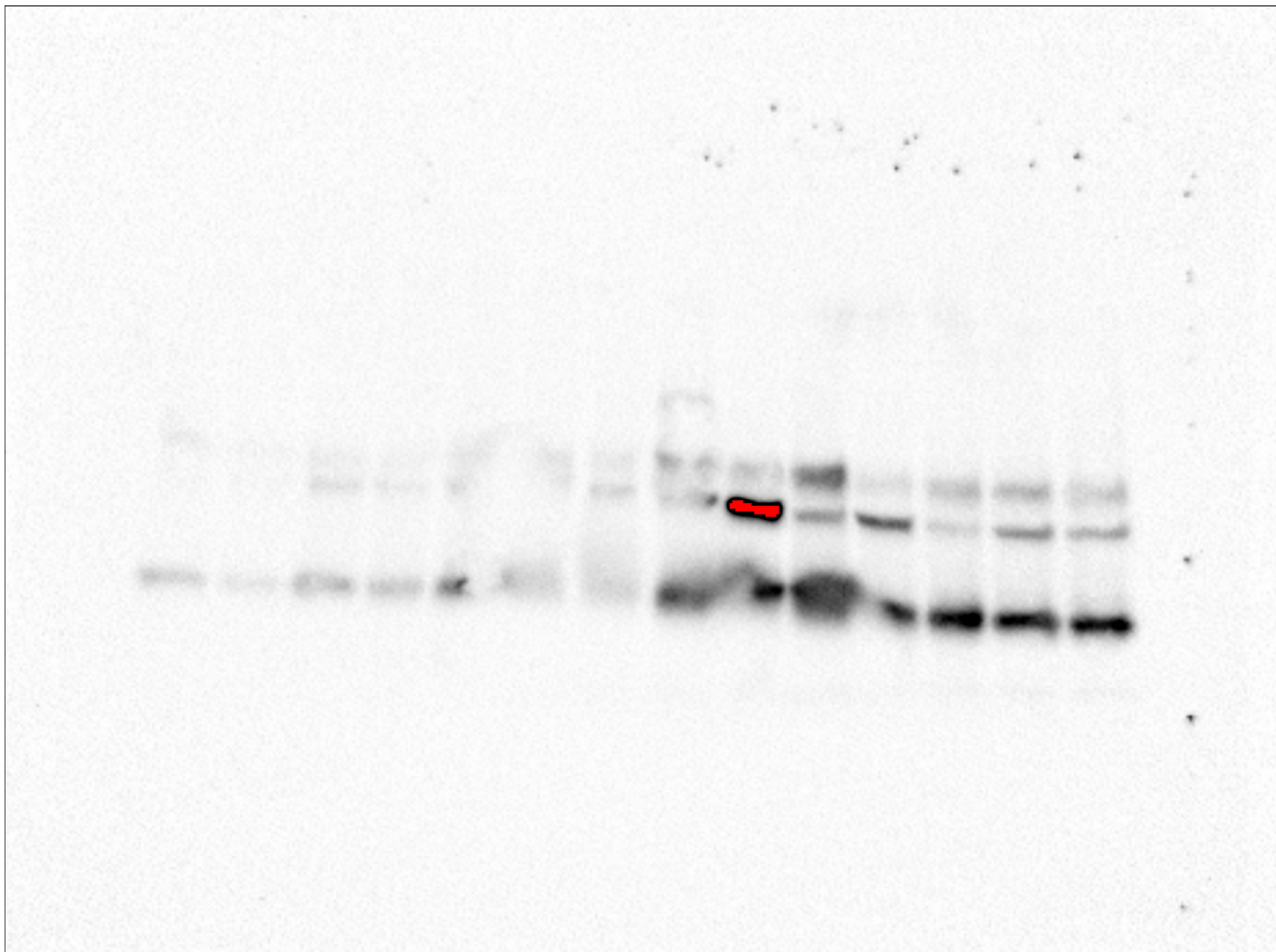

Supplement: Supplementary file 11 — Source Data [file 41467_2021_26982_MOESM11_ESM.zip › Figure 2/2G/bActin_Liver_Exposure_27.8sec.pdf]

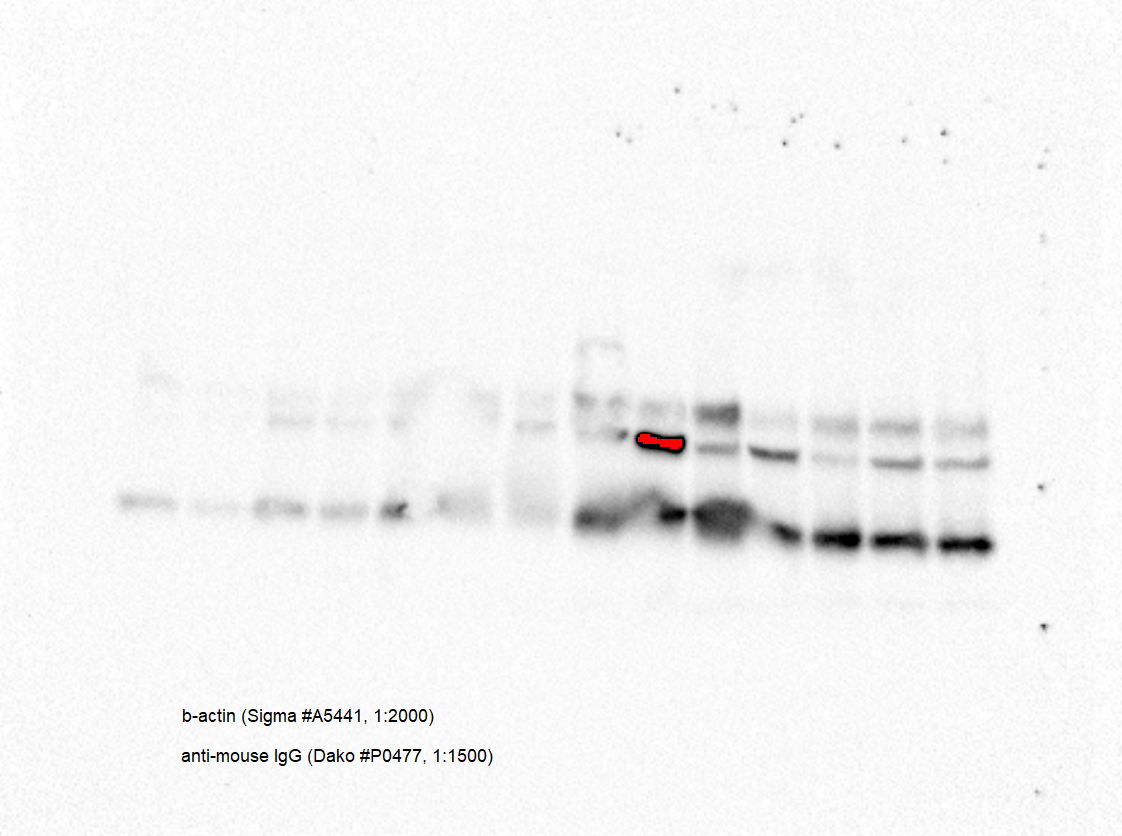

Supplement: Supplementary file 11 — Source Data [file 41467_2021_26982_MOESM11_ESM.zip › Figure 2/2G/bActin_Liver_Exposure_27.8sec.tif]

**Figure2\_G\_left (heart)**

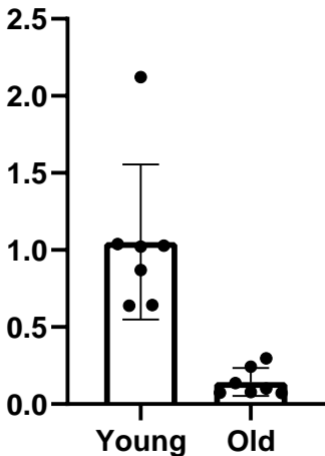

Supplement: Supplementary file 11 — Source Data [file 41467_2021_26982_MOESM11_ESM.zip › Figure 2/2G/Figure2_G_left (heart).pdf]

**Figure2\_G\_middle (kidney)**

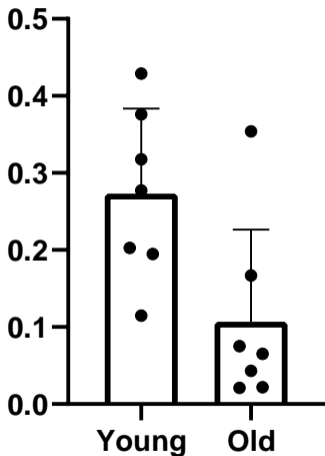

Supplement: Supplementary file 11 — Source Data [file 41467_2021_26982_MOESM11_ESM.zip › Figure 2/2G/Figure2_G_middle (kidney).pdf]

**Figure2\_G\_right (liver)**

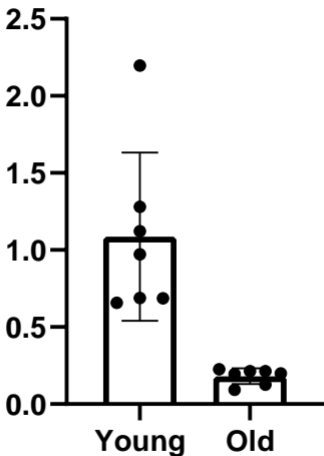

Supplement: Supplementary file 11 — Source Data [file 41467_2021_26982_MOESM11_ESM.zip › Figure 2/2G/Figure2_G_right (liver).pdf]

# Heart - Kidney - Liver (YvsO)

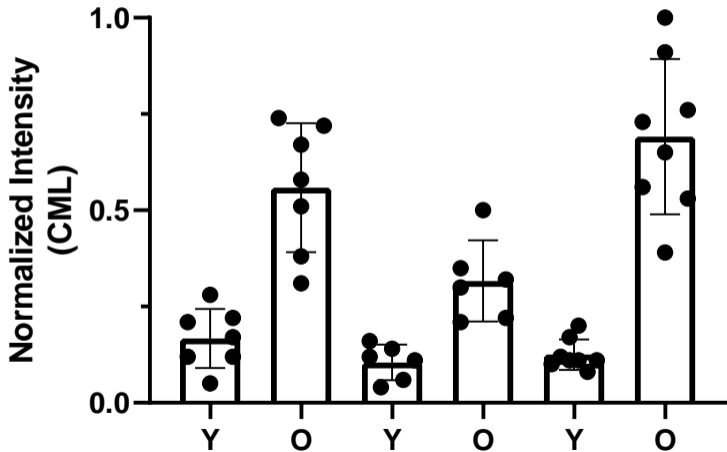

Supplement: Supplementary file 11 — Source Data [file 41467_2021_26982_MOESM11_ESM.zip › Figure 2/2H/Tissues together.pdf]

**TMT - Heart - Hist1h4a**

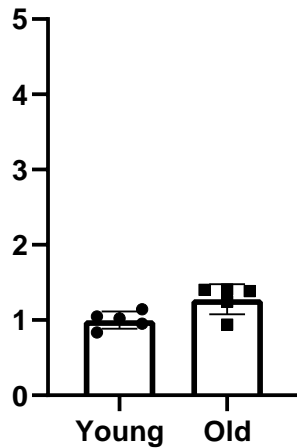

**PRM - Heart - Hist1h4a**

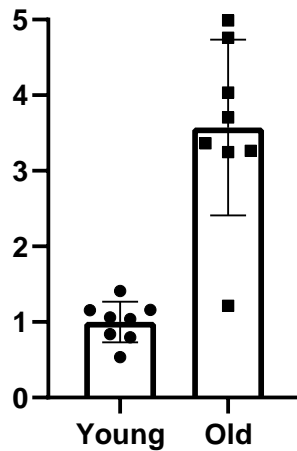

Supplement: Supplementary file 11 — Source Data [file 41467_2021_26982_MOESM11_ESM.zip › Figure 2/2I/Figure2_I_left.pdf]

**TMT - Kidney - Hist1h4a**

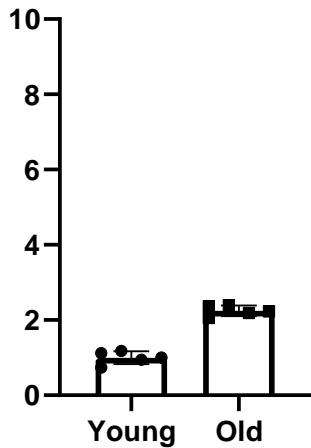

**PRM - Kidney - Hist1h4a**

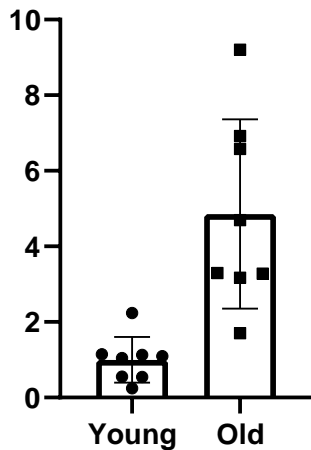

Supplement: Supplementary file 11 — Source Data [file 41467_2021_26982_MOESM11_ESM.zip › Figure 2/2I/Figure2_I_middle.pdf]

**TMT - Liver - Hist1h4a**

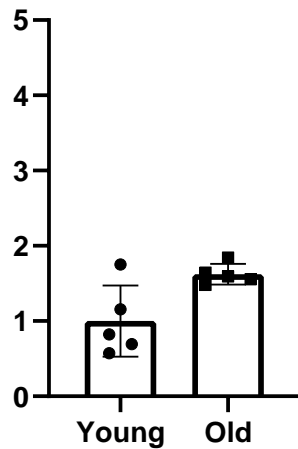

**PRM - Liver - Hist1h4a**

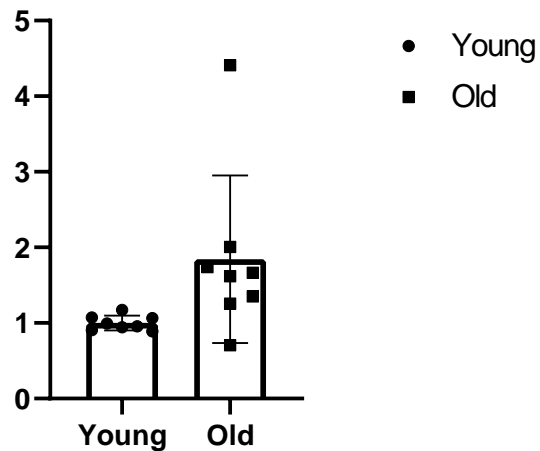

Supplement: Supplementary file 11 — Source Data [file 41467_2021_26982_MOESM11_ESM.zip › Figure 2/2I/Figure2_I_right.pdf]

**TMT - Heart - Slc25a4**

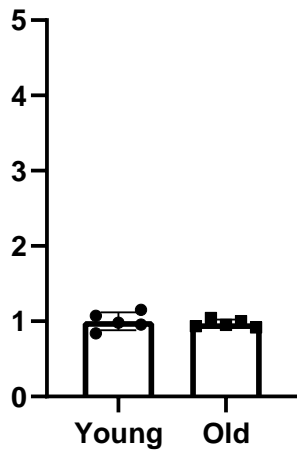

**PRM - Heart - Slc25a4**

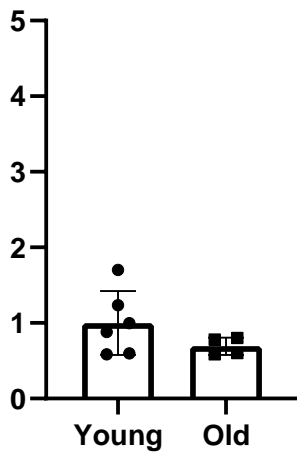

Supplement: Supplementary file 11 — Source Data [file 41467_2021_26982_MOESM11_ESM.zip › Figure 2/2J/Figure2_I_left.pdf]

**TMT - Heart - Atp5c1**

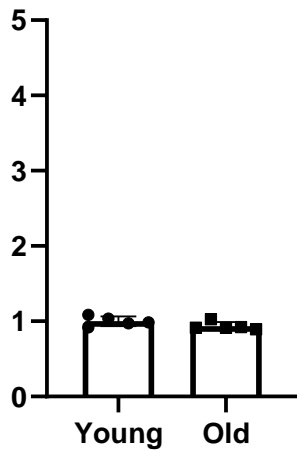

**PRM - Heart - Atp5c1**

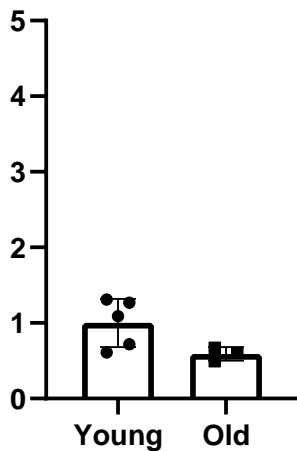

Supplement: Supplementary file 11 — Source Data [file 41467_2021_26982_MOESM11_ESM.zip › Figure 2/2J/Figure2_I_right.pdf]

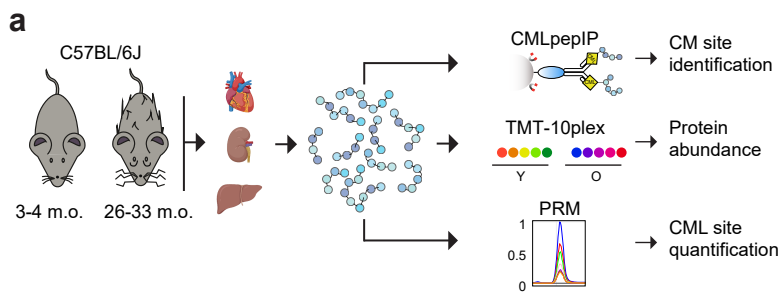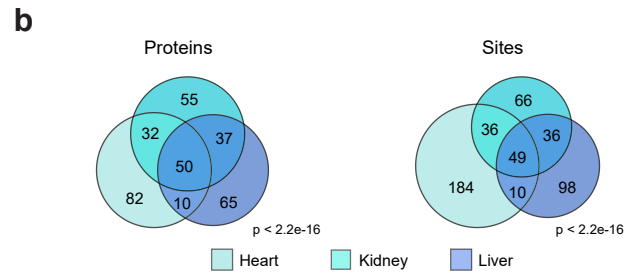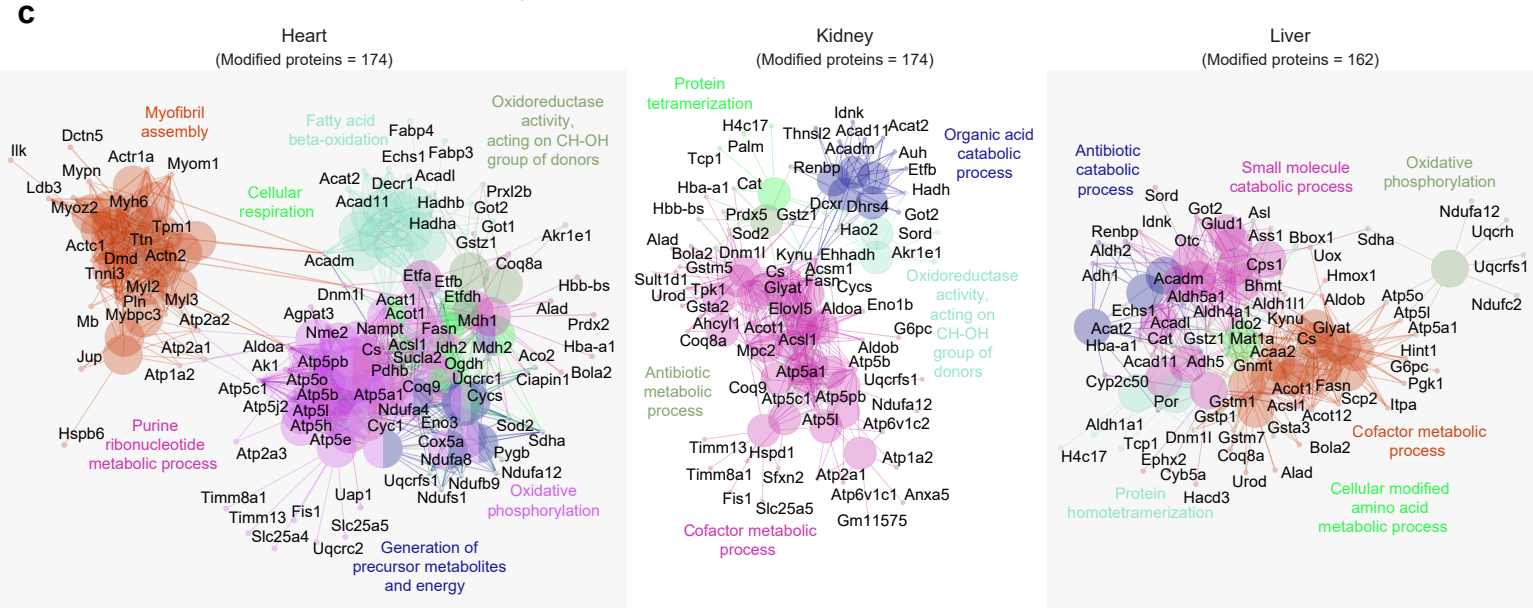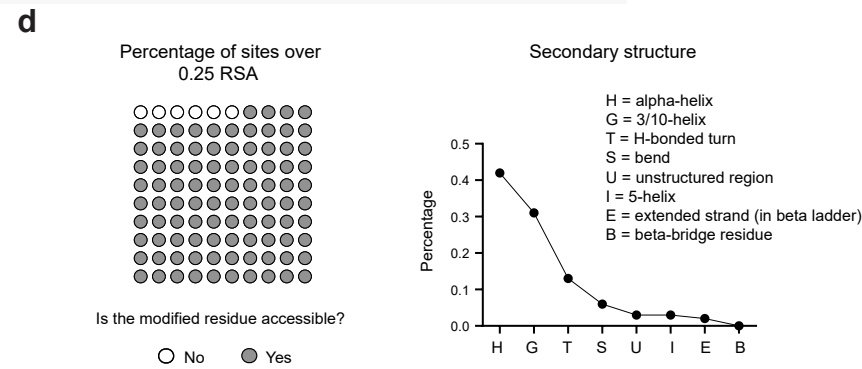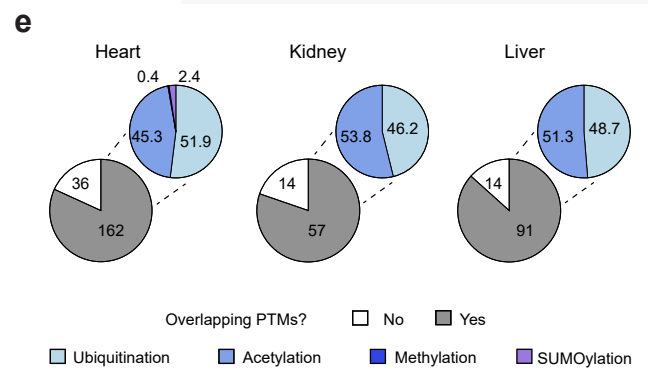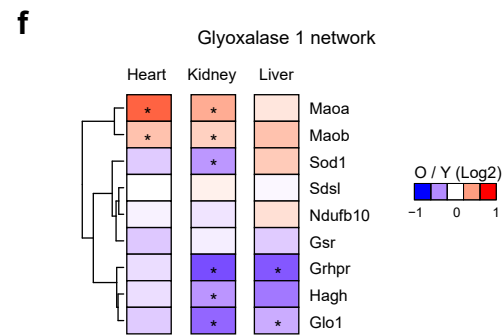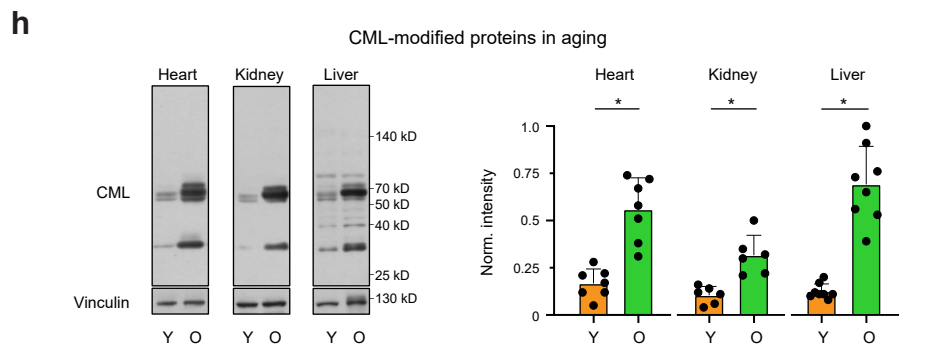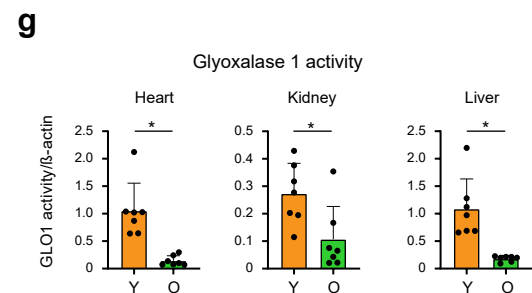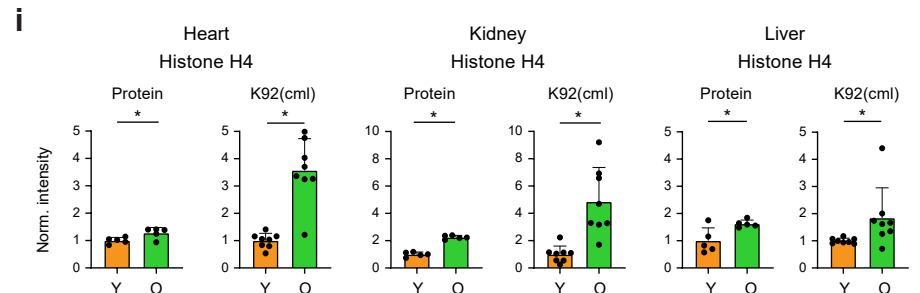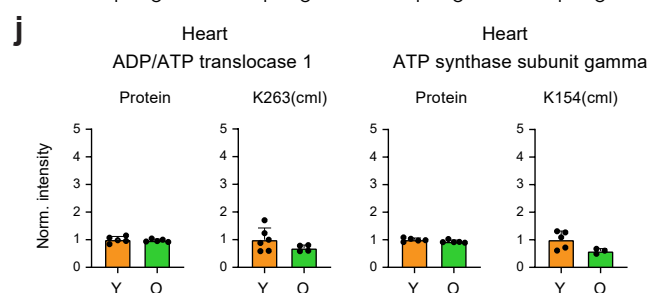

Supplement: Supplementary file 11 — Source Data [file 41467_2021_26982_MOESM11_ESM.zip › Figure 2/Figure2.pdf]

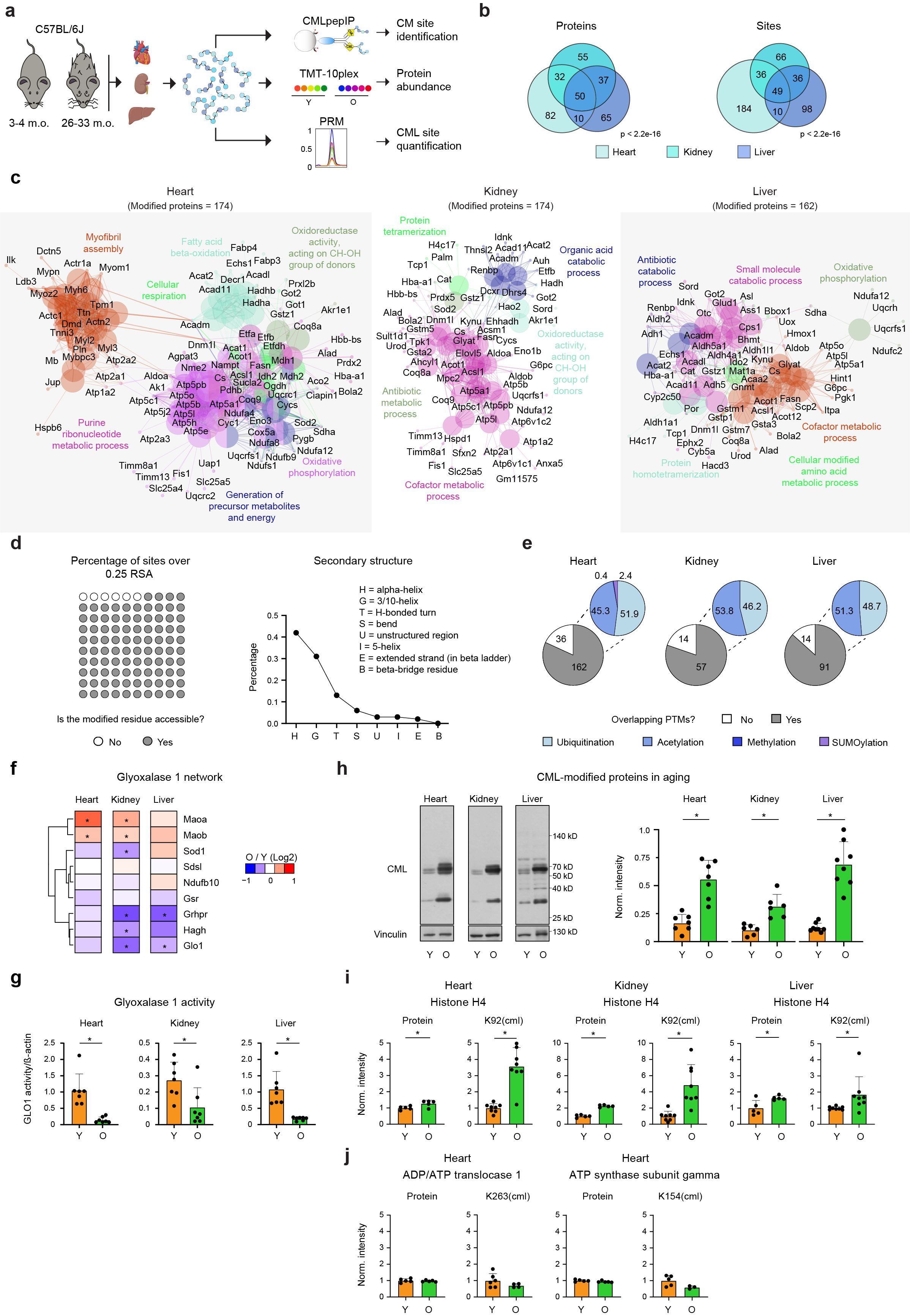

Supplement: Supplementary file 11 — Source Data [file 41467_2021_26982_MOESM11_ESM.zip › Figure 2/Figure2.png]

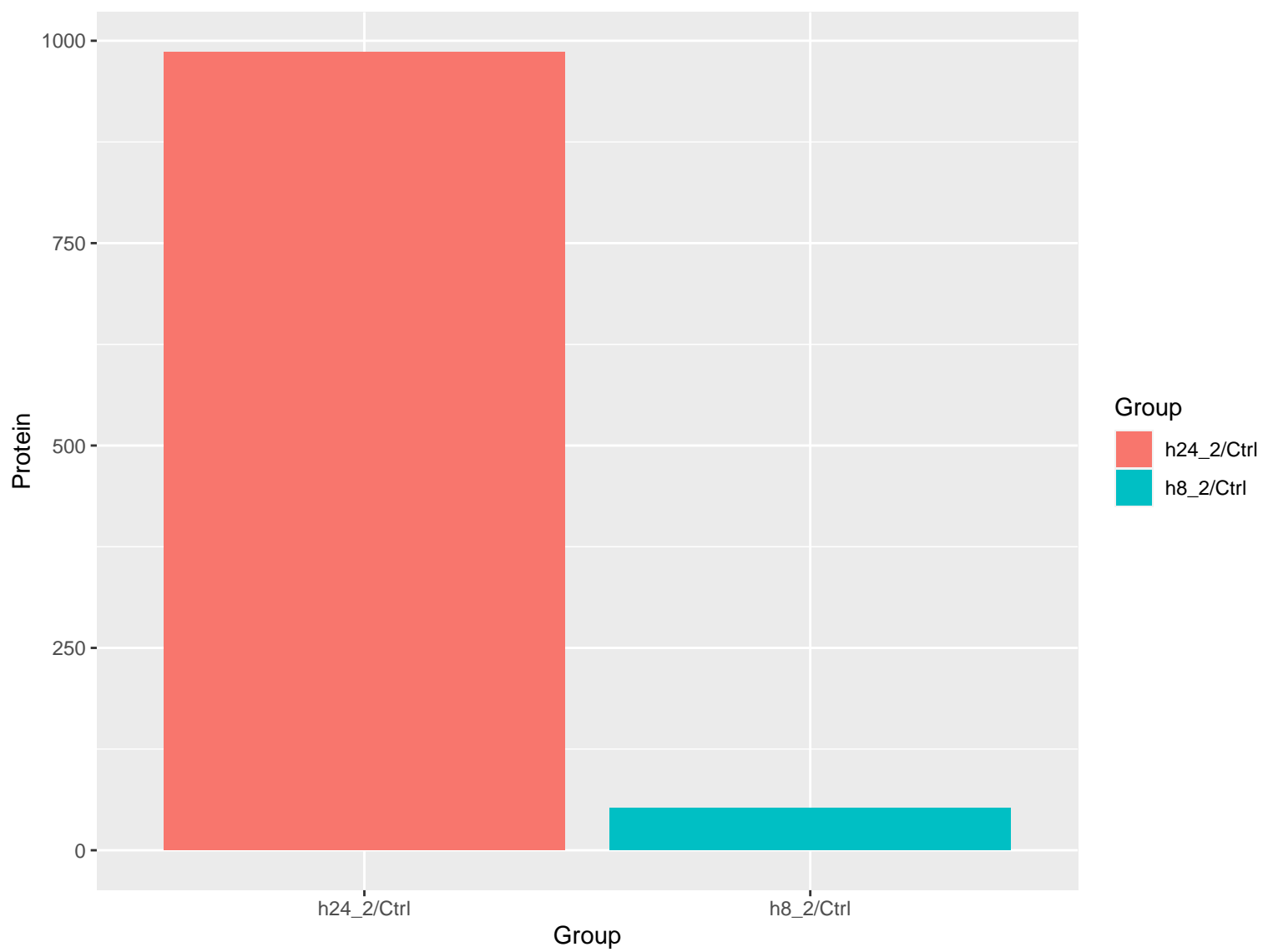

Supplement: Supplementary file 11 — Source Data [file 41467_2021_26982_MOESM11_ESM.zip › Figure 3/3B/Figure3_B_bottom_left.pdf]

H8\_2mM\_vs\_Ctrl[index, "X.DEPRECATED..AVG.Log2.Ratio"]

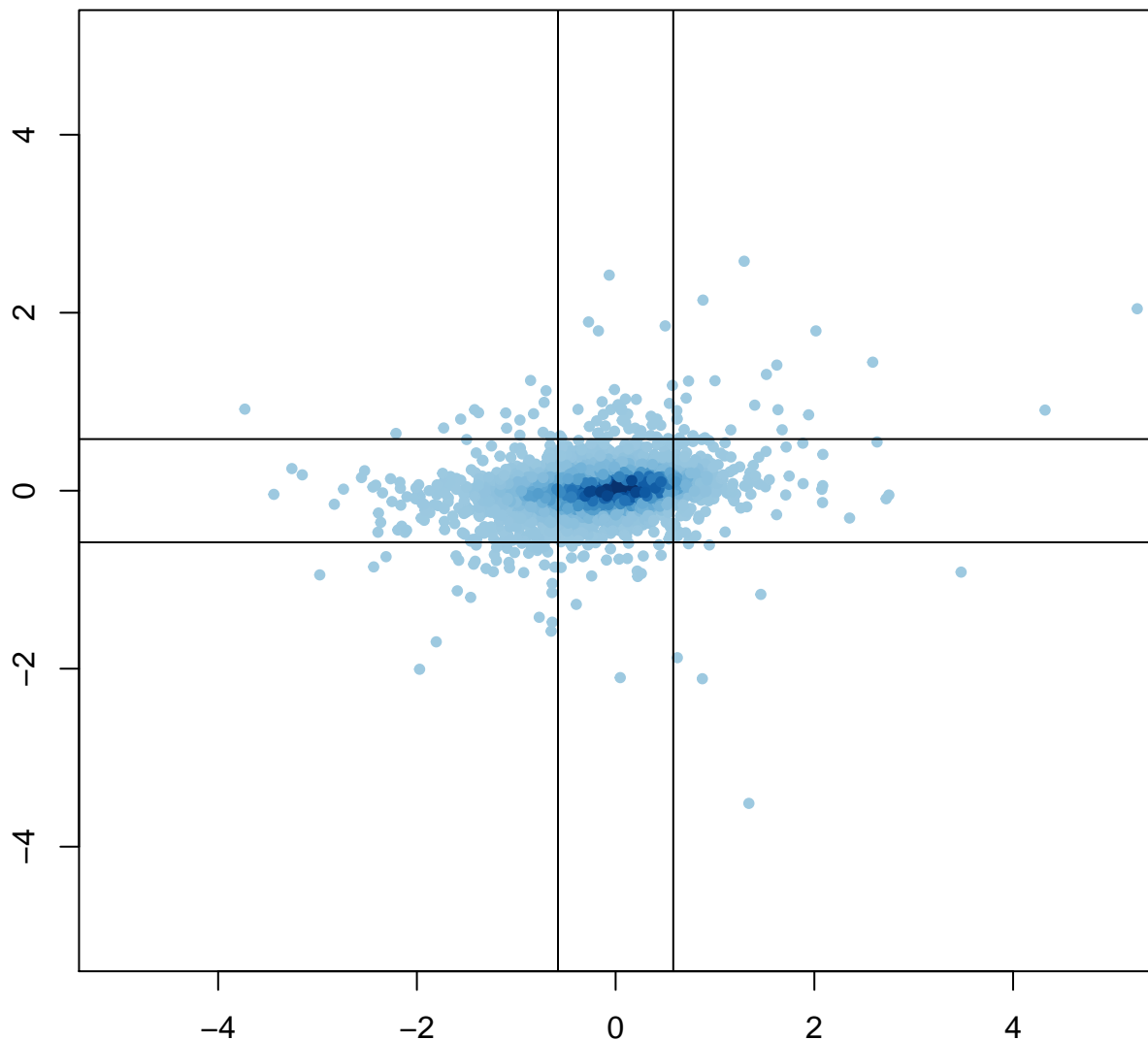

H24\_2mM\_vs\_Ctrl[index, "X.DEPRECATED..AVG.Log2.Ratio"]

Supplement: Supplementary file 11 — Source Data [file 41467_2021_26982_MOESM11_ESM.zip › Figure 3/3B/Figure3_B_bottom_right.pdf]

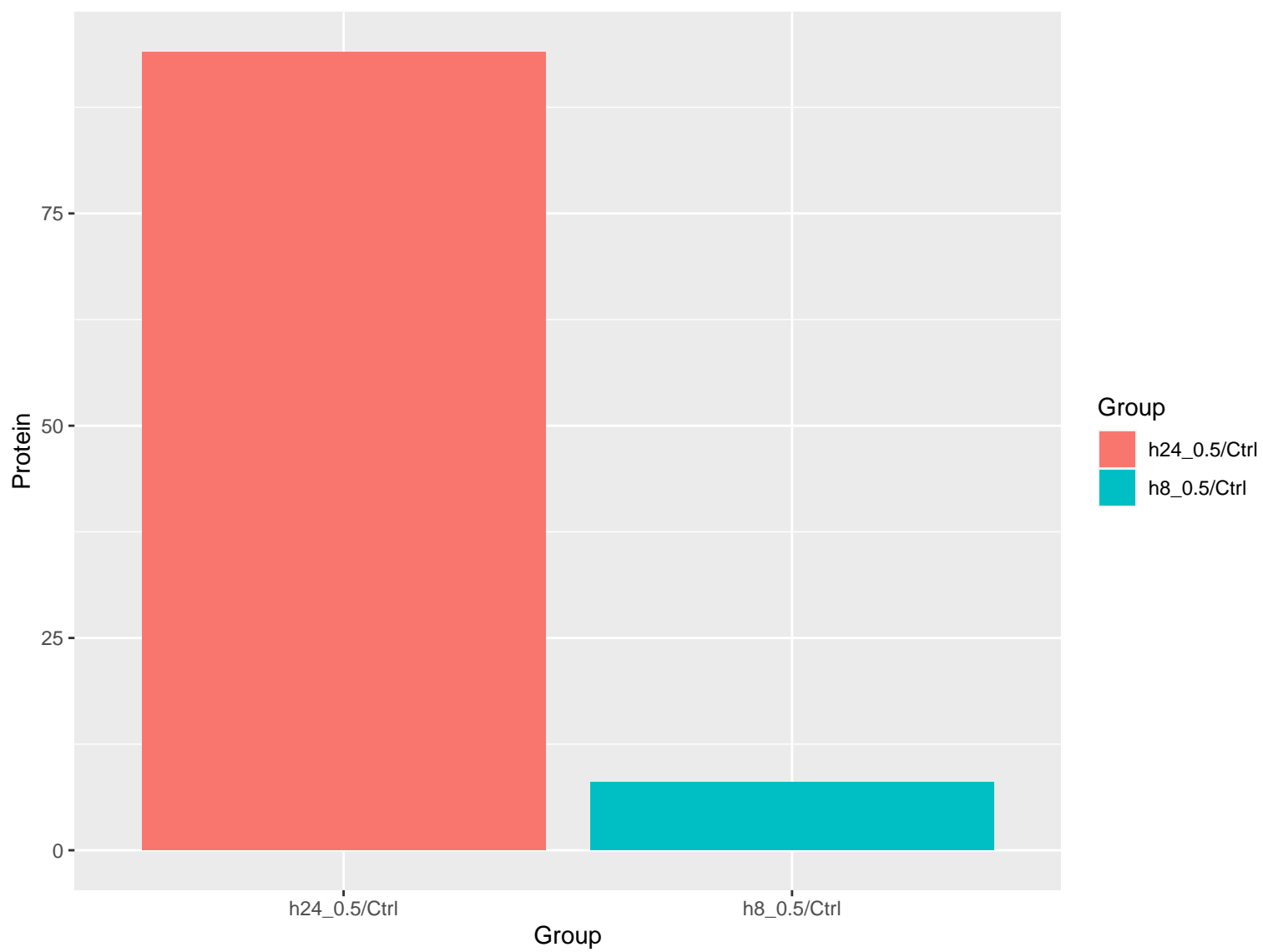

Supplement: Supplementary file 11 — Source Data [file 41467_2021_26982_MOESM11_ESM.zip › Figure 3/3B/Figure3_B_upper_left.pdf]

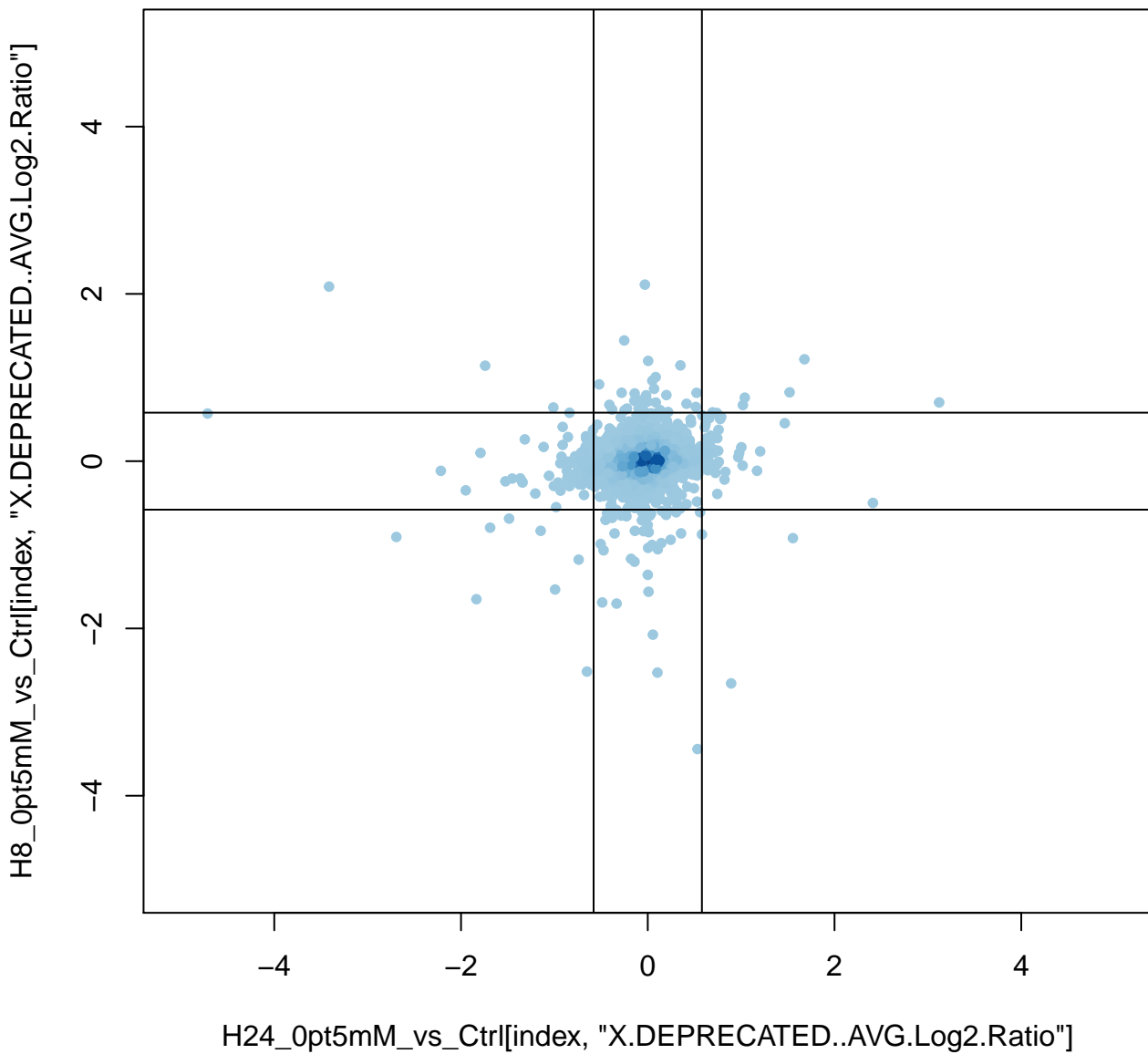

Supplement: Supplementary file 11 — Source Data [file 41467_2021_26982_MOESM11_ESM.zip › Figure 3/3B/Figure3_B_upper_right.pdf]

## All Protein expression vs CM modif

DEPRECATED.AVG.Log2.Ratio

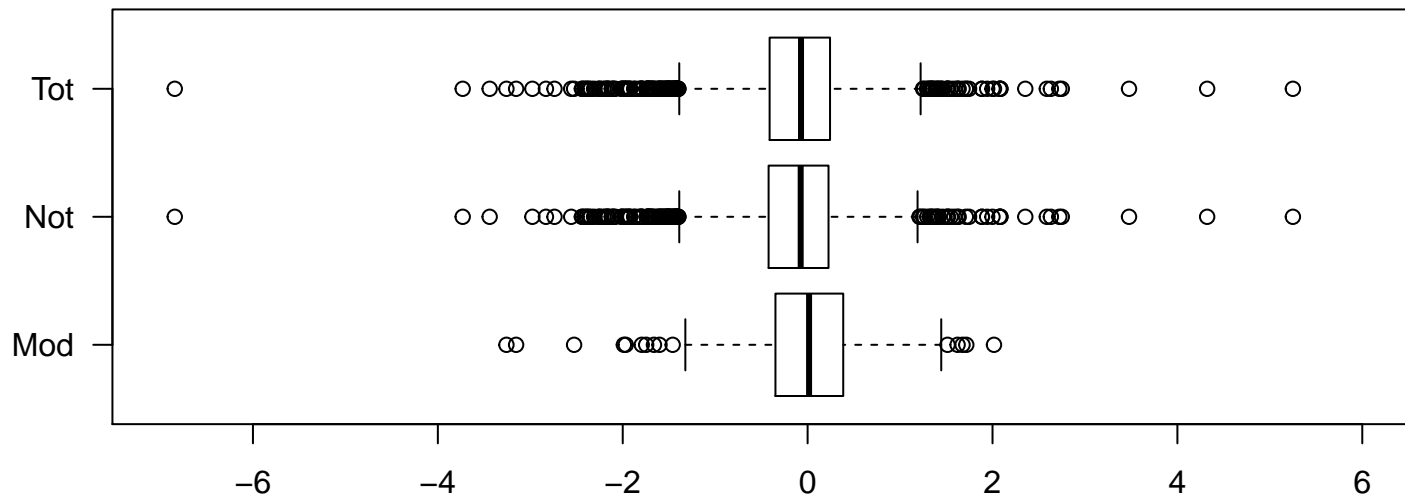

Supplement: Supplementary file 11 — Source Data [file 41467_2021_26982_MOESM11_ESM.zip › Figure 3/3C/200525_FC_2mM_vs_Ctrl_Modified_Protein_vs_totalProteome_24h.pdf]

# KEGG – 2mM vs Ctrl

reorder(description, -NES)

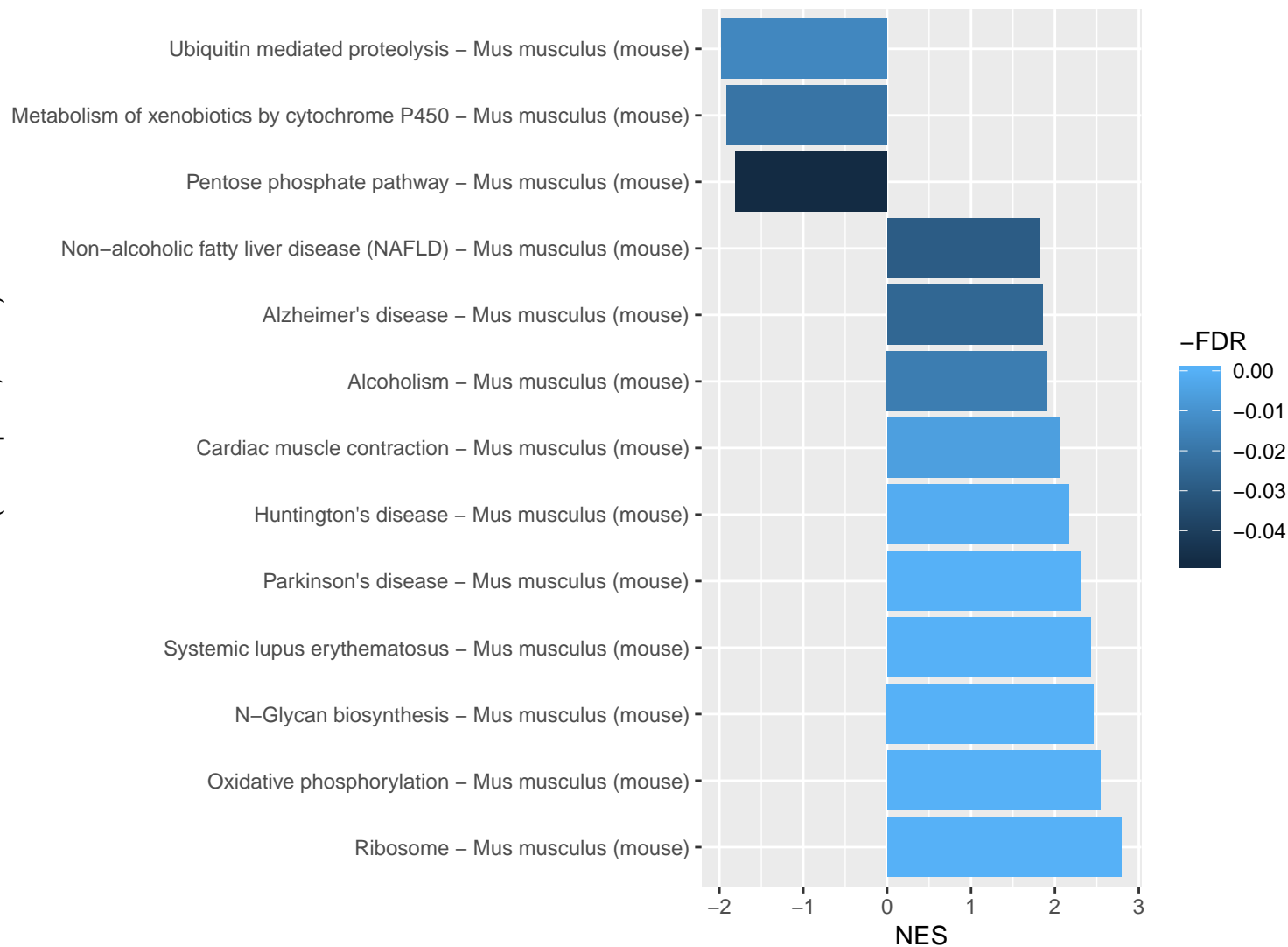

Supplement: Supplementary file 11 — Source Data [file 41467_2021_26982_MOESM11_ESM.zip › Figure 3/3D/Figure3_D.pdf]

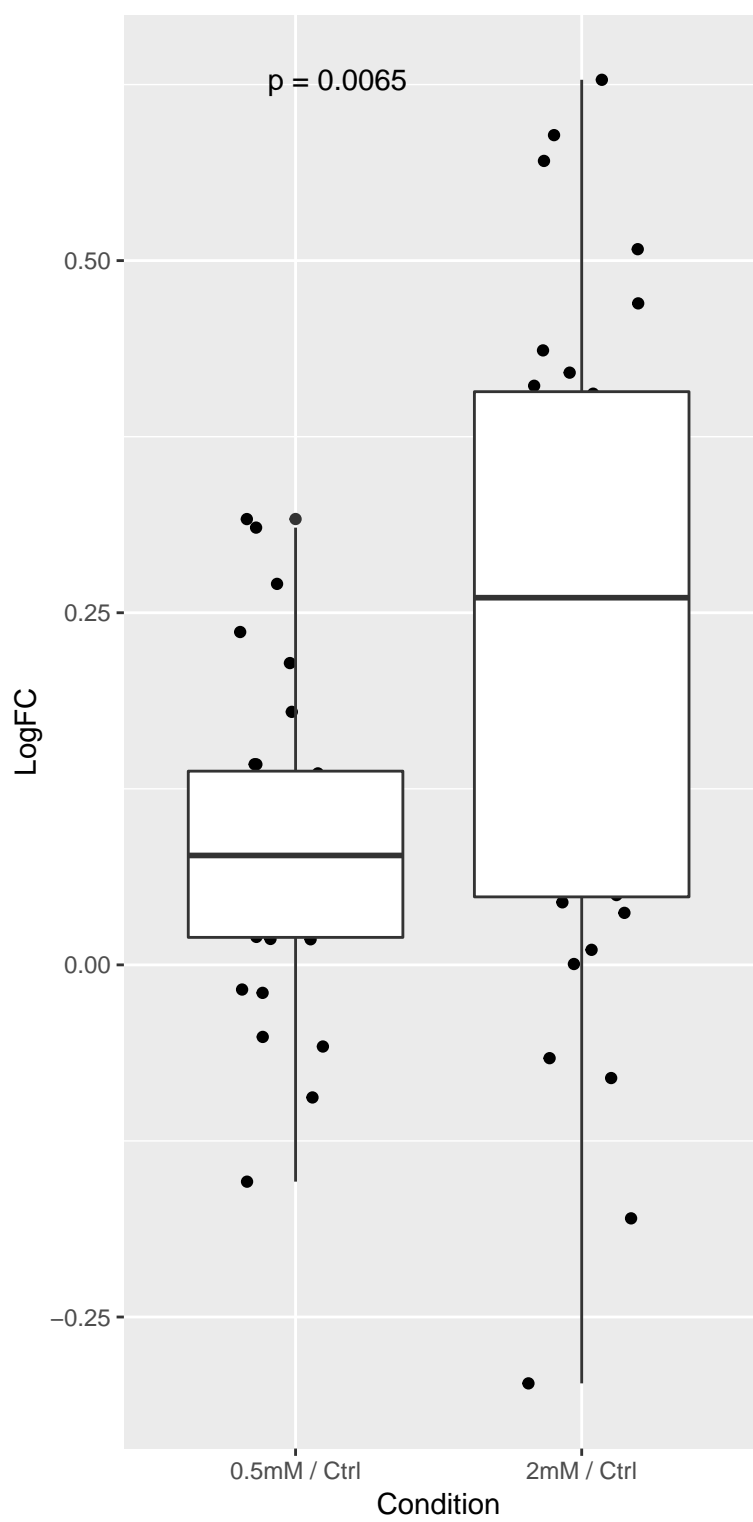

Supplement: Supplementary file 11 — Source Data [file 41467_2021_26982_MOESM11_ESM.zip › Figure 3/3E/Figure3_E_left.pdf]

## 20S Proteasome Activity

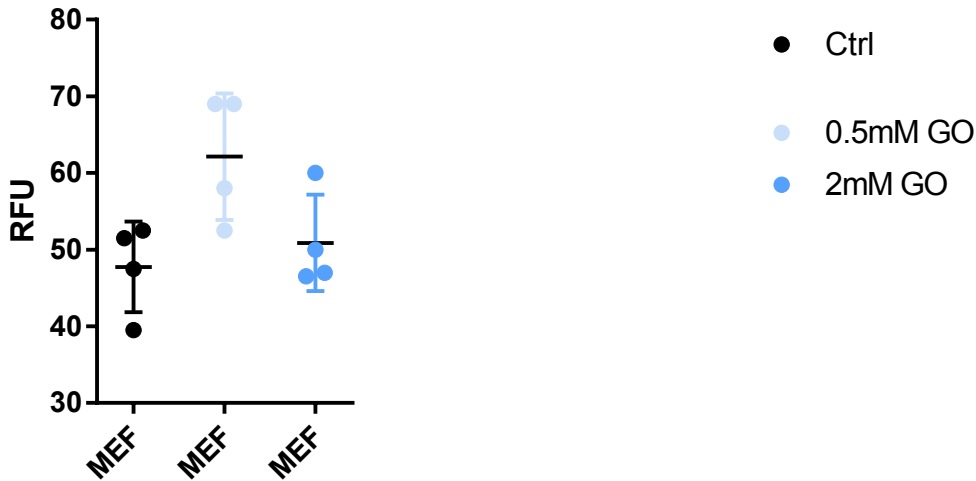

Supplement: Supplementary file 11 — Source Data [file 41467_2021_26982_MOESM11_ESM.zip › Figure 3/3E/Figure3E_right.pdf]

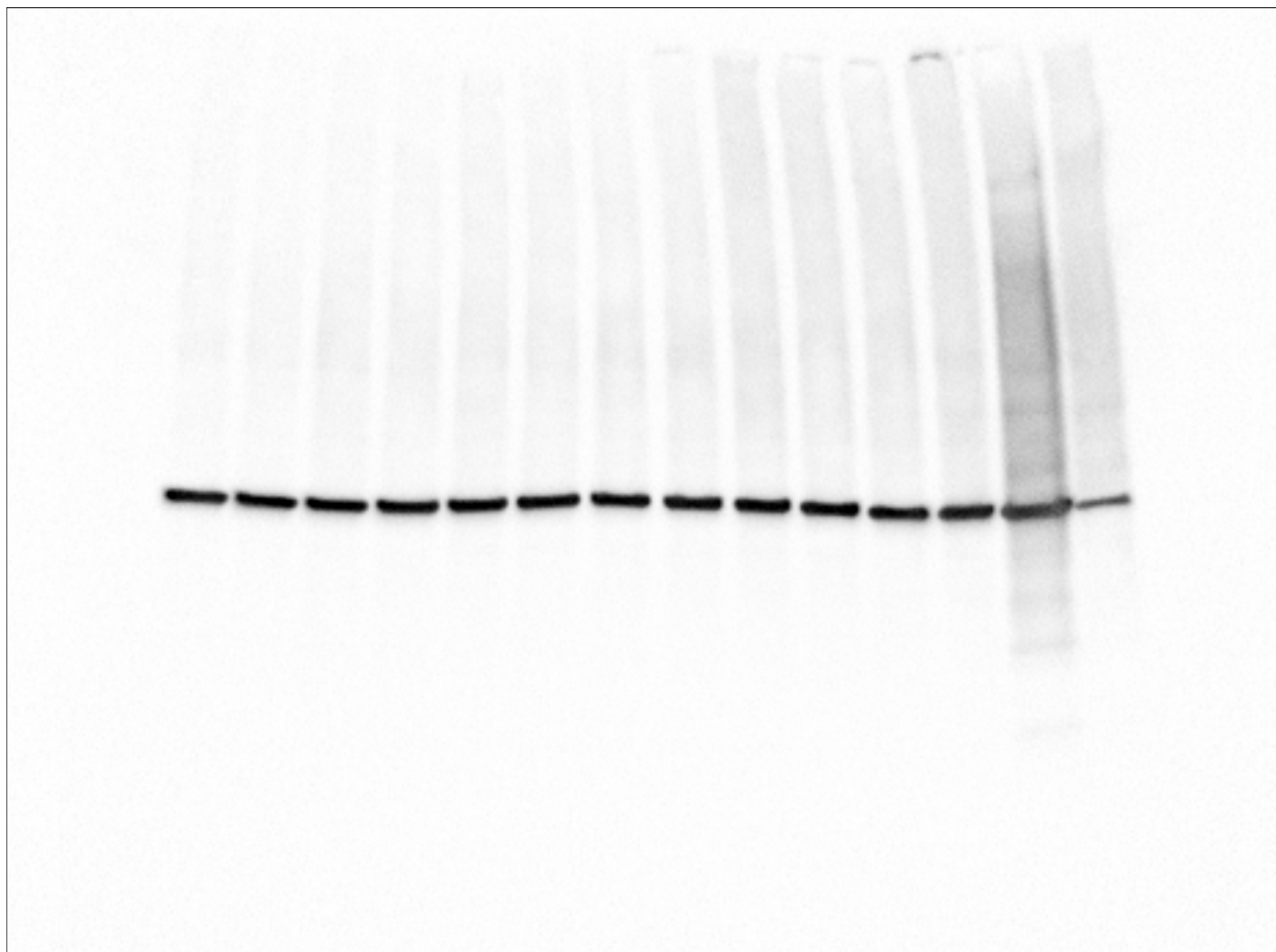

Supplement: Supplementary file 11 — Source Data [file 41467_2021_26982_MOESM11_ESM.zip › Figure 3/3F/bActin_Exposure_2.0sec.pdf]

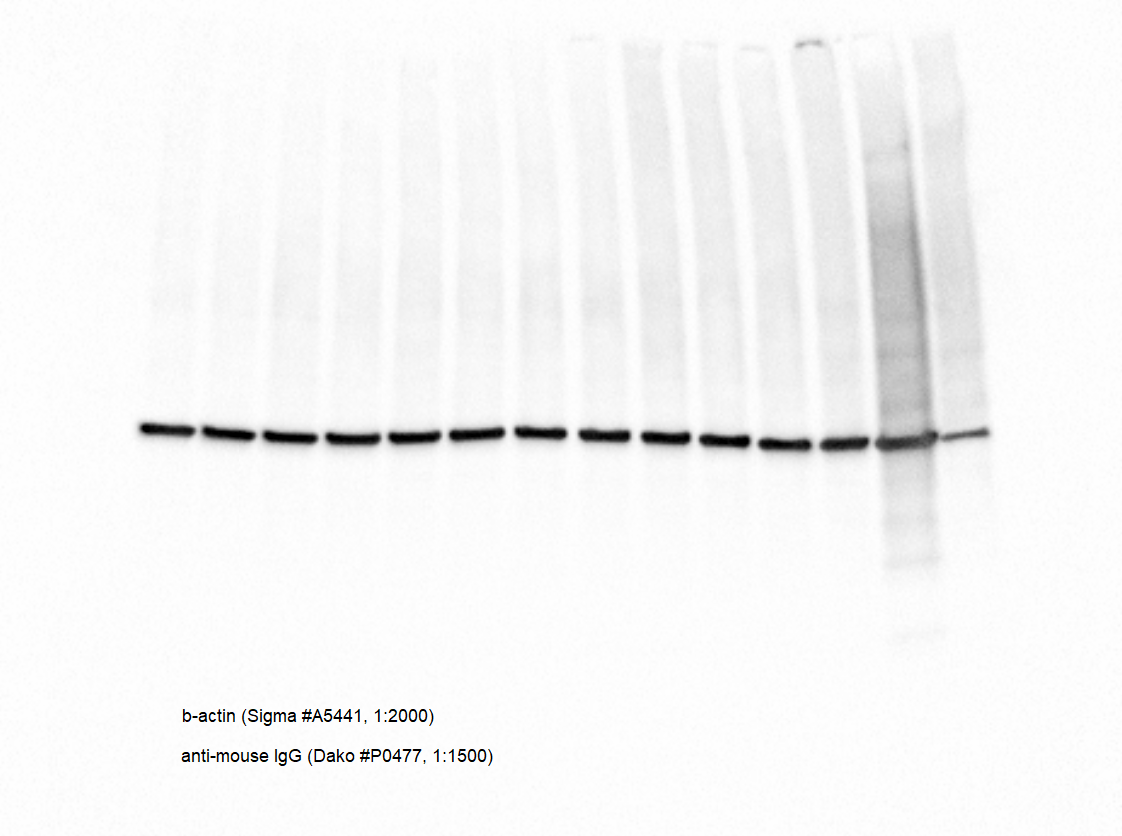

Supplement: Supplementary file 11 — Source Data [file 41467_2021_26982_MOESM11_ESM.zip › Figure 3/3F/bActin_Exposure_2.0sec.tif]

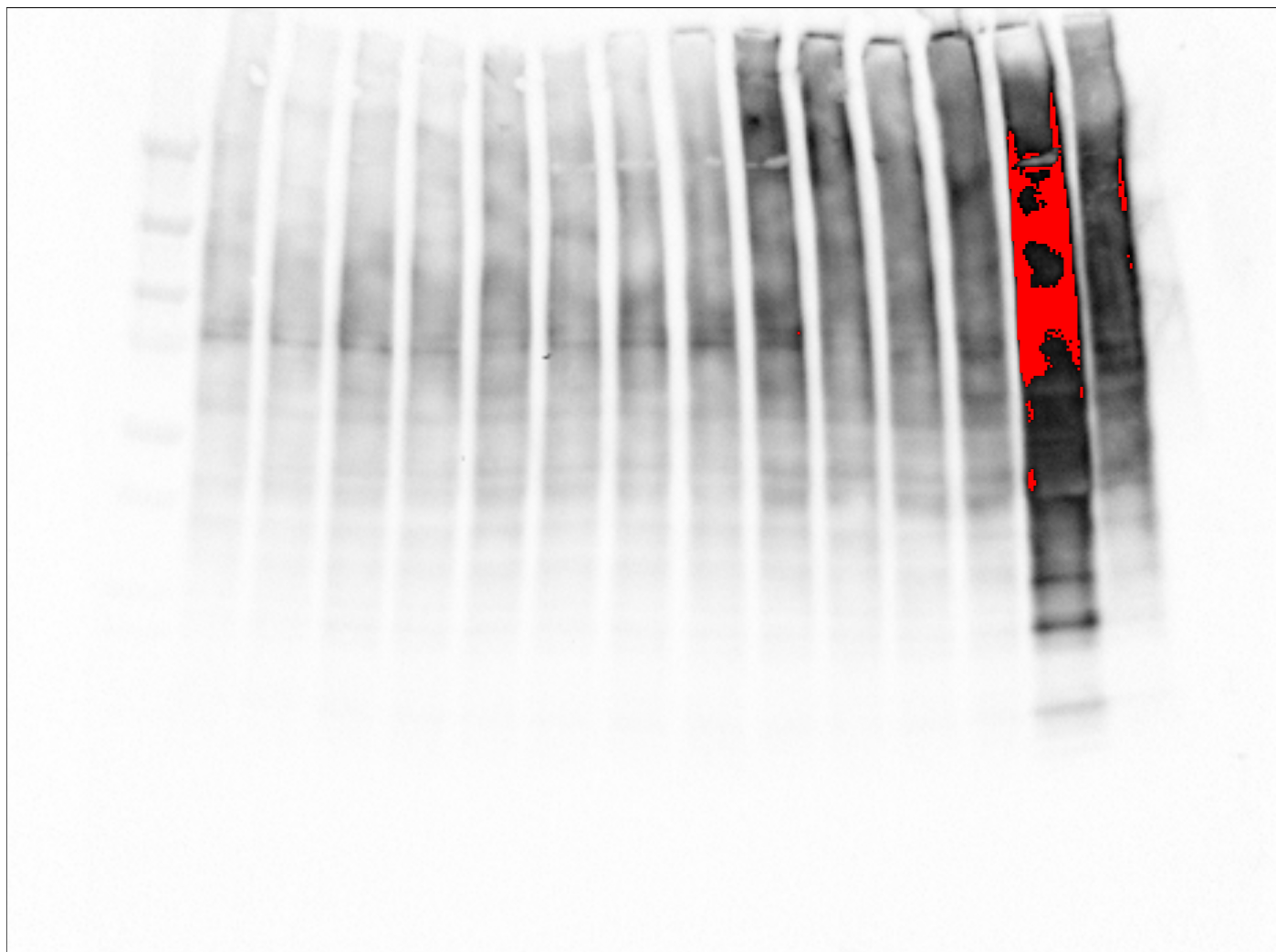

Supplement: Supplementary file 11 — Source Data [file 41467_2021_26982_MOESM11_ESM.zip › Figure 3/3F/Ubiquitin_Exposure_17.3sec.pdf]

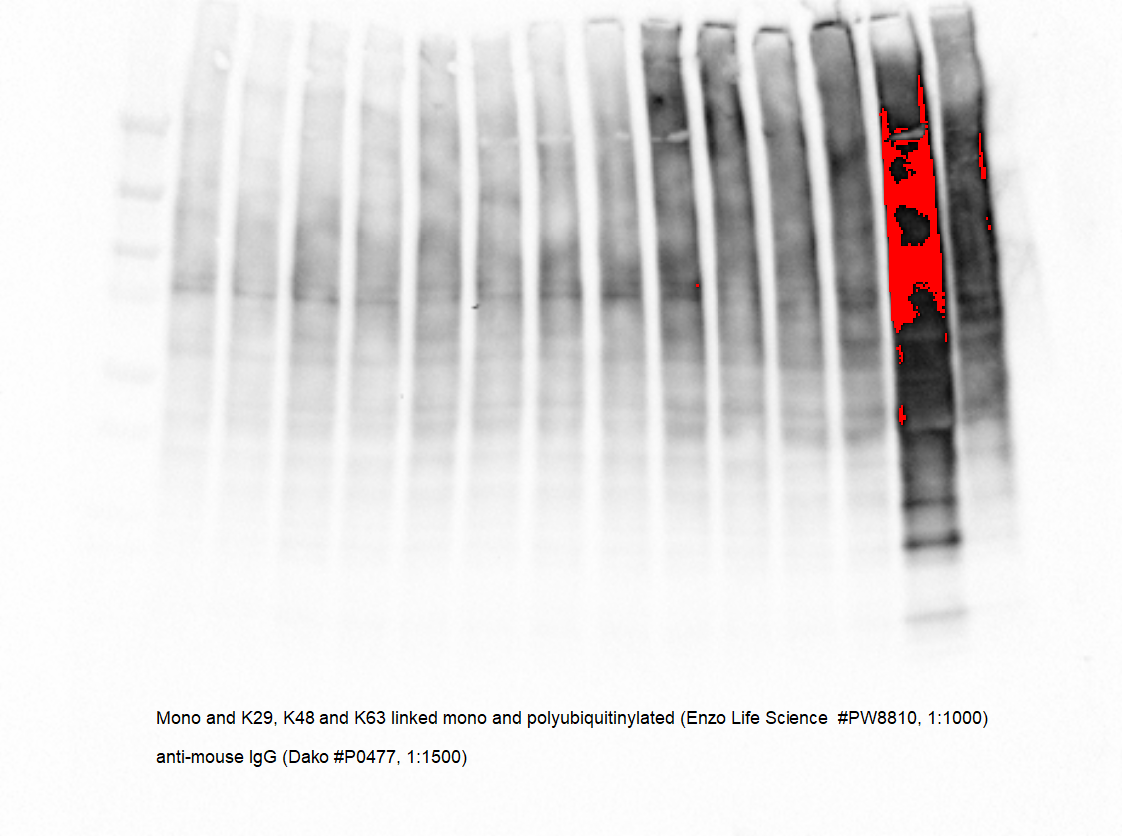

Supplement: Supplementary file 11 — Source Data [file 41467_2021_26982_MOESM11_ESM.zip › Figure 3/3F/Ubiquitin_Exposure_17.3sec.tif]

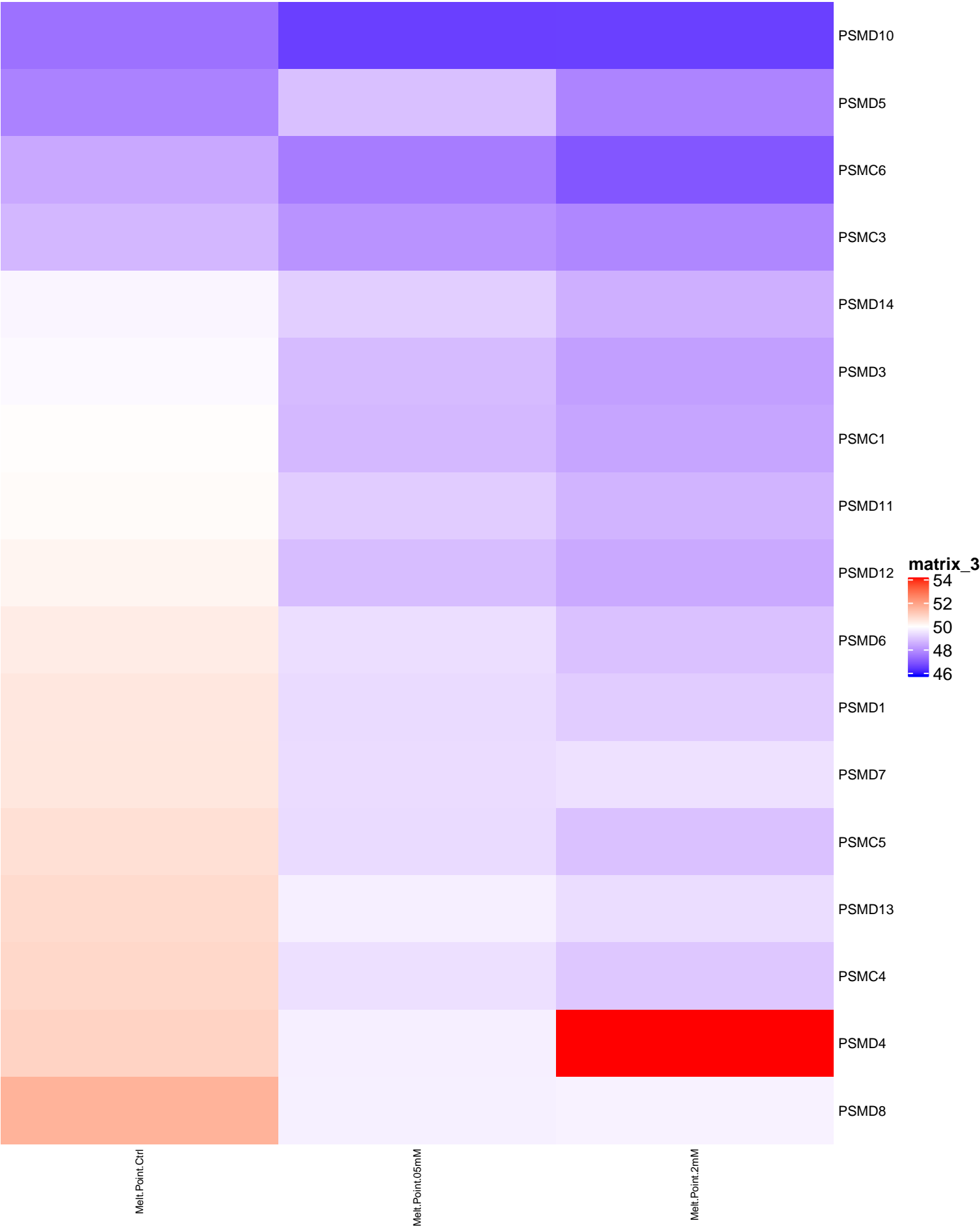

Supplement: Supplementary file 11 — Source Data [file 41467_2021_26982_MOESM11_ESM.zip › Figure 3/3G/Figure3_G.pdf]

CML (normalized to the ctrl on gels)

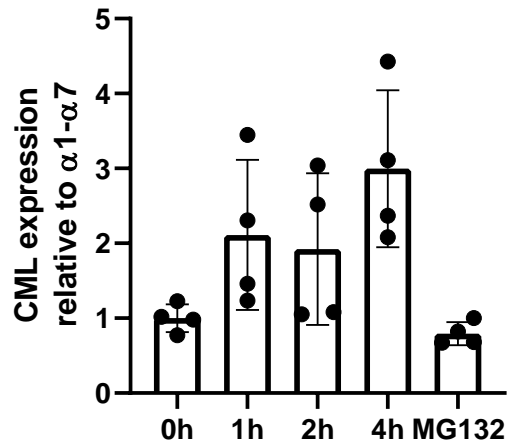

Activity (normalized to the ctrl on gels)

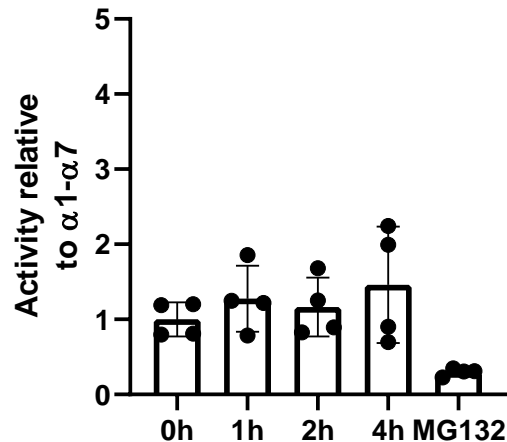

Supplement: Supplementary file 11 — Source Data [file 41467_2021_26982_MOESM11_ESM.zip › Figure 3/3H/Normalized to the ctrl on the gels.pdf]

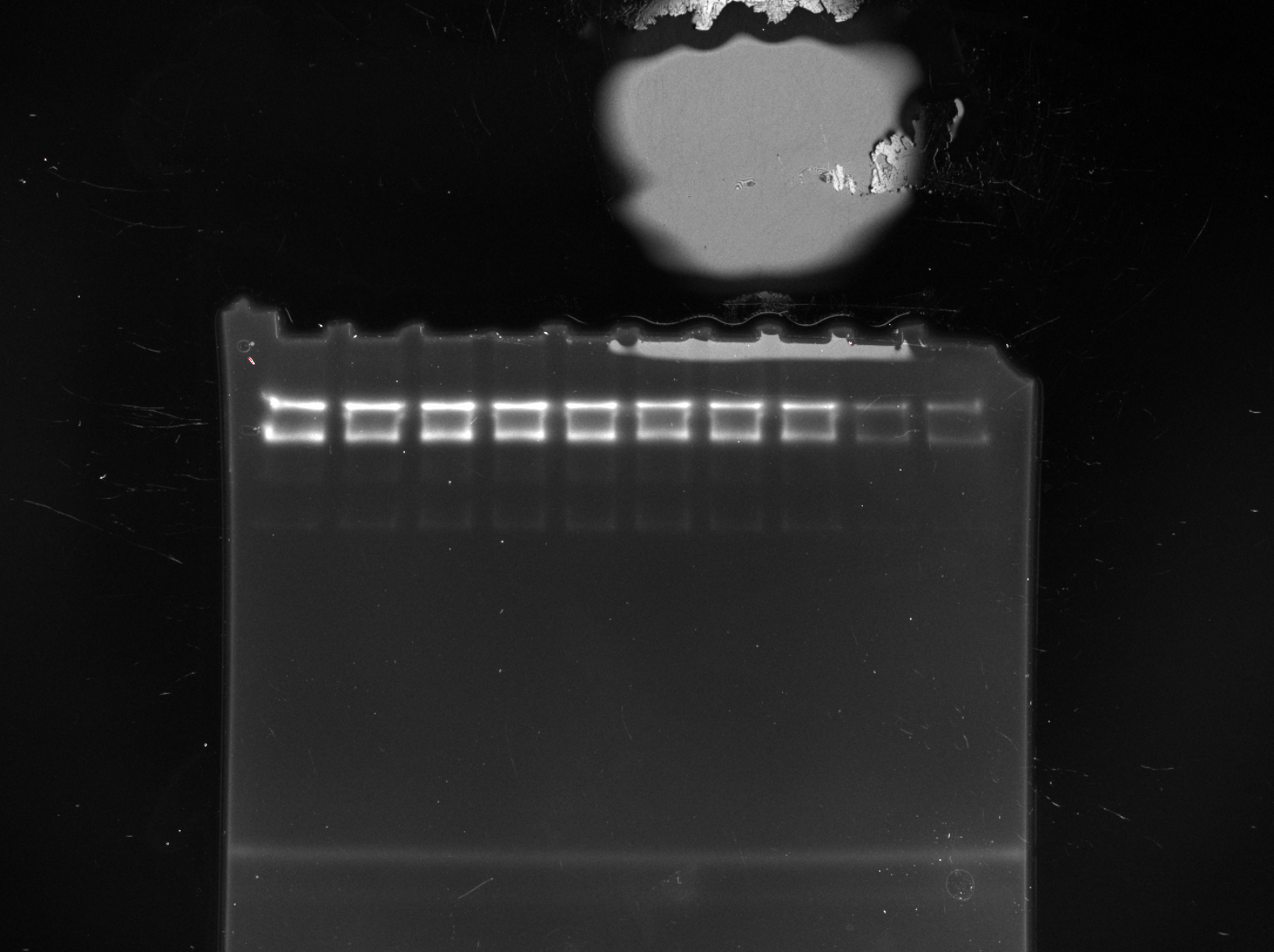

Supplement: Supplementary file 11 — Source Data [file 41467_2021_26982_MOESM11_ESM.zip › Figure 3/3H/rep1_2/Activity/research_ori 2021-06-07_16h44m22s_2sec.tif]

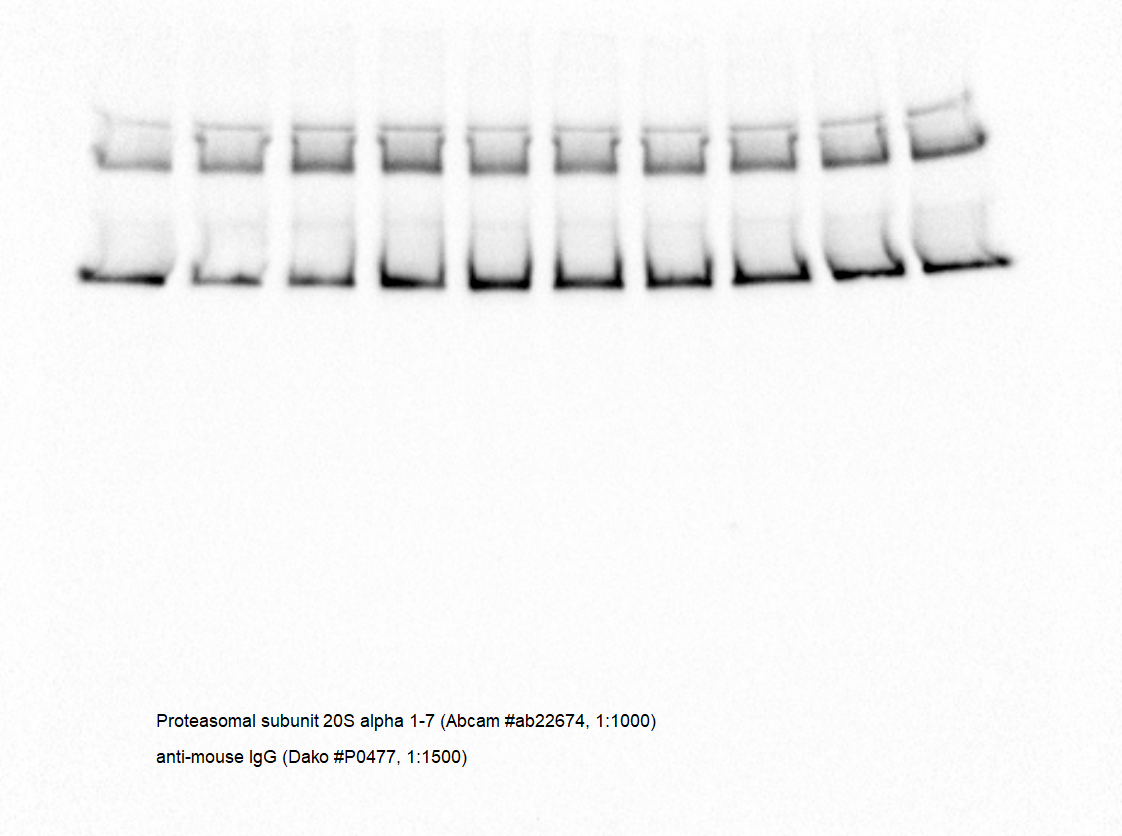

Supplement: Supplementary file 11 — Source Data [file 41467_2021_26982_MOESM11_ESM.zip › Figure 3/3H/rep1_2/Alpha1_7/research_ori 2021-06-09_10h58m36s_Exposure_4.0sec.tif]

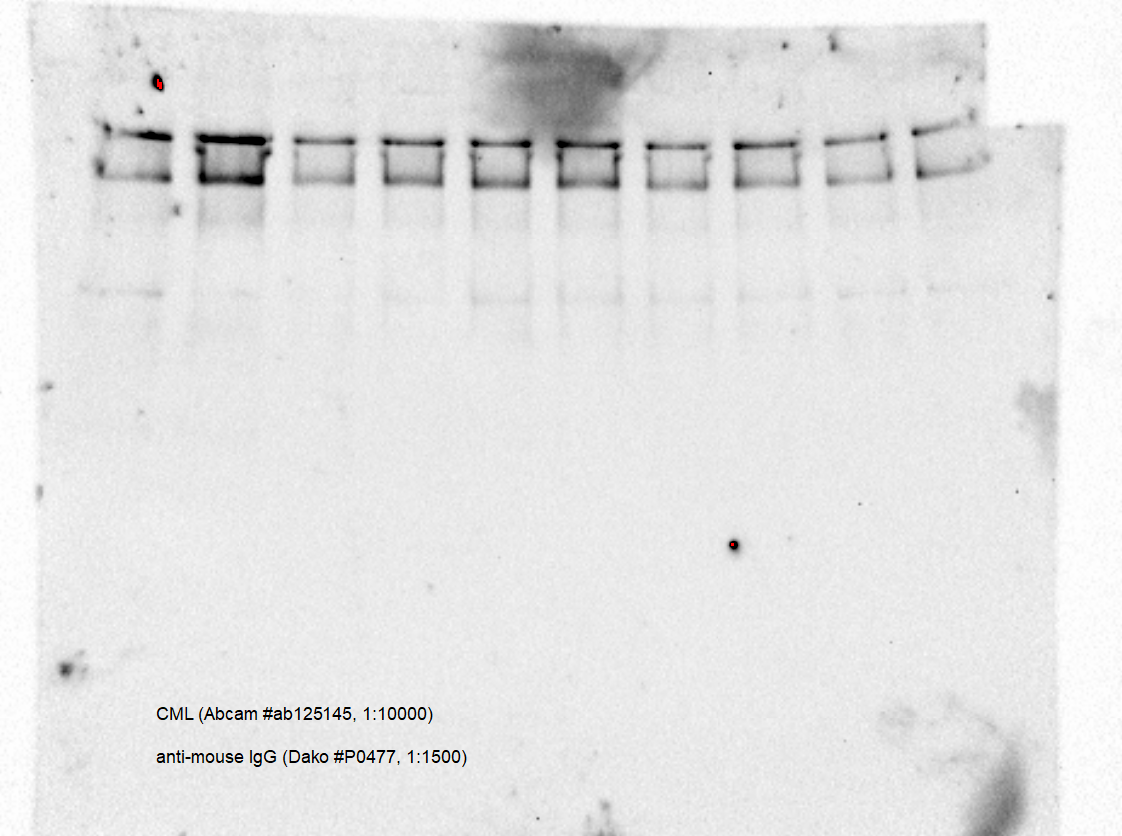

Supplement: Supplementary file 11 — Source Data [file 41467_2021_26982_MOESM11_ESM.zip › Figure 3/3H/rep1_2/CML/research_ori 2021-06-08_09h26m45s_Exposure_27.8sec.tif]

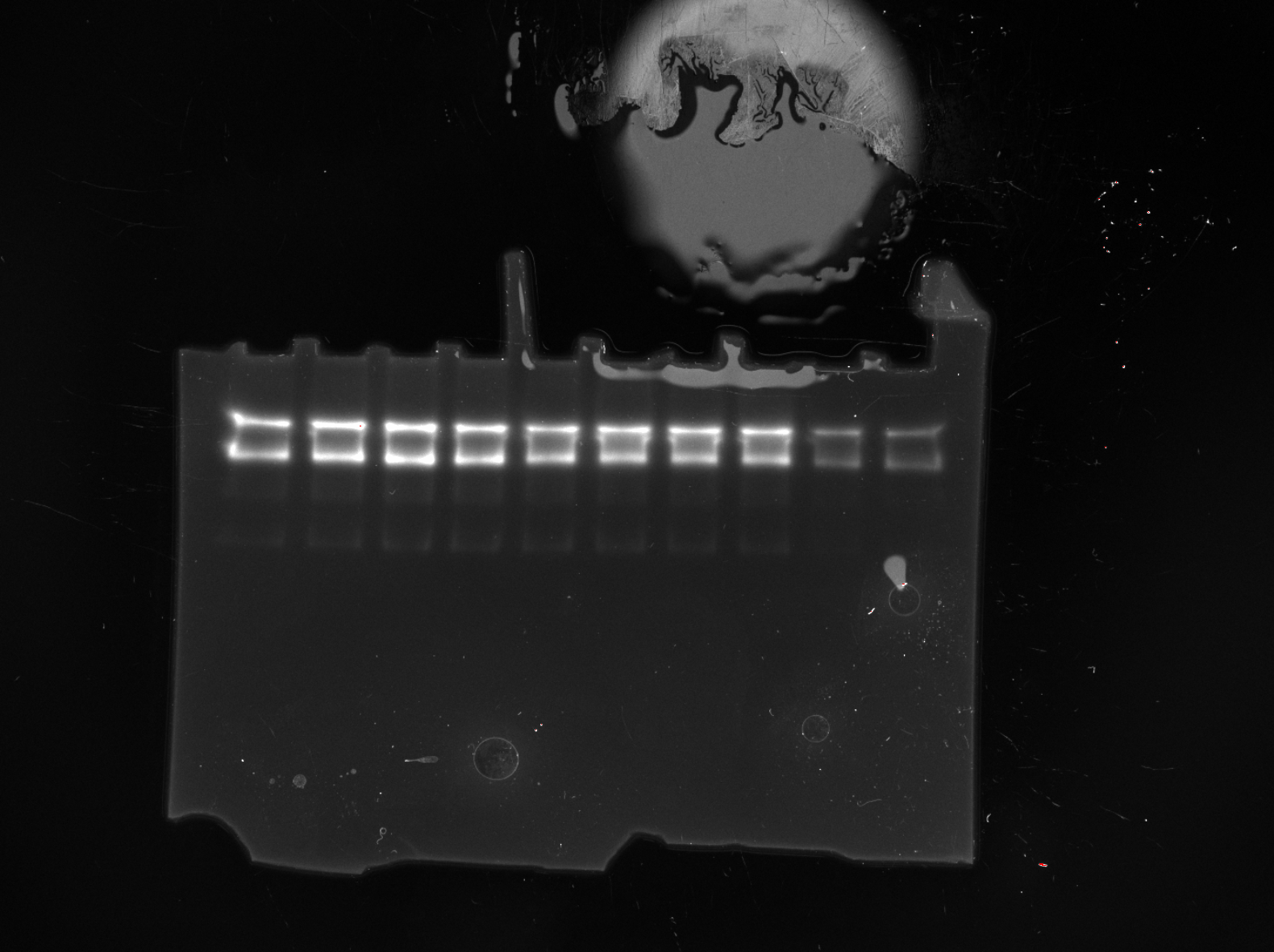

Supplement: Supplementary file 11 — Source Data [file 41467_2021_26982_MOESM11_ESM.zip › Figure 3/3H/rep3_4/Activity/research_ori 2021-06-07_16h47m11s_2sec.tif]

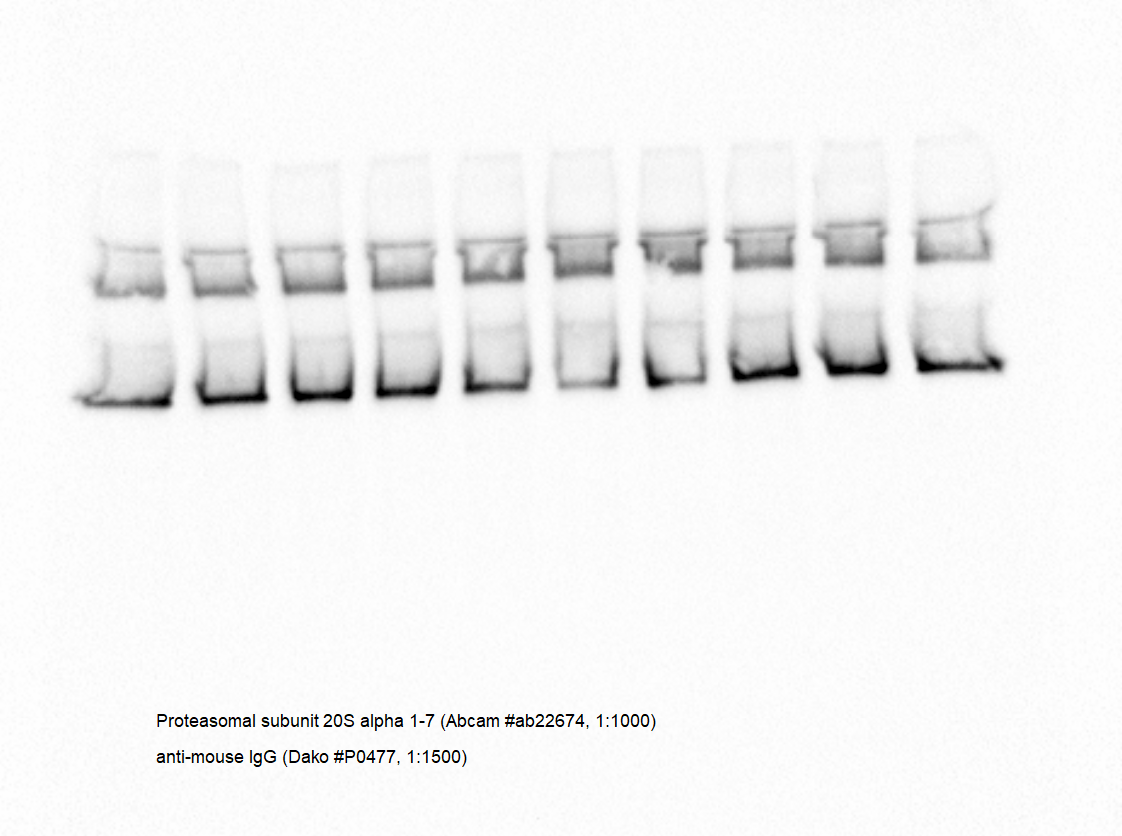

Supplement: Supplementary file 11 — Source Data [file 41467_2021_26982_MOESM11_ESM.zip › Figure 3/3H/rep3_4/Alpha1_7/research_ori 2021-06-09_10h52m12s_Exposure_4.0sec.tif]

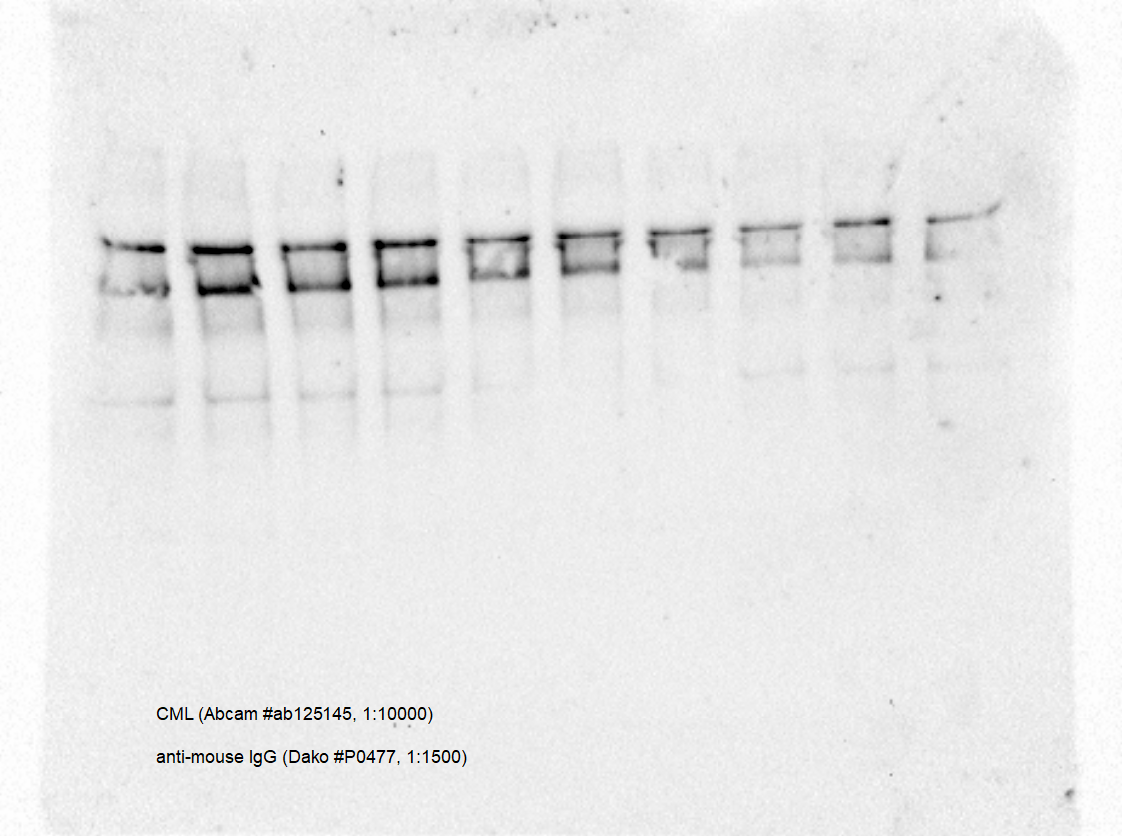

Supplement: Supplementary file 11 — Source Data [file 41467_2021_26982_MOESM11_ESM.zip › Figure 3/3H/rep3_4/CML/research_ori 2021-06-08_09h34m48s_Exposure_27.8sec.tif]

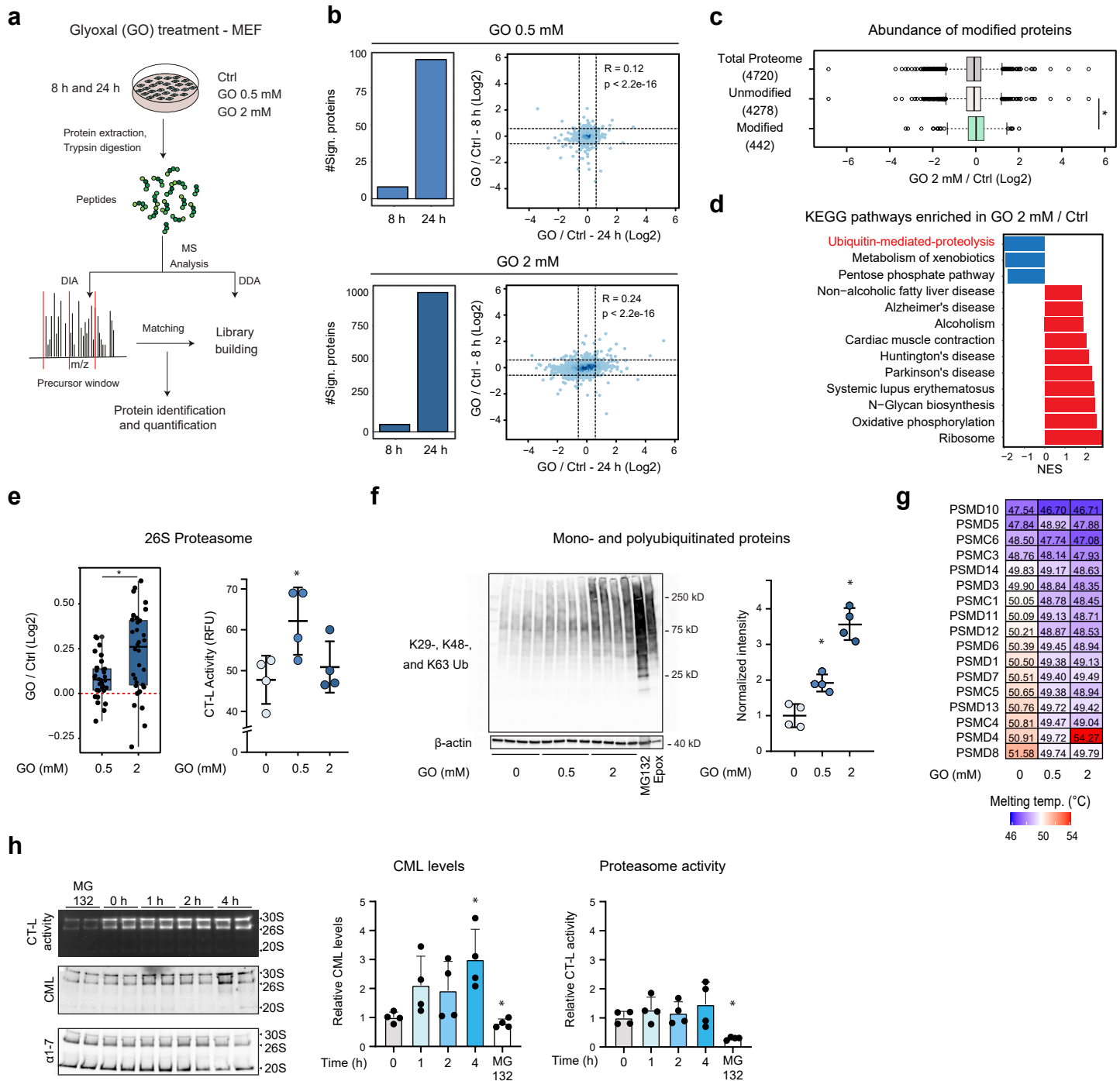

Supplement: Supplementary file 11 — Source Data [file 41467_2021_26982_MOESM11_ESM.zip › Figure 3/Figure3.pdf]

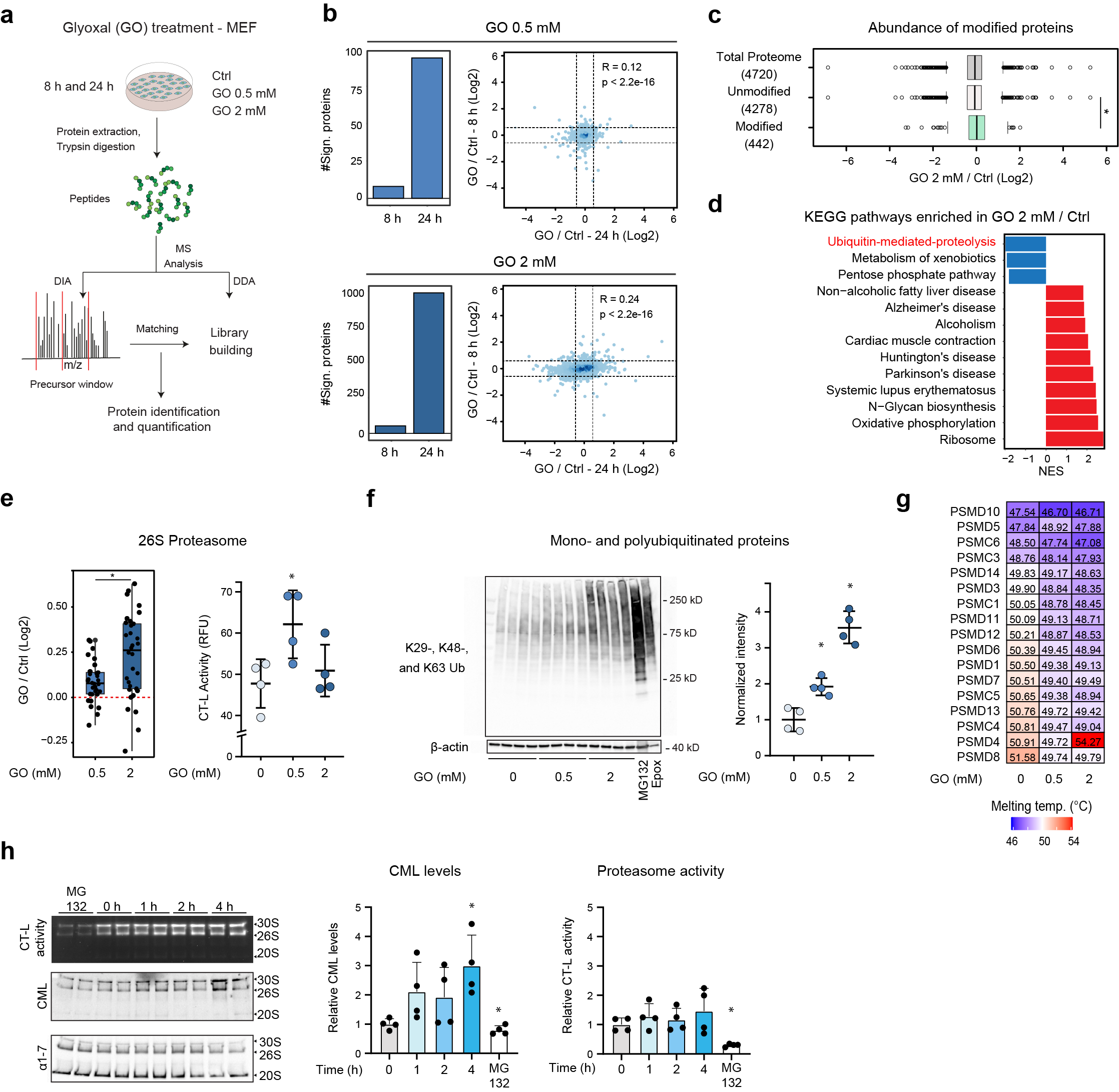

Supplement: Supplementary file 11 — Source Data [file 41467_2021_26982_MOESM11_ESM.zip › Figure 3/Figure3.png]

**Data 1**

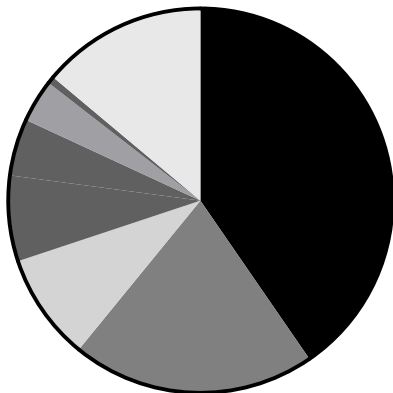

- Nucleus
- Cytoskeleton
- Mitochondrion
- ER
- Golgi
- ECM
- Others
- Plasma membrane

**Total=100**

Supplement: Supplementary file 11 — Source Data [file 41467_2021_26982_MOESM11_ESM.zip › Figure 4/4A/Piechart_166proteins.pdf]

reorder(term.description, Log10FDR, decreasing = FALSE)

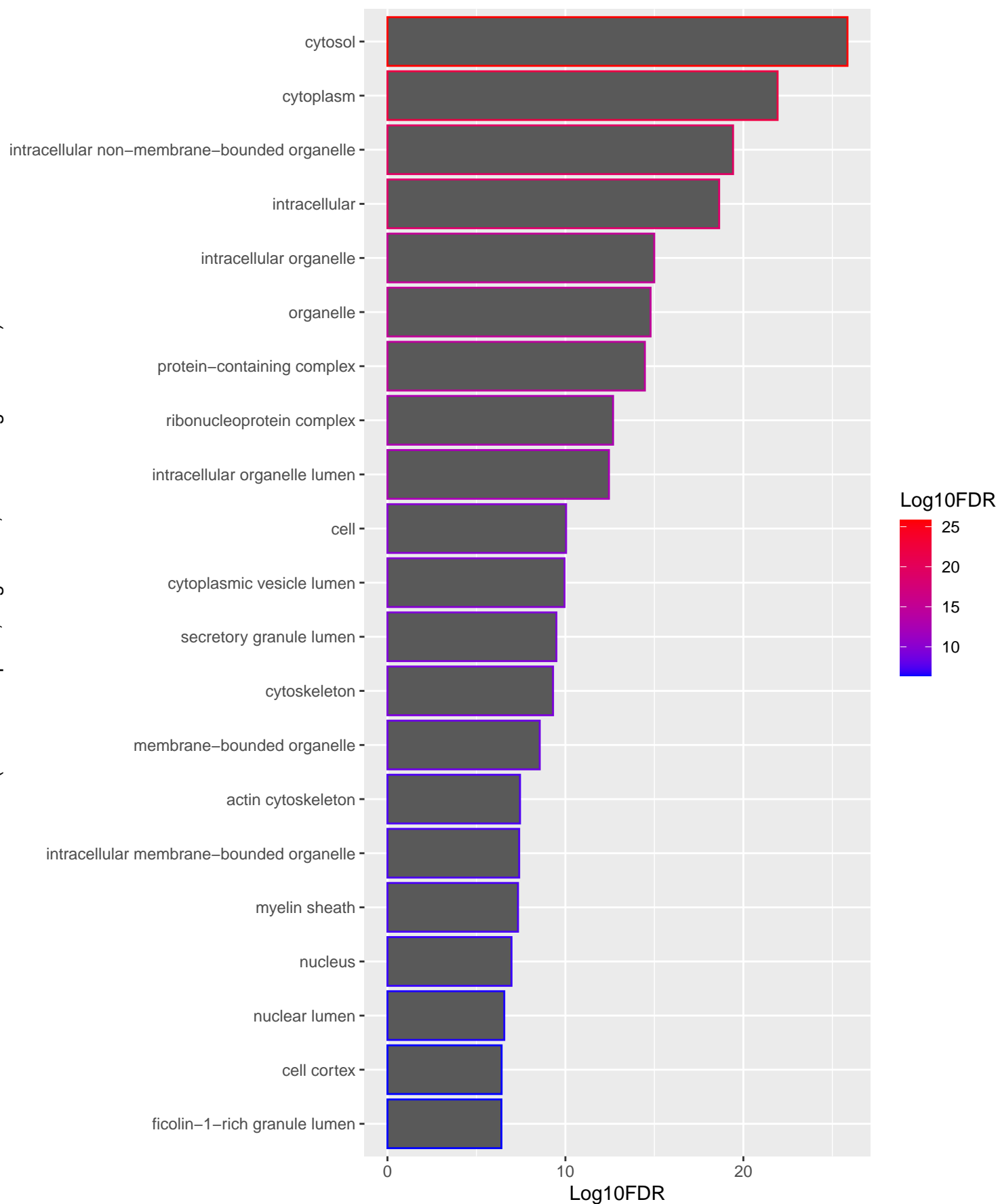

Supplement: Supplementary file 11 — Source Data [file 41467_2021_26982_MOESM11_ESM.zip › Figure 4/4A/String_Huvec.pdf]

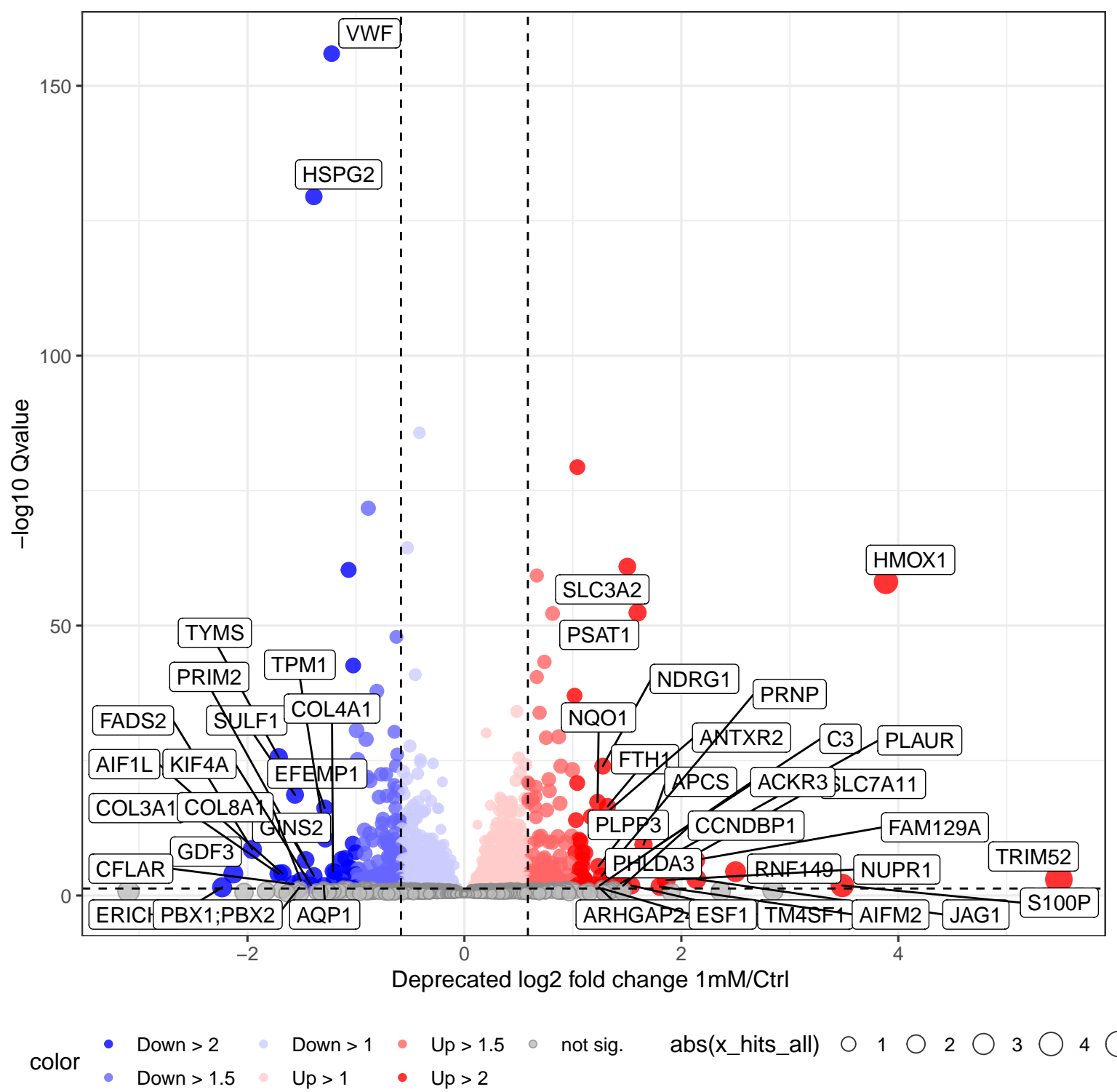

Supplement: Supplementary file 11 — Source Data [file 41467_2021_26982_MOESM11_ESM.zip › Figure 4/4B/Figure4_B_upper.pdf]

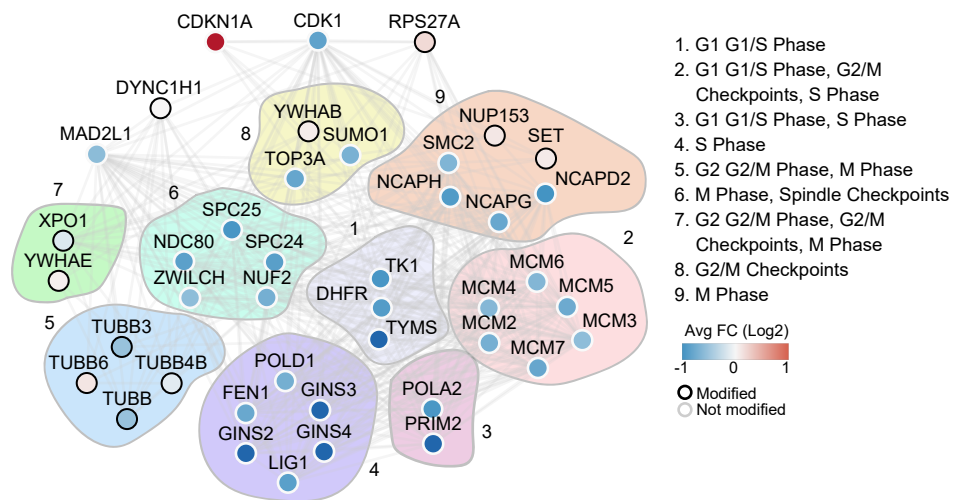

Supplement: Supplementary file 11 — Source Data [file 41467_2021_26982_MOESM11_ESM.zip › Figure 5/Fig 5C.pdf]

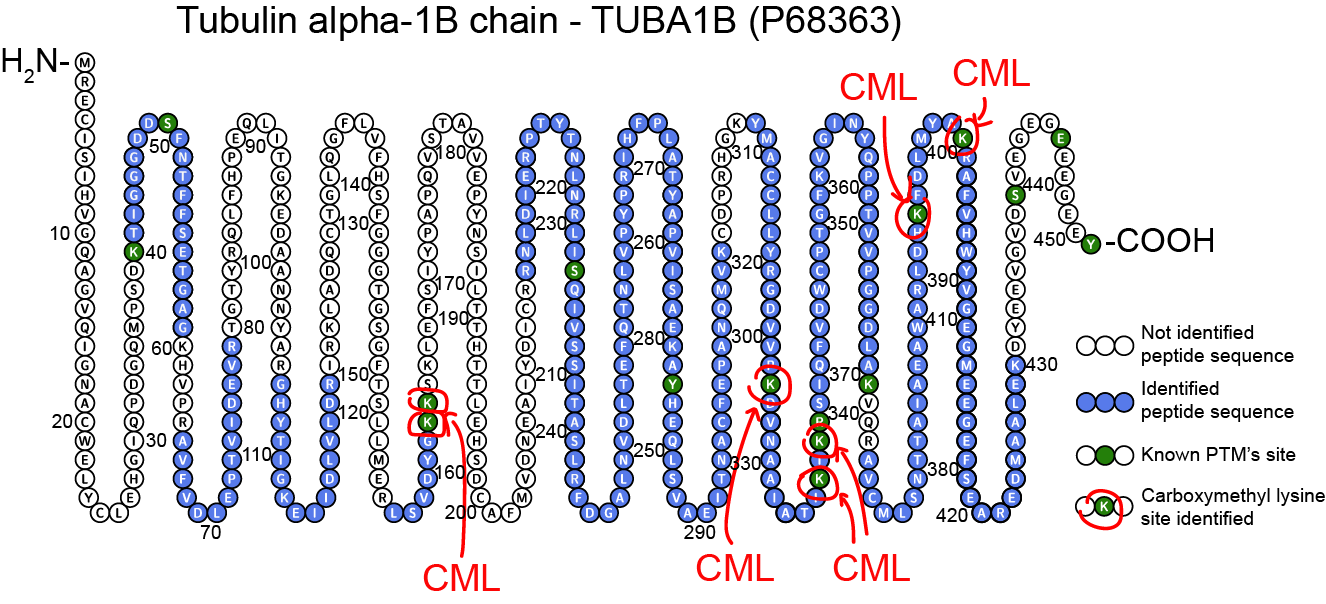

Supplement: Supplementary file 11 — Source Data [file 41467_2021_26982_MOESM11_ESM.zip › Figure 6/6A/Figure6.png]

Tubulin alpha-1B chain - TUBA1B (P68363)

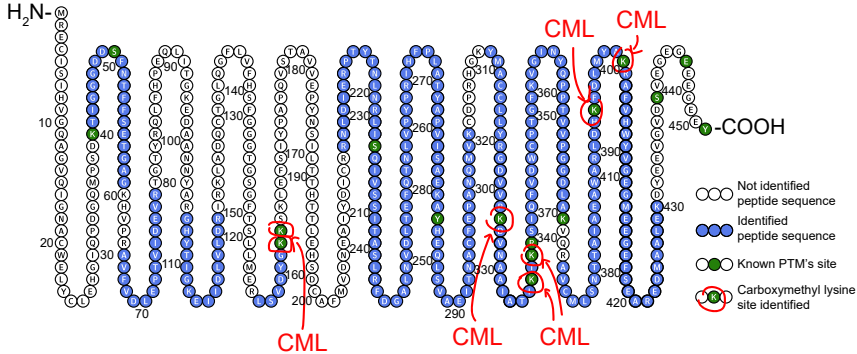

Supplement: Supplementary file 11 — Source Data [file 41467_2021_26982_MOESM11_ESM.zip › Figure 6/6A/Figure6A.pdf]

Ratio

fmol Cml Tubb4b/fmol Tubb4b  
(K58)

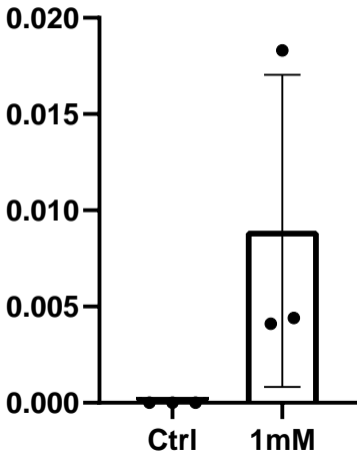

Supplement: Supplementary file 11 — Source Data [file 41467_2021_26982_MOESM11_ESM.zip › Figure 6/6G/210719_Quantification.pdf]

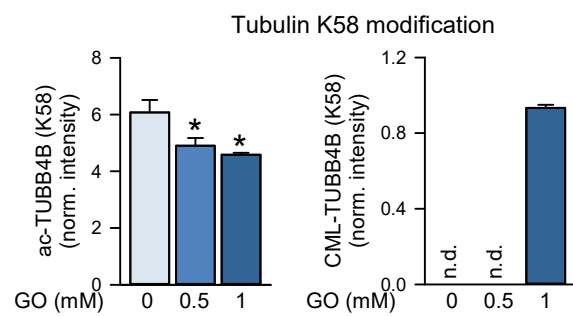

Supplement: Supplementary file 11 — Source Data [file 41467_2021_26982_MOESM11_ESM.zip › Figure 6/6H/Figure6_G.pdf]

Tubulin alpha-1B chain - TUBA1B (P68363)

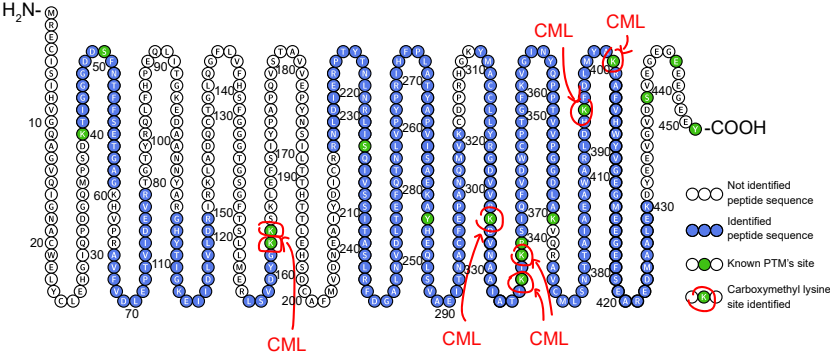

Supplement: Supplementary file 11 — Source Data [file 41467_2021_26982_MOESM11_ESM.zip › Figure 6/Fig 6A.pdf]

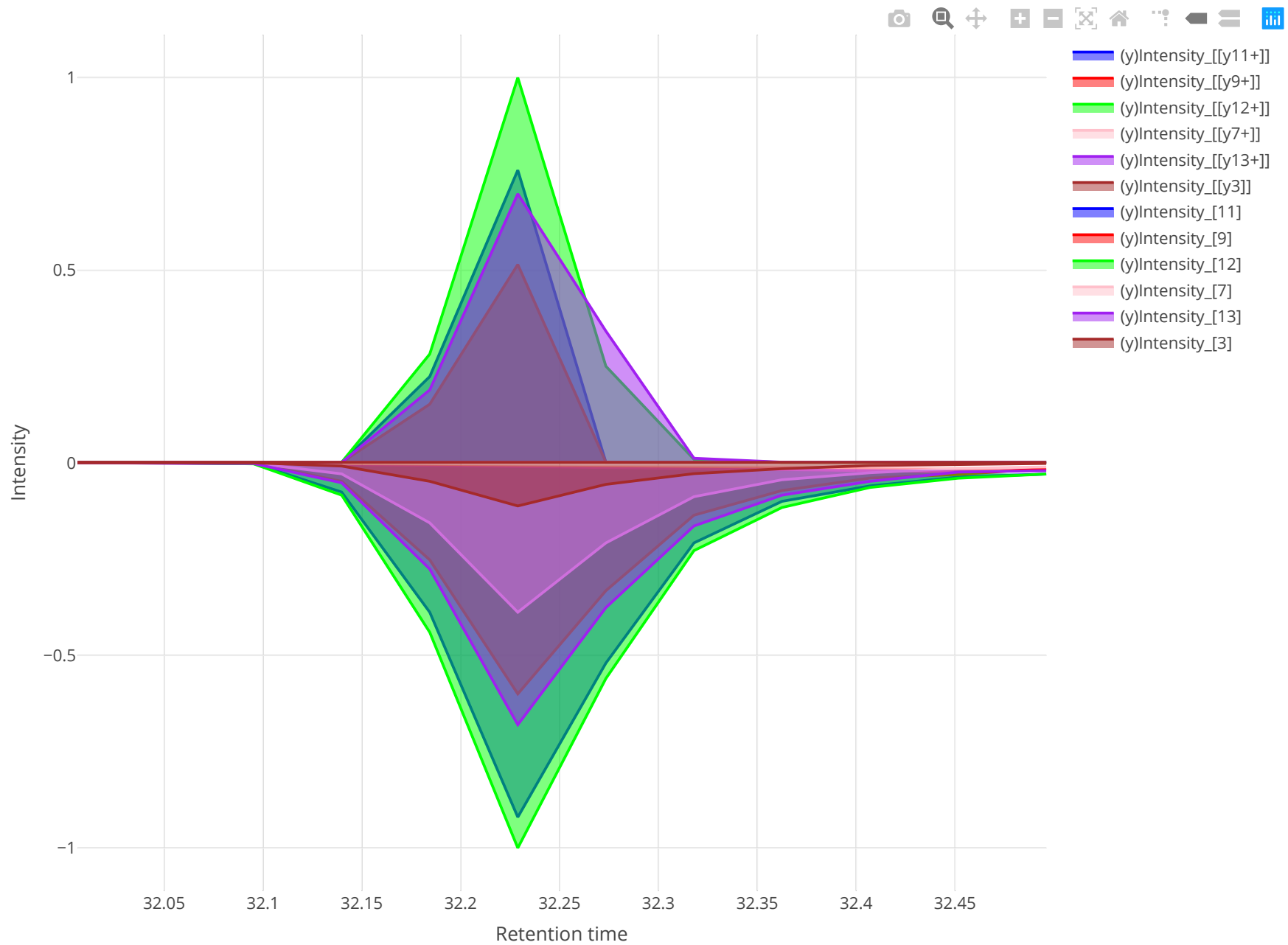

Supplement: Supplementary file 11 — Source Data [file 41467_2021_26982_MOESM11_ESM.zip › Figure 7/7G/100uM TBB4B_MOUSE._INVYYNEATGGK[CML]YVPR_.2.html.pdf]

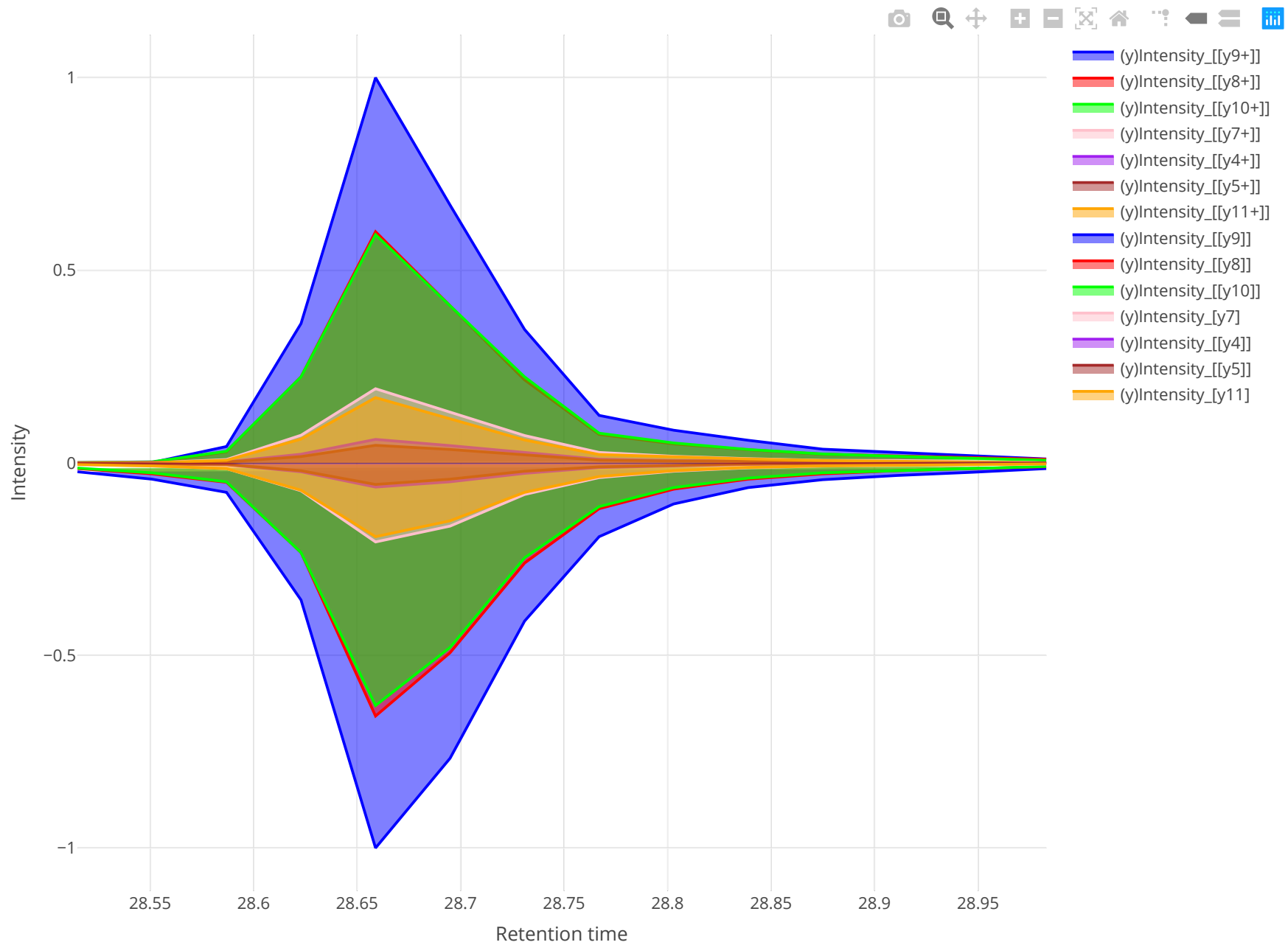

Supplement: Supplementary file 11 — Source Data [file 41467_2021_26982_MOESM11_ESM.zip › Figure 7/7G/100uM_1 TUBB4B_INVYYNEATGGK_.2.html.pdf]

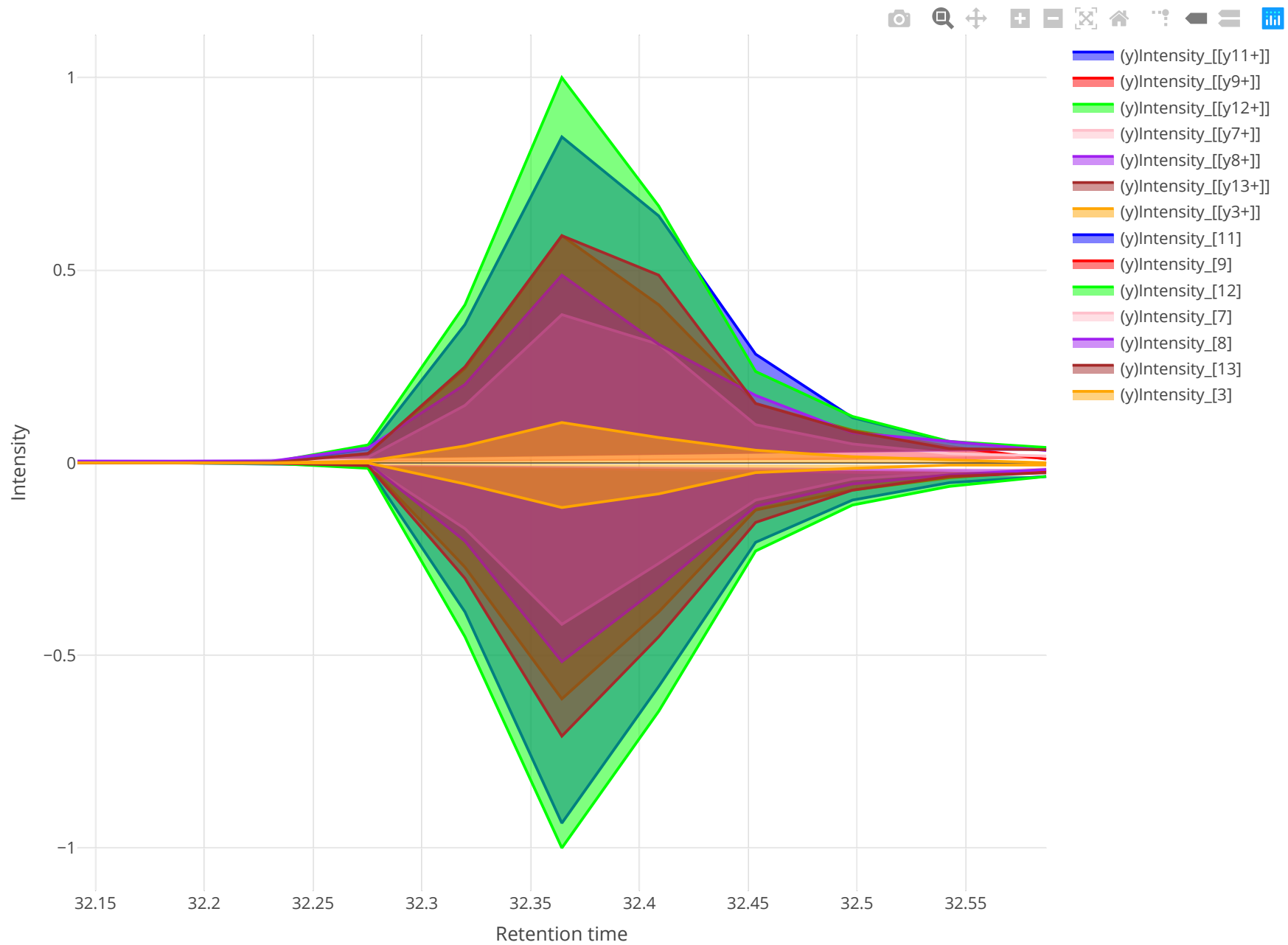

Supplement: Supplementary file 11 — Source Data [file 41467_2021_26982_MOESM11_ESM.zip › Figure 7/7G/1mM TBB4B_MOUSE._INVYYNEATGGK[CML]YVPR_.2.html.pdf]

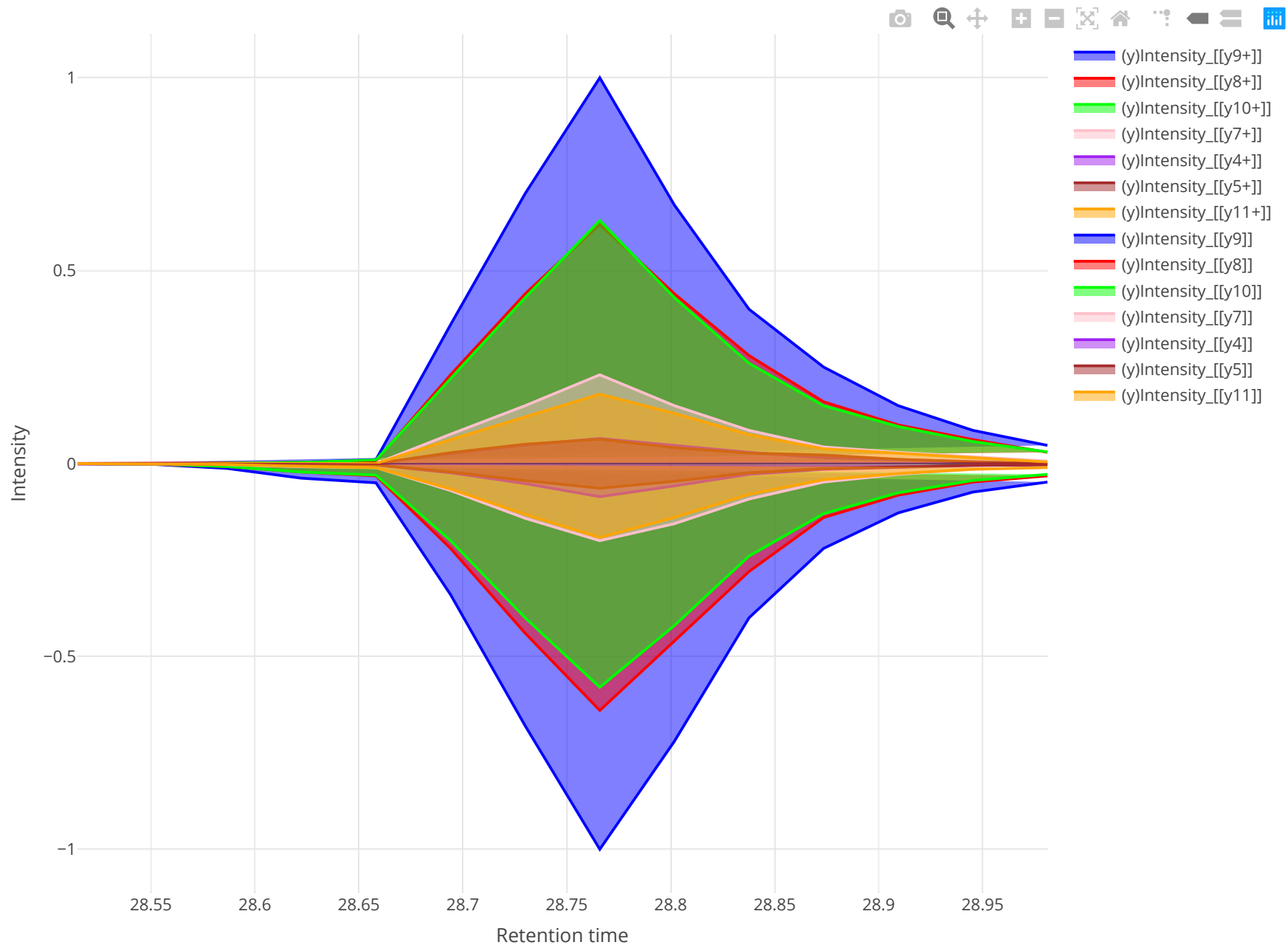

Supplement: Supplementary file 11 — Source Data [file 41467_2021_26982_MOESM11_ESM.zip › Figure 7/7G/1mM TBB4B_MOUSE._INVYYNEATGGK_.2.html.pdf]

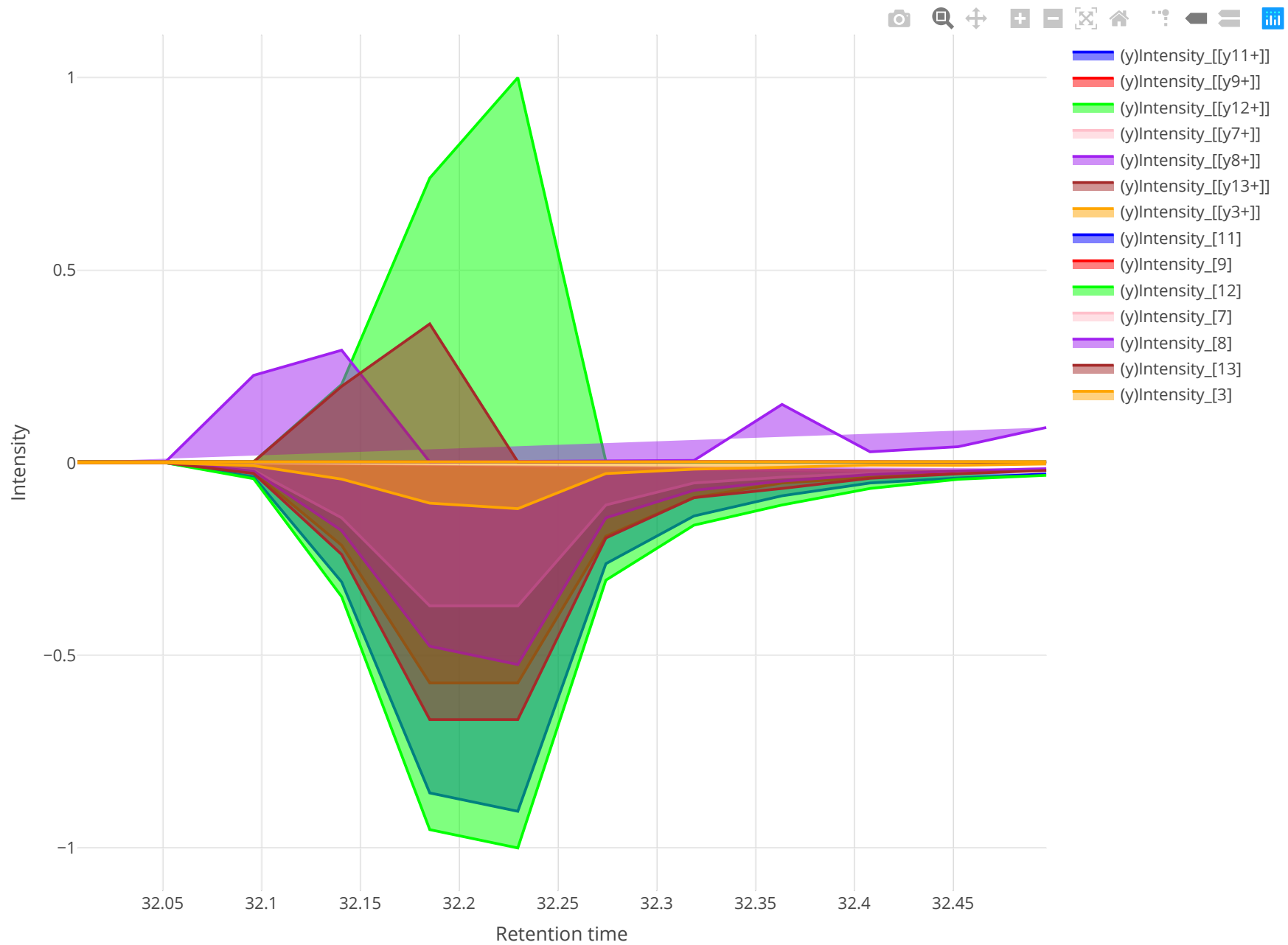

Supplement: Supplementary file 11 — Source Data [file 41467_2021_26982_MOESM11_ESM.zip › Figure 7/7G/Ctrl_2 INVYYNEATGGK[CML]YVPR_.2.html.pdf]

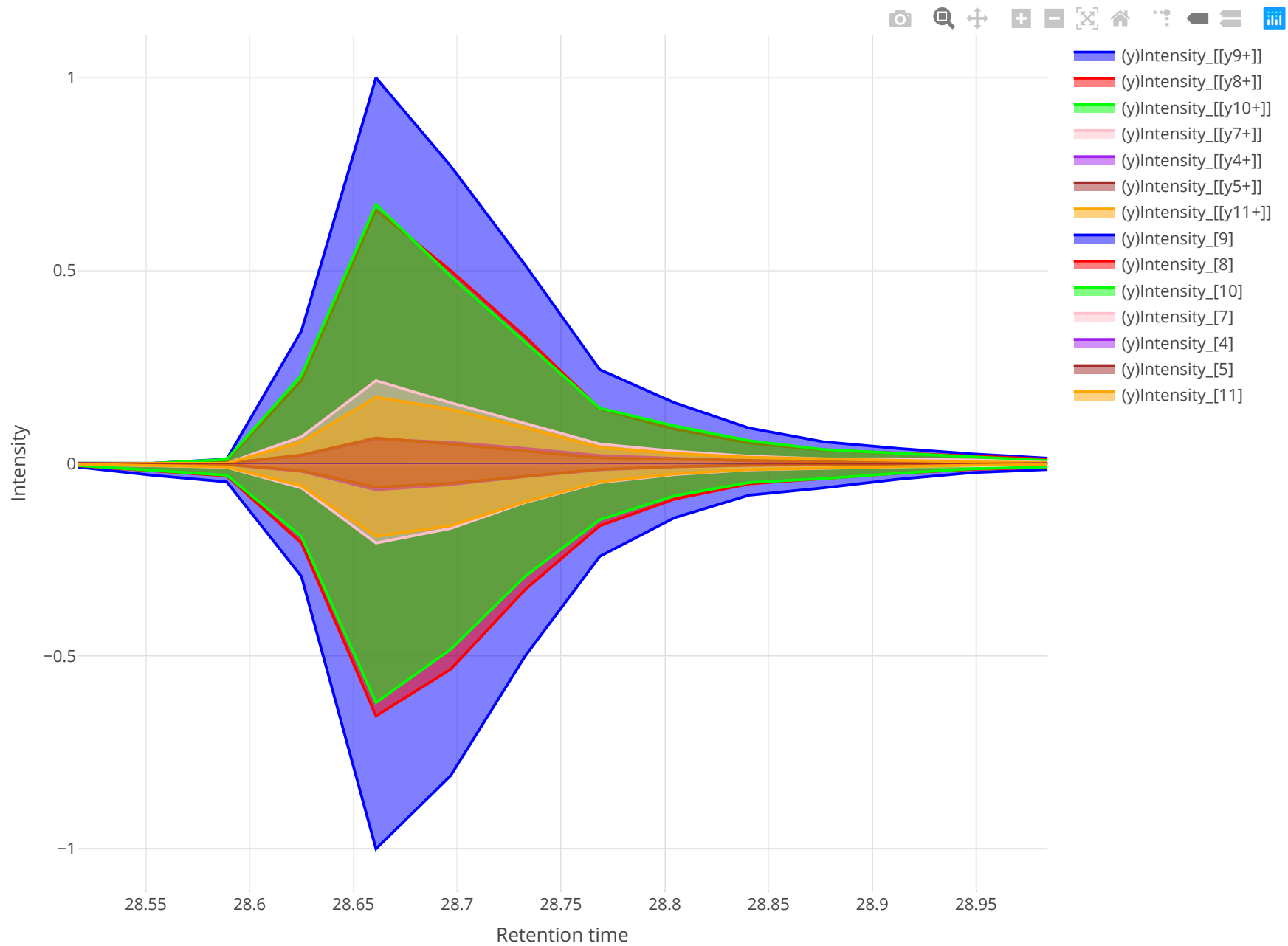

Supplement: Supplementary file 11 — Source Data [file 41467_2021_26982_MOESM11_ESM.zip › Figure 7/7G/Ctrl_2_INVYYNEATGGK_.2.html.pdf]

Ratio

fmol Cml Tubb4b/fmol Tubb4b

(K58)

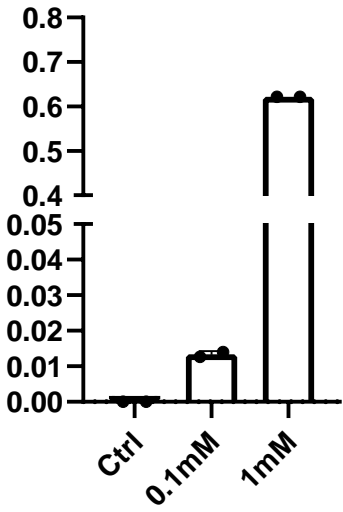

Supplement: Supplementary file 11 — Source Data [file 41467_2021_26982_MOESM11_ESM.zip › Figure 7/7H/Cml_Tubb4bK58_Spectrodive (ratio).pdf]

# Parallel reaction monitoring

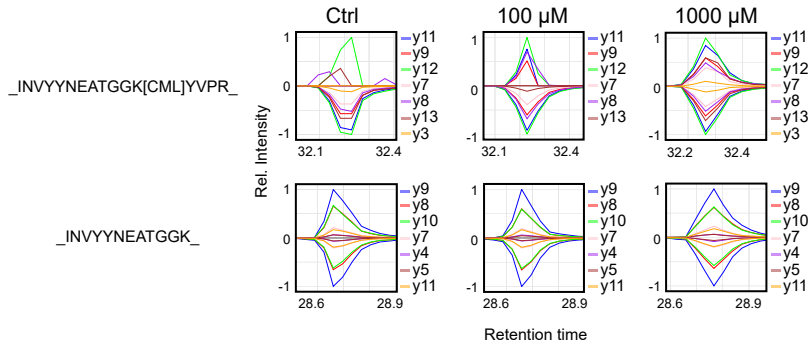

# Quantification

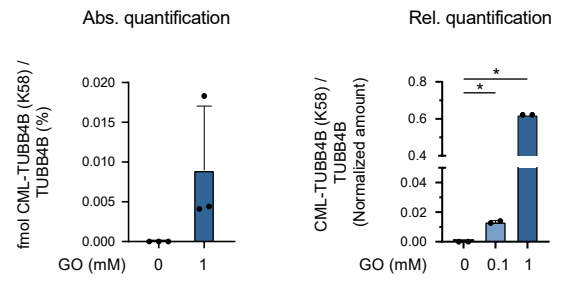

Supplement: Supplementary file 11 — Source Data [file 41467_2021_26982_MOESM11_ESM.zip › Figure 7/Fig 7G,H, 6G.pdf]

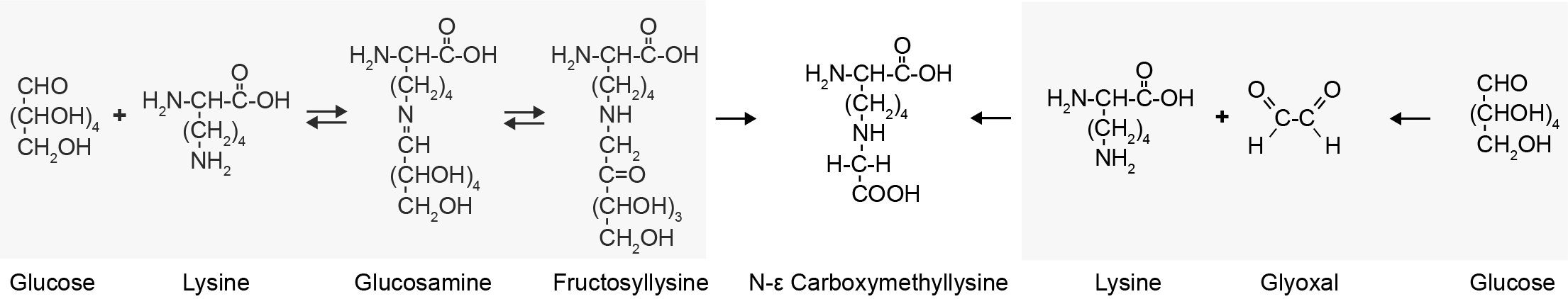

Supplement: Supplementary file 11 — Source Data [file 41467_2021_26982_MOESM11_ESM.zip › FigureS1/1A/CML_Reaction.png]

Intersection CM sites

200  
150  
100  
50  
0

200

92

5

4

3

2

1

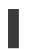

Ctrl

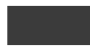

GO0.5mM

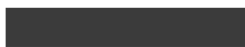

GO1mM

300  
200  
100  
0

CM sites

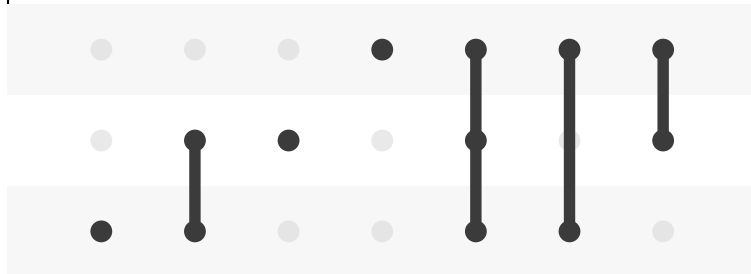

Supplement: Supplementary file 11 — Source Data [file 41467_2021_26982_MOESM11_ESM.zip › FigureS1/1C/UpSetPlot_CM_overlap_ID_w_combi_Huvec.pdf]

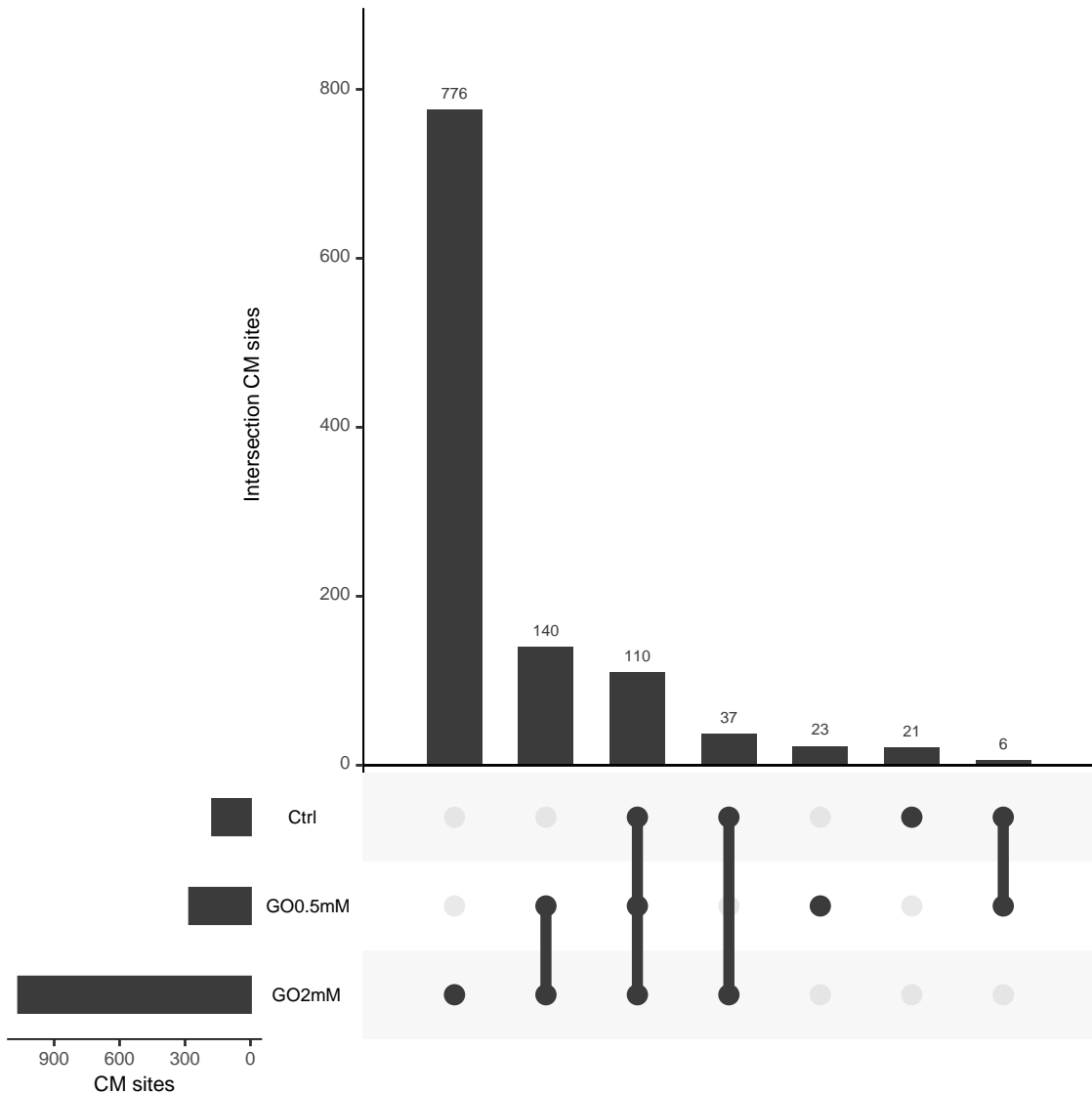

Supplement: Supplementary file 11 — Source Data [file 41467_2021_26982_MOESM11_ESM.zip › FigureS1/1C/UpSetPlot_CM_overlap_ID_w_combi_Mef.pdf]

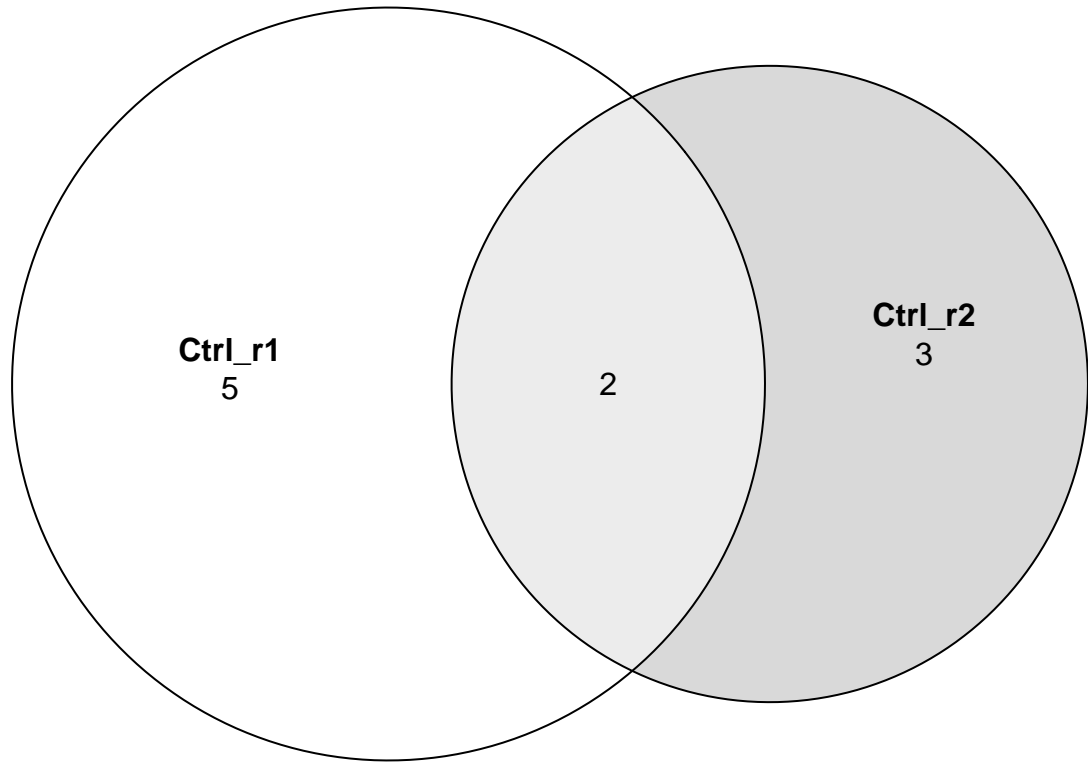

Supplement: Supplementary file 11 — Source Data [file 41467_2021_26982_MOESM11_ESM.zip › FigureS1/1D/Huvec/210610_VD_CM_elutions_Ctrl.pdf]

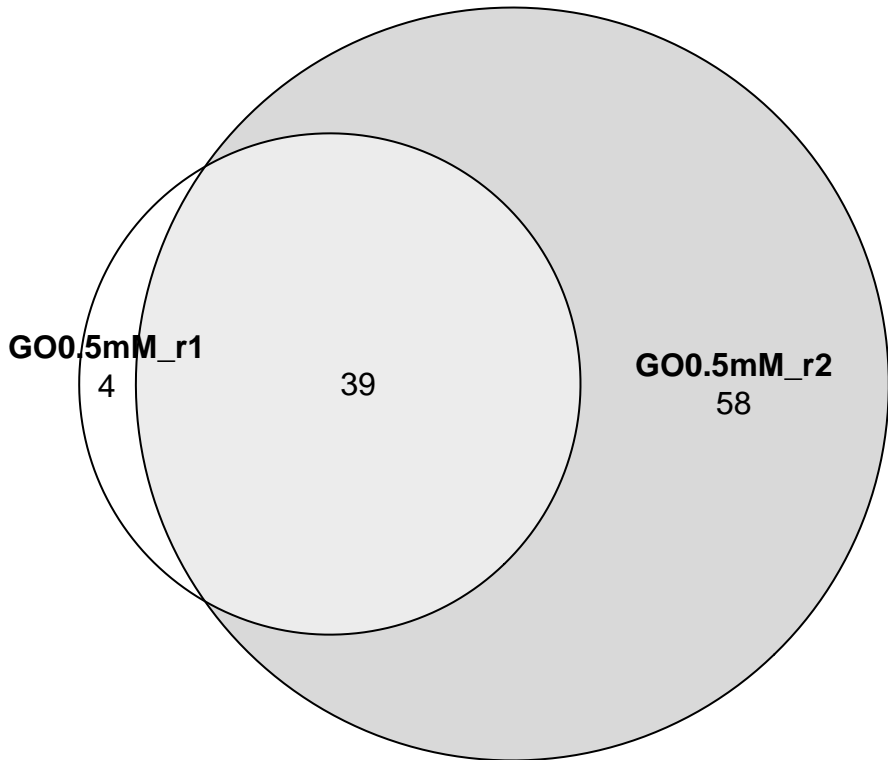

Supplement: Supplementary file 11 — Source Data [file 41467_2021_26982_MOESM11_ESM.zip › FigureS1/1D/Huvec/210610_VD_CM_elutions_GO0.5mM.pdf]

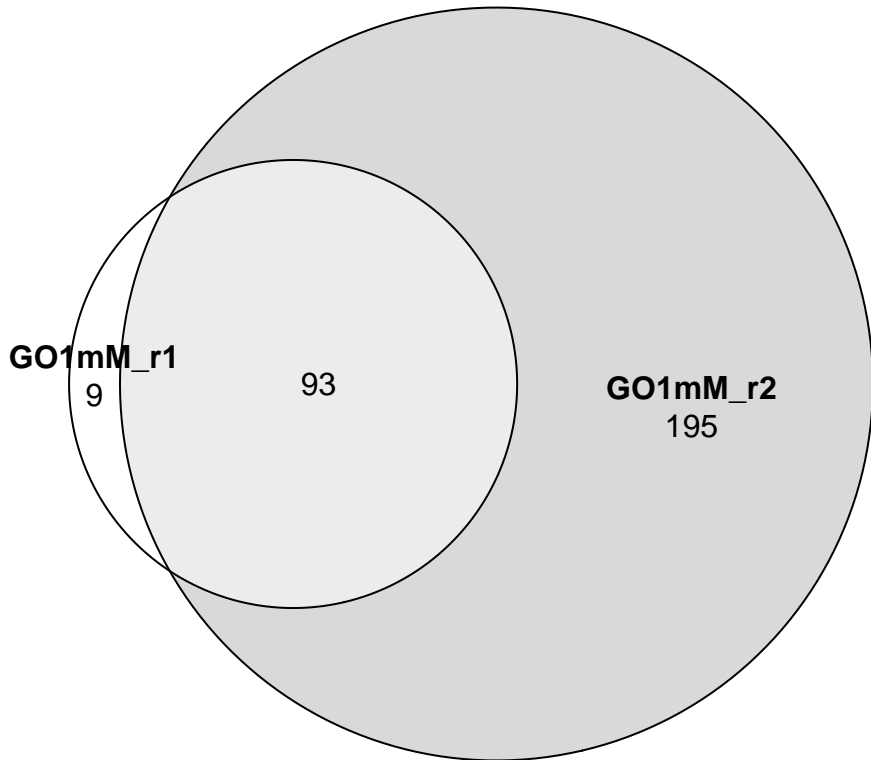

Supplement: Supplementary file 11 — Source Data [file 41467_2021_26982_MOESM11_ESM.zip › FigureS1/1D/Huvec/210610_VD_CM_elutions_GO1mM.pdf]

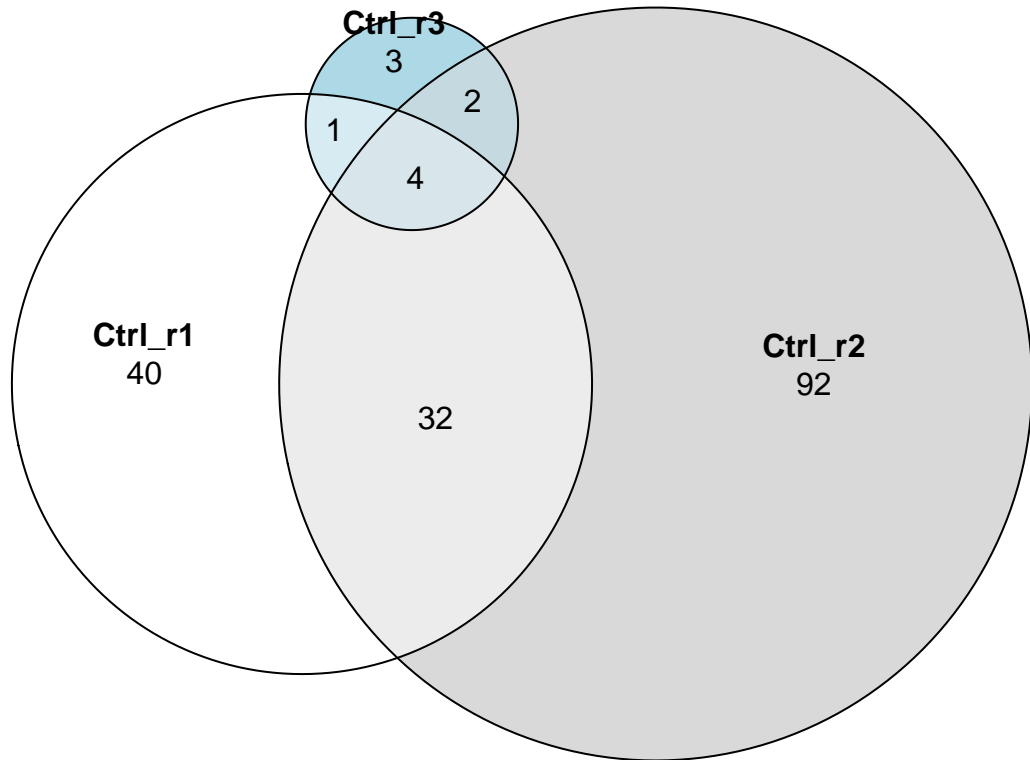

Supplement: Supplementary file 11 — Source Data [file 41467_2021_26982_MOESM11_ESM.zip › FigureS1/1D/Mef/210610_VD_CM_elutions_Ctrl.pdf]

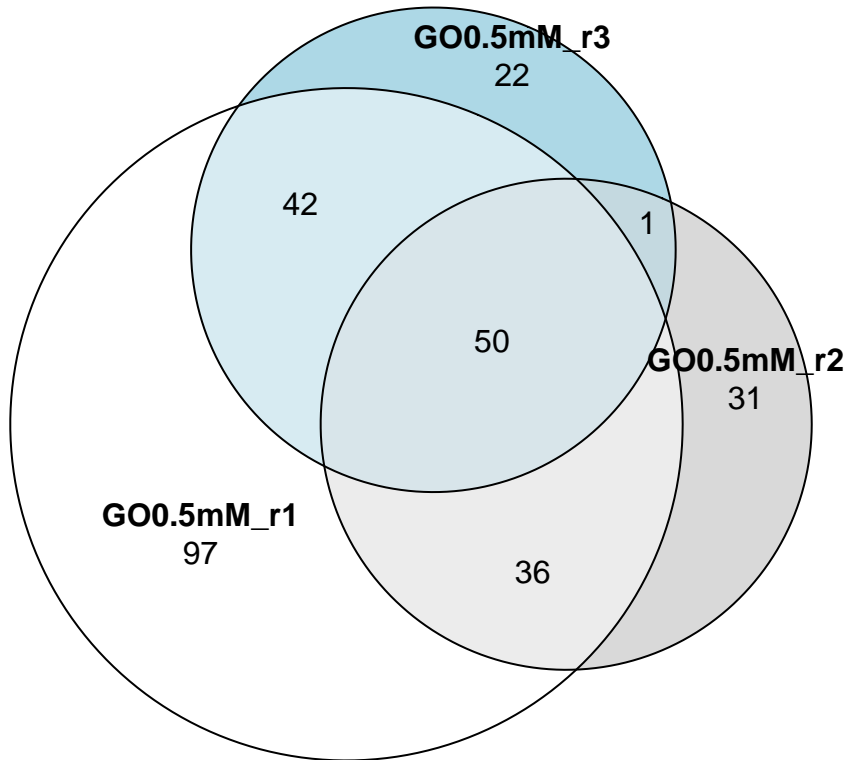

Supplement: Supplementary file 11 — Source Data [file 41467_2021_26982_MOESM11_ESM.zip › FigureS1/1D/Mef/210610_VD_CM_elutions_GO0.5mM.pdf]

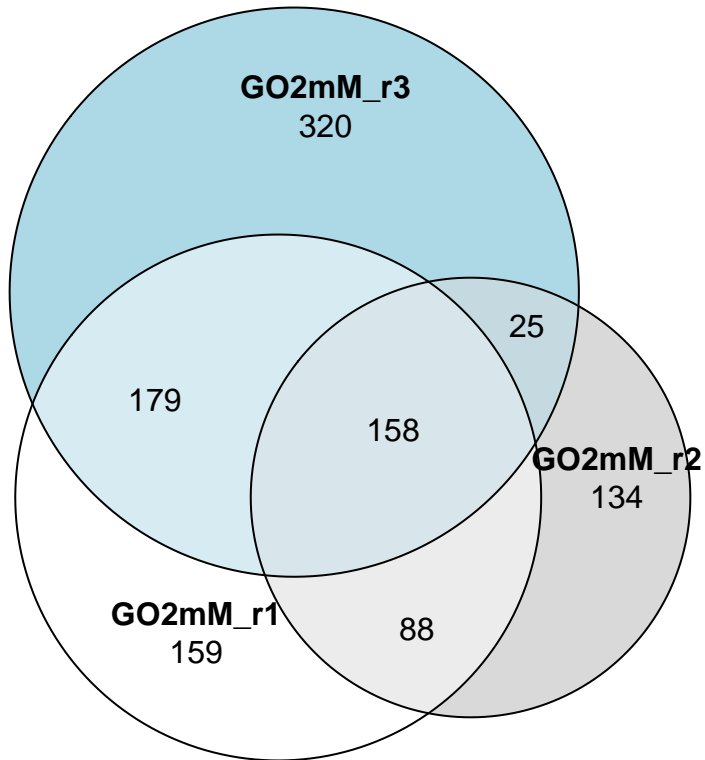

Supplement: Supplementary file 11 — Source Data [file 41467_2021_26982_MOESM11_ESM.zip › FigureS1/1D/Mef/210610_VD_CM_elutions_GO2mM.pdf]

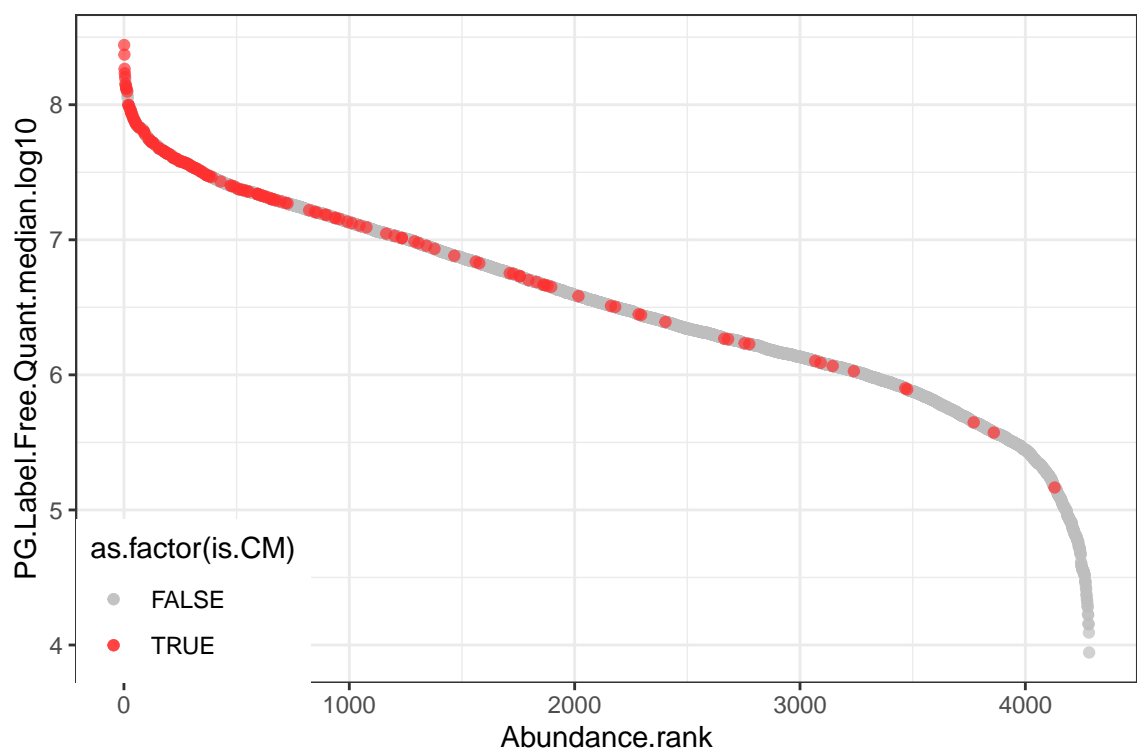

Supplement: Supplementary file 11 — Source Data [file 41467_2021_26982_MOESM11_ESM.zip › FigureS1/1E/Huvec/210919_rank_plot_Cm_modified_proteins.pdf]

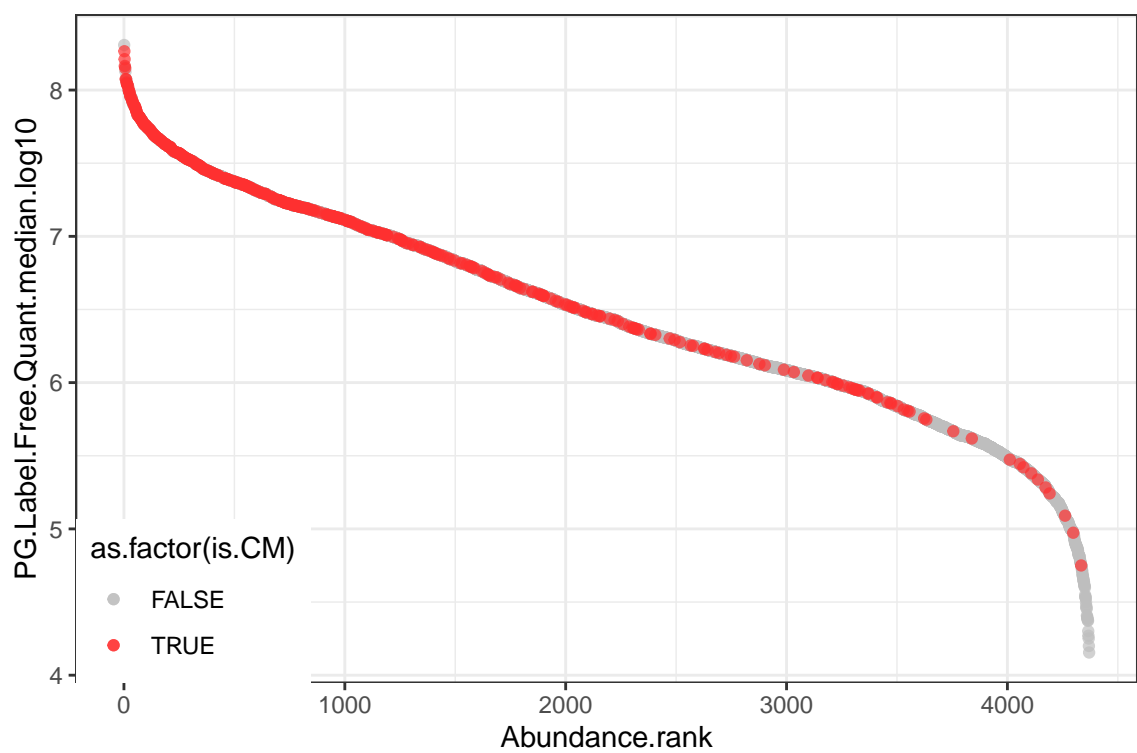

Supplement: Supplementary file 11 — Source Data [file 41467_2021_26982_MOESM11_ESM.zip › FigureS1/1E/Mef/210919_rank_plot_Cm_modified_proteins.pdf]

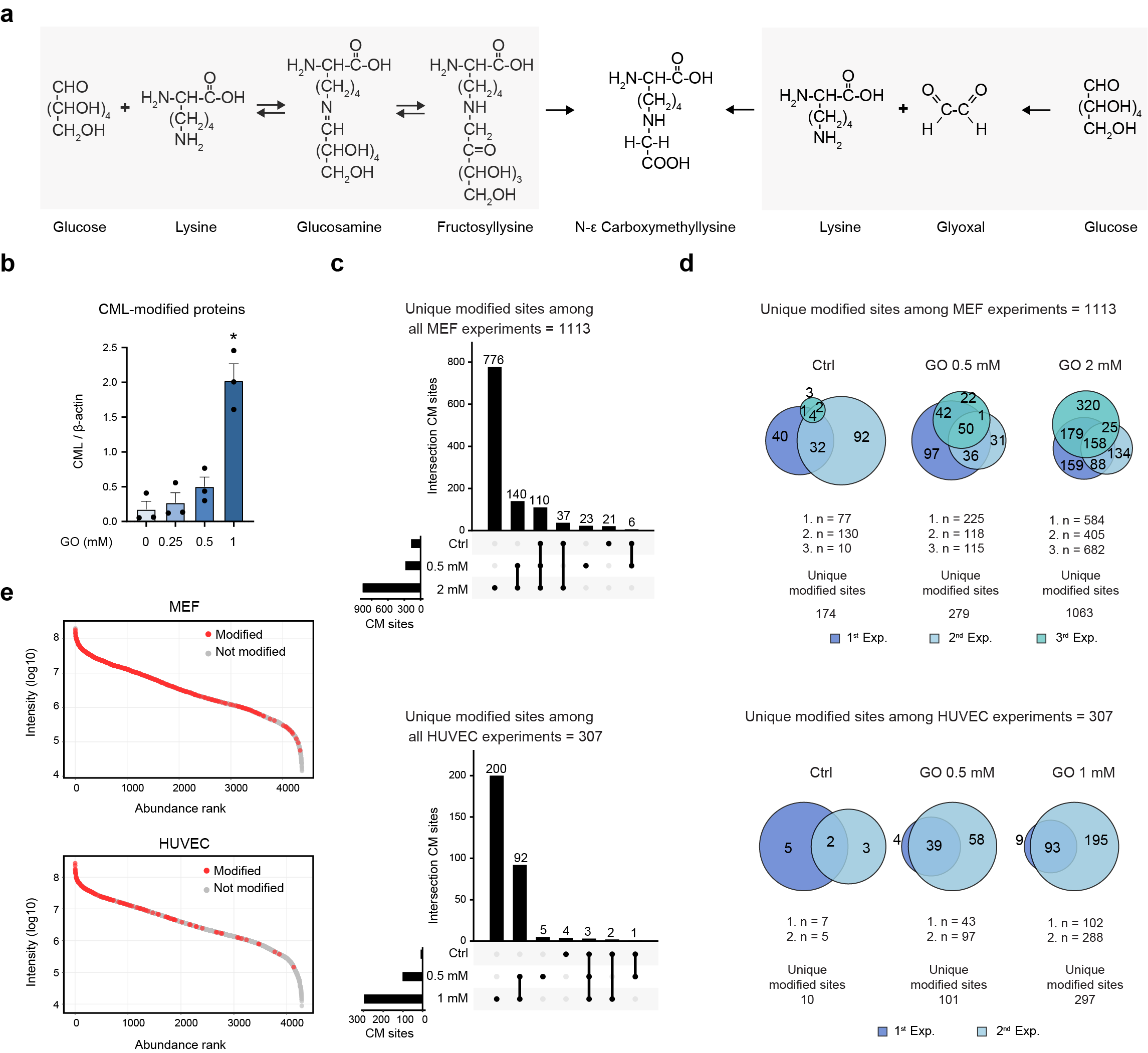

Supplement: Supplementary file 11 — Source Data [file 41467_2021_26982_MOESM11_ESM.zip › FigureS1/FigureS1.png]

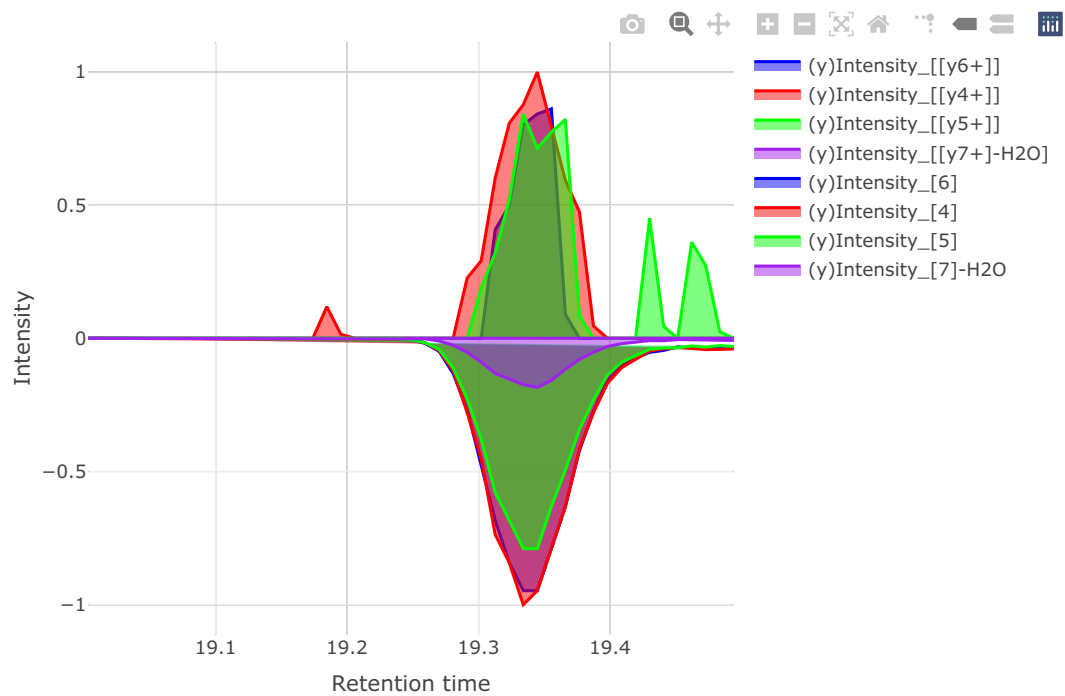

Supplement: Supplementary file 11 — Source Data [file 41467_2021_26982_MOESM11_ESM.zip › FigureS2/2A/ACSL1_MOUSE._IFGQANTSLK[CML]R_.3.pdf]

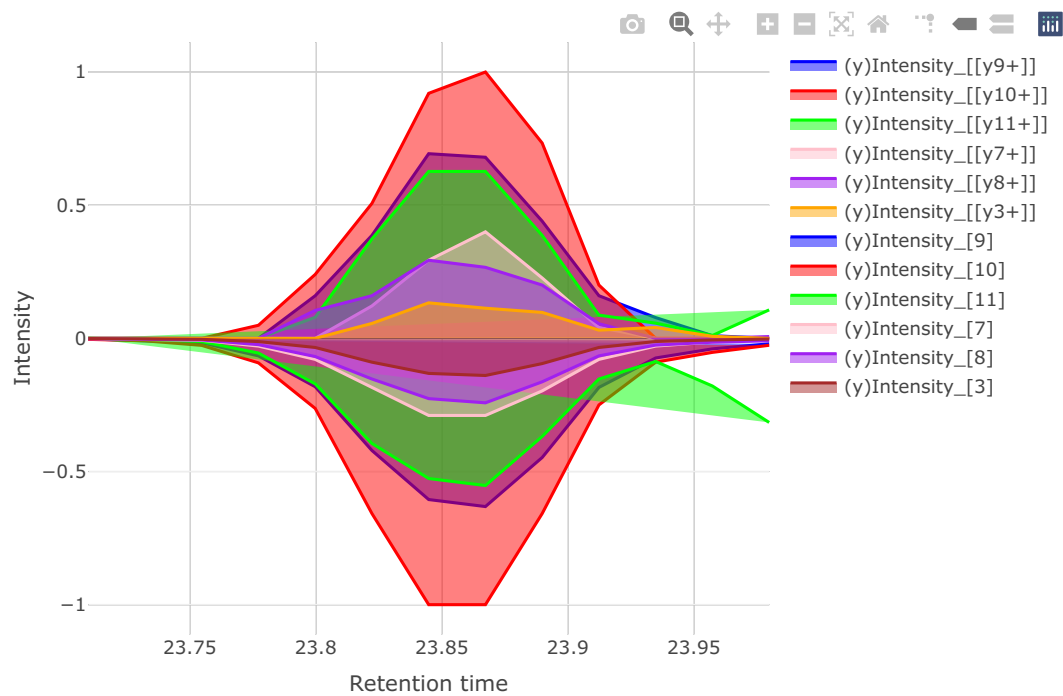

Supplement: Supplementary file 11 — Source Data [file 41467_2021_26982_MOESM11_ESM.zip › FigureS2/2A/ADT1_MOUSE._IAK[CML]DEGANAFFK_.2.pdf]

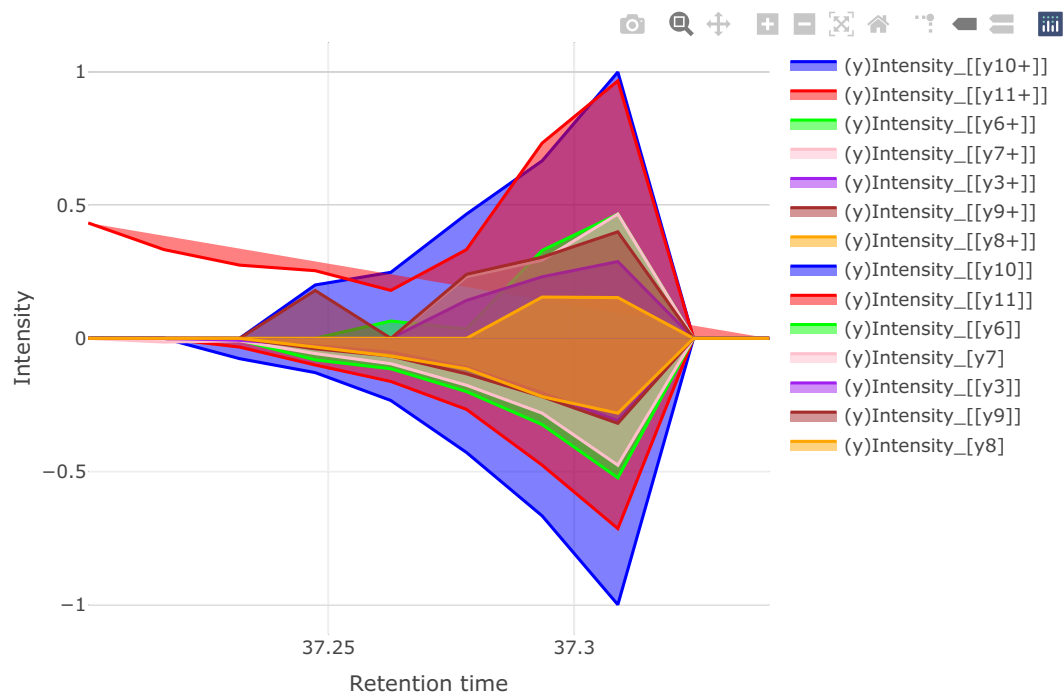

Supplement: Supplementary file 11 — Source Data [file 41467_2021_26982_MOESM11_ESM.zip › FigureS2/2A/ANXA5_MOUSE._DLVDDLK[CML]SELTGK_.2.pdf]

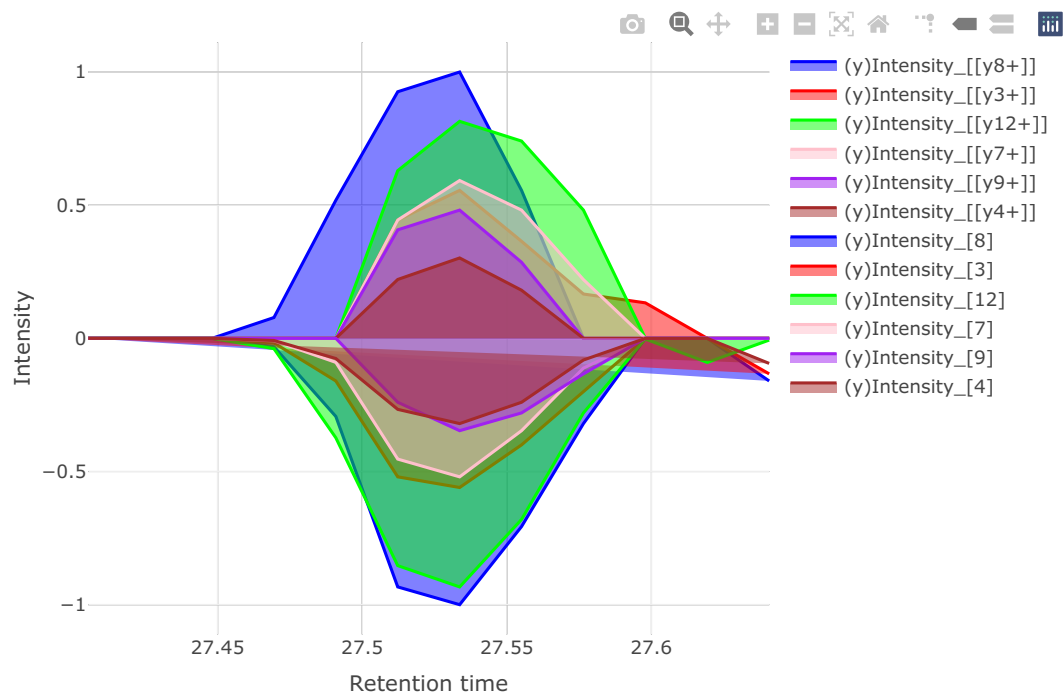

Supplement: Supplementary file 11 — Source Data [file 41467_2021_26982_MOESM11_ESM.zip › FigureS2/2A/AT5F1_MOUSE._QIQDAIDMEK[CML]AQQALVQK_.3.pdf]

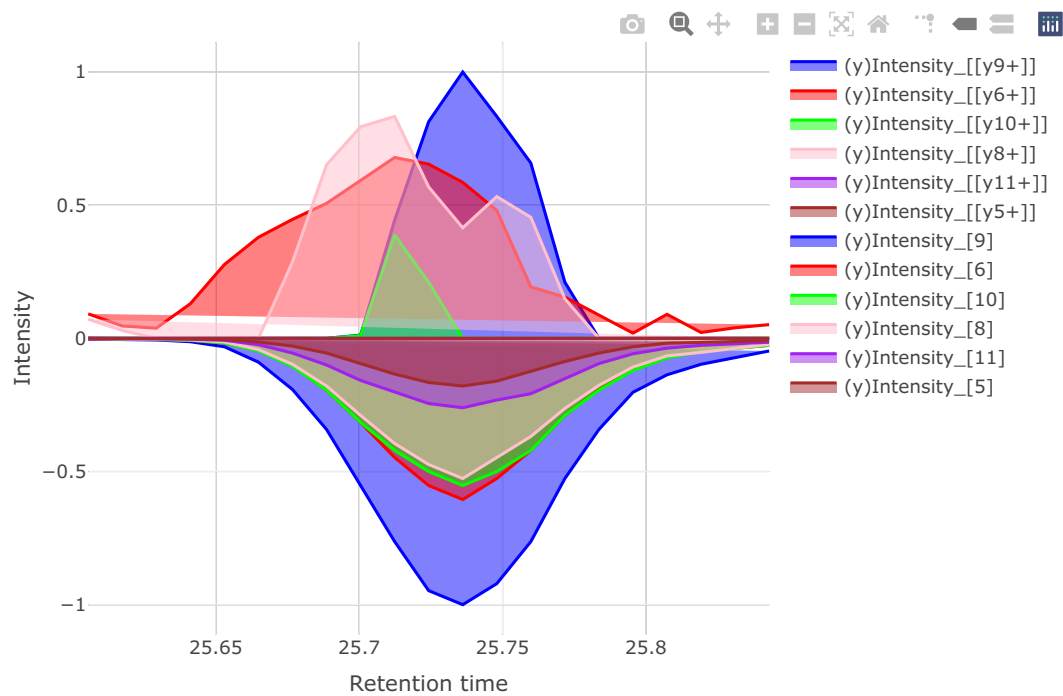

Supplement: Supplementary file 11 — Source Data [file 41467_2021_26982_MOESM11_ESM.zip › FigureS2/2A/ATPA_MOUSE._VVDALGNAIDGK[CML]GPIGSK_.3.pdf]

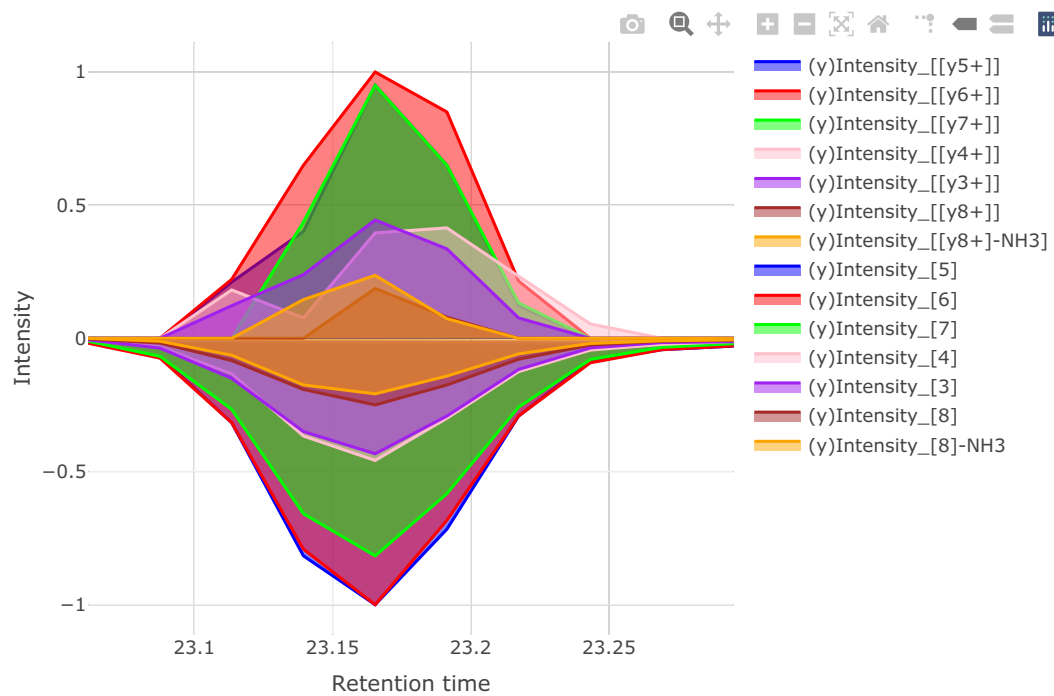

Supplement: Supplementary file 11 — Source Data [file 41467_2021_26982_MOESM11_ESM.zip › FigureS2/2A/ATPB_MOUSE._GVQK[CML]ILQDYK_.2.pdf]
