# Supplementary material for: Mapping protein carboxymethylation sites provides insights into their role in proteostasis and cell proliferation
Source: Nat Commun. 2021 Nov 18;12:6743. doi: 10.1038/s41467-021-26982-6 (PMC8602705; doi:10.1038/s41467-021-26982-6)
Supplement: Supplementary file 11 — Source Data [file 41467_2021_26982_MOESM11_ESM.zip › FigureS2/2A/NDUA8_MOUSE._PGIVELPTLEELK[CML]VEEVK_.3.pdf]

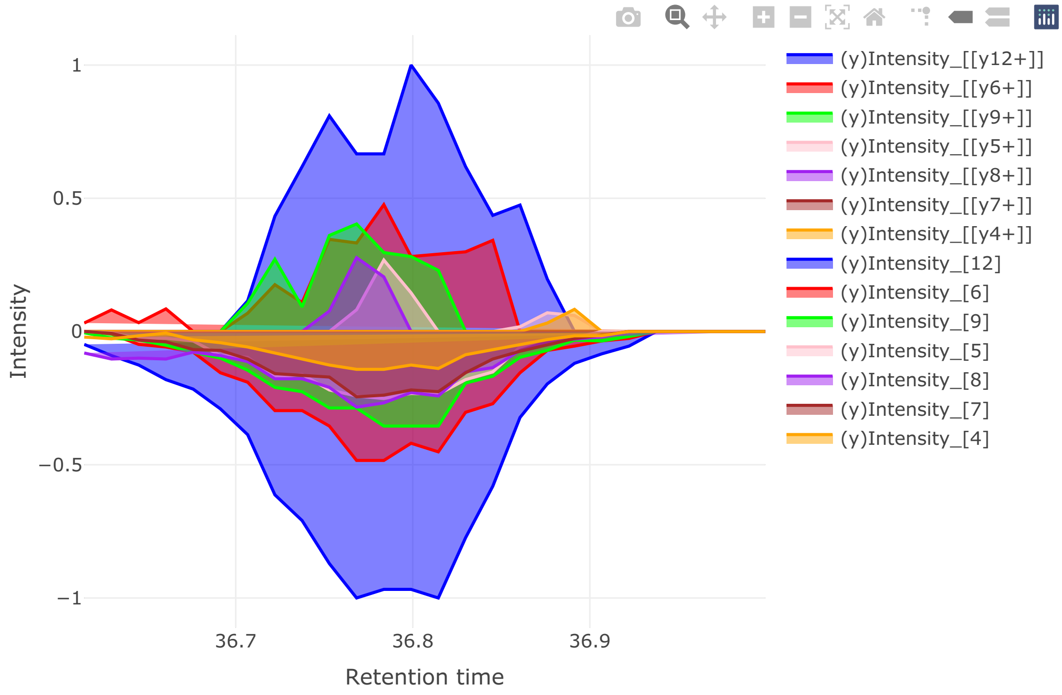

Intensity
Retention time
1
0.5
0
−0.5
−1
36.7
36.8
36.9
(y)Intensity_[[y12+]]
(y)Intensity_[[y6+]]
(y)Intensity_[[y9+]]
(y)Intensity_[[y5+]]
(y)Intensity_[[y8+]]
(y)Intensity_[[y7+]]
(y)Intensity_[[y4+]]
(y)Intensity_[12]
(y)Intensity_[6]
(y)Intensity_[9]
(y)Intensity_[5]
(y)Intensity_[8]
(y)Intensity_[7]
(y)Intensity_[4]
