# Supplementary material for: Mapping protein carboxymethylation sites provides insights into their role in proteostasis and cell proliferation
Source: Nat Commun. 2021 Nov 18;12:6743. doi: 10.1038/s41467-021-26982-6 (PMC8602705; doi:10.1038/s41467-021-26982-6)
Supplement: Supplementary file 11 — Source Data [file 41467_2021_26982_MOESM11_ESM.zip › FigureS2/2D/Overlap_tissues_table_w_cytoplasm_new_v2.pdf]

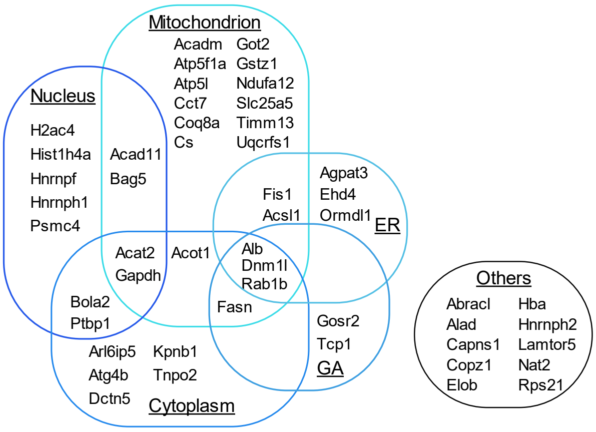
Mitochondrion
Acadm
Got2
Atp5f1a
Gstz1
Atp5l
Ndufa12
Cct7
Slc25a5
Coq8a
Timm13
Cs
Uqcrfs1
Nucleus
H2ac4
Hist1h4a
Hnrnpf
Hnrnph1
Psmc4
Acad11
Bag5
Agpat3
Ehd4
Ormdl1
ER
Fis1
Acsl1
Acat2
Gapdh
Acot1
Alb
Dnm1l
Rab1b
Fasn
Bola2
Ptbp1
Gosr2
Tcp1
GA
Arl6ip5
Kpnb1
Atg4b
Tnpo2
Dctn5
Cytoplasm
Others
Abracl
Hba
Alad
Hnrnph2
Capns1
Lamtor5
Copz1
Nat2
Elob
Rps21
