# Supplementary material for: Mapping protein carboxymethylation sites provides insights into their role in proteostasis and cell proliferation
Source: Nat Commun. 2021 Nov 18;12:6743. doi: 10.1038/s41467-021-26982-6 (PMC8602705; doi:10.1038/s41467-021-26982-6)
Supplement: Supplementary file 11 — Source Data [file 41467_2021_26982_MOESM11_ESM.zip › FigureS2/2E/210618_Scatterplot_turnover_PG.Label.Free.Quant_Heart_Cm.pdf]

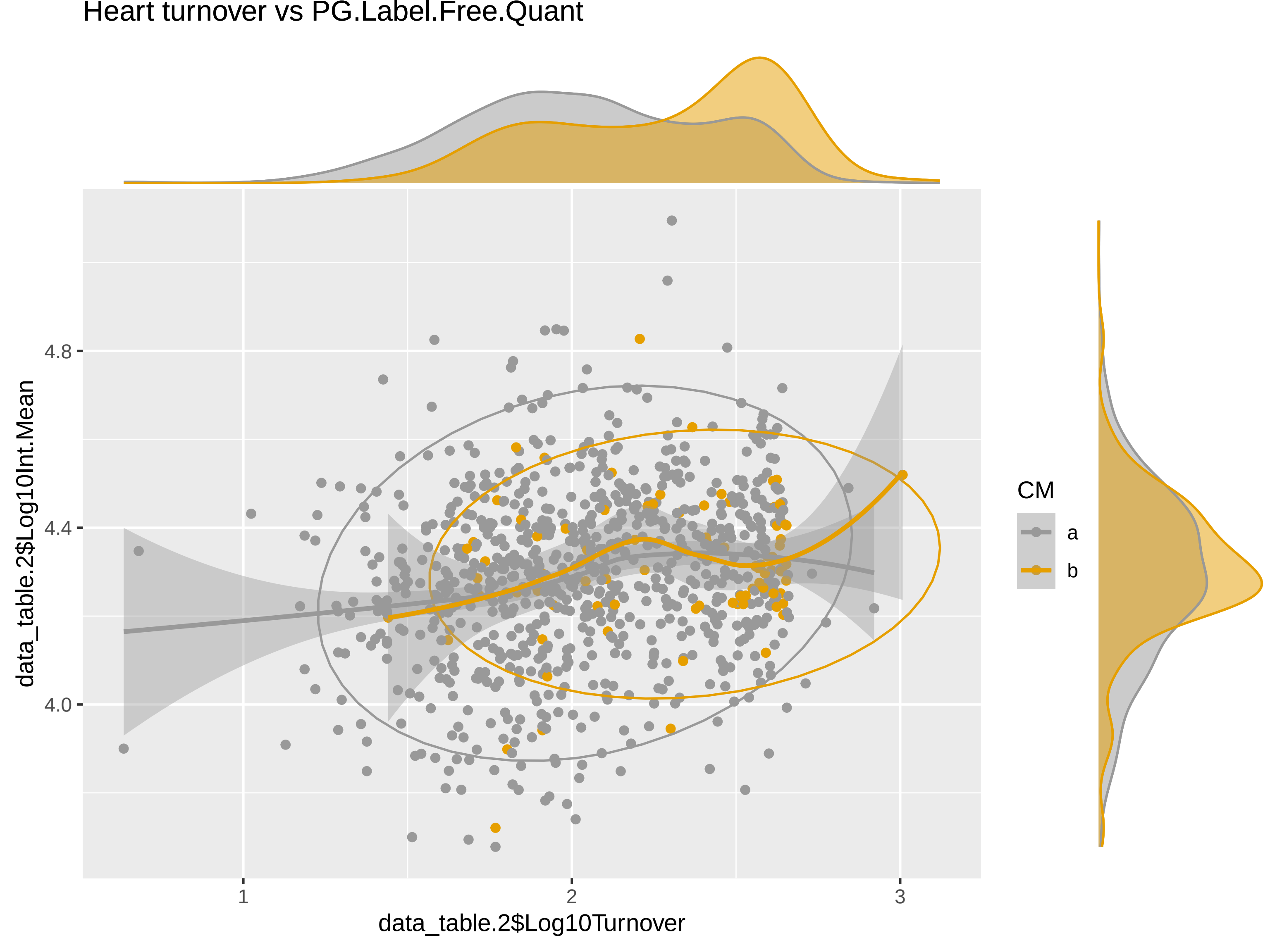
Heart turnover vs PG.Label.Free.Quant
data_table.2$Log10Int.Mean
4.8
4.4
4.0
1
2
3
data_table.2$Log10Turnover
CM
a
b
