# Supplementary material for: Mapping protein carboxymethylation sites provides insights into their role in proteostasis and cell proliferation
Source: Nat Commun. 2021 Nov 18;12:6743. doi: 10.1038/s41467-021-26982-6 (PMC8602705; doi:10.1038/s41467-021-26982-6)
Supplement: Supplementary file 11 — Source Data [file 41467_2021_26982_MOESM11_ESM.zip › FigureS2/2F/Figure2_E_left.pdf]

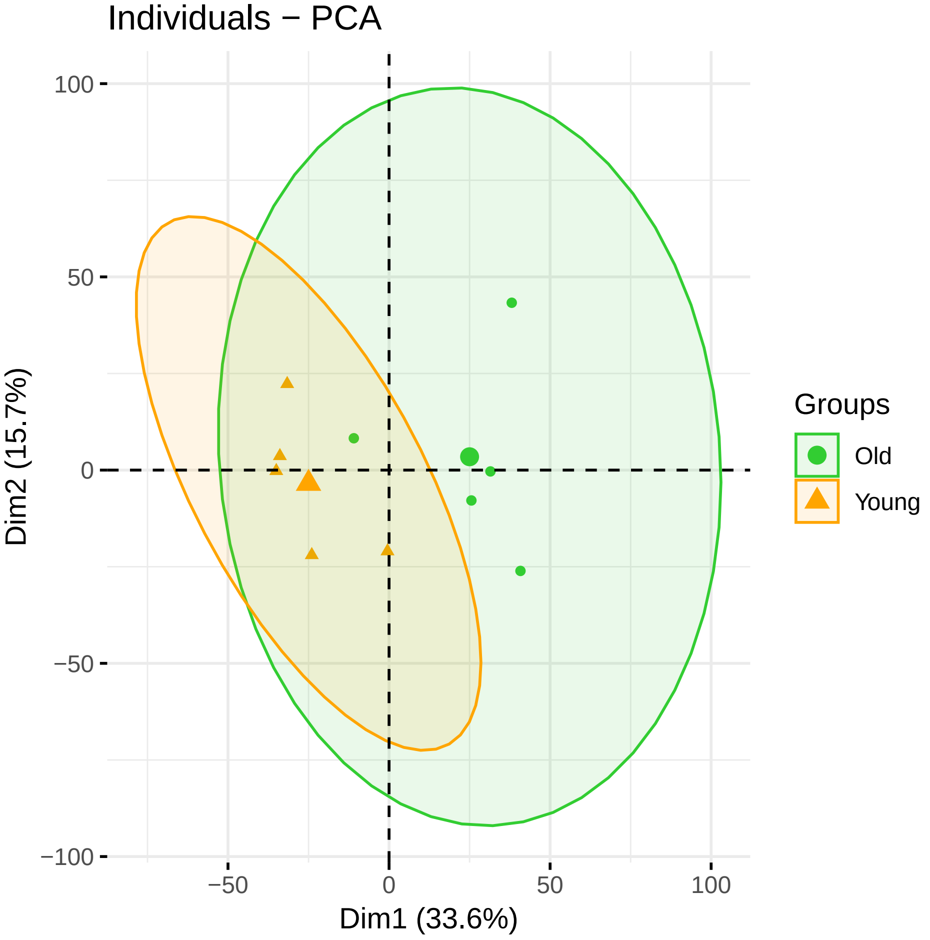

Individuals − PCA
100
50
0
−50
−100
Dim2 (15.7%)
−50
0
50
100
Dim1 (33.6%)
Groups
Old
Young
