# Supplementary material for: Mapping protein carboxymethylation sites provides insights into their role in proteostasis and cell proliferation
Source: Nat Commun. 2021 Nov 18;12:6743. doi: 10.1038/s41467-021-26982-6 (PMC8602705; doi:10.1038/s41467-021-26982-6)
Supplement: Supplementary file 11 — Source Data [file 41467_2021_26982_MOESM11_ESM.zip › FigureS2/2G/FigureS2_G_bottom.pdf]

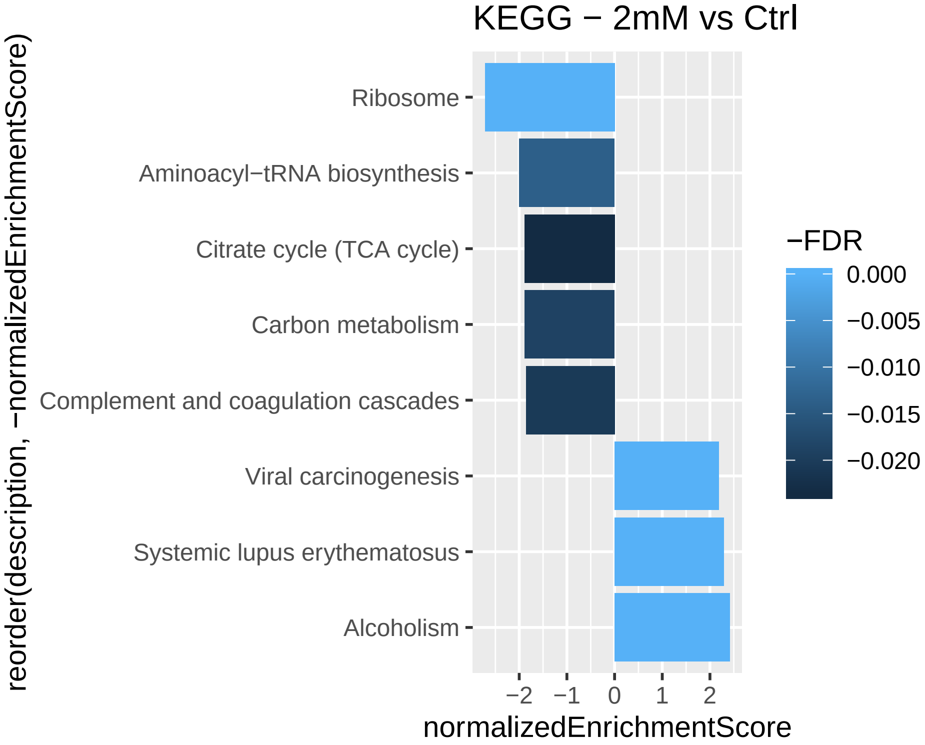
KEGG − 2mM vs Ctrl
Ribosome
Aminoacyl−tRNA biosynthesis
Citrate cycle (TCA cycle)
Carbon metabolism
Complement and coagulation cascades
Viral carcinogenesis
Systemic lupus erythematosus
Alcoholism
reorder(description, −normalizedEnrichmentScore)
−2
−1
0
1
2
normalizedEnrichmentScore
−FDR
0.000
−0.005
−0.010
−0.015
−0.020
