# Supplementary material for: Mapping protein carboxymethylation sites provides insights into their role in proteostasis and cell proliferation
Source: Nat Commun. 2021 Nov 18;12:6743. doi: 10.1038/s41467-021-26982-6 (PMC8602705; doi:10.1038/s41467-021-26982-6)
Supplement: Supplementary file 11 — Source Data [file 41467_2021_26982_MOESM11_ESM.zip › FigureS2/2G/FigureS2_G_middle.pdf]

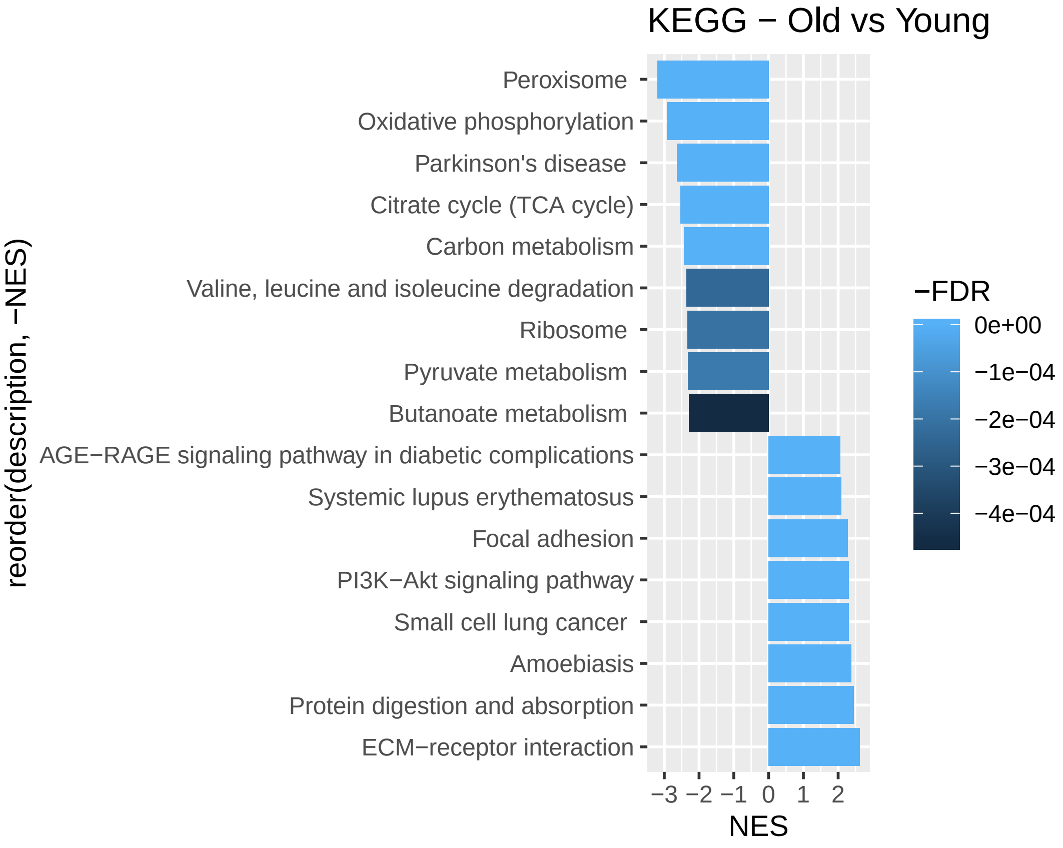
KEGG − Old vs Young
Peroxisome
Oxidative phosphorylation
Parkinson's disease
Citrate cycle (TCA cycle)
Carbon metabolism
Valine, leucine and isoleucine degradation
Ribosome
Pyruvate metabolism
Butanoate metabolism
AGE−RAGE signaling pathway in diabetic complications
Systemic lupus erythematosus
Focal adhesion
PI3K−Akt signaling pathway
Small cell lung cancer
Amoebiasis
Protein digestion and absorption
ECM−receptor interaction
reorder(description, −NES)
−3 −2 −1 0 1 2
NES
−FDR
0e+00
−1e−04
−2e−04
−3e−04
−4e−04
