# Supplementary material for: Mapping protein carboxymethylation sites provides insights into their role in proteostasis and cell proliferation
Source: Nat Commun. 2021 Nov 18;12:6743. doi: 10.1038/s41467-021-26982-6 (PMC8602705; doi:10.1038/s41467-021-26982-6)
Supplement: Supplementary file 11 — Source Data [file 41467_2021_26982_MOESM11_ESM.zip › FigureS2/2G/FigureS2_G_upper.pdf]

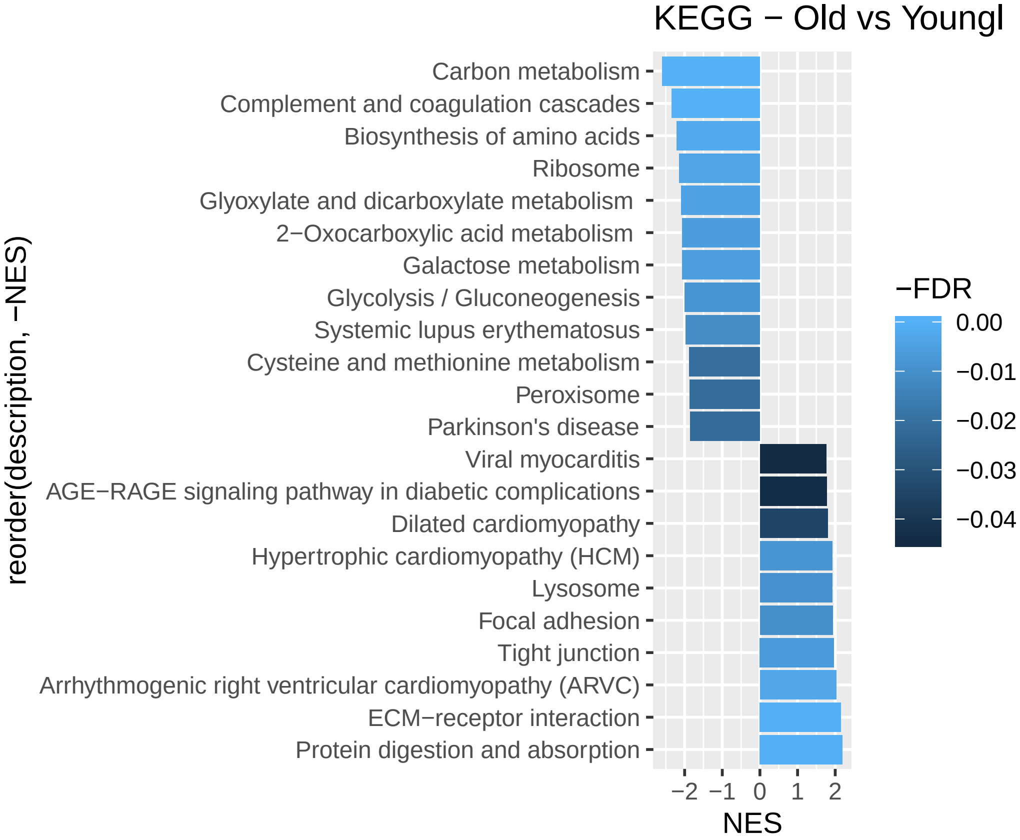
KEGG − Old vs YoungI
Carbon metabolism
Complement and coagulation cascades
Biosynthesis of amino acids
Ribosome
Glyoxylate and dicarboxylate metabolism
2−Oxocarboxylic acid metabolism
Galactose metabolism
Glycolysis / Gluconeogenesis
Systemic lupus erythematosus
Cysteine and methionine metabolism
Peroxisome
Parkinson's disease
Viral myocarditis
AGE−RAGE signaling pathway in diabetic complications
Dilated cardiomyopathy
Hypertrophic cardiomyopathy (HCM)
Lysosome
Focal adhesion
Tight junction
Arrhythmogenic right ventricular cardiomyopathy (ARVC)
ECM−receptor interaction
Protein digestion and absorption
reorder(description, −NES)
−2 −1 0 1 2
NES
−FDR
0.00
−0.01
−0.02
−0.03
−0.04
