# Supplementary material for: Mapping protein carboxymethylation sites provides insights into their role in proteostasis and cell proliferation
Source: Nat Commun. 2021 Nov 18;12:6743. doi: 10.1038/s41467-021-26982-6 (PMC8602705; doi:10.1038/s41467-021-26982-6)

a

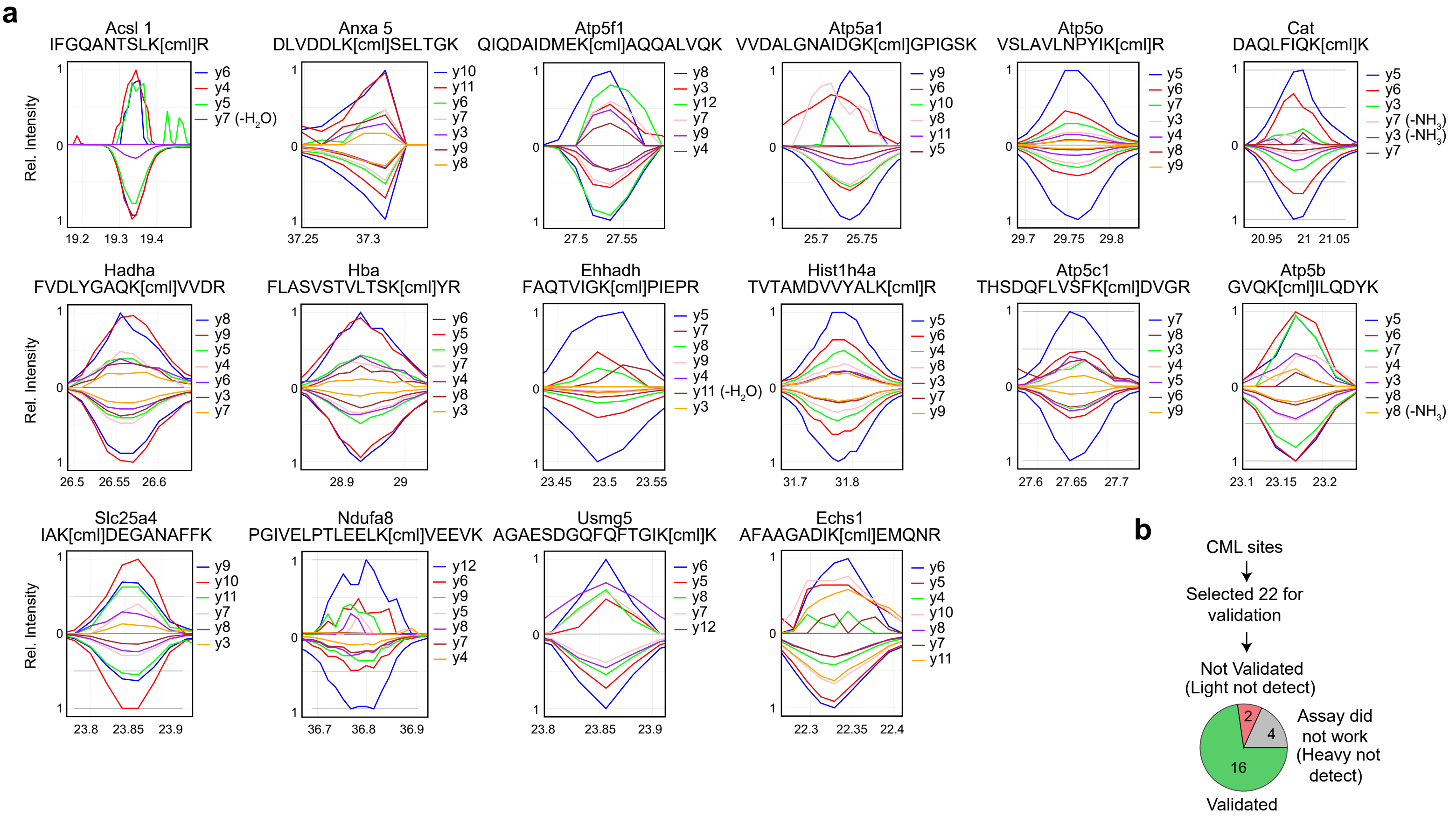

c

ID	Gene	CM site
P62806	HIST1H4A	Heart, Kidney, Liver (K92)
P43277	HIST1H1D	Heart (K64)
P10853	HIST1H2BF, HIST1H2BM, HIST1H2BB, HIST1H2BH, HIST2H2BB, HIST1H2BC, HIST1H2BK, HIST1H2BP	Heart (K47; K35)
P22752	HIST1H2AB, HIST3H2A, HIST1H2AF, HIST1H2AH, HIST1H2AK, H2AFJ	Heart, Kidney, Liver (K96)
P02301	H3F3C, H3F3A	Liver (K123)
Q64523	HIST2H2AC, HIST2H2AA1	Liver (K96)

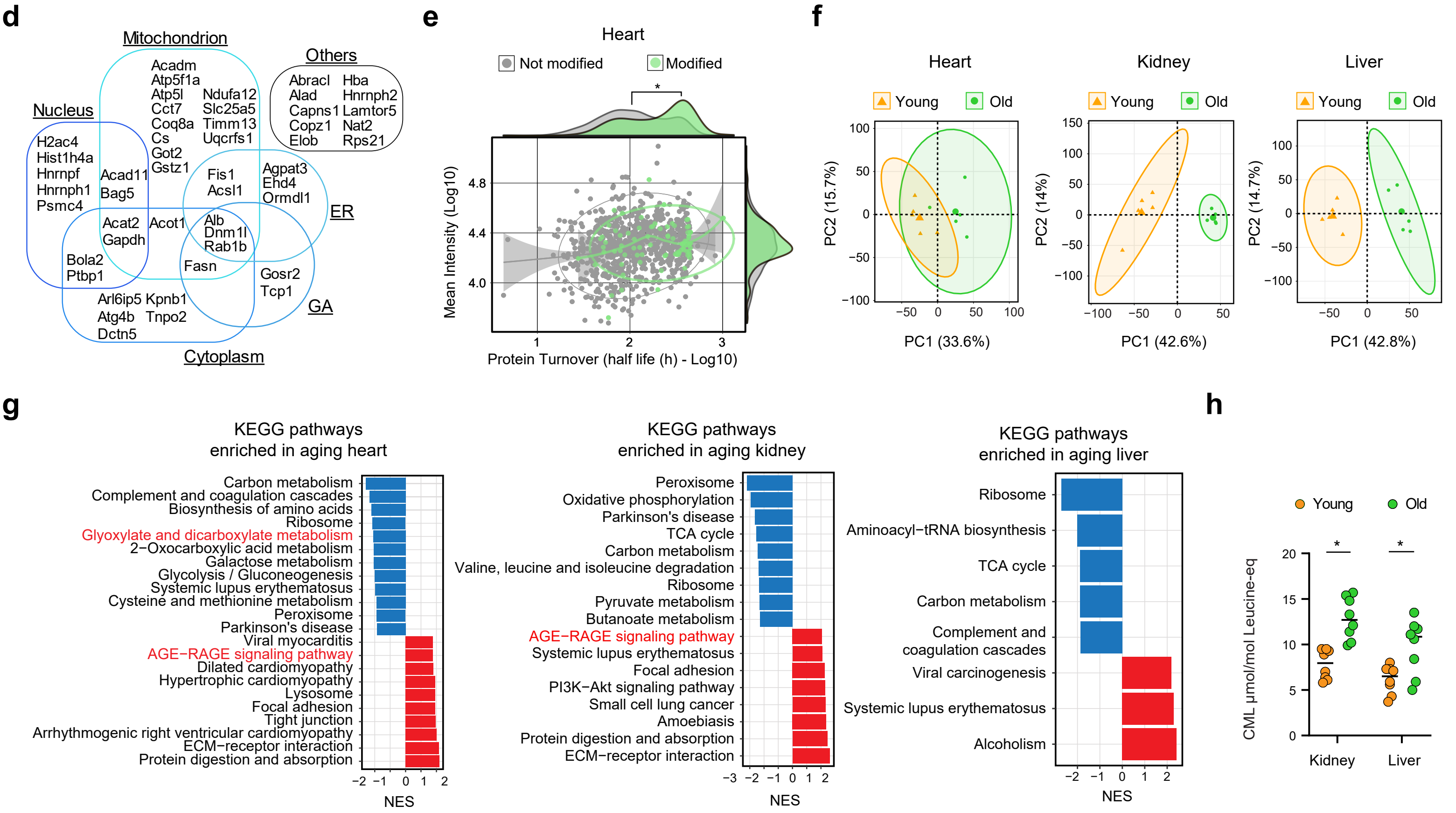

Supplement: Supplementary file 11 — Source Data [file 41467_2021_26982_MOESM11_ESM.zip › FigureS2/FigureS2.pdf]
