# Supplementary material for: Mapping protein carboxymethylation sites provides insights into their role in proteostasis and cell proliferation
Source: Nat Commun. 2021 Nov 18;12:6743. doi: 10.1038/s41467-021-26982-6 (PMC8602705; doi:10.1038/s41467-021-26982-6)
Supplement: Supplementary file 11 — Source Data [file 41467_2021_26982_MOESM11_ESM.zip › FigureS3/3B/FigureS3_B_first.pdf]

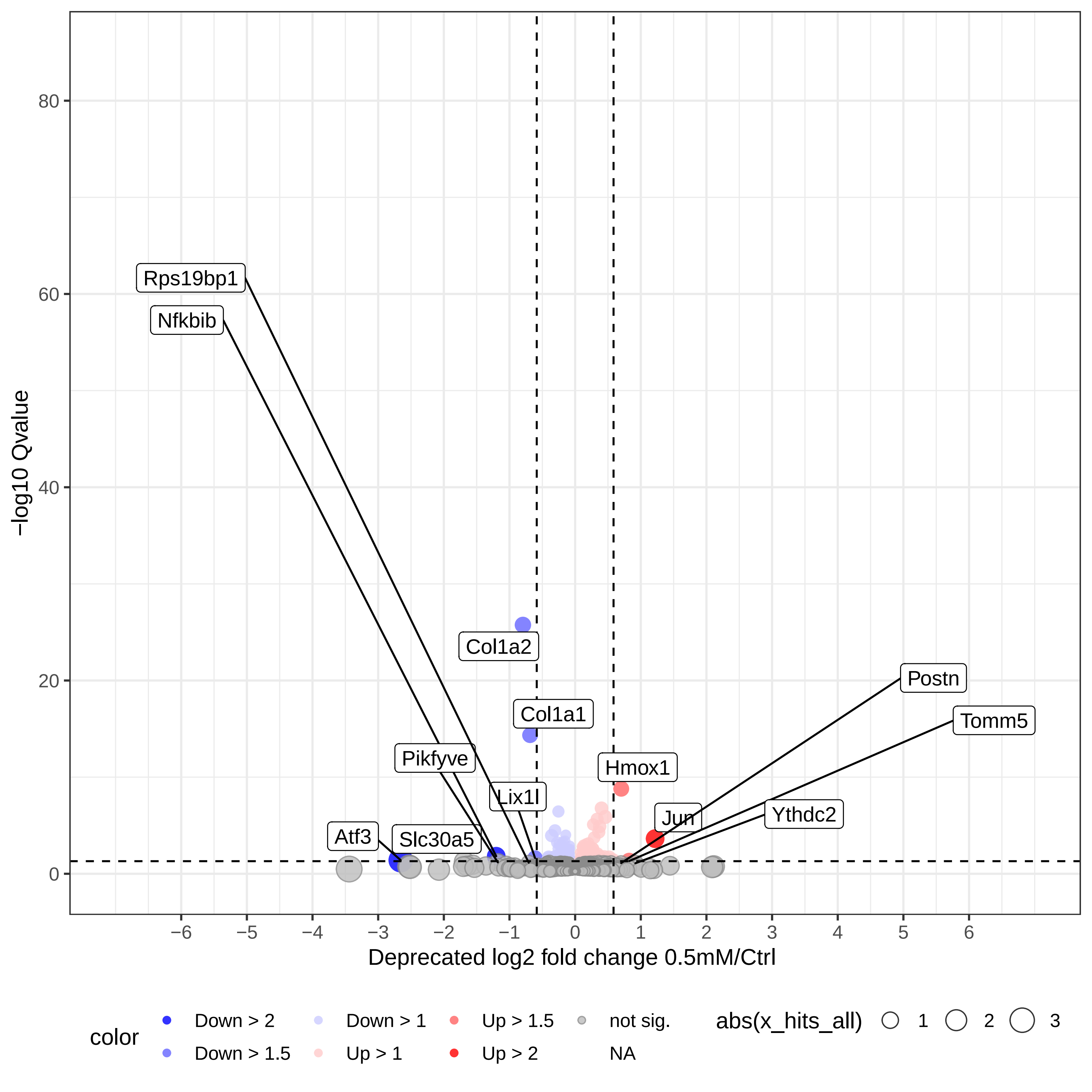

−log10 Qvalue
80
60
40
20
0
−6
−5
−4
−3
−2
−1
0
1
2
3
4
5
6
Deprecated log2 fold change 0.5mM/Ctrl
Rps19bp1
Nfkbib
Col1a2
Col1a1
Pikfyve
Lix1l
Hmox1
Postn
Tomm5
Jun
Ythdc2
Atf3
Slc30a5
color
Down > 2
Down > 1.5
Down > 1
Up > 1
Up > 1.5
Up > 2
not sig.
NA
abs(x_hits_all)
1
2
3
