# Supplementary material for: Mapping protein carboxymethylation sites provides insights into their role in proteostasis and cell proliferation
Source: Nat Commun. 2021 Nov 18;12:6743. doi: 10.1038/s41467-021-26982-6 (PMC8602705; doi:10.1038/s41467-021-26982-6)
Supplement: Supplementary file 11 — Source Data [file 41467_2021_26982_MOESM11_ESM.zip › FigureS3/3B/FigureS3_B_first_right.pdf]

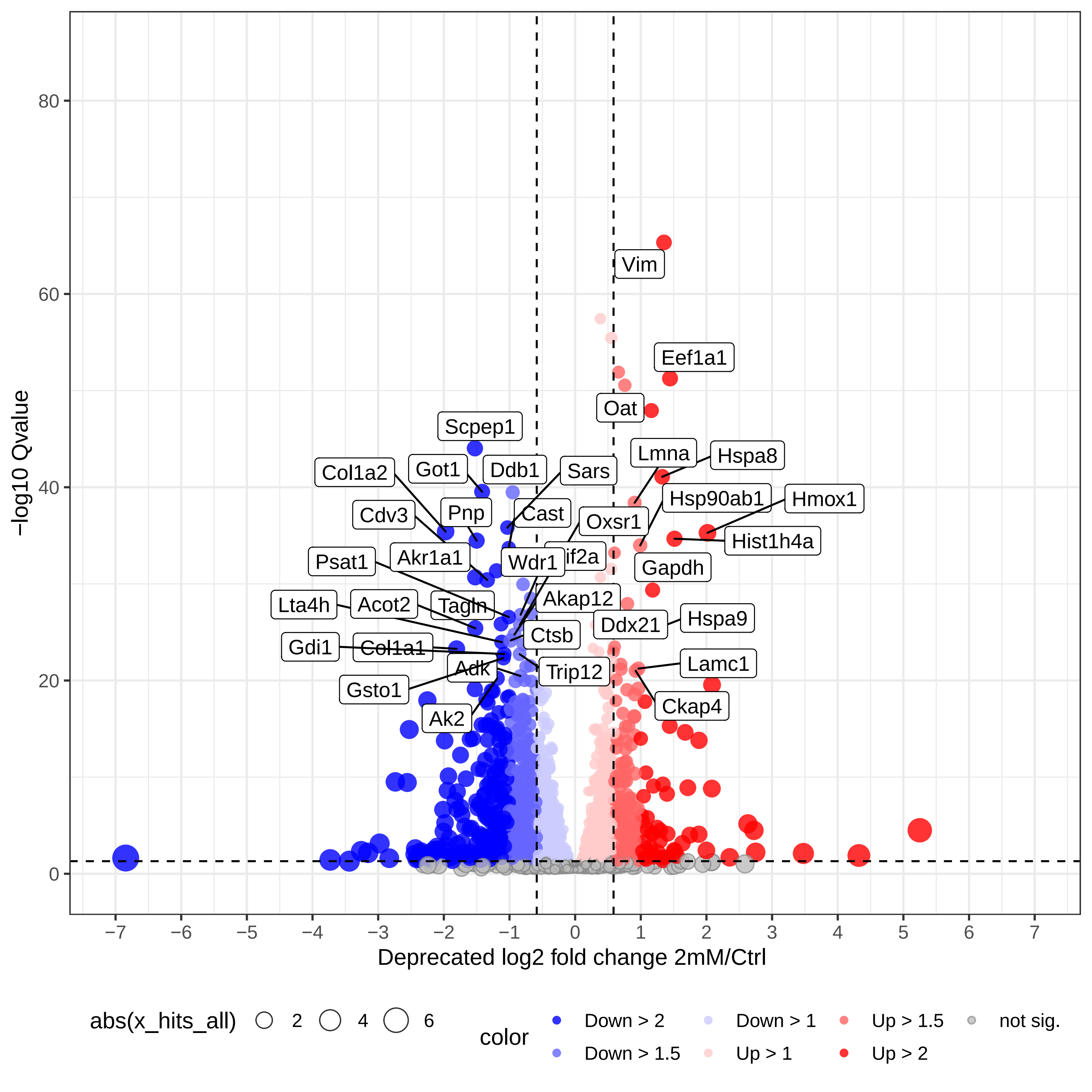

-log10 Qvalue
Deprecated log2 fold change 2mM/Ctrl
80
60
40
20
0
-7
-6
-5
-4
-3
-2
-1
0
1
2
3
4
5
6
7
Vim
Eef1a1
Oat
Scpep1
Lmna
Hspa8
Col1a2
Got1
Ddb1
Sars
Hsp90ab1
Hmox1
Cdv3
Pnp
Cast
Oxsr1
Hist1h4a
Psat1
Akr1a1
Wdr1
Gapdh
Lta4h
Acot2
Tagln
Akap12
Ddx21
Hspa9
Ctsb
Gdi1
Col1a1
Adk
Trip12
Lamc1
Gsto1
Ckap4
Ak2
abs(x_hits_all)
2
4
6
color
Down > 2
Down > 1.5
Down > 1
Up > 1
Up > 1.5
Up > 2
not sig.
