# Supplementary material for: Mapping protein carboxymethylation sites provides insights into their role in proteostasis and cell proliferation
Source: Nat Commun. 2021 Nov 18;12:6743. doi: 10.1038/s41467-021-26982-6 (PMC8602705; doi:10.1038/s41467-021-26982-6)
Supplement: Supplementary file 11 — Source Data [file 41467_2021_26982_MOESM11_ESM.zip › FigureS3/3B/FigureS3_B_second.pdf]

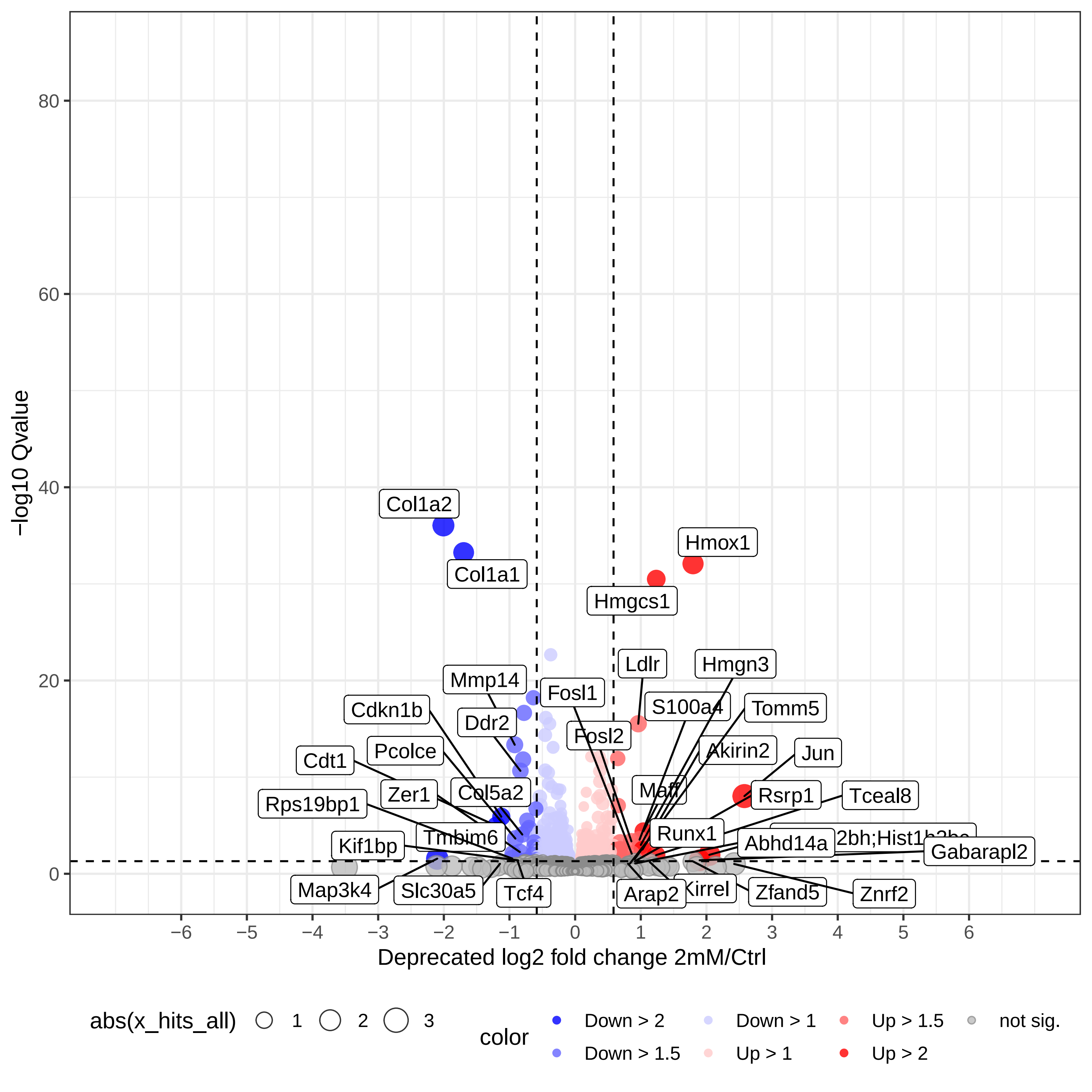

−log10 Qvalue
80
60
40
20
0
Deprecated log2 fold change 2mM/Ctrl
−6
−5
−4
−3
−2
−1
0
1
2
3
4
5
6
Col1a2
Col1a1
Hmox1
Hmgcs1
Ldlr
Hmgn3
Mmp14
Fosl1
S100a4
Tomm5
Cdkn1b
Ddr2
Fosl2
Akirin2
Jun
Cdt1
Pcolce
Maff
Rsrp1
Tceal8
Zer1
Col5a2
Rps19bp1
Runx1
Abhd14a
Gabarapl2
Tmbim6
Kif1bp
Map3k4
Slc30a5
Tcf4
Arap2
Kirrel
Zfand5
Znrf2
abs(x_hits_all)
1
2
3
color
Down > 2
Down > 1.5
Down > 1
Up > 1
Up > 1.5
Up > 2
not sig.
