# Supplementary material for: Mapping protein carboxymethylation sites provides insights into their role in proteostasis and cell proliferation
Source: Nat Commun. 2021 Nov 18;12:6743. doi: 10.1038/s41467-021-26982-6 (PMC8602705; doi:10.1038/s41467-021-26982-6)
Supplement: Supplementary file 11 — Source Data [file 41467_2021_26982_MOESM11_ESM.zip › FigureS3/3B/FigureS3_B_third.pdf]

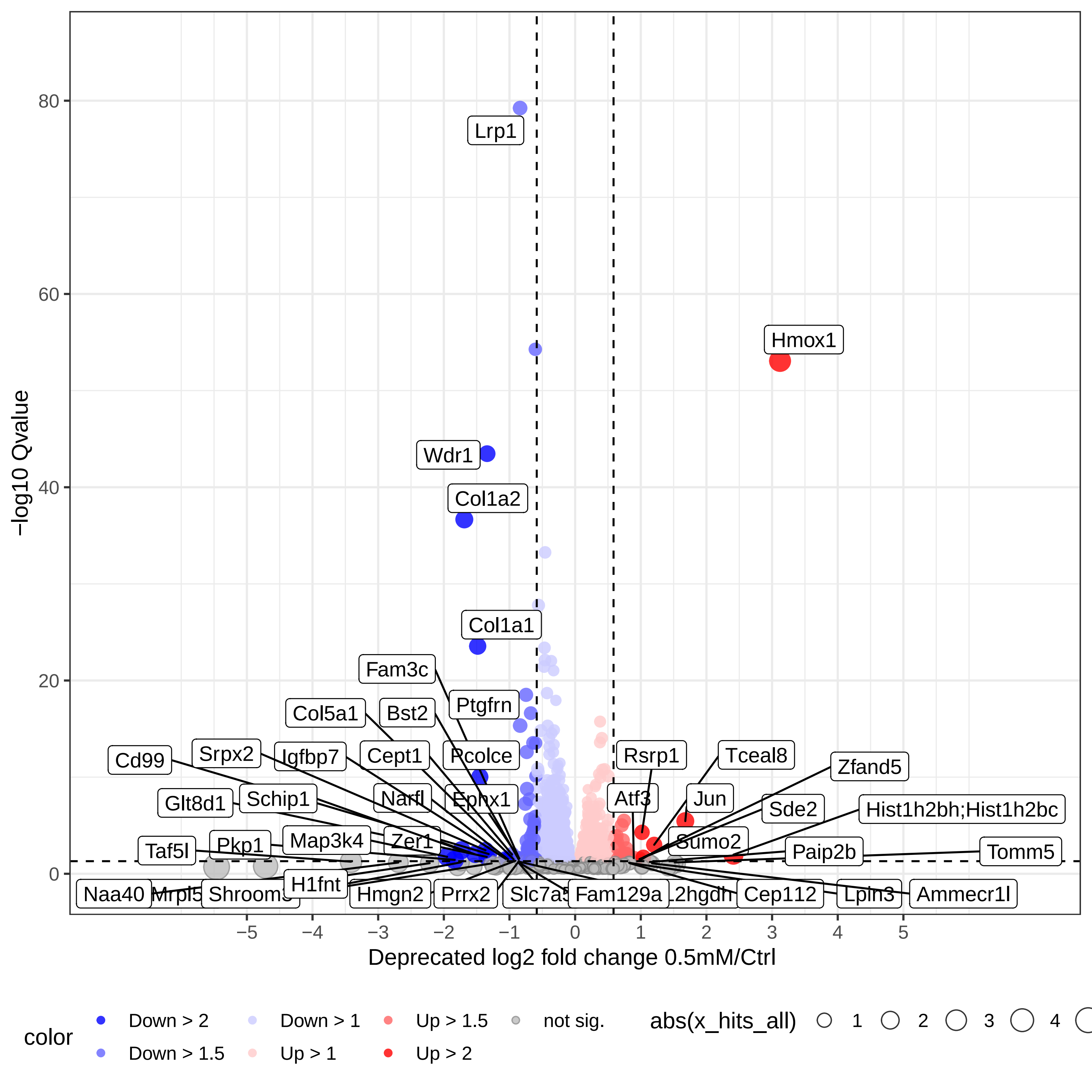

80
60
40
20
0
−log10 Qvalue
Lrp1
Hmox1
Wdr1
Col1a2
Col1a1
Fam3c
Ptgfrn
Col5a1
Bst2
Cd99
Srpx2
Igfbp7
Cept1
Pcolce
Rsrp1
Tceal8
Zfand5
Glt8d1
Schip1
Narfl
Ephx1
Atf3
Jun
Sde2
Hist1h2bh;Hist1h2bc
Taf5l
Pkp1
Map3k4
Zer1
Sumo2
Paip2b
Tomm5
H1fnt
Naa40
Shroom3
Hmgn2
Prrx2
Fam129a
Cep112
Lpin3
Ammecr1l
−5
−4
−3
−2
−1
0
1
2
3
4
5
Deprecated log2 fold change 0.5mM/Ctrl
color
Down > 2
Down > 1.5
Down > 1
Up > 1
Up > 1.5
Up > 2
not sig.
abs(x_hits_all)
1
2
3
4
