# Supplementary material for: Mapping protein carboxymethylation sites provides insights into their role in proteostasis and cell proliferation
Source: Nat Commun. 2021 Nov 18;12:6743. doi: 10.1038/s41467-021-26982-6 (PMC8602705; doi:10.1038/s41467-021-26982-6)
Supplement: Supplementary file 11 — Source Data [file 41467_2021_26982_MOESM11_ESM.zip › FigureS3/3C/FigureS3_C.pdf]

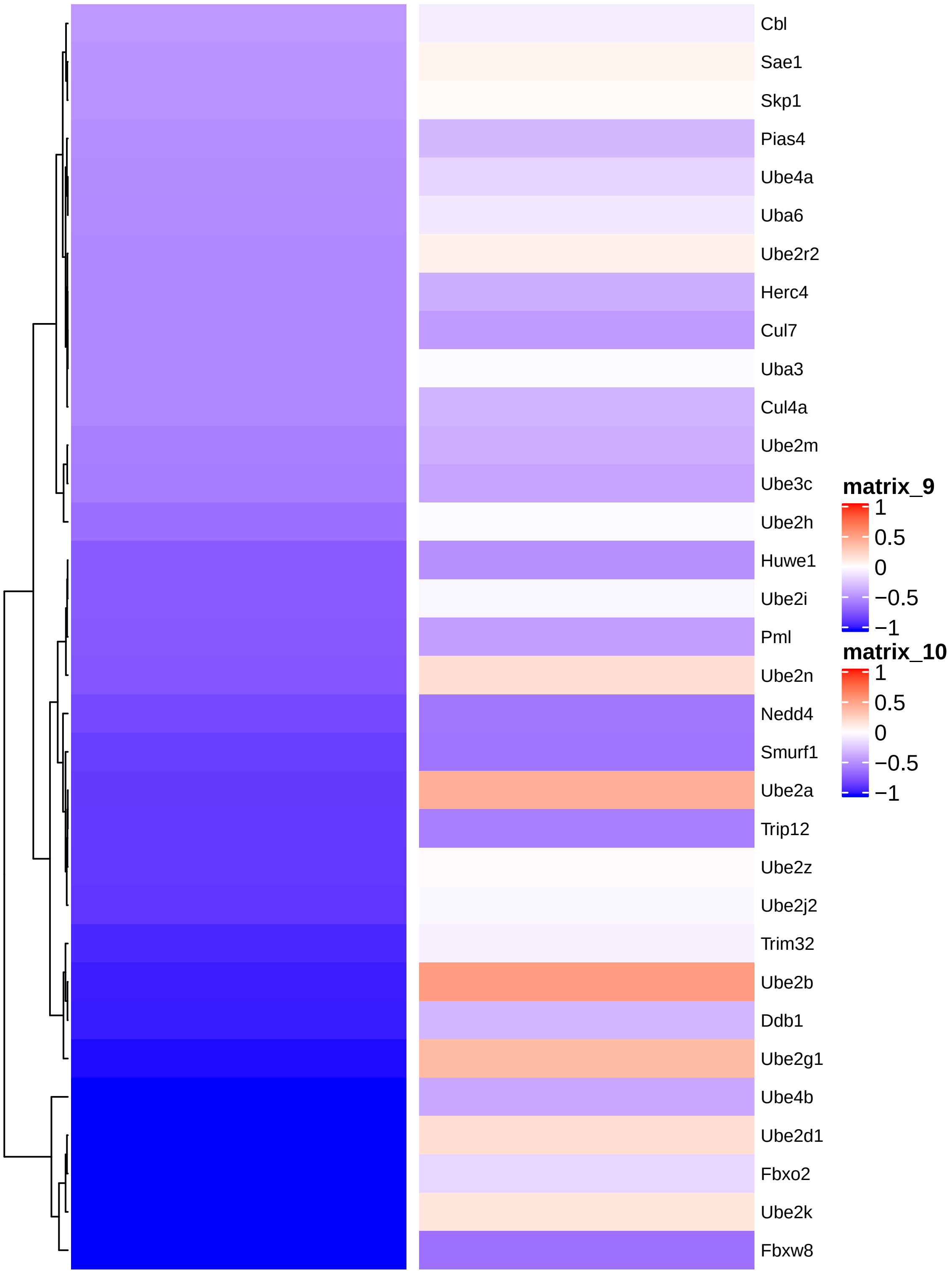

Cbl
Sae1
Skp1
Pias4
Ube4a
Uba6
Ube2r2
Herc4
Cul7
Uba3
Cul4a
Ube2m
Ube3c
Ube2h
Huwe1
Ube2i
Pml
Ube2n
Nedd4
Smurf1
Ube2a
Trip12
Ube2z
Ube2j2
Trim32
Ube2b
Ddb1
Ube2g1
Ube4b
Ube2d1
Fbxo2
Ube2k
Fbxw8
matrix_9
1
0.5
0
−0.5
−1
matrix_10
1
0.5
0
−0.5
−1
