# Supplementary material for: Mapping protein carboxymethylation sites provides insights into their role in proteostasis and cell proliferation
Source: Nat Commun. 2021 Nov 18;12:6743. doi: 10.1038/s41467-021-26982-6 (PMC8602705; doi:10.1038/s41467-021-26982-6)
Supplement: Supplementary file 11 — Source Data [file 41467_2021_26982_MOESM11_ESM.zip › FigureS3/3D/FigureS3_D.pdf]

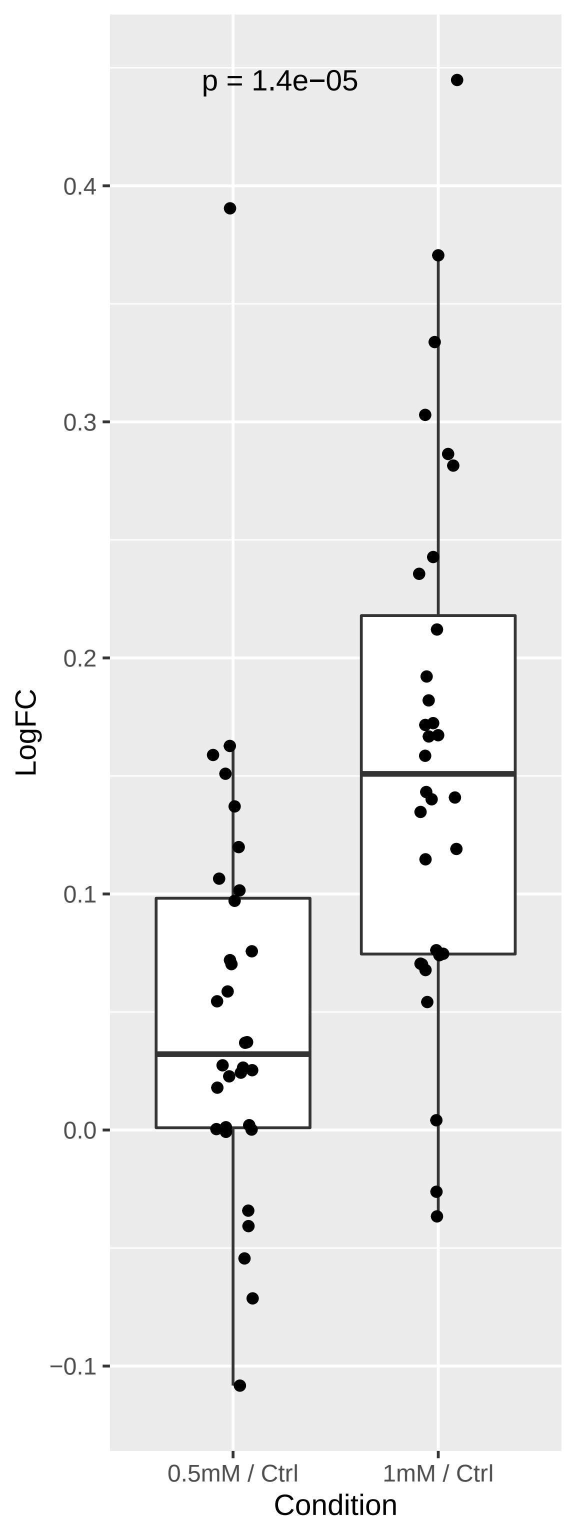

p = 1.4e−05
0.4
0.3
0.2
0.1
0.0
−0.1
LogFC
0.5mM / Ctrl
1mM / Ctrl
Condition
