# Supplementary material for: Mapping protein carboxymethylation sites provides insights into their role in proteostasis and cell proliferation
Source: Nat Commun. 2021 Nov 18;12:6743. doi: 10.1038/s41467-021-26982-6 (PMC8602705; doi:10.1038/s41467-021-26982-6)

a

ID	Gene	Type	Protein name	CM site
Q9R1P4	PSMA1	26S Proteasome	Proteasome subunit alpha type-1	MEF (K30)
P54775	PSMC4	26S Proteasome	26S protease regulatory subunit 6B	MEF (K330), Heart, Kidney, Liver (ProtNterm)
Q9R1P3	PSMB2	26S Proteasome	Proteasome subunit beta type-2	Kidney (ProtNterm)
Q3TXS7	PSMD1	26S Proteasome	26S proteasome non-ATPase regulatory subunit 1	Kidney (ProtNterm)
O95352	ATG7	E1 ligase	Ubiquitin-like modifier-activating enzyme ATG7	HUVEC (K140)
Q9Z1F9	UBA2	E1 ligase	SUMO-activating enzyme subunit 2	MEF (K271), HUVEC (K271)
Q8C7R4	UBA6	E1 ligase	Ubiquitin-like modifier-activating enzyme 6	MEF (K519)
P62983	RPS27A	E1, E2, E3 ligase	Ubiquitin-40S ribosomal protein S27a	MEF (K63; K11; K6; K33), HUVEC (K63; K11; K27), Heart (K11; K48)
P63280	UBE2I	E2 ligase	SUMO-conjugating enzyme UBC9	MEF (K74)
P68037	UBE2L3	E2 ligase	Ubiquitin-conjugating enzyme E2 L3	MEF (K73)
Q6ZPJ3	UBE2O	E2 ligase	(E3-independent) E2 ubiquitin-conjugating enzyme UBE2O	MEF (K360; K368; K379; K380)
Q9Z0H7	BCL10	E3 ligase	B-cell lymphoma/leukemia 10	MEF (K115; K118)
Q7TMY8	HUWE1	E3 ligase	E3 ubiquitin-protein ligase HUWE1	MEF (K4137; K4142), HUVEC (K4134; K4139)
P46935	NEDD4	E3 ligase	E3 ubiquitin-protein ligase NEDD4	MEF (K484; K429; K186)
P17918	PCNA	E3 ligase	Proliferating cell nuclear antigen	MEF (K254; K117; K80)
P29590	PML	E3 ligase	Protein PML	HUVEC (K487; K490)
Q8BFU3	RNF214	E3 ligase	RING finger protein 214	MEF (K136; K142; K360; K366)
Q8K2Y0	RNF219	E3 ligase	RING finger protein 219	MEF (K498; K505)
Q62318	TRIM28	E3 ligase	Transcription intermediary factor 1-beta	MEF (K774; K779; K770), HUVEC (K366; K304)
Q6WKZ8	UBR2	E3 ligase	E3 ubiquitin-protein ligase UBR2	MEF (K1496)
A2AN08	UBR4	E3 ligase	E3 ubiquitin-protein ligase UBR4	MEF (K1929), HUVEC (K1931; K1060)
Q8BU04	UBR7	E3 ligase	Putative E3 ubiquitin-protein ligase UBR7	MEF (K412; K413)
Q01853	VCP	E3 ligase	Transitional endoplasmic reticulum ATPase	MEF (K236), HUVEC (K236)
Q0P5W1	VPS8	E3 ligase	Vacuolar protein sorting-associated protein 8 homolog	MEF (K1313; K1316)
Q80YR4	ZNF598	E3 ligase	Zinc finger protein 598	MEF (K94)
B2RRE7	OTUD4	Otubain Proteases	OTU domain-containing protein 4	MEF (K990)
Q80U87	USP8	Ubiquitin-Specific Proteases	Ubiquitin carboxyl-terminal hydrolase 8	MEF (K707; K713)
P70398	USP9X	Ubiquitin-Specific Proteases	Probable ubiquitin carboxyl-terminal hydrolase FAF-X	MEF (K1722; K360)
Q92995	USP13	Ubiquitin-Specific Proteases	Ubiquitin carboxyl-terminal hydrolase 13	HUVEC (K99)
Q99LD4	GPS1	UPS-associated	COP9 signalosome complex subunit 1	MEF (K204)
P56399	USP5	UPS-associated	Ubiquitin carboxyl-terminal hydrolase 5	MEF (K288; K291), HUVEC (K288; K291)

b

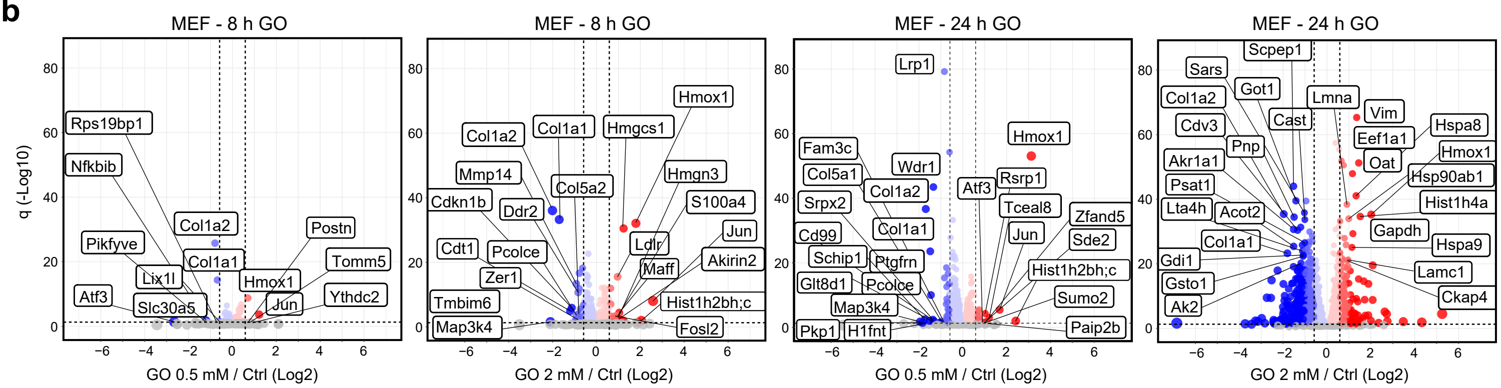

c

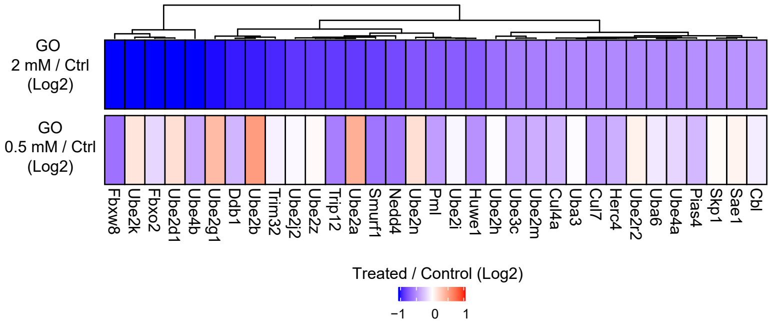

d

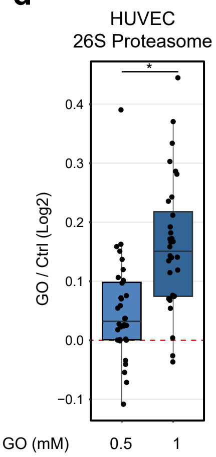

Supplement: Supplementary file 11 — Source Data [file 41467_2021_26982_MOESM11_ESM.zip › FigureS3/FigureS3.pdf]
