# Supplementary material for: Mapping protein carboxymethylation sites provides insights into their role in proteostasis and cell proliferation
Source: Nat Commun. 2021 Nov 18;12:6743. doi: 10.1038/s41467-021-26982-6 (PMC8602705; doi:10.1038/s41467-021-26982-6)
Supplement: Supplementary file 11 — Source Data [file 41467_2021_26982_MOESM11_ESM.zip › FigureS4/4A/meltCurve_PSMC5.pdf]

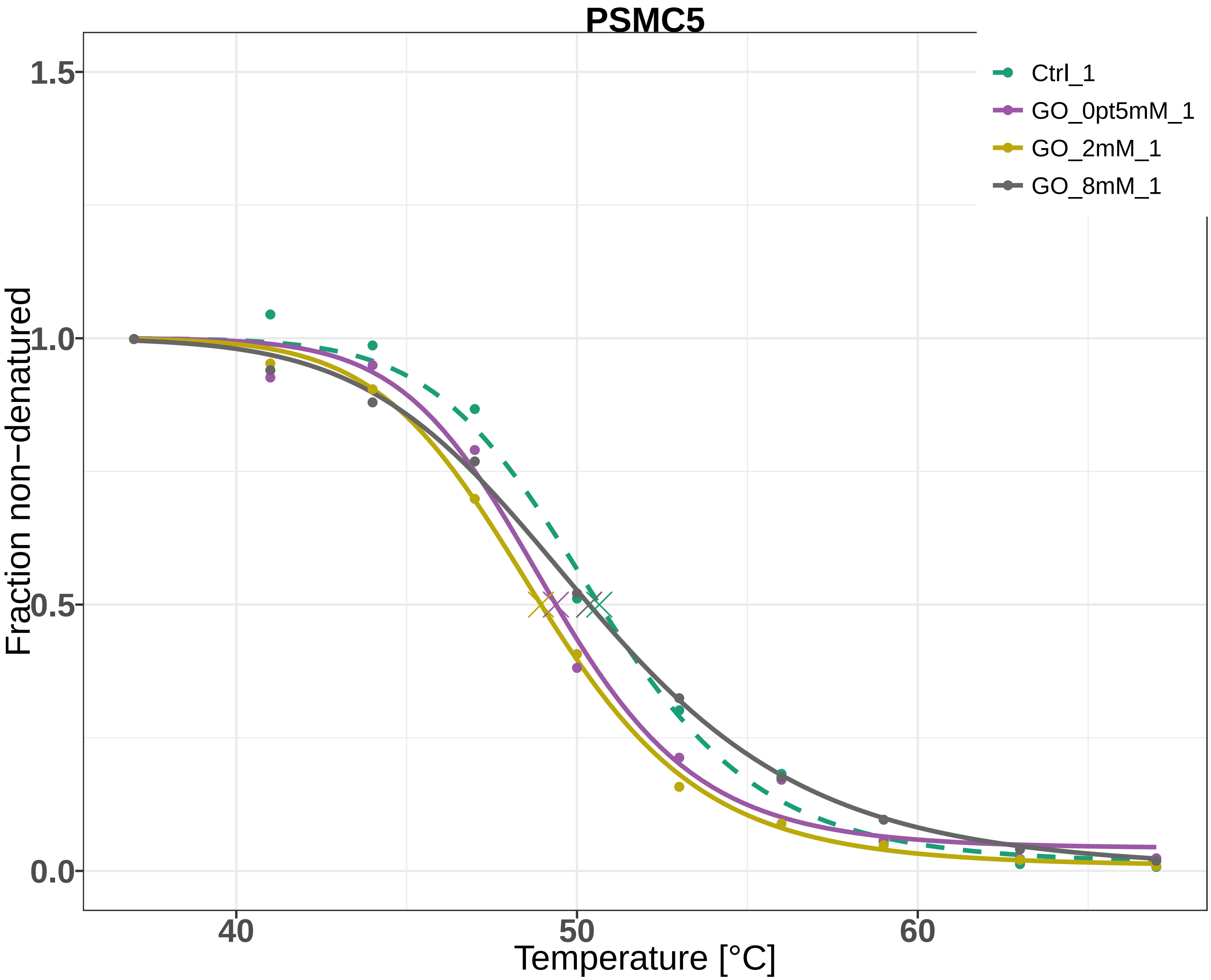

	meltPoint	slope	plateau	R2
Ctrl_1	50.65	−0.1	0.01	0.99
GO_0pt5mM_1	49.38	−0.11	0.04	0.99
GO_2mM_1	48.94	−0.1	0.01	1
GO_8mM_1	50.36	−0.076	0	1
