# Supplementary material for: Mapping protein carboxymethylation sites provides insights into their role in proteostasis and cell proliferation
Source: Nat Commun. 2021 Nov 18;12:6743. doi: 10.1038/s41467-021-26982-6 (PMC8602705; doi:10.1038/s41467-021-26982-6)
Supplement: Supplementary file 11 — Source Data [file 41467_2021_26982_MOESM11_ESM.zip › FigureS4/4A/meltCurve_PSMD14.pdf]

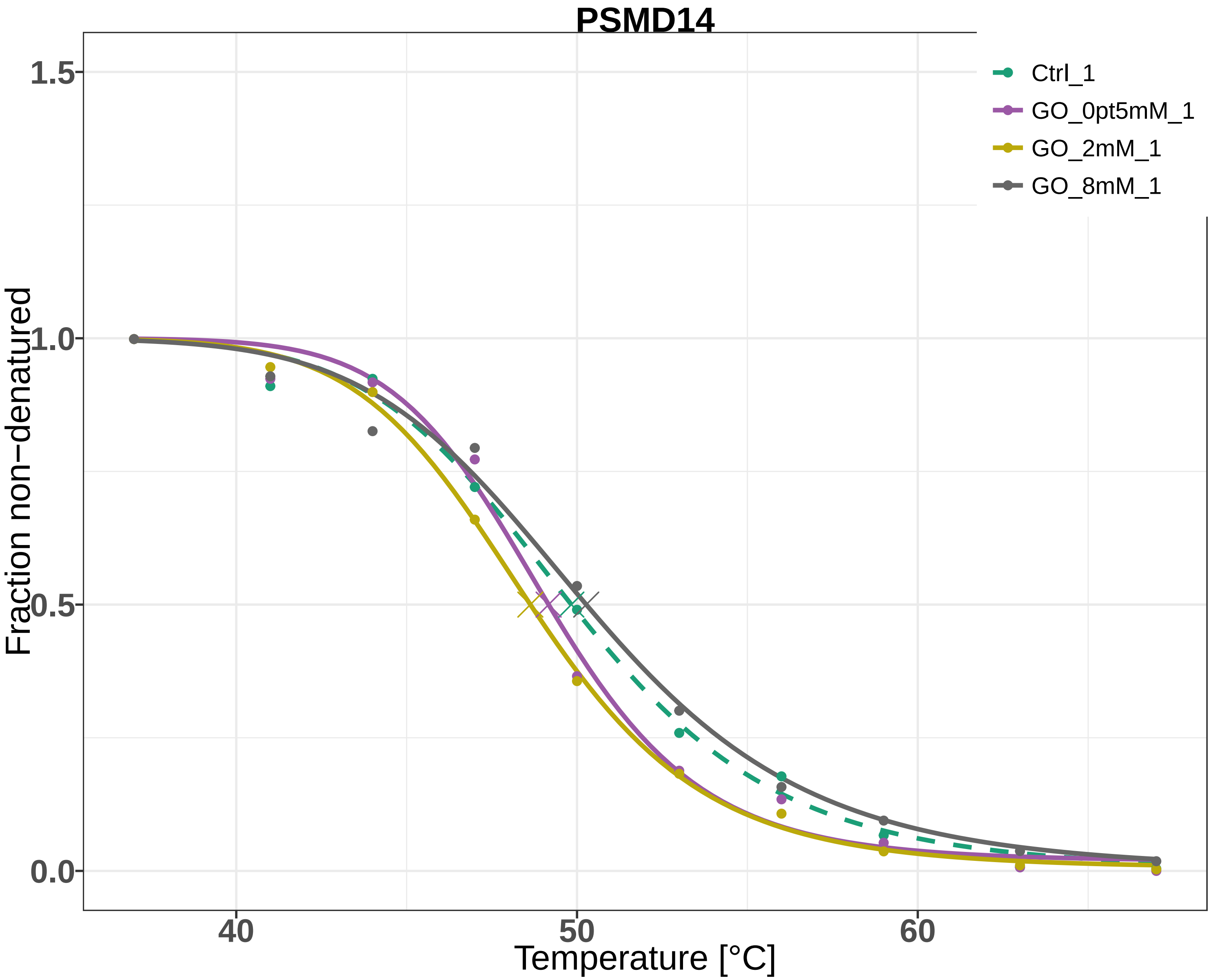


| | meltPoint | slope | plateau | R2 |
|---|---|---|---|---|
| Ctrl_1 | 49.83 | −0.082 | 0 | 1 |
| GO_0pt5mM_1 | 49.17 | −0.11 | 0.02 | 0.99 |
| GO_2mM_1 | 48.63 | −0.097 | 0.01 | 1 |
| GO_8mM_1 | 50.27 | −0.077 | 0 | 0.99 |
