# Supplementary material for: Mapping protein carboxymethylation sites provides insights into their role in proteostasis and cell proliferation
Source: Nat Commun. 2021 Nov 18;12:6743. doi: 10.1038/s41467-021-26982-6 (PMC8602705; doi:10.1038/s41467-021-26982-6)
Supplement: Supplementary file 11 — Source Data [file 41467_2021_26982_MOESM11_ESM.zip › FigureS4/4A/meltCurve_PSMD5.pdf]

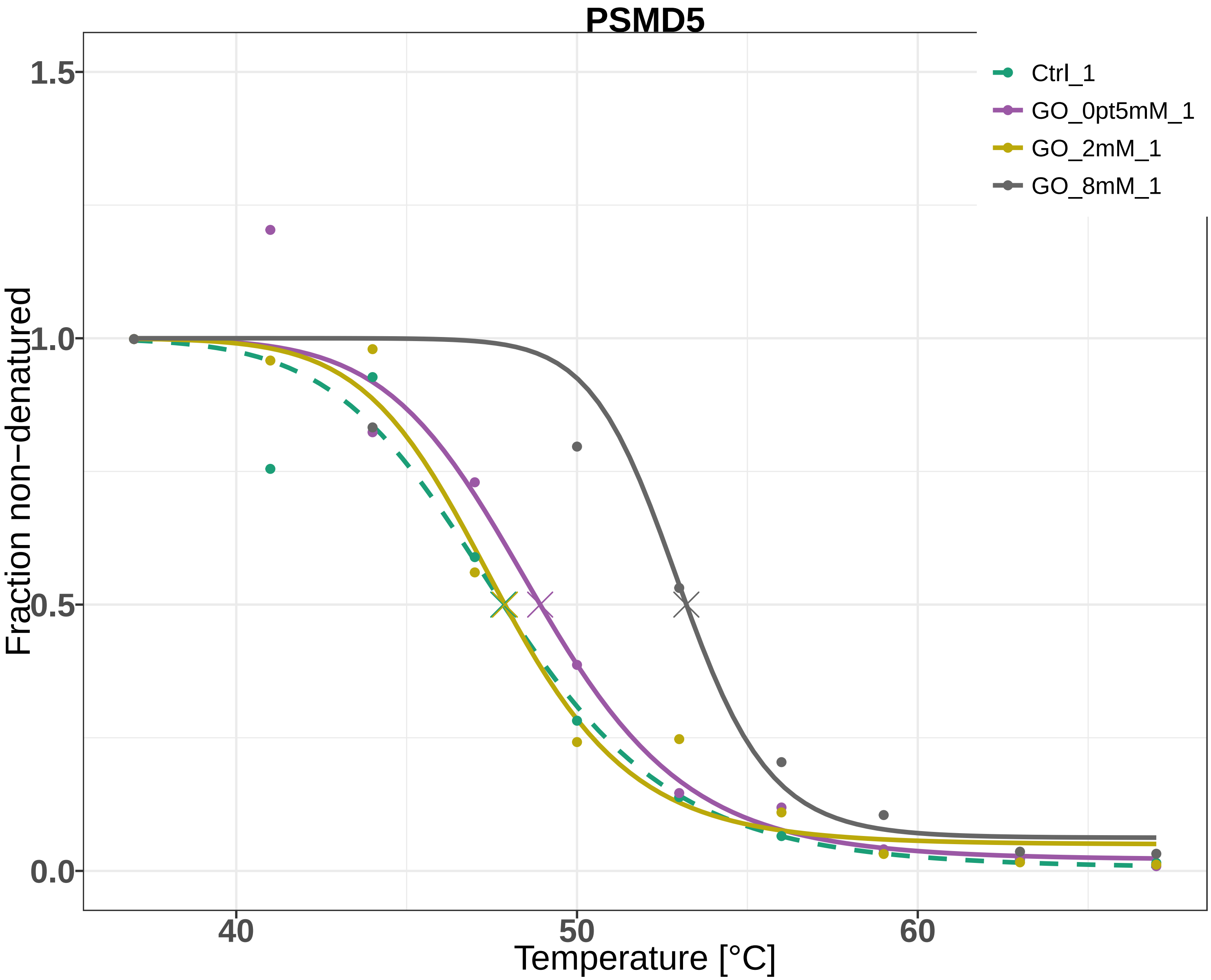

	meltPoint	slope	plateau	R2
**Ctrl_1**	**47.84**	**−0.099**	**0**	**0.96**
**GO_0pt5mM_1**	**48.92**	**−0.11**	**0.02**	**0.97**
**GO_2mM_1**	**47.88**	**−0.12**	**0.05**	**0.98**
**GO_8mM_1**	**53.21**	**−0.18**	**0.06**	**0.58**
