# Supplementary material for: Mapping protein carboxymethylation sites provides insights into their role in proteostasis and cell proliferation
Source: Nat Commun. 2021 Nov 18;12:6743. doi: 10.1038/s41467-021-26982-6 (PMC8602705; doi:10.1038/s41467-021-26982-6)
Supplement: Supplementary file 11 — Source Data [file 41467_2021_26982_MOESM11_ESM.zip › FigureS4/4A/meltCurve_PSMD7.pdf]

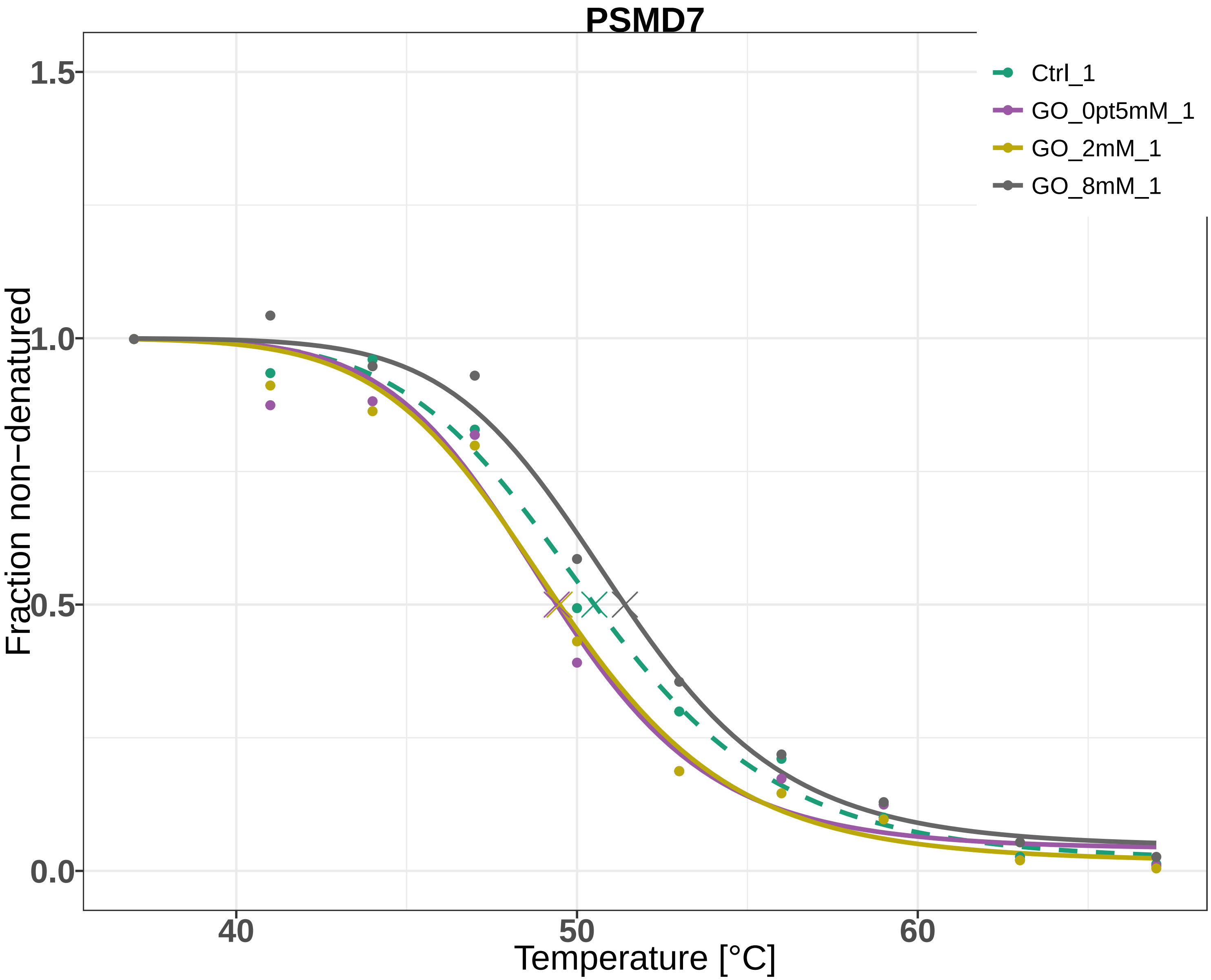

	meltPoint	slope	plateau	R2
Ctrl_1	50.51	−0.087	0.02	0.99
GO_0pt5mM_1	49.4	−0.1	0.04	0.98
GO_2mM_1	49.49	−0.095	0.02	0.99
GO_8mM_1	51.41	−0.096	0.04	0.99
