# Supplementary material for: Mapping protein carboxymethylation sites provides insights into their role in proteostasis and cell proliferation
Source: Nat Commun. 2021 Nov 18;12:6743. doi: 10.1038/s41467-021-26982-6 (PMC8602705; doi:10.1038/s41467-021-26982-6)
Supplement: Supplementary file 11 — Source Data [file 41467_2021_26982_MOESM11_ESM.zip › FigureS4/4B/201005_DensityPlot_MeltingTemp.pdf]

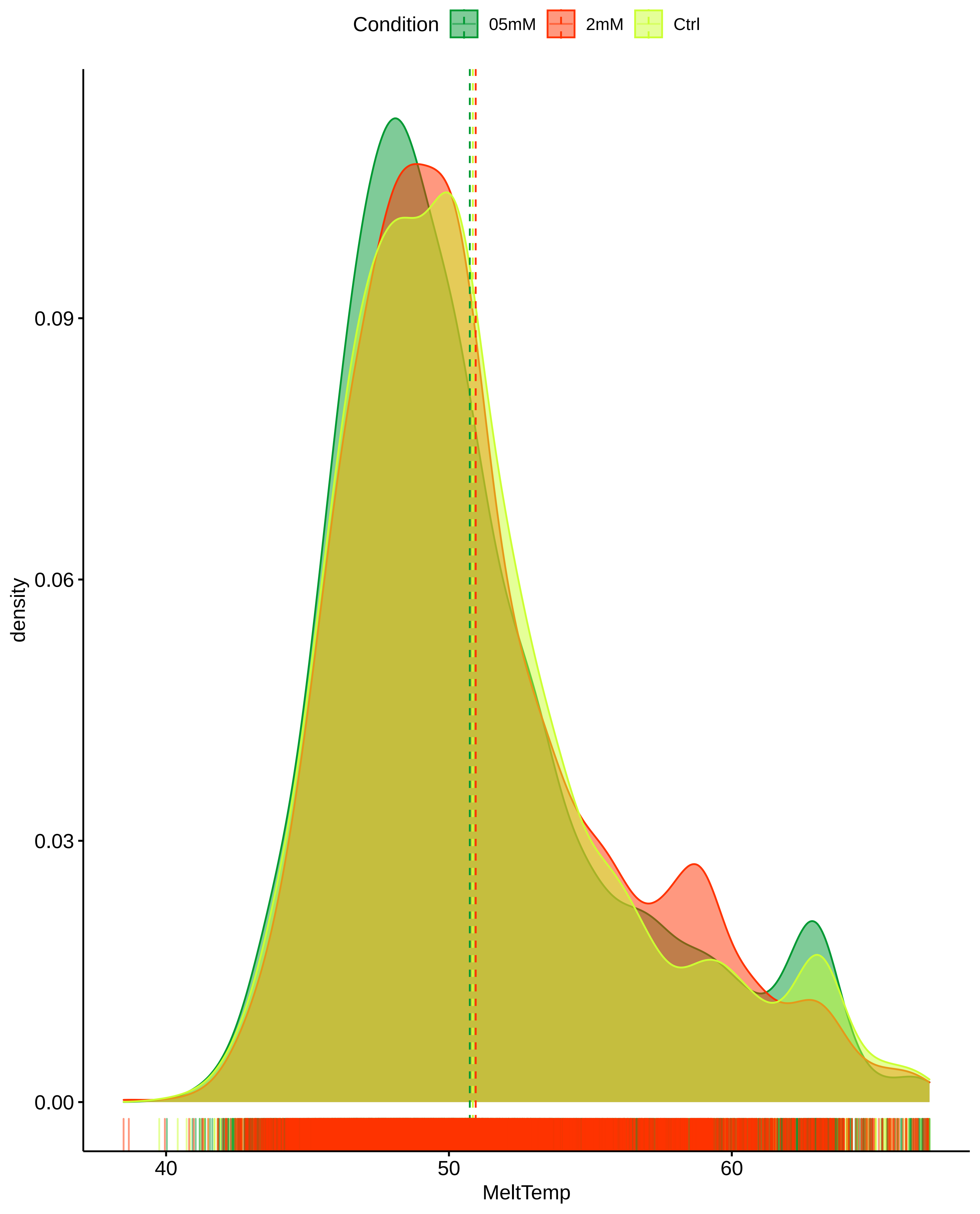

Condition
05mM
2mM
Ctrl
density
0.09
0.06
0.03
0.00
40
50
60
MeltTemp
