# Supplementary material for: Mapping protein carboxymethylation sites provides insights into their role in proteostasis and cell proliferation
Source: Nat Commun. 2021 Nov 18;12:6743. doi: 10.1038/s41467-021-26982-6 (PMC8602705; doi:10.1038/s41467-021-26982-6)
Supplement: Supplementary file 11 — Source Data [file 41467_2021_26982_MOESM11_ESM.zip › FigureS4/4C/201021_DensityPlot_CM_Ctrl_MeltingTemp.pdf]

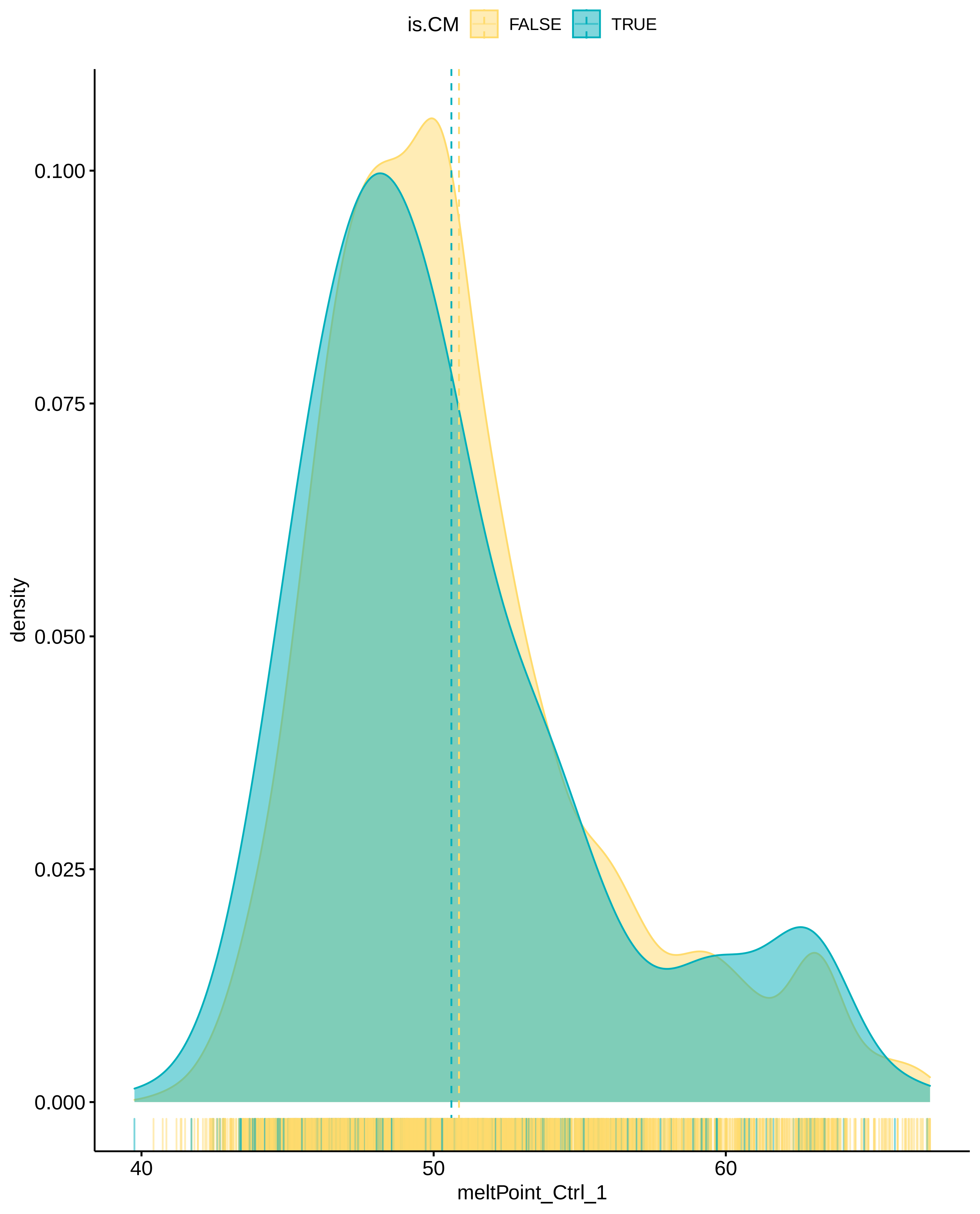

is.CM
FALSE
TRUE
0.100
0.075
0.050
0.025
0.000
density
40
50
60
meltPoint_Ctrl_1
