# Supplementary material for: Mapping protein carboxymethylation sites provides insights into their role in proteostasis and cell proliferation
Source: Nat Commun. 2021 Nov 18;12:6743. doi: 10.1038/s41467-021-26982-6 (PMC8602705; doi:10.1038/s41467-021-26982-6)
Supplement: Supplementary file 11 — Source Data [file 41467_2021_26982_MOESM11_ESM.zip › FigureS4/FigureS4.pdf]

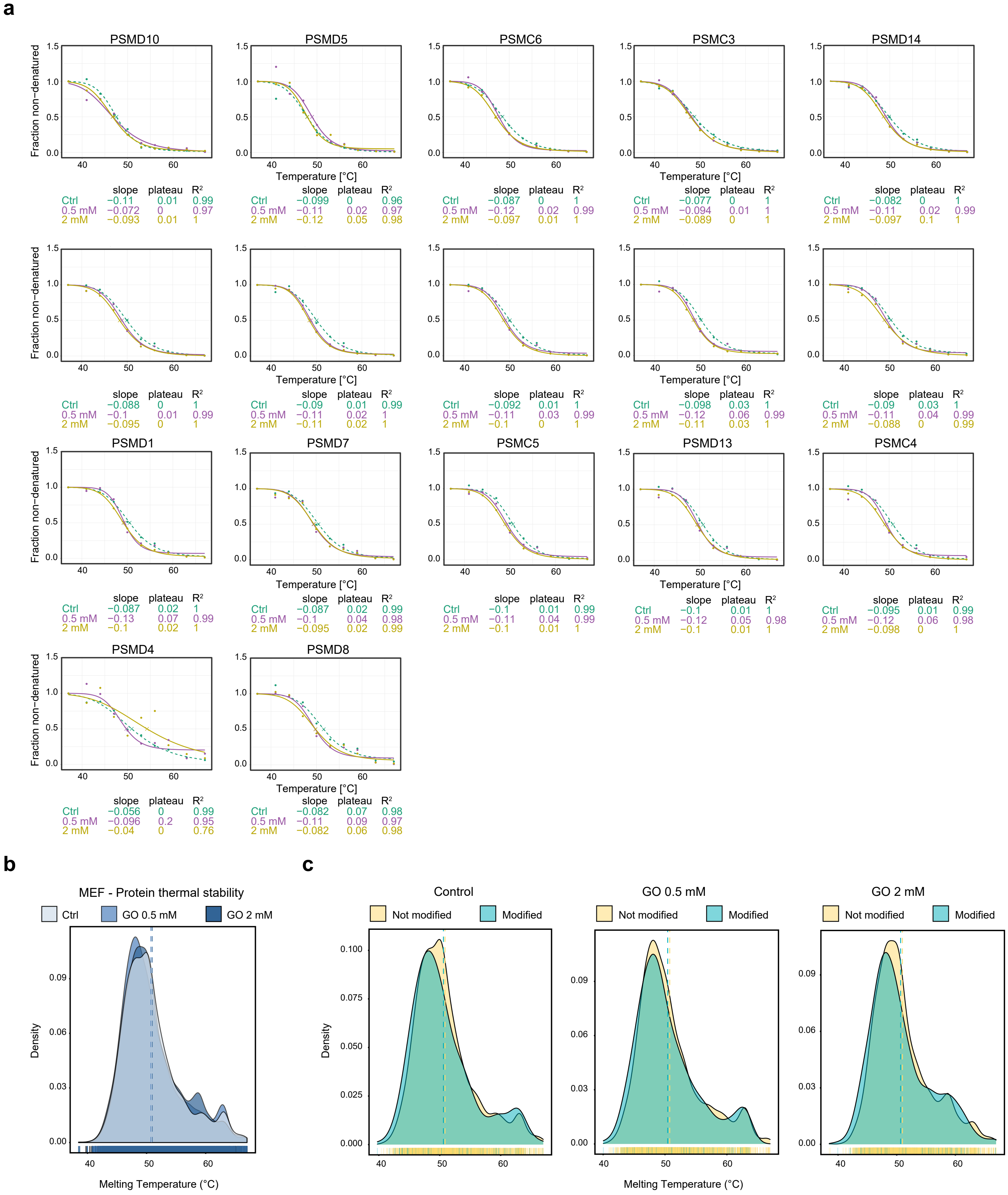
a
PSMD10
Ctrl slope −0.11 plateau 0.01 R² 0.99
0.5 mM slope −0.072 plateau 0 R² 0.97
2 mM slope −0.093 plateau 0.01 R² 1
PSMD5
Ctrl slope −0.099 plateau 0 R² 0.96
0.5 mM slope −0.11 plateau 0.02 R² 0.97
2 mM slope −0.12 plateau 0.05 R² 0.98
PSMC6
Ctrl slope −0.087 plateau 0 R² 1
0.5 mM slope −0.12 plateau 0.02 R² 0.99
2 mM slope −0.097 plateau 0.01 R² 1
PSMC3
Ctrl slope −0.077 plateau 0 R² 1
0.5 mM slope −0.094 plateau 0.01 R² 1
2 mM slope −0.089 plateau 0 R² 1
PSMD14
Ctrl slope −0.082 plateau 0 R² 1
0.5 mM slope −0.11 plateau 0.02 R² 0.99
2 mM slope −0.097 plateau 0.1 R² 1
Ctrl slope −0.088 plateau 0 R² 1
0.5 mM slope −0.1 plateau 0.01 R² 0.99
2 mM slope −0.095 plateau 0 R² 1
Ctrl slope −0.09 plateau 0.01 R² 0.99
0.5 mM slope −0.11 plateau 0.02 R² 1
2 mM slope −0.11 plateau 0.02 R² 1
Ctrl slope −0.092 plateau 0.01 R² 1
0.5 mM slope −0.11 plateau 0.03 R² 0.99
2 mM slope −0.1 plateau 0 R² 1
Ctrl slope −0.098 plateau 0.03 R² 1
0.5 mM slope −0.12 plateau 0.06 R² 0.99
2 mM slope −0.11 plateau 0.03 R² 1
Ctrl slope −0.09 plateau 0.03 R² 1
0.5 mM slope −0.11 plateau 0.04 R² 0.99
2 mM slope −0.088 plateau 0 R² 0.99
PSMD1
Ctrl slope −0.087 plateau 0.02 R² 1
0.5 mM slope −0.13 plateau 0.07 R² 0.99
2 mM slope −0.1 plateau 0.02 R² 1
PSMD7
Ctrl slope −0.087 plateau 0.02 R² 0.99
0.5 mM slope −0.1 plateau 0.04 R² 0.98
2 mM slope −0.095 plateau 0.02 R² 0.99
PSMC5
Ctrl slope −0.1 plateau 0.01 R² 0.99
0.5 mM slope −0.11 plateau 0.04 R² 0.99
2 mM slope −0.1 plateau 0.01 R² 1
PSMD13
Ctrl slope −0.1 plateau 0.01 R² 1
0.5 mM slope −0.12 plateau 0.05 R² 0.98
2 mM slope −0.1 plateau 0.01 R² 1
PSMC4
Ctrl slope −0.095 plateau 0.01 R² 0.99
0.5 mM slope −0.12 plateau 0.06 R² 0.98
2 mM slope −0.098 plateau 0 R² 1
PSMD4
Ctrl slope −0.056 plateau 0 R² 0.99
0.5 mM slope −0.096 plateau 0.2 R² 0.95
2 mM slope −0.04 plateau 0 R² 0.76
PSMD8
Ctrl slope −0.082 plateau 0.07 R² 0.98
0.5 mM slope −0.11 plateau 0.09 R² 0.97
2 mM slope −0.082 plateau 0.06 R² 0.98
Fraction non−denatured
Temperature [°C]
b
MEF - Protein thermal stability
Ctrl
GO 0.5 mM
GO 2 mM
Density
Melting Temperature (°C)
c
Control
GO 0.5 mM
GO 2 mM
Not modified
Modified
Density
Melting Temperature (°C)
