# Supplementary material for: Mapping protein carboxymethylation sites provides insights into their role in proteostasis and cell proliferation
Source: Nat Commun. 2021 Nov 18;12:6743. doi: 10.1038/s41467-021-26982-6 (PMC8602705; doi:10.1038/s41467-021-26982-6)
Supplement: Supplementary file 11 — Source Data [file 41467_2021_26982_MOESM11_ESM.zip › FigureS6/6B/210611_Boxplot_MAP.pdf]

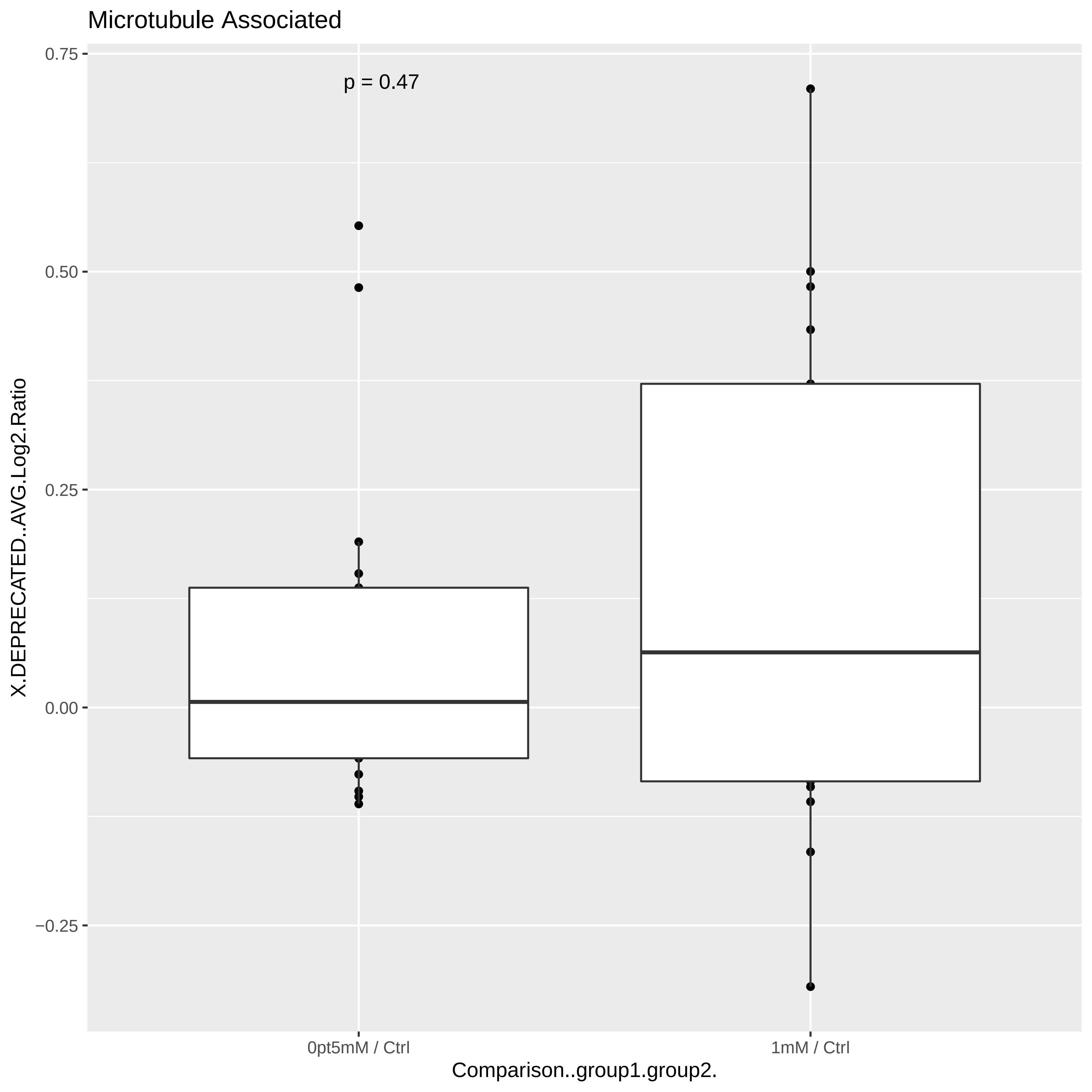

Microtubule Associated
p = 0.47
0.75
0.50
0.25
0.00
−0.25
X.DEPRECATED..AVG.Log2.Ratio
0pt5mM / Ctrl
1mM / Ctrl
Comparison..group1.group2.
