# Supplementary material for: Mapping protein carboxymethylation sites provides insights into their role in proteostasis and cell proliferation
Source: Nat Commun. 2021 Nov 18;12:6743. doi: 10.1038/s41467-021-26982-6 (PMC8602705; doi:10.1038/s41467-021-26982-6)
Supplement: Supplementary file 11 — Source Data [file 41467_2021_26982_MOESM11_ESM.zip › FigureS6/6B/210611_heatmap_MAP.pdf]

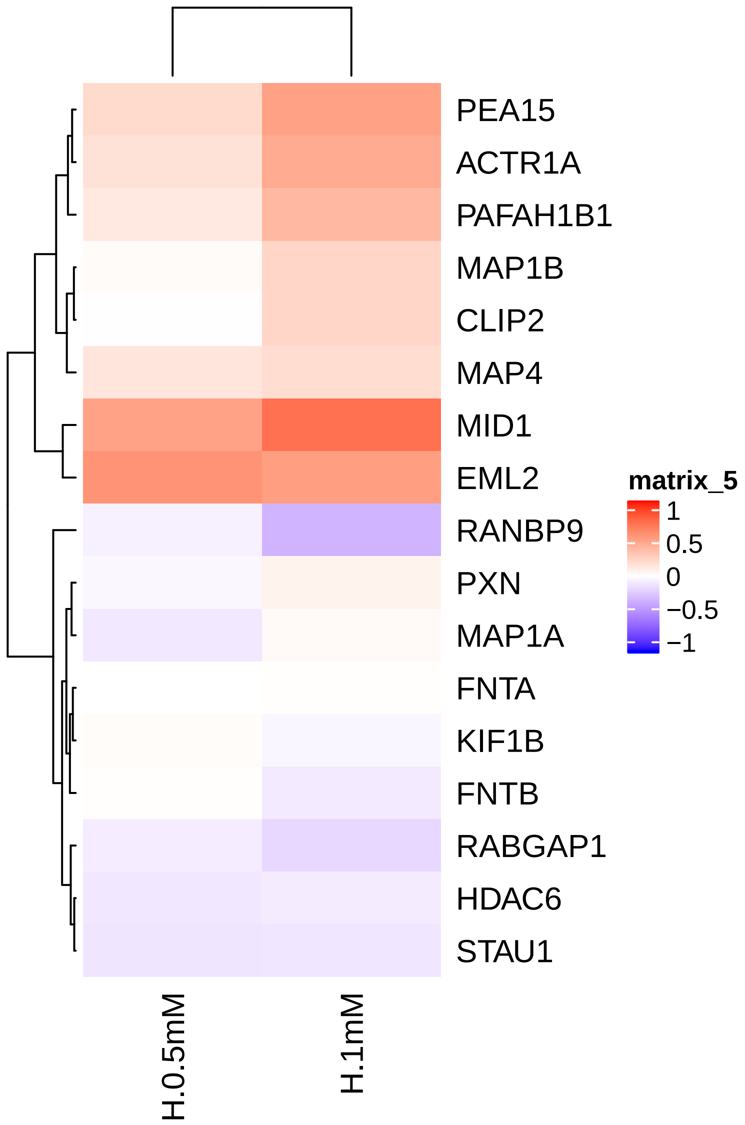
PEA15
ACTR1A
PAFAH1B1
MAP1B
CLIP2
MAP4
MID1
EML2
RANBP9
PXN
MAP1A
FNTA
KIF1B
FNTB
RABGAP1
HDAC6
STAU1
matrix_5
1
0.5
0
−0.5
−1
H.0.5mM
H.1mM
