# Supplementary material for: Mapping protein carboxymethylation sites provides insights into their role in proteostasis and cell proliferation
Source: Nat Commun. 2021 Nov 18;12:6743. doi: 10.1038/s41467-021-26982-6 (PMC8602705; doi:10.1038/s41467-021-26982-6)
Supplement: Supplementary file 11 — Source Data [file 41467_2021_26982_MOESM11_ESM.zip › FigureS6/Fig S6B.pdf]

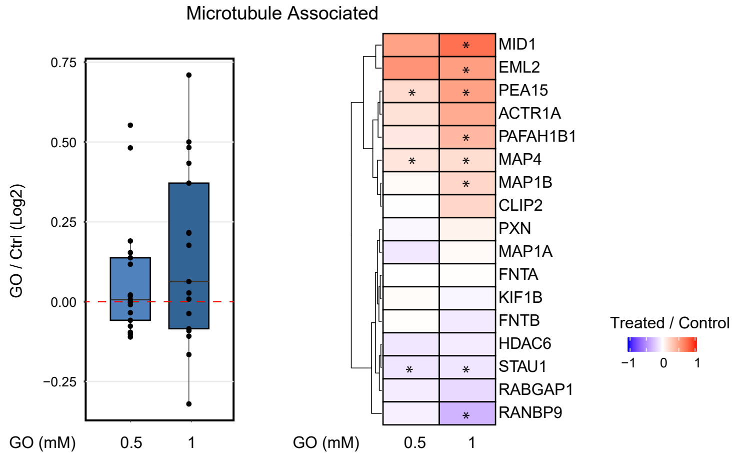
Microtubule Associated
GO / Ctrl (Log2)
0.75
0.50
0.25
0.00
−0.25
GO (mM)
0.5
1
MID1
EML2
PEA15
ACTR1A
PAFAH1B1
MAP4
MAP1B
CLIP2
PXN
MAP1A
FNTA
KIF1B
FNTB
HDAC6
STAU1
RABGAP1
RANBP9
GO (mM)
0.5
1
Treated / Control
−1
0
1
