# Supplementary material for: Mapping protein carboxymethylation sites provides insights into their role in proteostasis and cell proliferation
Source: Nat Commun. 2021 Nov 18;12:6743. doi: 10.1038/s41467-021-26982-6 (PMC8602705; doi:10.1038/s41467-021-26982-6)
Supplement: Supplementary file 11 — Source Data [file 41467_2021_26982_MOESM11_ESM.zip › FigureS7/Fig S7A.pdf]

ID	Gene	Protein Name	CM site
P68363	TUBA1B	Tubulin alpha-1B chain	HUVEC (K394; K401; K163; K164; K326; K336; K338), MEF (K394; K401; K163; K164; K336; K338; K326; K96; K352; K370; K60)
P07437	TUBB	Tubulin beta chain	HUVEC (K379; K58)
P68371	TUBB4B	Tubulin beta-4B chain	HUVEC (K324; K58), MEF (K324; K58; K216; K336), Heart, Kidney (K324)
Q9BUF5	TUBB6	Tubulin beta-6 chain	HUVEC (K379), MEF (K57; K58)
Q13509	TUBB3	Tubulin beta-3 chain	HUVEC (K58)
P99024	TUBB5	Tubulin beta-5 chain	MEF (K379; K58; K362)
