# Supplementary material for: Mapping protein carboxymethylation sites provides insights into their role in proteostasis and cell proliferation
Source: Nat Commun. 2021 Nov 18;12:6743. doi: 10.1038/s41467-021-26982-6 (PMC8602705; doi:10.1038/s41467-021-26982-6)
Supplement: Supplementary file 11 — Source Data [file 41467_2021_26982_MOESM11_ESM.zip › FigureS7/Fig S7B.pdf]

ID Porcine	Sequence	ID Human	Gene	Protein name	CM site Human
Q2XVP4	DVNAAIATIK(1)TK(1)R	P68363	TUBA1B	Tubulin alpha-1B chain	K336; K338
Q2XVP4	FDLMYAK(1)R	P68363	TUBA1B	Tubulin alpha-1B chain	K401
Q2XVP4	GDVVPK(1)DVNAAIATIK(1)TK	P68363	TUBA1B	Tubulin alpha-1B chain	K336; K326
Q2XVP4	LDHK(1)FDLMYAK	P68363	TUBA1B	Tubulin alpha-1B chain	K394
Q2XVP4	LDHK(1)FDLMYAK(1)R	P68363	TUBA1B	Tubulin alpha-1B chain	K401; K394
Q2XVP4	LSVDYGK(1)K(1)SK	P68363	TUBA1B	Tubulin alpha-1B chain	K163; K164
Q767L7	ISVYYNEATGGK(1)YVPR	P07437	TUBB	Tubulin beta chain	K58
Q767L7	MAVTFIGNSTAIQELFK(1)R	P07437	TUBB	Tubulin beta chain	K379
A0A5G2R693	ISVYYNEASSHK(1)YVPR	Q13509	TUBB3	Tubulin beta-3 chain	K58
A0A287A275	INVYYNEATGGK(1)YVPR	P68371	TUBB4B	Tubulin beta-4B chain	K58
P02554	MSMK(1)EVDEQMLNVQNK	P68371	TUBB4B	Tubulin beta-4B chain	K324
