# Supplementary material for: Mapping protein carboxymethylation sites provides insights into their role in proteostasis and cell proliferation
Source: Nat Commun. 2021 Nov 18;12:6743. doi: 10.1038/s41467-021-26982-6 (PMC8602705; doi:10.1038/s41467-021-26982-6)
Supplement: Supplementary file 11 — Source Data [file 41467_2021_26982_MOESM11_ESM.zip › FigureS8/8A/Huvec_GO_LongTerm_newPCA.pdf]

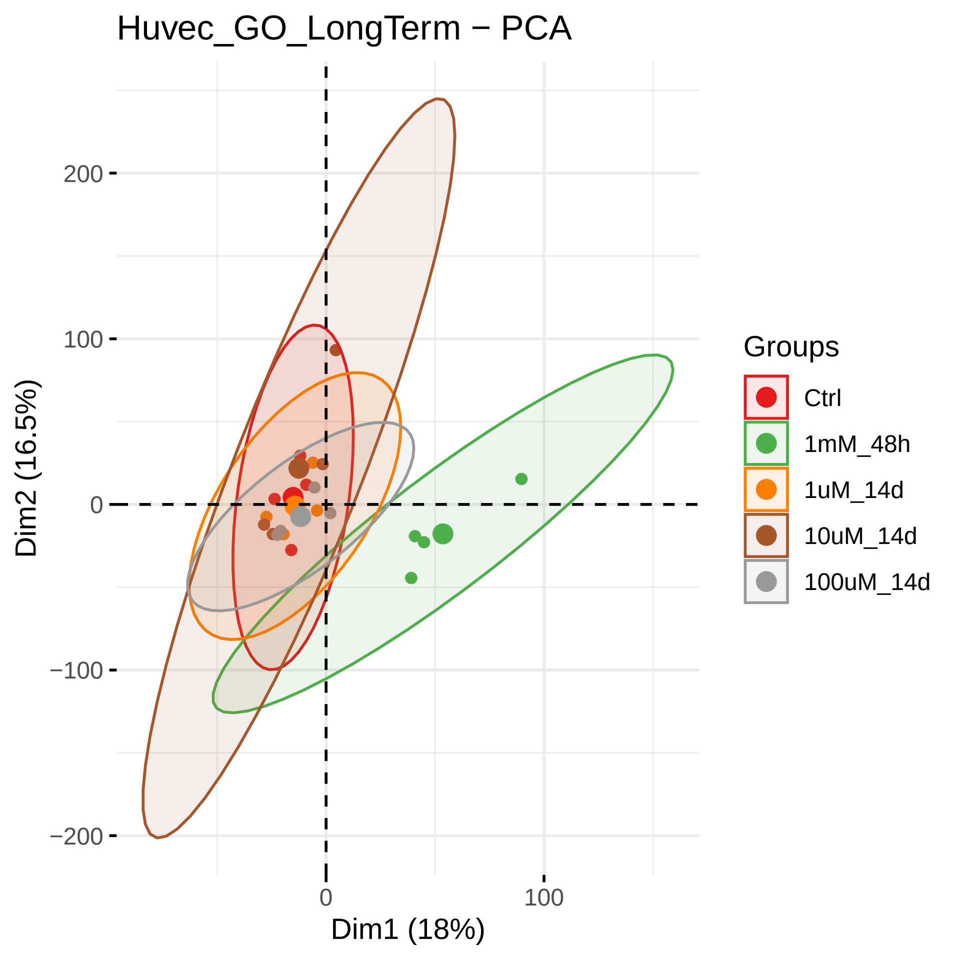

Huvec_GO_LongTerm − PCA
200
100
0
−100
−200
0
100
Dim1 (18%)
Dim2 (16.5%)
Groups
Ctrl
1mM_48h
1uM_14d
10uM_14d
100uM_14d
