# Supplementary material for: Mapping protein carboxymethylation sites provides insights into their role in proteostasis and cell proliferation
Source: Nat Commun. 2021 Nov 18;12:6743. doi: 10.1038/s41467-021-26982-6 (PMC8602705; doi:10.1038/s41467-021-26982-6)
Supplement: Supplementary file 11 — Source Data [file 41467_2021_26982_MOESM11_ESM.zip › FigureS8/8B/210624_Heatmap_CellCycle_proteins_LT_GO_vs_ST_GO_w_Hmox1.pdf]

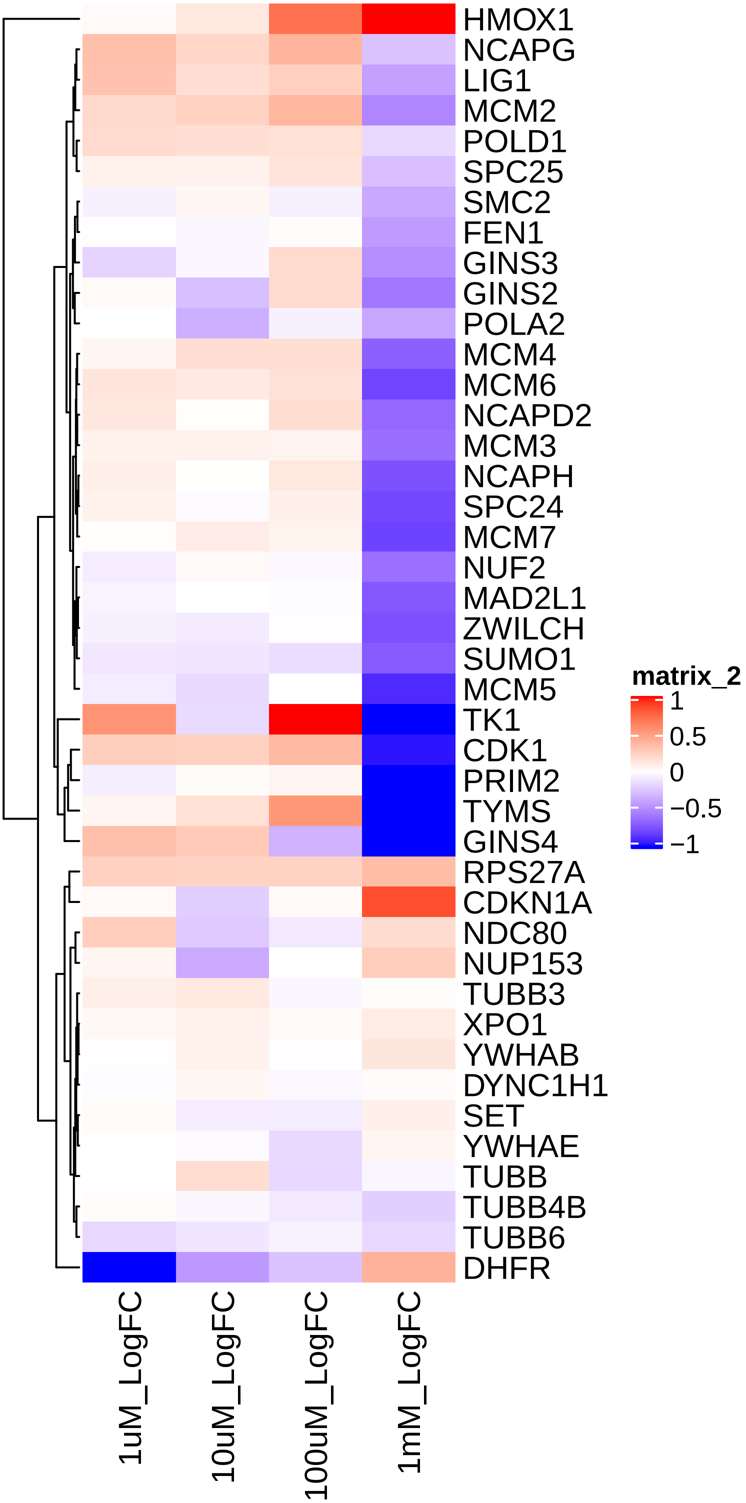
HMOX1
NCAPG
LIG1
MCM2
POLD1
SPC25
SMC2
FEN1
GINS3
GINS2
POLA2
MCM4
MCM6
NCAPD2
MCM3
NCAPH
SPC24
MCM7
NUF2
MAD2L1
ZWILCH
SUMO1
MCM5
TK1
CDK1
PRIM2
TYMS
GINS4
RPS27A
CDKN1A
NDC80
NUP153
TUBB3
XPO1
YWHAB
DYNC1H1
SET
YWHAE
TUBB
TUBB4B
TUBB6
DHFR
1uM_LogFC
10uM_LogFC
100uM_LogFC
1mM_LogFC
matrix_2
1
0.5
0
−0.5
−1
